# Targeting FADS1-mediated lipid metabolism and signaling: a novel therapeutic strategy for precision oncology in colorectal and esophageal cancers

**DOI:** 10.1038/s41420-025-02768-3

**Published:** 2025-10-16

**Authors:** Jingxuan Lian, Xiaohui Duan, Wenjie Chen, Xinhong Zhang, Ming Lu, Zheshen Lin, Zhentian Wu, Litian Ma, Rong Liang

**Affiliations:** 1 Department of hematology, Xijing Hospital, Air Force Medical University, Xi’an, Shaanxi China; 2 Department of Traditional Chinese Medicine, Tangdu Hospital, Fourth Military Medical University, Xi’an, Shaanxi China; 3 Department of Thoracic Surgery, Tangdu Hospital, Fourth Military Medical University, Xi’an, Shaanxi China; 4 Department of Gastroenterology, Tangdu Hospital, Fourth Military Medical University, Xi’an, Shaanxi China

**Keywords:** Cancer, Gastrointestinal cancer

## Abstract

Gastrointestinal (GI) cancers exhibit aberrant lipid metabolism, yet the causal mechanisms remain elusive. Here, we integrated Mendelian randomization (MR) and multi-omics data to dissect metabolic drivers of 20 GI diseases. Focusing on colorectal (CC) and esophageal cancer (EC), we identified five metabolites (e.g., 1,2-di-palmitoyl-sn-glycero-3-phosphocholine) and arachidonic acid ethyl ester as causal drivers. Summary-data-based MR and colocalization analysis (PP.H4 > 0.75) revealed FADS1 as a master regulator of these metabolites, with genetic variants exhibiting tissue-specific lipidomic effects. Functional validation using FADS1-knockout cell lines and mouse models demonstrated that FADS1 inhibition suppresses tumor cell proliferation, migration, and invasion while promoting apoptosis. In vivo, FADS1 deletion reduced chemically induced CC/EC tumor burden by 62–75%, accompanied by decreased Ki-67/MMP-9 expression and inflammatory infiltration. Mechanistically, FADS1 ablation disrupted lipid metabolism (reduced linoleic acid and arachidonic acid) and attenuated PI3K/AKT and MAPK signaling. Multi-omics integration further corroborated FADS1-mediated epigenetic regulation (e.g., mQTL-driven DNA methylation). This study establishes FADS1 as a pivotal orchestrator of GI carcinogenesis via metabolic reprogramming and signaling dysregulation, offering a compelling therapeutic target for precision oncology in CC and EC.

**Figure:**
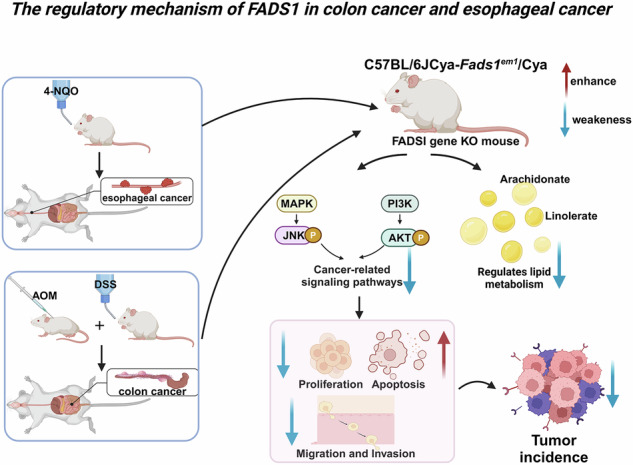
Regulatory mechanisms of FADS1 in CC and EC.

Regulatory mechanisms of FADS1 in CC and EC.

## Introduction

Metabolites, as intermediates or end products of metabolic reactions [1, 2], are modulated by genetic predisposition, dietary patterns, gut microbiota composition, and disease states [3, 4], and have been implicated in the pathogenesis of gastrointestinal (GI) diseases [5]. Advances in metabolomics have facilitated the identification of disease biomarkers and potential therapeutic targets [6]. Altered alpha-1-acid glycoprotein (AAG) metabolic pathways have been linked to gastric cancer [7–9], and identified distinct metabolite profiles in esophageal malignancies [10]. However, such observational findings are prone to residual confounding and reverse causality [11, 12], limiting causal inference.

Mendelian randomization (MR), a method that utilizes genetic variants as instrumental variables (IVs) to infer causal relationships between exposures and outcomes [13–15], offers an approach to overcome these limitations by exploiting the random allocation of alleles at conception [16]. Two-sample MR enables the evaluation of exposure–outcome relationships across independent populations [17]. Summary data-based Mendelian randomization (SMR) further extends this framework by integrating genome-wide association study (GWAS) data with quantitative trait loci (QTL) datasets to prioritize candidate causal genes [18–20]. To distinguish true pleiotropy from confounding due to linkage disequilibrium (LD), the heterogeneity in dependent instruments (HEIDI) test is implemented within the SMR framework [21].

This study employs MR and multi-omics integration to dissect causal metabolite-disease networks in 20 GI diseases, prioritizing FADS1’s mechanistic role in colorectal and esophageal cancers (ECs). Through FADS1 knockout models, we validate its functional impact on tumor progression, offering novel insights for precision oncology and therapeutic target development.

## Results

### MR analysis based on metabolomics and its association with GI diseases

**Fig. 1:**
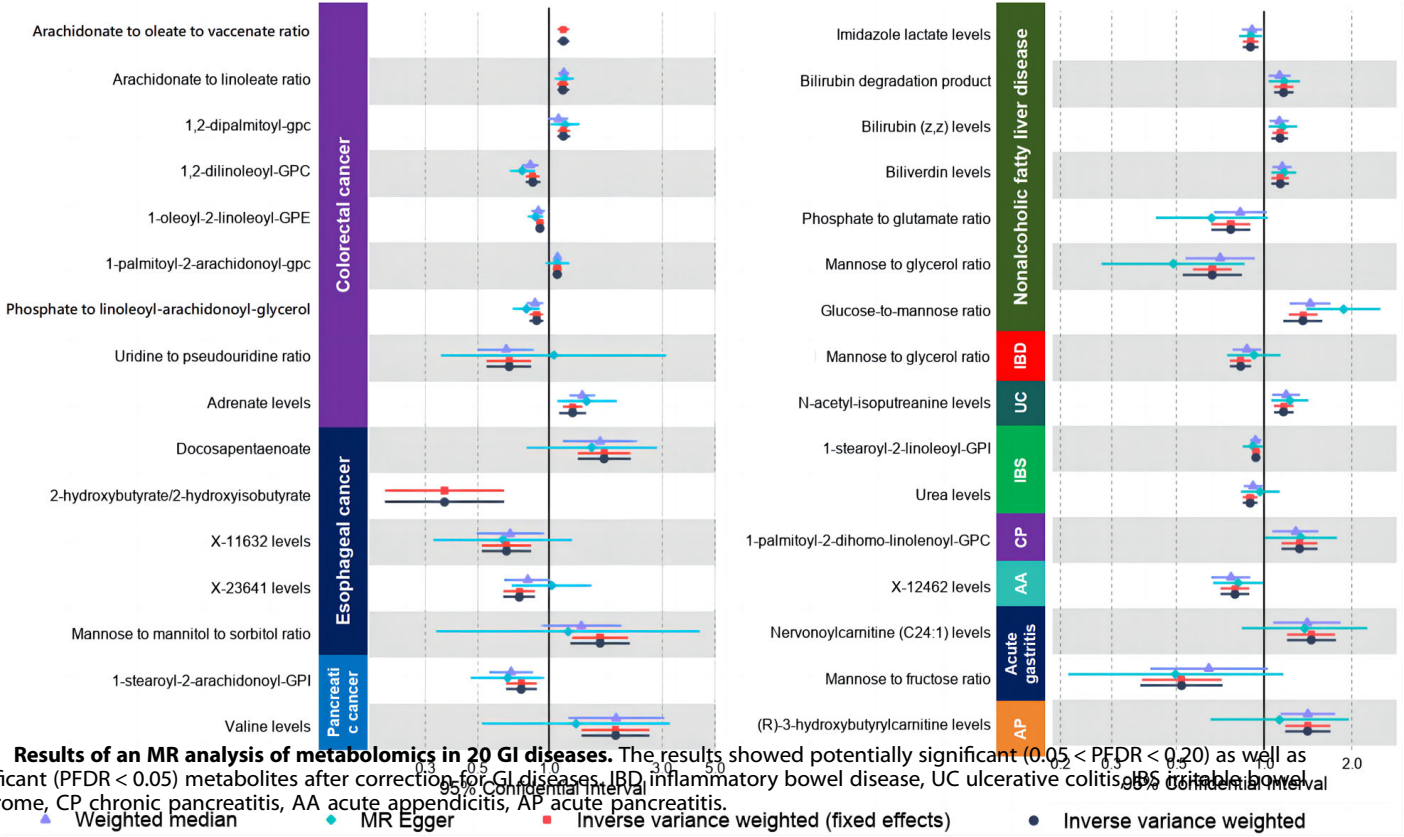
Results of an MR analysis of metabolomics in 20 GI diseases. The results showed potentially significant (0.05 < PFDR < 0.20) as well as significant (PFDR < 0.05) metabolites after correction for GI diseases. IBD inflammatory bowel disease, UC ulcerative colitis, IBS irritable bowel syndrome, CP chronic pancreatitis, AA acute appendicitis, AP acute pancreatitis.

Among 20 GI diseases, no significant metabolites (PFDR > 0.20) were identified in liver cancer, gastric cancer, GERD, chronic gastritis, gastric/duodenal ulcers, diverticulosis, or Crohn’s disease (Fig. 1). In colorectal cancer (CC), five metabolites showed robust associations: phosphatidylethanolamine ratios to stearoyl-oleoyl-glycerol (OR = 1.15, 95%CI 1.08–1.22, *p* = 1.05 × 10^−6^) and linoleoyl-glycerol (OR = 1.14, 95%CI 1.07–1.21, *p* = 8.31 × 10^−6^), 1,2-di-palmitoyl-GPC (OR = 1.14, *p* = 2.00 × 10^−5^), 1,2-di-linoleoyl-GPC (OR = 0.85, *p* = 2.34 × 10^−5^), and 1-oleoyl-2-linoleoyl-GPE (OR = 0.91, *p* = 2.34 × 10^−5^). EC exhibited one significant association with arachidonic acid ethyl ester (OR = 1.70, 95%CI 1.59–1.82, *p* = 5.38 × 10^−5^). Non-alcoholic fatty liver disease (NAFLD) linked bilirubin degradation products (OR = 1.16, *p* = 6.59 × 10^−5^) and glucose-to-mannose ratio (OR = 1.36, *p* = 8.83 × 10^−5^) to disease risk. Detailed associations for other GI diseases are provided in Table S2. Sensitivity analyses confirmed robustness across MR methods (Fig. S1) and ruled out horizontal pleiotropy (p-MR-Egger > 0.05; Table S3), with NAFLD showing global pleiotropy despite outlier removal (Table S4).

### MR analysis of eQTLs for multiple metabolites associated with cancer prognosis

**Fig. 2:**
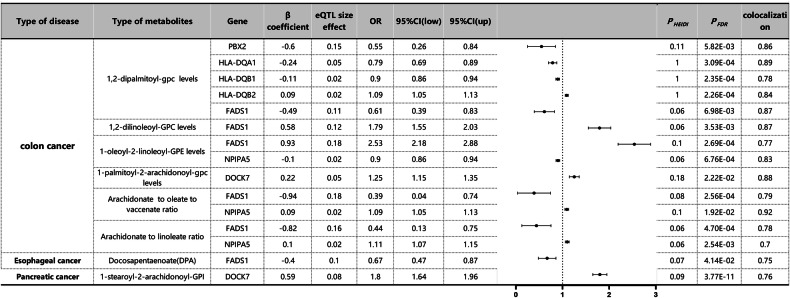
SMR and colocation outcomes of eQTLs of multiple metabolites with causal association with cancer prognosis. β > 0 indicates a positive correlation, and β < 0 indicates a negative correlation. Ratios are calculated from expected values (β coefficients) for causal effect estimates. Colocation is determined by PP.H4 between eQTLs and cancer prognosis, where the PP.H4 threshold >0.75 is considered strong evidence of colocation. The results are limited to those with a PP.H4 value of 0.70 or higher.

Through SMR analysis, we identified causal associations between blood metabolites influencing diverse cancer prognoses and outcomes (Fig. 2). To control type I errors across the genome, we performed multiple testing corrections to determine significant correlations (PFDR < 0.05, Benjamini–Hochberg correction). Subsequently, HEIDI tests were conducted using the SMR software (PHEIDI > 0.05) to investigate whether this correlation was driven by shared causal variants rather than pleiotropy. Consequently, we identified seven genes associated with six metabolites in CC, with genes related to AA ethyl ester level only found in EC and genes linked to 1-stearoyl-2-arachidonoyl-GPI level exclusively discovered in pancreatic cancer. Additional colocalization analysis was conducted to mitigate the potential confounding effects of LD. Posterior probabilities (PP.H4) greater than 0.75 indicated clear colocalization evidence between cancer GWAS and eQTL sites. Causal effect estimates were represented as beta coefficients, and odds ratios (OR) were calculated based on the expected beta coefficients, as shown in Fig. 2 and Table S5. In CC, FADS1 was identified as a significant risk or protective factor for all five metabolites, particularly in 1,2-di-palmitoyl-sn-glycero-3-phosphocholine (OR 1.79, 95%CI 1.55–2.03, PSMR = 3.53 × 10^−3^) and 1,2-di-linoleoyl-glycero-3-phosphocholine levels (OR 2.53, 95%CI 2.18–2.88, PSMR = 2.69 × 10^−4^). Furthermore, NPIPA5 exhibited similar associations with three metabolites (1-oleoyl-2-linoleoyl-glycero-3-phosphoethanolamine level, OR 0.90, 95%CI 0.86–0.94, PSMR = 6.76 × 10^−4^; phosphatidyllethanolamine to stearoyl-oleoyl-glycerol ratio, OR 1.09, 95%CI 1.05–1.13, PSMR = 1.92 × 10^−2^; phosphatidyllethanolamine to linoleoyl-glycerol ratio, OR 1.11, 95%CI 1.07–1.15, PSMR = 2.54 × 10^−3^). For EC, FADS1 displayed a protective role in its development (AA ethyl ester level, OR 0.67, 95%CI 0.47–0.87, PSMR = 4.14 × 10^−2^). Conversely, DOCK7 showed a significant increase in pancreatic cancer risk (1-stearoyl-2-arachidonoyl-glycero-3-phosphoinositol level, OR 1.80, 95%CI 1.64–1.96, PSMR = 3.77 × 10^−11^). Additionally, the remaining results were categorized as related to intestinal diseases and other digestive system disorders and analyzed using SMR to identify risk and protective factors associated with these specific outcomes. Refer to Tables S6 and S7 and Figs. S2 and S3 for detailed results.

### Causal associations between various metabolites and cancer prognosis revealed by MR analysis based on multiple metabolite QTLs

**Fig. 3:**
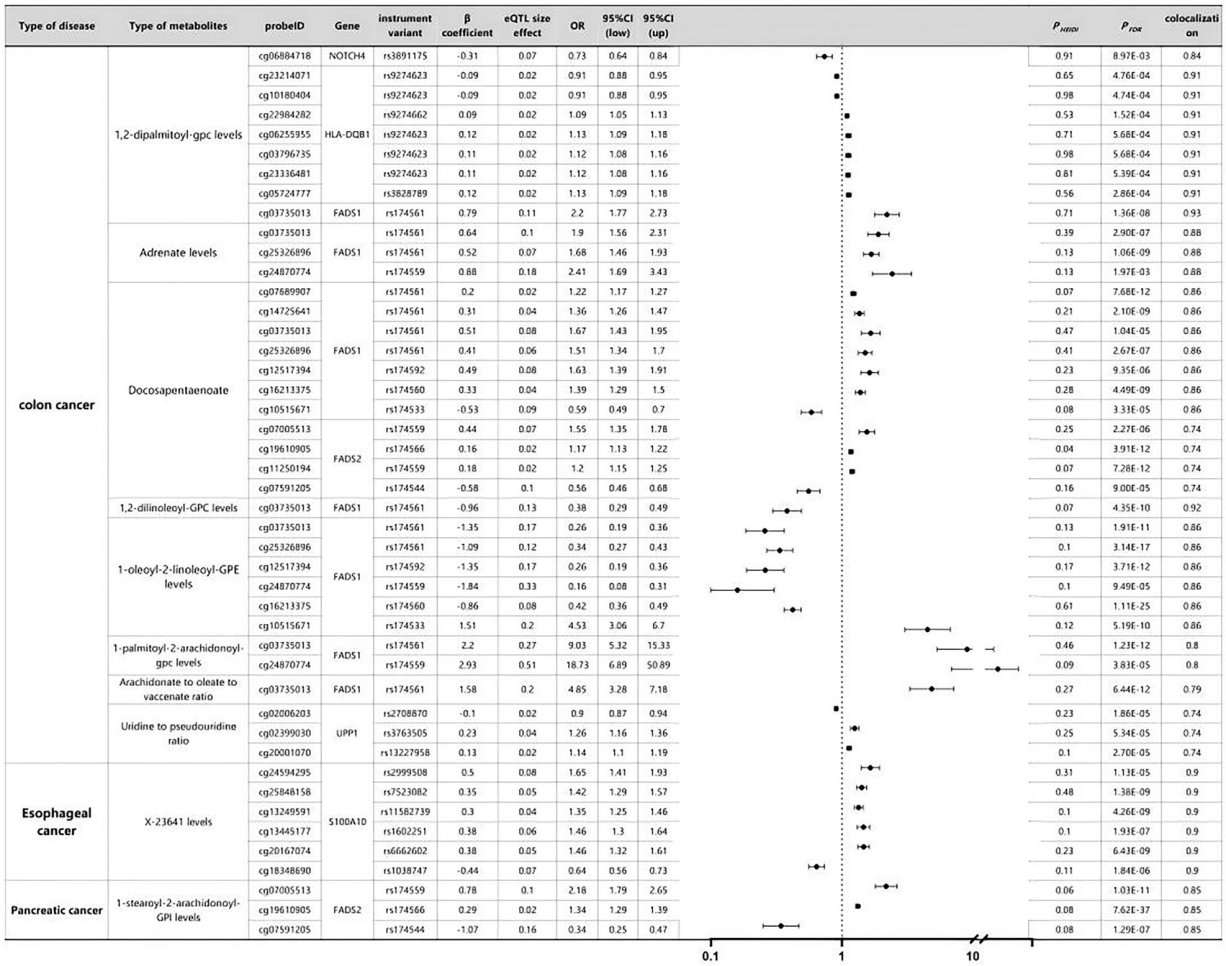
SMR and colocation outcomes of mQTLs of multiple metabolites with causal association with cancer prognosis. β > 0 indicates a positive correlation, and β < 0 indicates a negative correlation. The ratio is calculated based on the causal estimate’s expected value (β coefficient). Colocation was determined by PP.H4 between eQTLs and cancer prognosis, with PP.H4 thresholds greater than 0.75 considered strong evidence of colocation. The results shown are limited to those with PP. Results with an H4 value greater than 0.70.

We conducted Benjamini–Hochberg correction (FDR < 0.05) and HEIDI tests to investigate the causal relationship between metabolite DNA methylation and cancer prognosis. We identified 23 association signals related to CC, comprising 14 specific loci associated with eight metabolites, 6 signals related to EC involving 6 specific loci (at X-23641 level), and 3 signals associated with pancreatic cancer involving 3 specific loci (at 1-stearoyl-2-arachidonyl-GPI level) (Fig. 3 and Table S8). Colocalization analysis revealed that different genetic variations in FADS1 had varying effects on methylation levels, consequently influencing outcomes. For instance, a decrease of one standard deviation in FADS1 methylation level induced by rs6682266, associated with a 1-oleic-2-linoleoyl-GPE level, significantly reduced the CC risk by 58% (OR: 0.42, 95% CI: 0.36–0.49, FDR: 1.11 × 10^−25^); conversely, an increase in FADS1 methylation level caused by rs174533 significantly increased the CC risk by 353% (OR: 4.53, 95% CI: 3.06–6.70, FDR: 5.19 × 10^−10^). We observed that different CpG sites regulating FADS1 exhibited distinct methylation patterns among various metabolites (Fig. 3). Furthermore, particularly at the 1-palmitoyl-2-arachidonyl-GPC level, rs174561 (OR: 9.03, 95% CI: 5.32–15.33, FDR: 1.23 × 10^−12^) and rs174559 (OR: 18.83, 95% CI: 6.89–50.98, FDR: 3.83 × 10^−5^) significantly increased the risk of CC. Five loci were positively associated with EC risk in EC, while one locus showed a negative association. In pancreatic cancer, two loci were positively correlated with pancreatic cancer risk, and one locus exhibited a negative correlation. Additionally, we identified divergent results concerning other GI diseases and their respective specific loci and association signals, detailed in Tables S9 and S10 and Figs. S4 and S5.

### Results of SMR analysis revealing the relationship between DNA methylation and causal metabolite-related gene expression

Moreover, the impact of gene methylation on gene expression has been well established. Therefore, we conducted SMR analysis to explore the causal relationship between metabolite-related gene methylation and gene expression by integrating genetic variants shared between methylation and expression. Following multiple testing corrections and HEIDI tests, we compiled a comprehensive gene list containing DNA methylation CpG sites of metabolite-related genes (Table S11). The SMR results indicated a significant association between NSUN4 methylation, regulated by rs174560 and NSUN4 expression. Furthermore, the methylation of UPP1 modulated by rs7459020, rs3763505, and rs9639018 was related to UPP1 expression. Additionally, PBX2 expression was influenced by rs2760981.

### MR analysis based on eQTLs revealed a causal role of various metabolites in the prognosis of GI diseases

**Fig. 4:**
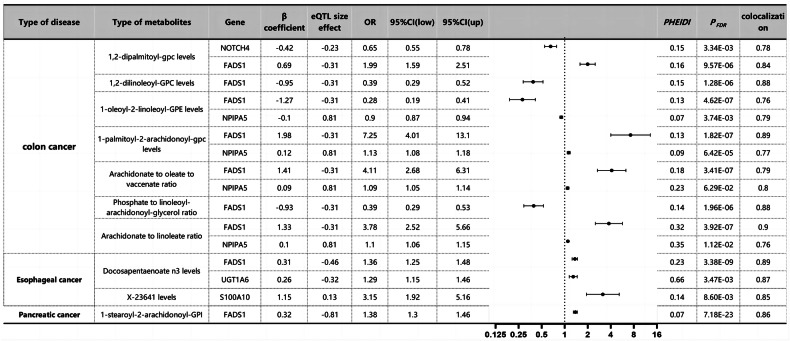
Multiple metabolites with causal associations with cancer prognosis correspond to SMR and colocation outcomes of tissue eQTLs. β > 0 indicates a positive correlation, and β < 0 indicates a negative correlation. The ratio is calculated based on the causal estimate’s expected value (β coefficient). Colocation was determined by PP.H4 between eQTLs and cancer prognosis, with a PP.H4 threshold greater than 0.75 considered strong evidence of colocation. The results shown are limited to those with PP. Results with an H4 value greater than 0.70.

Subsequently, we used SMR analysis to examine tissue eQTL data related to various GI diseases (Tables S12–S14). Interestingly, as depicted in Fig. 4, among numerous associated metabolites, FADS1 and NPIP5 exhibited significant causal associations with CC. Moreover, NOTCH4 (OR: 0.65, 95% CI: 0.55–0.78, FDR: 3.34 × 10^−3^) was found to be associated with CC, and analysis of blood mQTL data revealed a significant correlation between NOTCH4 methylation level and CC. For EC, we identified FADS1 and UGT1A6 as additional risk factors for this disease. Furthermore, after SMR analysis of tissue eQTL data, S100A10 (OR: 3.15, 95% CI: 1.92–5.16, FDR: 8.60 × 10^−3^) exhibited a significant relationship with EC at the gene methylation level. Importantly, previous studies did not uncover an association between FADS1 and pancreatic cancer. NRBP1 was shown to have a significant association with IBD, as depicted in Fig. S6. In NAFLD, four metabolites revealed three genes related to the disease, while UC showed three genes. CP exhibited a significant association with the sole gene indicated in Tables S13 and S14.

### Integration of multi-omics evidence

Upon integrating evidence across multiple omics levels, we identified significant associations between FADS1 and four metabolites in CC using different QTL datasets (blood mQTL, blood eQTL, and tissue eQTL), categorizing them as primary evidence according to Table 1. Noteworthy is the consistent directional effect of FADS1 in both blood mQTL and tissue eQTL analyses; however, its directional effect in blood eQTL analysis is the opposite. Additionally, NOTCH4 exhibited significant correlations in blood mQTL and tissue eQTL analyses, maintaining a consistent directional effect. Similarly, NPIPA5 consistently showed directional effects in both blood eQTL and tissue eQTL analyses. Furthermore, our study also revealed secondary evidence associated with EC (FADS1, S100A10), NRBP1, IBS (TOR1A), and NAFLD (USP40, CCBL2).

**Table 1:** Candidate genes associated with various gastrointestinal diseases, including their predicted methylation and expression patterns.

Type of disease	Type of metabolites	Gene	mQTL from blood				eQTL from blood			eQTL from tissue		
			Probe	OR	95% CI (low)	95% CI (up)	OR	95% CI (low)	95%CI (up)	OR	95% CI (low)	95% CI (up)
**Colon cancer**	1,2-dipalmitoyl-gpc levels	FADS1	cg03735013	2.2	1.77	2.73	0.61	0.39	0.83	1.99	1.59	2.51
		NOTCH4	cg06884718	0.73	0.64	0.84				0.65	0.55	0.78
	1,2-dilinoleoyl-GPC levels	FADS1	cg03735013	0.38	0.29	0.49	1.79	1.55	2.03	0.39	0.29	0.52
	1-oleoyl-2-linoleoyl-GPE levels	FADS1	cg03735013	0.26	0.19	0.36	2.53	2.18	2.88	0.28	0.19	0.41
			cg25326896	0.34	0.27	0.43						
			cg12517394	0.26	0.19	0.36						
			cg24870774	0.16	0.08	0.31						
			cg16213375	0.42	0.36	0.49						
			cg10515671	4.53	3.06	6.7						
		NPIPA5					0.9	0.86	0.94	0.9	0.87	0.94
	Arachidonate to oleate to vaccenate ratio	FADS1	cg03735013	4.85	3.28	7.18	0.39	0.04	0.74	4.11	2.68	6.31
		NPIPA5					1.09	1.05	1.13	1.09	1.05	1.14
	1-palmitoyl-2-arachidonoyl-gpc levels	FADS1	cg03735013	9.03	5.32	15.33				7.25	4.01	13.1
			cg24870774	18.73	6.89	50.89						
	Arachidonate to linoleate ratio	FADS1					0.44	0.13	0.75	3.78	2.52	5.66
		NPIPA5					1.11	1.07	1.15	1.1	1.06	1.15
**Esophageal cancer**	Docosapentaenoate n3 levels	FADS1					0.67	0.47	0.87	1.36	1.25	1.48
	X-23641 levels	S100A10	cg24594295	1.65	1.41	1.93				3.15	1.92	5.16
			cg25848158	1.42	1.29	1.57						
			cg13249591	1.35	1.25	1.46						
			cg13445177	1.46	1.3	1.64						
			cg20167074	1.46	1.32	1.61						
			cg18348690	0.64	0.56	0.73						
**IBD**	Mannose to glycerol ratio	NRBP1	cg15478930	1.42	1.21	1.66				0.64	0.54	0.75
**IBS**	Urea levels	TOR1A					0.77	0.67	0.88	0.79	0.68	0.9
**Non-alcoholic liver disease**	Bilirubin levels	USP40					1.54	1.34	1.76	0.71	0.62	0.81
	Biliverdin levels	USP40					1.48	1.31	1.66	0.73	0.65	0.83
	Imidazole lactate levels	CCBL2	cg14669130	0.88	0.84	0.91	0.22	0.17	0.28			
			cg11309454	0.9	0.87	0.94						
			cg23627354	0.9	0.86	0.93						

*IBD* inflammatory bowel disease, *IBS* irritable bowel syndrome.

### Expression analysis of FADS1 in CC and EC

**Fig. 5:**
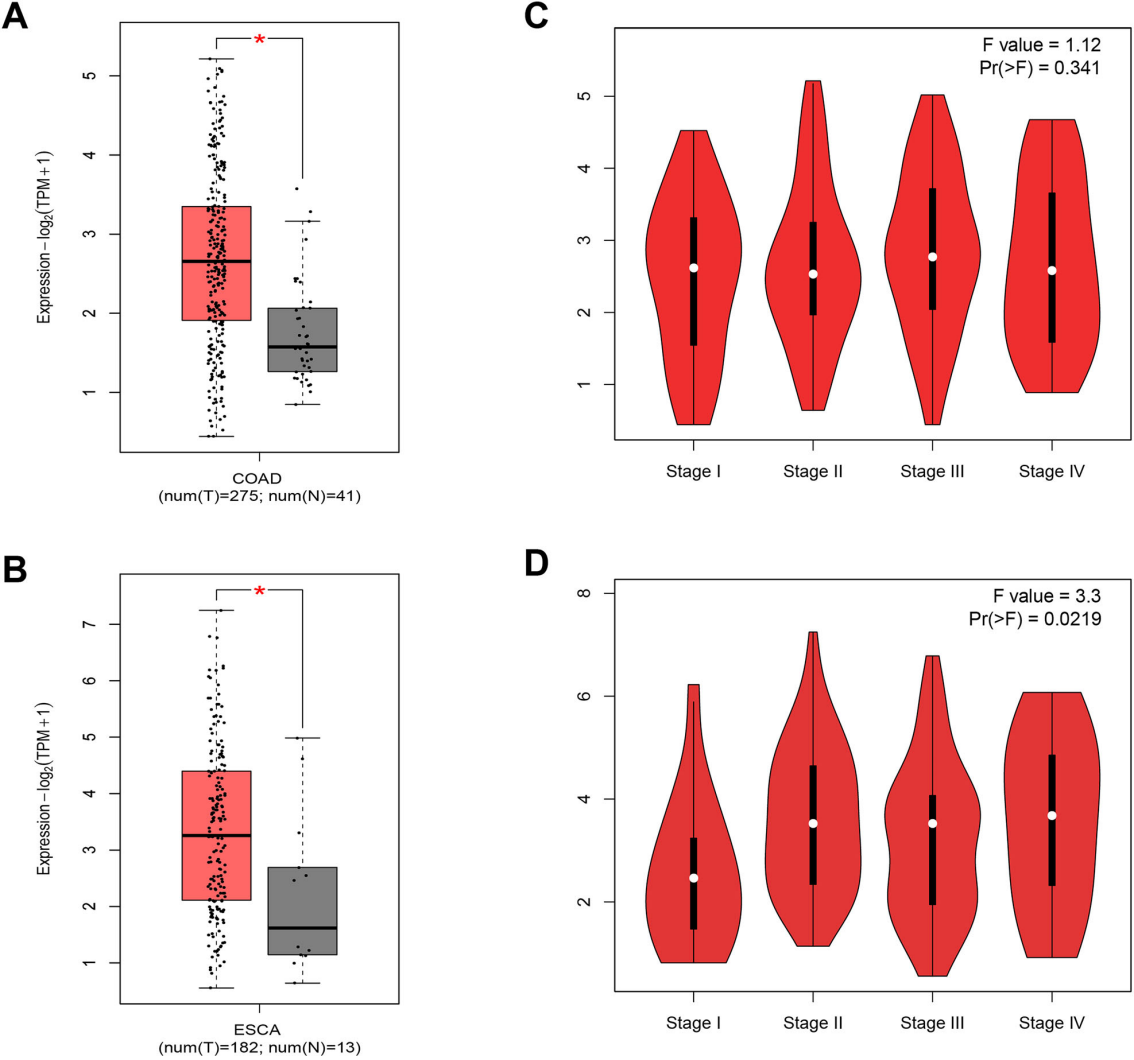
Expression and prognosis of FADS1 in CC and EC. **A** Gene expression levels of FADS1 in CC and normal tissue samples; **B** Gene expression levels of FADS1 in EC and normal tissue samples; **C** Changes in the expression of FADS1 in different stages of CC; **D** Changes in the expression of FADS1 in different stages of EC, indicating that FADS1 is gradually upregulated during cancer development.

FADS1 expression levels were analyzed using transcriptomic data from TCGA. Compared to normal tissues, FADS1 expression was significantly upregulated in both CC and EC tissues (Fig. 5A, B). In CC, the average expression level was approximately 1.5-fold higher than in normal tissue, while in EC, it was nearly twofold higher (*p* < 0.001). Across clinical stages, FADS1 expression showed a progressive increase in EC, with the highest levels observed in stage IV (Fig. 5D). In contrast, although FADS1 expression varied slightly across CC stages, no statistically significant differences were detected (Fig. 5C).

### Regulation of CC and EC cell proliferation, migration, invasion, and cell apoptosis by FADS1

**Fig. 6:**
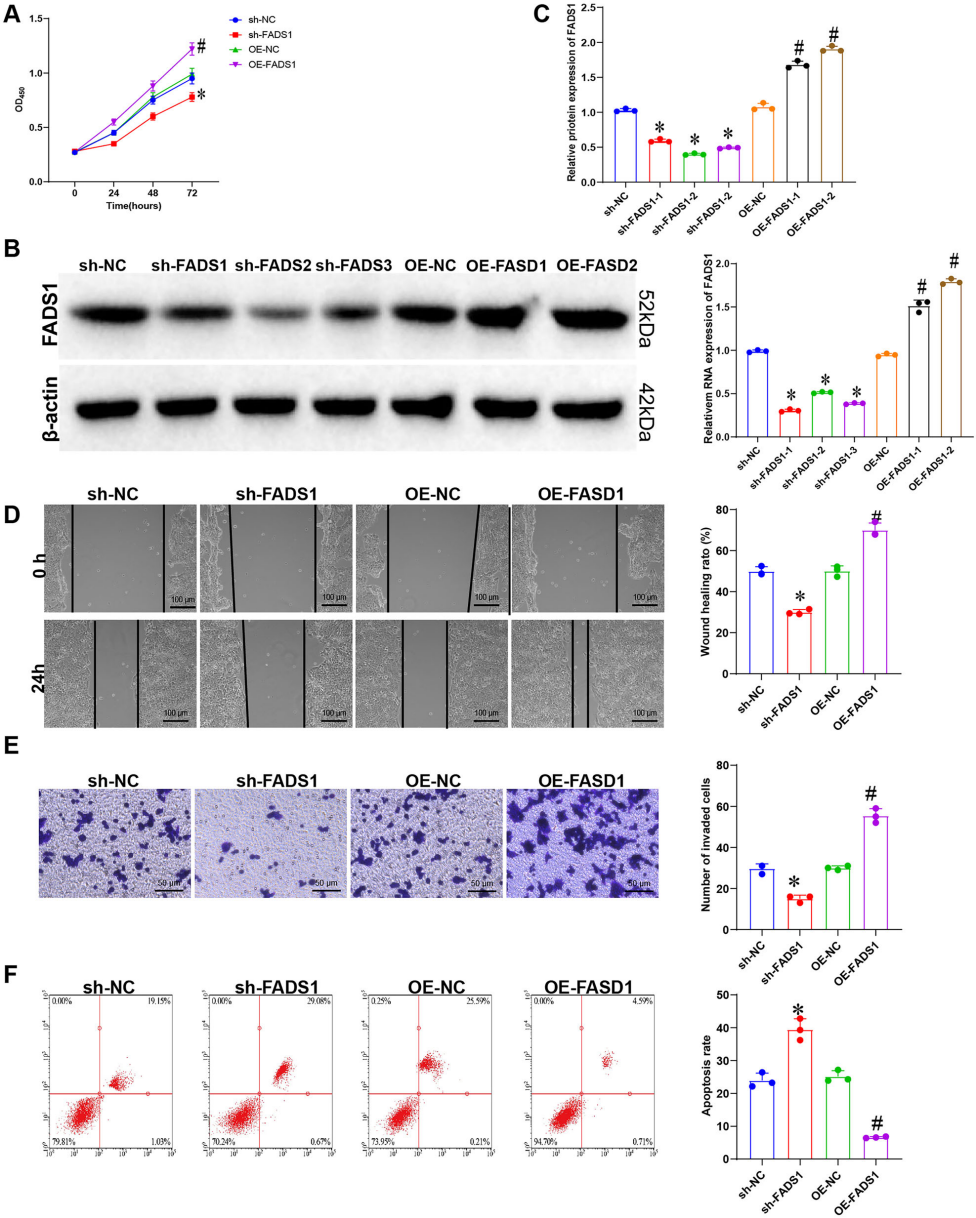
Effect of FADS1 on CC HCT116 cell function. **A** RT-qPCR verified the silencing and overexpression efficiency of FADS1; **B** WB verified the silencing and overexpression efficiency of FADS1; **C** Effect of FADS1 on cell proliferation; **D** Effect of FADS1 on cell migration; **E** Effect of cell invasion; **F** Effect of FADS1 on apoptosis of CC cells. The cell experiments were repeated three times, *indicating a comparison between the two groups, *p* < 0.05.

The roles of FADS1 in CC and EC were investigated through in vitro experiments. Stable FADS1-overexpressing and knockdown cell lines were generated, and gene expression changes were confirmed by RT-qPCR (Figs. 6A and S7A) and Western blot analysis (Figs. 6B and S7B). CCK-8 assays showed that FADS1 overexpression significantly enhanced cell proliferation, whereas its knockdown suppressed proliferation (Figs. 6C and S7C). Wound healing and Transwell assays demonstrated increased migration and invasion abilities in FADS1-overexpressing cells (Figs. 6D, E and S7D, E). Annexin V-FITC/PI staining revealed that FADS1 knockdown significantly increased apoptosis rates compared to controls (Figs. 6F and S7F).

### Establishment of FADS1 gene knockout mouse model and its impact on tumors

**Fig. 7:**
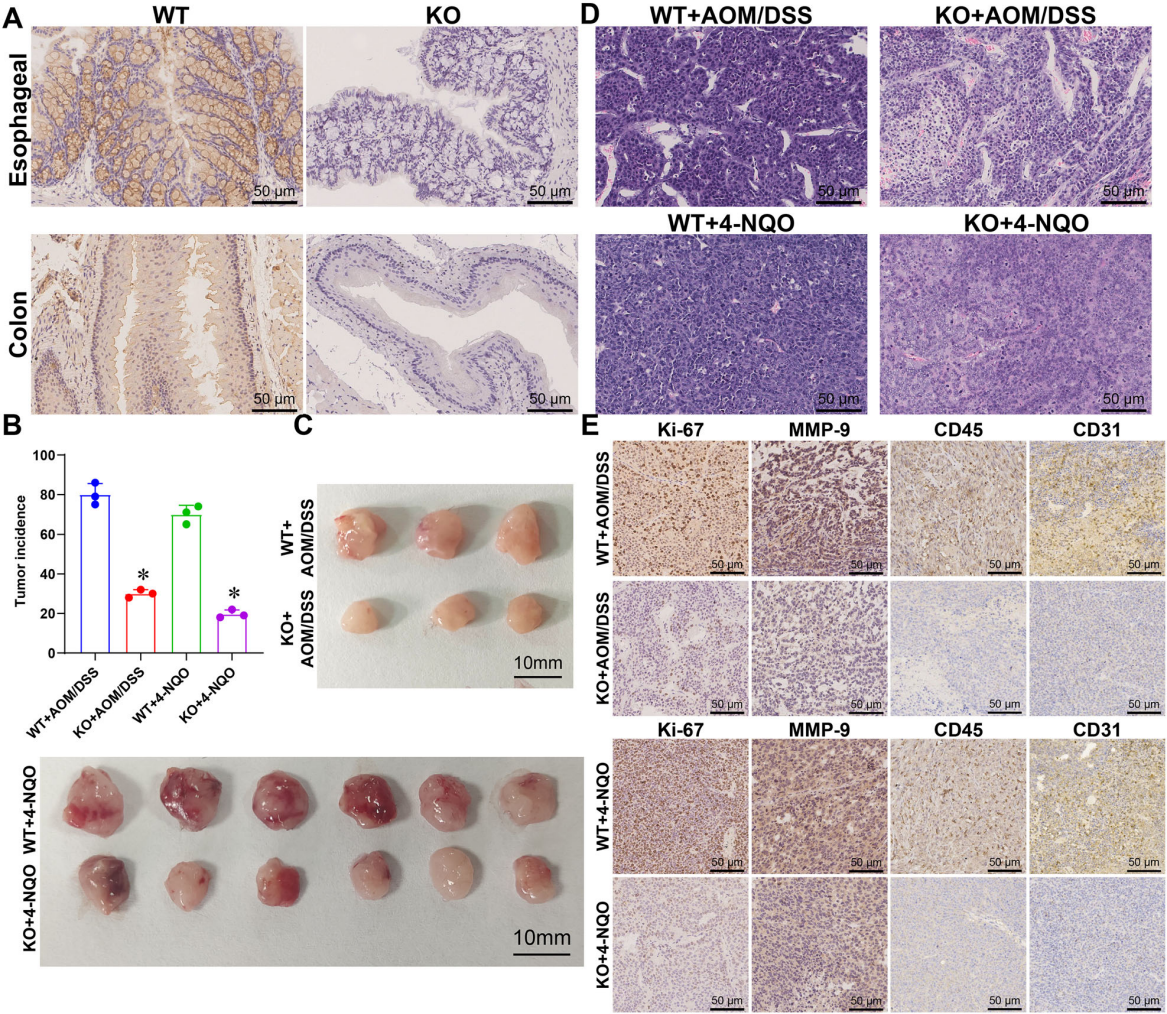
Establishment of a mouse model of FADS1 knockout and its effect on tumors. **A** Immunohistochemical staining to verify knockout of FADS1 gene; **B** Incidence of tumors in cancer model mice induced by chemical carcinogens; **C** Comparison of tumor volume between FADS1 knockout mice and control mice; **D** HE-stained cell density in tumor tissues of FADS1 knockout mice and control mice decreased; **E** Immunohistochemical staining of Ki-67, matrix metalloproteinase (MMP-9), CD45 immune tissue and CD31 in FADS1 knockout mice and control mice evaluated cell proliferation, cell invasion and inflammatory response.

To further investigate the role of FADS1 in CC and EC, we acquired the FADS1 gene knockout mouse model C57BL/6JCya-*Fads1*^*em1*^/Cya. Immunohistochemical staining confirmed near-complete loss of FADS1 protein expression in knockout tissues, validating the efficacy of the model (Fig. 7A). CC and EC models were established using AOM/DSS and 4-NQO, respectively. Within six months, tumor development was observed in wild-type mice, while tumor incidence and size were significantly reduced in FADS1 knockout mice (Fig. 7B). Pathological examination revealed that knockout mice exhibited attenuated tumor characteristics, including reduced cell proliferation and invasion (Fig. 7C–E). H&E staining showed lower nuclear-to-cytoplasmic ratios and decreased cell density (Fig. 7D). Immunohistochemical staining for Ki-67 and MMP-9 demonstrated diminished proliferative and invasive capacity (Fig. 7E), while CD45 and CD31 staining indicated reduced inflammatory cell infiltration and angiogenesis in the knockout group (Fig. 7E).

### Impact of FADS1 knockout on levels of lipid metabolism-related metabolites and cancer-related signaling pathways

**Fig. 8:**
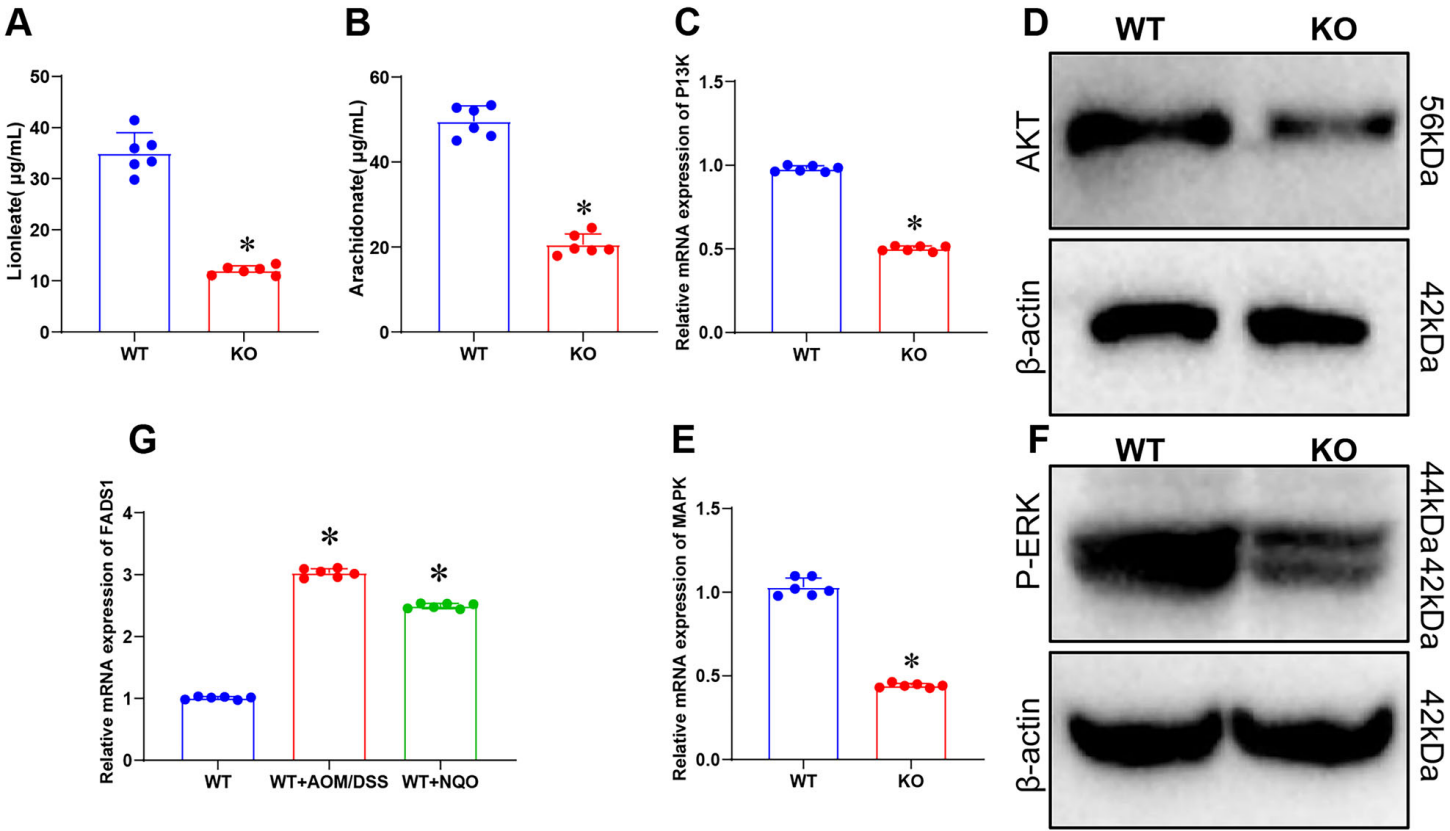
Effect of FADS1 gene knockout on lipid metabolites and cancer-related signaling pathways. **A** Effect of FADS1 knockout on linoleic acid levels; **B** Effect of FADS1 knockout on AA levels; **C**, **D** Effect of FADS1 knockout of PI3K/AKT signaling pathway; **C** Effect of FADS1 knockout on PI3K expression; **D** Effect of FADS1 knockout on phosphorylation levels of AKT protein; **E**, **F** Effect of FADS1 knockout on MAPK signaling pathway; **E** Effect of FADS1 knockout on MAPK expression; **F** Effect of FADS1 knockout on phosphorylation levels of ERK protein; **G** Effect of chemical carcinogens on FADS1 expression. The metric data in the Fig. are presented in Mean ± SD with 6 mice per group. *Indicates comparison between two groups, *p* < 0.05.

LC-MS analysis of mouse blood and tissue samples revealed that FADS1 knockout significantly altered metabolite profiles. Notably, lipid metabolism-related metabolites, including linoleic acid and AA, were markedly reduced in the knockout group (Fig. 8A, B), highlighting a regulatory role for FADS1 in lipid metabolism. Correlation analysis further confirmed strong associations between FADS1 and multiple metabolites (e.g., linoleic acid, AA), consistent with previous findings (Fig. 3 and Table S5). RT-qPCR and Western blot analyses showed that FADS1 knockout suppressed the activation of key cancer-related pathways, including PI3K/AKT and MAPK (Fig. 8C–F), which are known to regulate cell proliferation, migration, and survival. In mice exposed to chemical carcinogens, FADS1 expression was significantly upregulated (Fig. 8G), suggesting a response mechanism contributing to tumor development.

## Discussion

Causal relationships between metabolites and GI outcomes were evaluated using two-sample MR and LDSC. A subsequent three-step SMR analysis identified key genes associated with candidate causal metabolites. The findings indicate that distinct genetic determinants of metabolites contribute to varying risks across GI diseases, offering insight into the interplay among genetic variation, gene expression, methylation, and disease development.

FADS1 catalyzes the conversion of DGLA to arachidonic acid [22] and is associated with plasma levels of polyunsaturated fatty acids, HDL, LDL, and triglycerides [23]. Although previous studies linked FADS1 dysregulation to cancer progression in several malignancies [24, 25], its causal role in CC had not been established. This study demonstrates strong associations between FADS1 and fatty acid metabolism-related genes. High FADS1 expression, previously linked to high-fat diet exposure [26, 27], promotes proliferation, migration, and invasion of colorectal and EC cells while suppressing apoptosis, suggesting a metabolic role in tumor development. NPIPA5 was also identified as a novel mediator connecting metabolites and tumorigenesis [28].

The FADS1-AA axis may promote tumor development in CC by facilitating PGE2 synthesis and shaping the gut environment [29], highlighting FADS1 as a potential therapeutic target. This study also reveals a previously unreported causal relationship between FADS1 and EC, extending earlier findings that genetically predicted AA phospholipid levels are linked to risks of colorectal and lung cancers [30]. While the role of NPIPA5 remains poorly understood [28], its associations with metabolites and cancer emphasize the relevance of gene-metabolite interactions.

PBX2 has been reported to promote gastric cancer by enhancing infiltration [31], though its role in CC is unclear. Analysis in this study shows PBX2 is related to tumor stage, immune infiltration, and chemotherapy response [28], suggesting its potential as a therapeutic candidate. SMR analysis also identified disease-specific gene associations, including USP40 and FASN with fatty liver disease, TOR1A and TMC4 with inflammatory bowel disease, and CCL20 with gastritis (Figs. 2 and 3 and Tables S6 and S7).

This study provides the first experimental validation of FADS1 in colorectal and EC using both in vitro and mouse models. FADS1 knockout significantly reduces cell proliferation, migration, and invasion while increasing apoptosis [32, 33]. Unlike previous work focused on its metabolic role [32], this study links FADS1 directly to tumor progression. The mouse model findings further support the importance of FADS1 in cancer [33] and suggest translational potential.

FADS1 knockout disrupts lipid metabolism by reducing linoleic acid and arachidonic acid levels and also suppresses PI3K/AKT and MAPK signaling, which are key to proliferation, survival, and migration [34, 35]. This study reveals a dual mechanism by which FADS1 promotes cancer through metabolic regulation and pro-survival signaling. Although FADS1 is a promising therapeutic target, further studies are needed to dissect how it integrates metabolic and signaling pathways in cancer.

This study has several limitations. The relatively small sample size may affect the robustness and generalizability of the findings. Metabolite levels are susceptible to external influences such as diet and lifestyle, and potential confounders were not fully controlled. Although MR was applied to infer causality, experimental validation is still required. Additionally, the data were derived primarily from a specific population, which may limit applicability to other ethnic groups. Future studies should address these limitations by establishing orthotopic tumor models to evaluate the role of FADS1 in cancer metastasis, elucidating whether its regulation of PI3K/AKT and MAPK signaling involves arachidonic acid synthesis or transcriptional/post-transcriptional mechanisms, and incorporating clinical validation using human organoids or patient-derived primary tumor cells to enhance translational relevance.

## Conclusion

This study integrated MR and multi-omics analyses to uncover potential causal links between metabolites and common GI diseases, highlighting the key role of FADS1 in the development of CC and EC. Several metabolites were identified as significantly associated with CC, with genes such as FADS1 and NPIPA5 supported by SMR and colocalization evidence. Functional experiments confirmed that FADS1 promotes tumor cell proliferation, migration, and invasion, while its knockout suppresses tumor growth and inflammation in vivo, alters lipid metabolism, and inhibits PI3K/AKT and MAPK signaling. These findings provide mechanistic insights into GI cancer pathogenesis and support the utility of multi-omics approaches for identifying therapeutic targets and informing precision medicine strategies (Graphical Abstract).

## Materials and methods

### Research design

As outlined in Fig. S8, this study employed a two-sample MR framework [13–15] to identify metabolite-disease causalities using GWAS summary statistics (Table S1), followed by a three-step summary MR (SMR) approach integrating cis-eQTL/cis-mQTL data [18–20] to prioritize key regulatory genes. Sensitivity analyses (MR-Egger, IVW) confirmed robustness (Cochran’s *Q* *p* > 0.05), and colocalization analysis (PP.H4 > 0.75) validated pleiotropy-free associations [21], with multi-omics integration corroborating metabolic-pathway disruptions in GI cancers.

Detailed procedures, including reagent preparation and experimental protocols, are provided in Supplementary Materials 1. The overexpression vector for the FADS1 gene was constructed using the lentiviral vector pLenti-CMV-GFP-Puro (#17448, Addgene, USA), while the silencing vector was constructed using the lentiviral vector pLKO.1-puro (#8453, Addgene, USA). The relative gene expression levels were analyzed using the 2^−ΔΔCt^ method, normalized to the reference gene GAPDH, and all RT-qPCR detections were repeated three times (Table 2).

**Table 2:** RT-qPCR primer sequence.

Gene	Primer sequence (5′-3′)	Purpose
FADS1(Human)	Forword: 5′-GTTATCCAGCGAAAGAAGTGGG-3′	RT-qPCR for FADS1
	Reverse: 5′-CCAATAGTGGCACATAAGTGAGG-3′	RT-qPCR for FADS1
GAPDH (Human)	Forword: 5′-ACAACTTTGGTATCGTGGAAGG-3′	RT-qPCR Reference Gene
	Reverse: 5′-GCCATCACGCCACAGTTTC-3′	RT-qPCR Reference Gene
FADS1 (Mouse)	Forword: 5′-ACCCAGCTTTGAACCCACC-3′	RT-qPCR for FADS1
	Reverse: 5′-GAGGCCCATTCGCTCTACTG-3′	RT-qPCR for FADS1
GAPDH (Mouse)	Forword: 5′-CCCTTAAGAGGGATGCTGCC-3′	RT-qPCR Reference Gene
	Reverse: 5′-TACGGCCAAATCCGTTCACA-3′	RT-qPCR Reference Gene

*FADS1* Fatty acid desaturase 1, *GAPDH* Glyceraldehyde-3-phosphate dehydrogenase.

### Modeling of CC and EC in mice

FADS1 gene knockout (C57BL/6JCya-Fads1em1/Cya) and wild-type mice (C57BL/6JCya) aged 4–6 weeks were purchased from Shanghai Model Organisms Center. CC was induced via a single intraperitoneal injection of 10 mg/kg azoxymethane (AOM), followed by three 7-day cycles of 2.5% dextran sodium sulfate (DSS) in drinking water (total 16 weeks). EC was modeled by administering 100 μg/mL 4-nitroquinoline 1-oxide (4-NQO) in drinking water for 16 weeks. All animal experiments in this study were conducted strictly by the Guide for the Care and Use of Laboratory Animals to ensure animal welfare and experimental ethics. The study protocol was reviewed and approved by the Institutional Animal Care and Use Committee, with mice housed under SPF conditions (22–25 °C, 60–65% humidity, 12 h light/dark cycle) and euthanized by CO₂ at study completion.

### Statistical analysis

Statistical analysis involved three stages: two-sample MR to identify metabolite-disease causalities, summary-based SMR to prioritize key regulatory genes, and colocalization analysis to validate pleiotropy-free associations. Heterogeneity was assessed via Cochran’s *Q* and *I*² statistics, sensitivity analyses included MR-Egger and weighted median approaches, and F-statistics (>10) confirmed instrument validity. Multiple testing was controlled using Benjamini–Hochberg correction (FDR < 0.05), with nominal significance defined as *p* < 0.05 and adjusted *p* < 0.2.

## Supplementary information

Supplementary figures

Supplementary Materials 1

Supplementary Tables

Full and uncropped western blots

## Data Availability

All data can be provided as needed.
