# Supplementary material for: Targeting FADS1-mediated lipid metabolism and signaling: a novel therapeutic strategy for precision oncology in colorectal and esophageal cancers
Source: Cell Death Discov. 2025 Oct 16;11:460. doi: 10.1038/s41420-025-02768-3 (PMC12533223; doi:10.1038/s41420-025-02768-3)
Supplement: Supplementary file 1 — Supplementary figures [file 41420_2025_2768_MOESM1_ESM.docx]

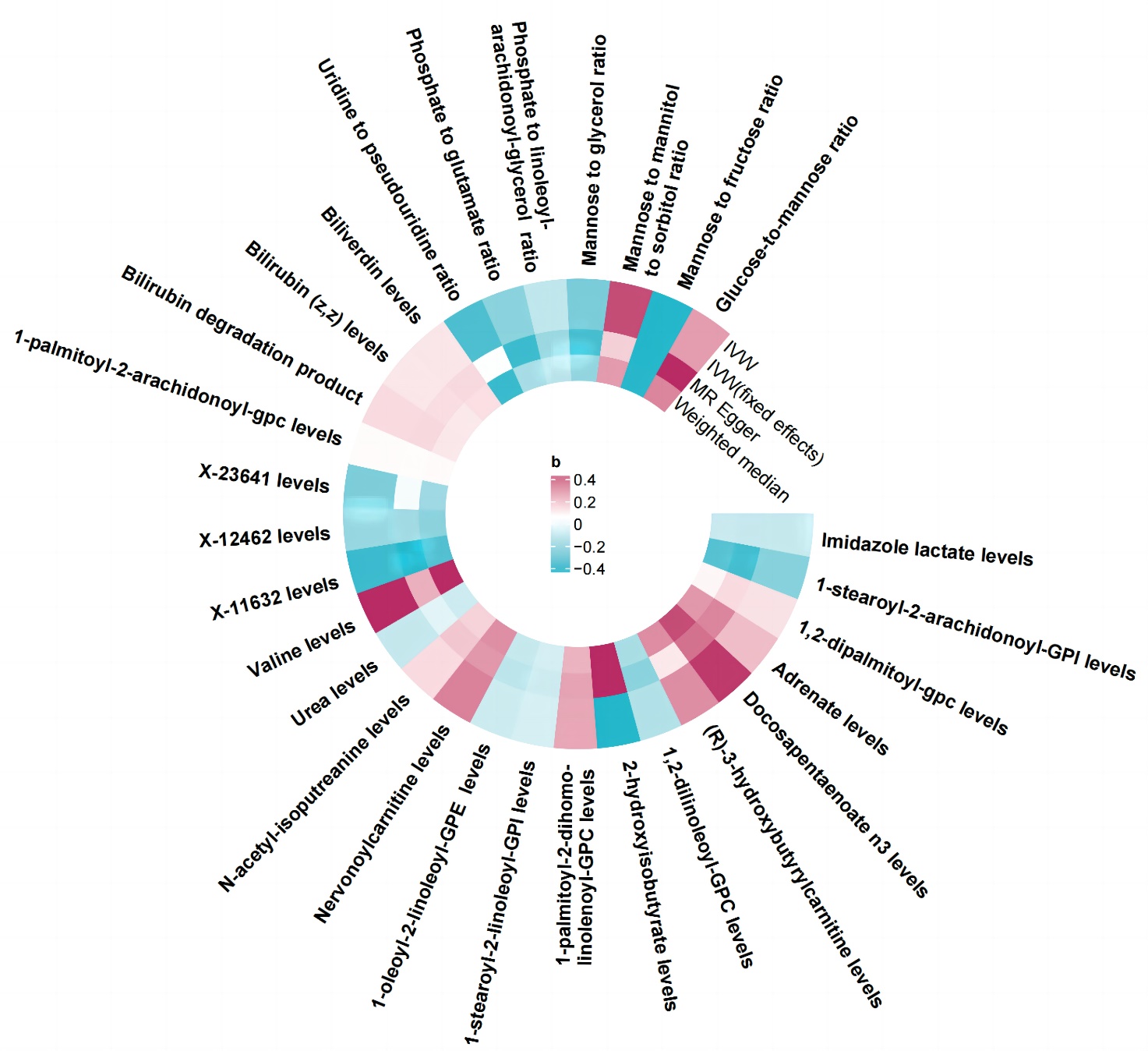


**Fig. S1: Consistency Verification of Sensitivity Analysis Results.**

Note: The Fig. illustrates the consistency of beta values obtained from various Mendelian Randomization (MR) analysis methods, including Inverse Variance Weighting (IVW), MR Egger, and Weighted Median. The results demonstrate robustness across different methods, supporting the causal relationship between metabolites and related diseases.


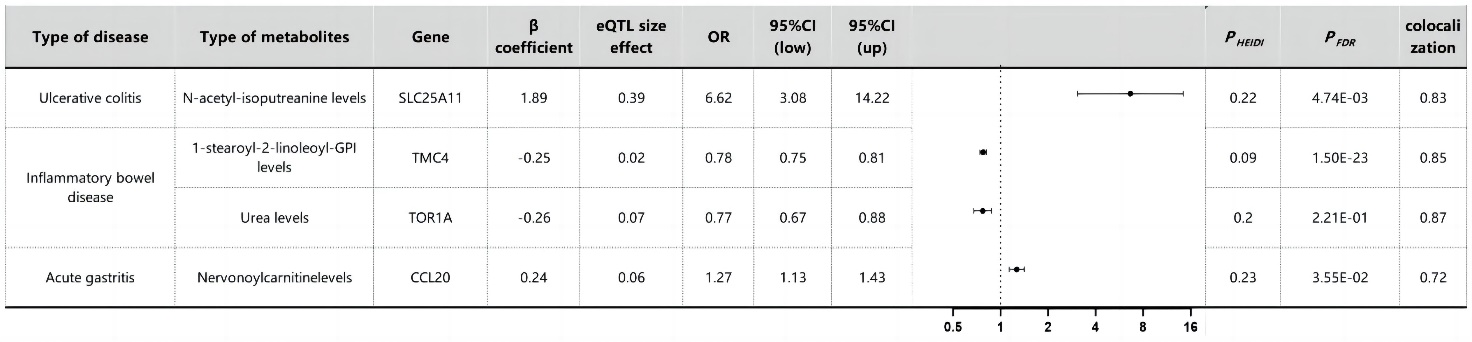


**Fig. S2: Causal Relationship Between Metabolites and Gastrointestinal Diseases Identified Through SMR Analysis.**

Note: The table presents metabolites associated with ulcerative colitis, inflammatory bowel disease, and acute gastritis, alongside their corresponding gene eQTL effect sizes, β coefficients, odds ratios (OR), and 95% confidence intervals. These associations were further validated for robustness through multiple testing correction and HEIDI testing, highlighting the potential roles of these metabolites in various gastrointestinal diseases.


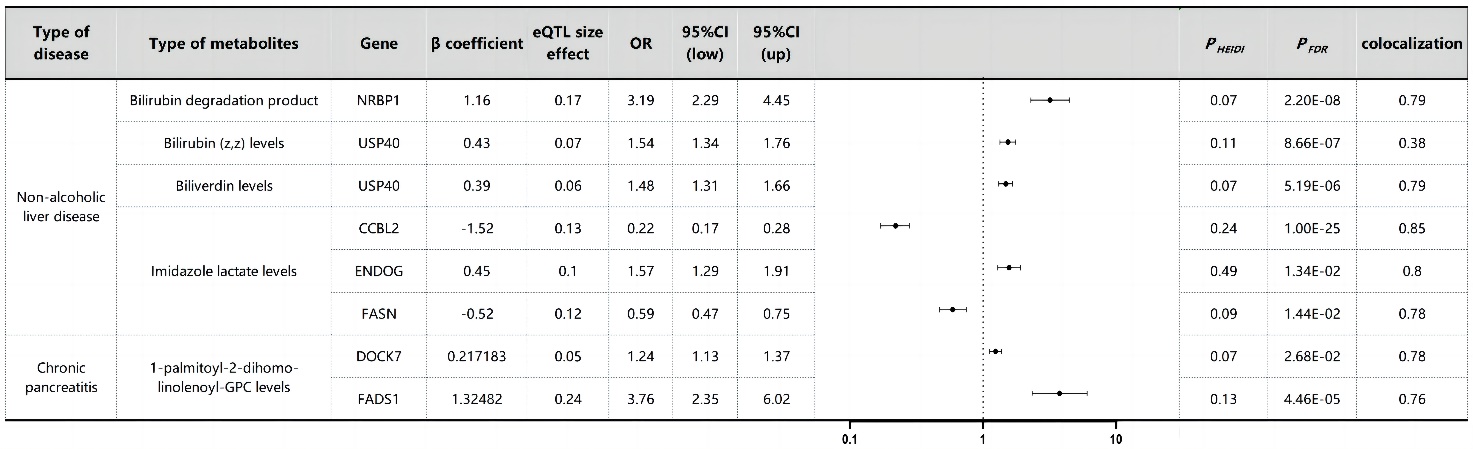


**Fig. S3: Causal Associations Between Genes and Metabolites in Non-Alcoholic Fatty Liver Disease and Chronic Pancreatitis Revealed by SMR Analysis.**

Note: The table displays metabolites associated with these diseases, their corresponding gene eQTL effect sizes, β coefficients, ORs, and 95% confidence intervals. Multiple testing correction and HEIDI testing were applied to confirm the robustness of these associations, revealing the potential roles of these metabolites in non-alcoholic fatty liver disease and chronic pancreatitis.


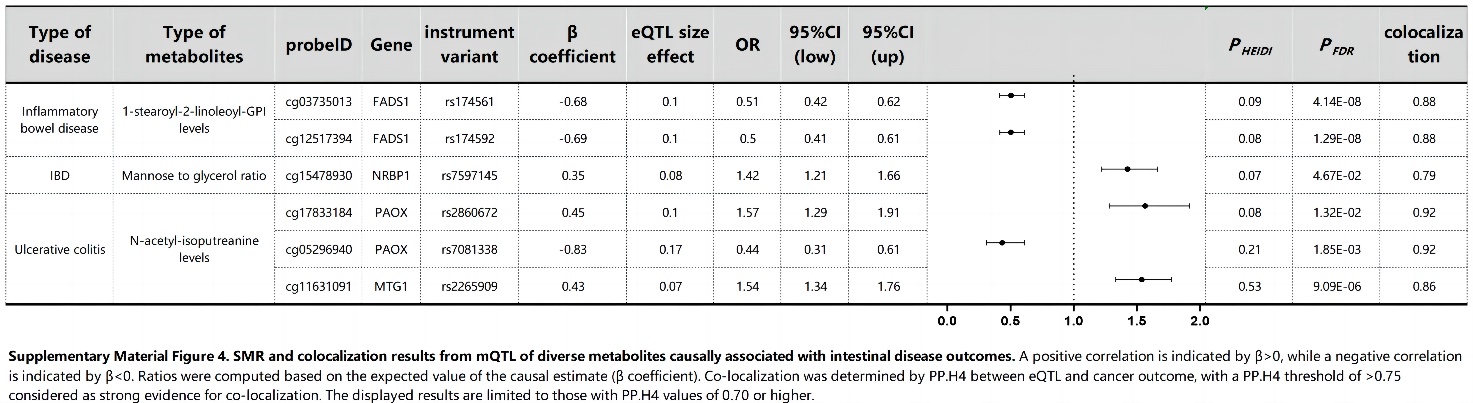


**Fig. S4: Causal Associations Between Multiple Metabolites and Gastrointestinal Diseases as Evidenced by SMR and Colocalization Analysis.**

Note: The Fig. shows association signals between various metabolites, based on mQTL, and inflammatory bowel disease or ulcerative colitis. Robustness of these associations was confirmed through multiple testing correction (Benjamini–Hochberg adjustment, PFDR < 0.05) and HEIDI testing (PHEIDI > 0.05), further supporting the role of these metabolites in gastrointestinal diseases. Colocalization analysis provided strong evidence of colocalization between eQTLs and cancer outcomes, with PP.H4 values exceeding 0.75 considered strong evidence.


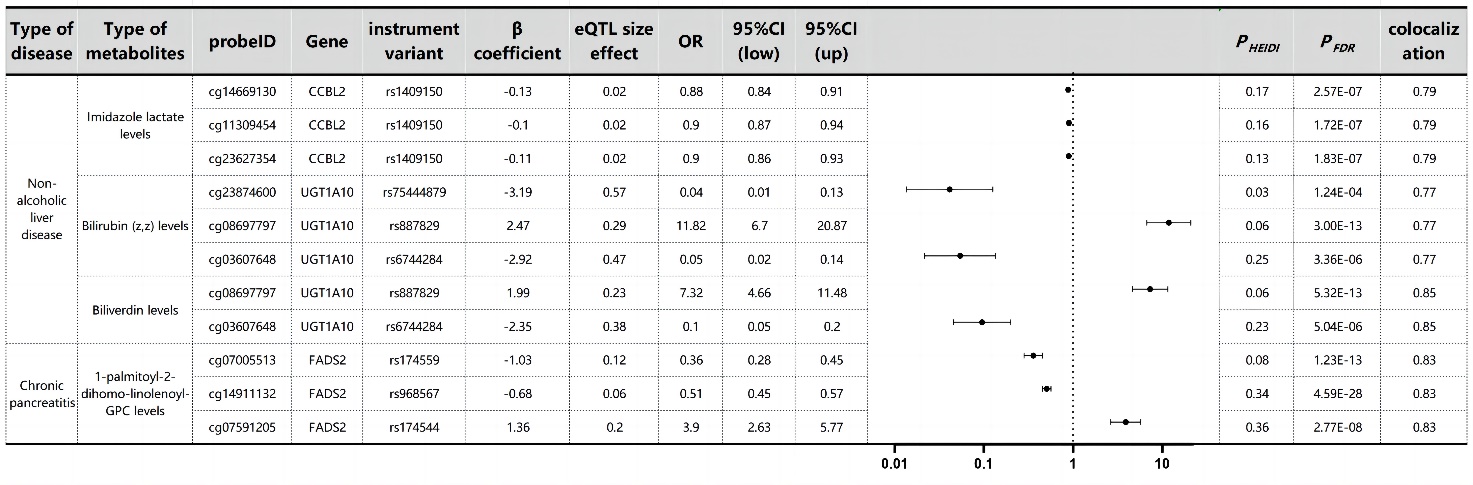


**Fig. S5: SMR and Colocalization Analysis Reveals Causal Associations Between Various Metabolites and Non-Alcoholic Fatty Liver Disease and Chronic Pancreatitis.**

Note: The table illustrates the impact of gene variants on metabolite DNA methylation levels and their influence on disease risk. The robustness of these associations was confirmed through multiple testing correction and HEIDI testing, with particular emphasis on the specific loci and genes involved in non-alcoholic fatty liver disease and chronic pancreatitis.


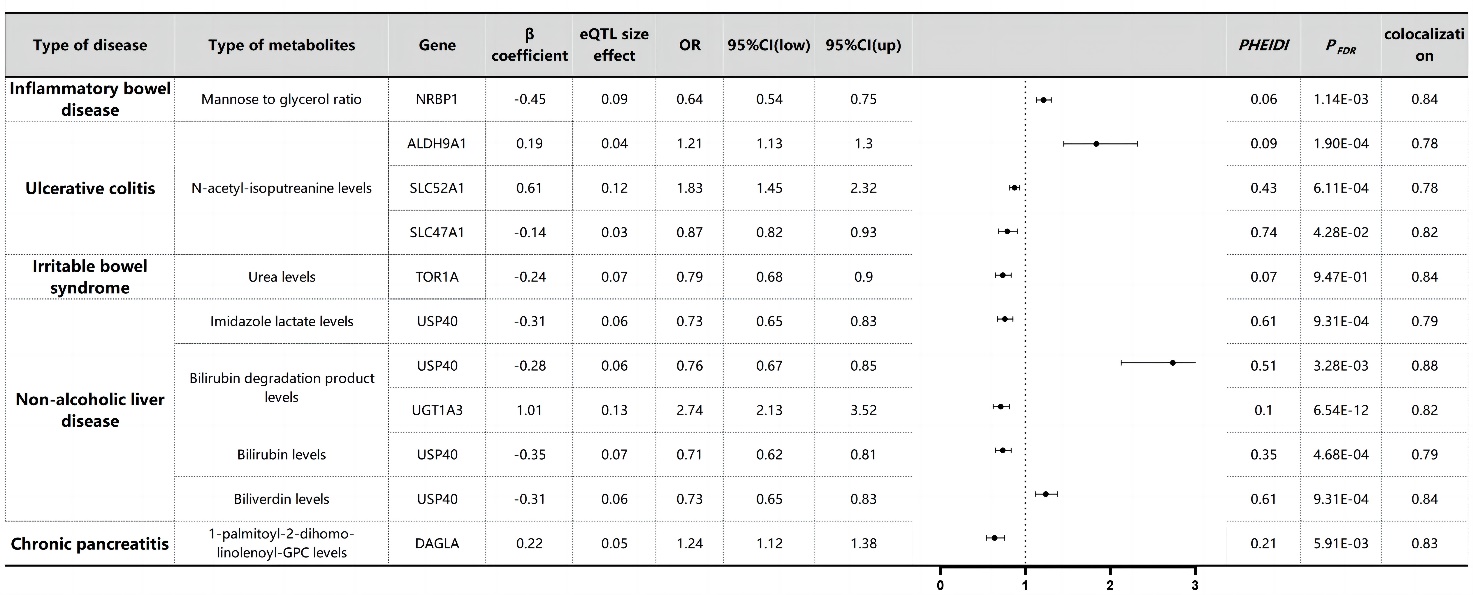


**Fig. S6: Causal Associations Between Various Metabolites and Gastrointestinal Diseases, Non-Alcoholic Fatty Liver Disease, and Chronic Pancreatitis.**

Note: The table presents gene variants associated with these diseases, metabolite levels, eQTL effects, β coefficients, and 95% confidence intervals. The robustness of these associations was further validated through PHEIDI testing and multiple testing correction, revealing potential biological mechanisms involved in these diseases.


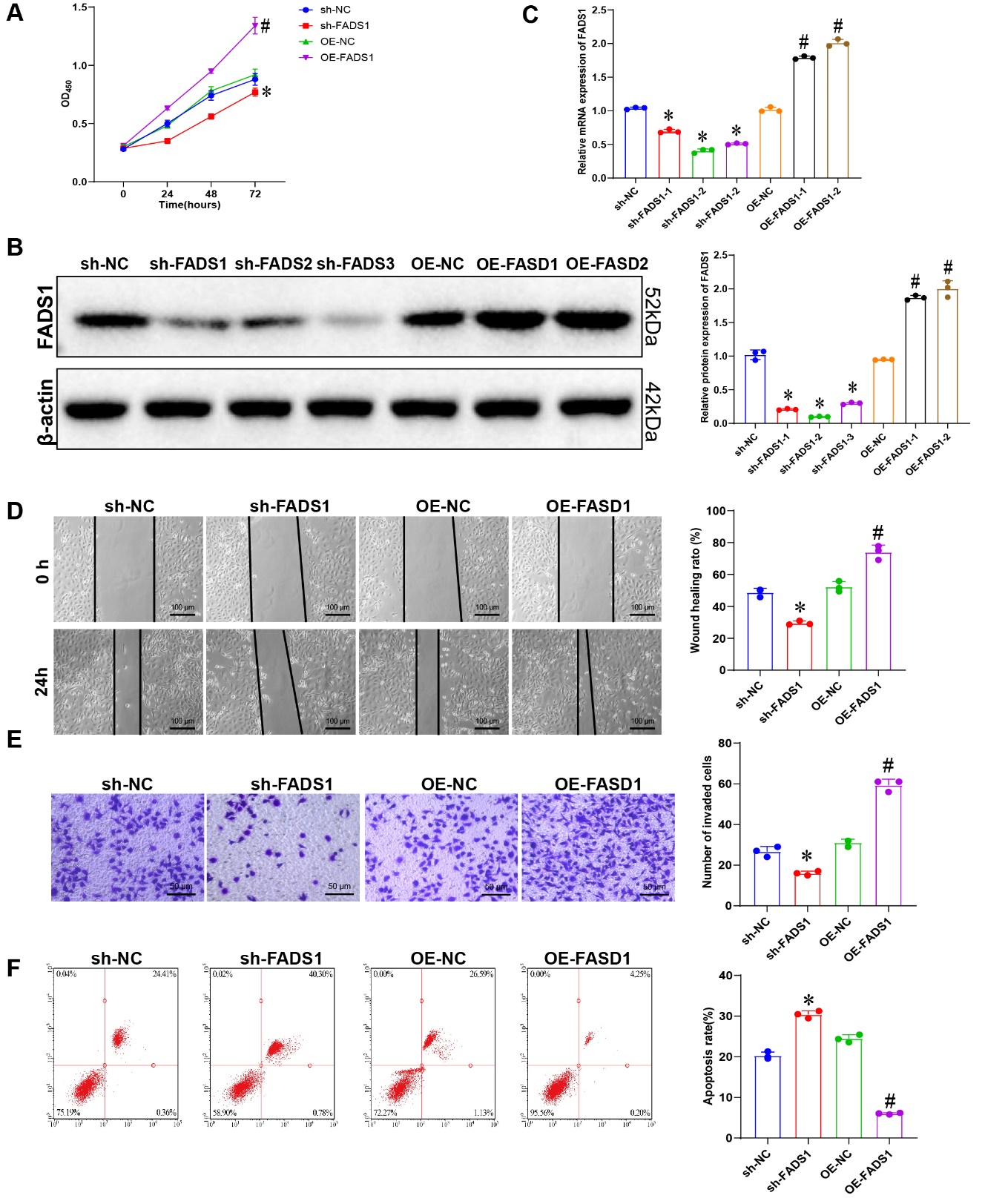


**Fig. S7. Effect of FADS1 on EC CW1474 cell function.**

Note: (A) RT-qPCR verified the silencing and overexpression efficiency of FADS1; (B) WB verified the silencing and overexpression efficiency of FADS1; (C) Effect of FADS1 on cell proliferation; (D) Effect of FADS1 on cell migration; (E) Effect of cell invasion; (F) Effect of FADS1 on apoptosis of EC cells. The cell experiments were repeated three times, *indicating a comparison between the two groups, *p*<0.05.


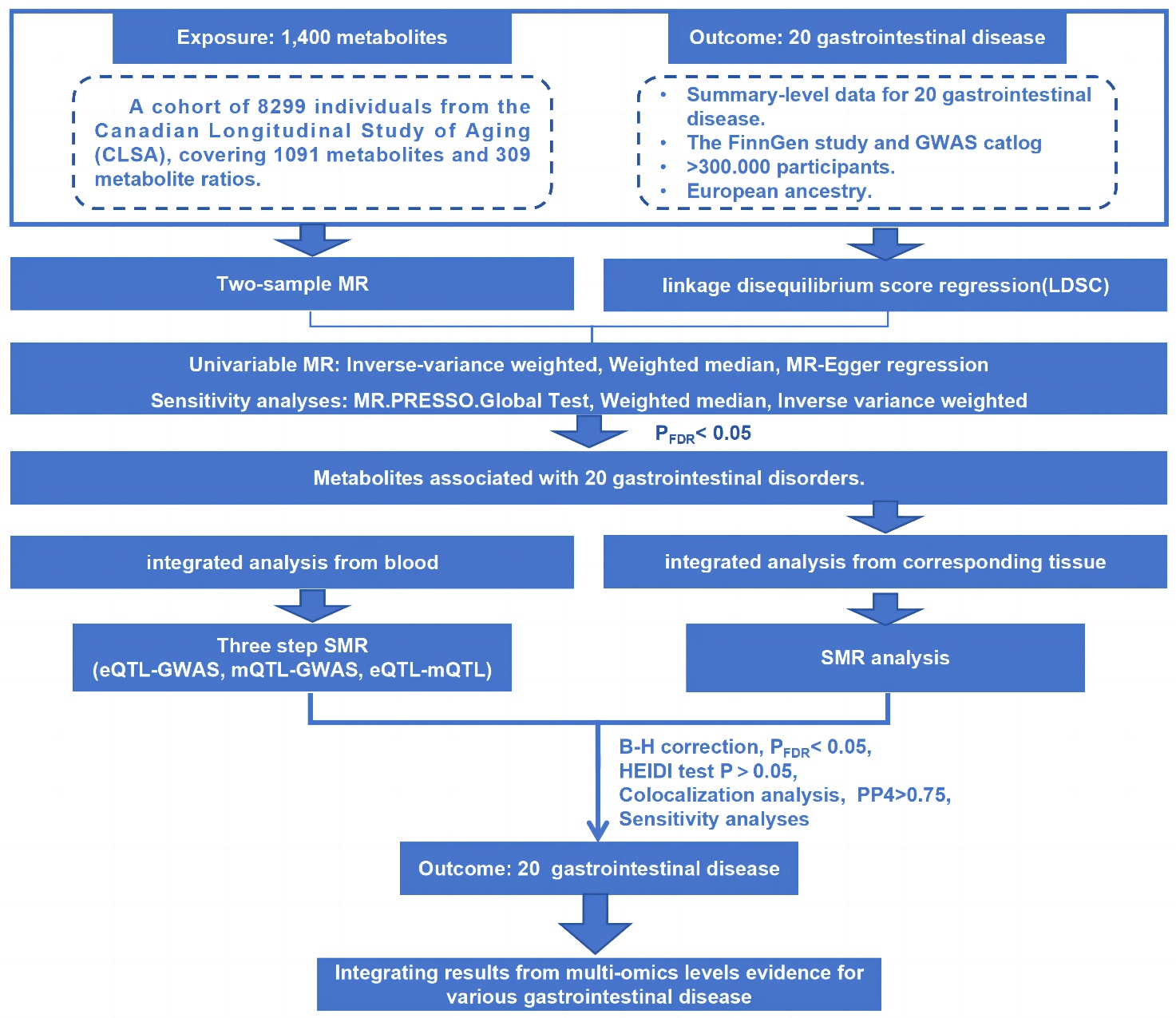


**Fig. S8. Flowchart of the analysis performed.**
