# Supplementary material for: Targeting FADS1-mediated lipid metabolism and signaling: a novel therapeutic strategy for precision oncology in colorectal and esophageal cancers
Source: Cell Death Discov. 2025 Oct 16;11:460. doi: 10.1038/s41420-025-02768-3 (PMC12533223; doi:10.1038/s41420-025-02768-3)
Supplement: Supplementary file 2 — Supplementary Materials 1 [file 41420_2025_2768_MOESM2_ESM.docx]

**Summary data-based Mendelian randomization (SMR)**

SMR was performed using SMR software (v1.0.3, Windows) with default settings to assess associations between genetically predicted molecular traits (e.g., gene expression, DNA methylation, protein abundance) and complex traits, including disease phenotypes. Genetic instruments were derived from expression quantitative trait loci (eQTL) data, and GWAS summary statistics were used to prioritize functional genes. The heterogeneity in dependent instruments (HEIDI) test, based on European reference data from the 1000 Genomes Project, was used to exclude associations potentially confounded by linkage disequilibrium (LD) (*p* < 0.05). To account for horizontal pleiotropy, the Mendelian Randomization Pleiotropy RESidual Sum and Outlier (MR-PRESSO) method was applied using the MRPRESSO R package. This included global heterogeneity testing and correction for outlier instruments. Most causal estimates remained stable after correction, indicating robust findings. Full results are shown in Table S3.

**Co-Localization Analysis**

Co-localization analysis was conducted to assess whether GWAS and QTL signals shared a common causal variant within the same genomic region. Bayesian co-localization testing was performed using the coloc R package (v5.1.0), which estimates posterior probabilities for five hypotheses: H0, no causal variant in either trait; H1, causal variant in trait 1 only; H2, causal variant in trait 2 only; H3, distinct causal variants in both traits; and H4, a shared causal variant. For each lead SNP identified in the GWAS of gastrointestinal traits, all variants within ±100 kb were included in the co-localization analysis. A posterior probability for H4 (PP.H4) greater than 0.75 was considered strong evidence of co-localization between GWAS and QTL signals.

**Linkage disequilibrium Score Regression (LDSC)**

Linkage disequilibrium score regression (LDSC) was used to evaluate genetic correlations between metabolites and gastrointestinal diseases based on GWAS summary statistics. Analyses were conducted using the LDSC software, with LD scores precomputed from HapMap3 single-nucleotide polymorphisms (SNPs) in individuals of European ancestry from the 1000 Genomes Project. Only autosomal SNPs with minor allele frequency (MAF) > 0.01 were included. A *p*-value threshold of < 0.05 was considered suggestive of a significant genetic correlation. The LDSC intercept was left unconstrained to account for potential residual confounding and sample overlap between GWAS datasets.

**Integrating Multisystemic Evidence-Level Findings**

This study integrated results from three types of QTL datasets to investigate metabolite-related gene regulation in gastrointestinal (GI) diseases. Candidate genes were classified by strength of evidence: level 1 evidence was assigned to genes showing significant associations across all three QTL datasets, while level 2 evidence was defined by significance in at least two datasets. To further refine these associations, colocalization analysis was performed, retaining only those with a posterior probability (PP.H4) greater than 0.75, thereby minimizing potential confounding due to linkage disequilibrium.

**Expression Analysis of FADS1 in** **colon cancer (CC) and esophageal cancer (EC)**

To evaluate FADS1 expression in CC and EC and its association with clinical stage, transcriptomic data were obtained from The Cancer Genome Atlas (TCGA), including 275 CC and 41 normal colon tissues, as well as 182 EC and 13 normal esophagus tissues. Gene expression was normalized to transcripts per million (TPM) and log2-transformed as log_2_(TPM + 1). Differential expression between tumor and normal tissues was assessed using the Wilcoxon rank-sum test, and one-way ANOVA was used to evaluate differences across clinical stages (I–IV). Analyses and visualizations were conducted in R. Box plots illustrated expression differences between cancerous (red) and normal (gray) tissues, with significance denoted by red asterisks. Violin plots showed stage-specific expression distributions, with white dots for medians and black bars for interquartile ranges. Statistical significance was defined as *p* < 0.05.

**Cell Culturing and Handling**

The cells used in the experiments were purchased from Sinozhongyuan. The CC cell line HCT116 and the EC cell line CW1474 (catalog numbers CCL-247EMT and CRL-3529, Sinozhongyuan) were cultured in a Heracell™ Vios 160i CO_2_ incubator (Thermo Scientific™, Germany) at 37°C with 5% CO₂. The culture medium for each cell line was as follows:

For HCT116 cells: RPMI-1640 medium (ATCC, USA) supplemented with 10% fetal bovine serum (ATCC, USA, #30-2000) and 1% penicillin-streptomycin (100 U/mL penicillin, 100 μg/mL streptomycin, ATCC, USA, #30-2001).

For CW1474 cells, the medium was RPMI-1640 (ATCC, USA, #30-2001) supplemented with 10% fetal bovine serum (ATCC, USA, #30-2000) and 1% penicillin-streptomycin (100 U/mL penicillin, 100 μg/mL streptomycin, ATCC, USA).

The medium was changed every 2-3 days during the culturing process to ensure healthy cell growth. When the cells reached 70-80% confluency, they were detached using trypsin, collected by centrifugation, and resuspended in a fresh medium. Subculture was performed at a ratio of 1:3 or 1:4 depending on the cell growth to maintain cell health and prevent contamination.

Regular monthly checks for mycoplasma contamination were conducted during the cell culture process. Before and after subculturing, the cells were tested for mycoplasma contamination. The cell culture supernatants were collected, and Hoechst 33342 dye solution (Sigma-Aldrich, USA) was added at a final concentration of 1 μg/ml. The mixture was then incubated at room temperature for 15-30 minutes. Bright blue fluorescence would indicate the presence of mycoplasma DNA when observed under a fluorescent microscope.

**Lentiviral Infection and Construction of Stable Transfected Cell Lines**

The FADS1 overexpression construct was generated using the pLenti-CMV-GFP-Puro vector (#17448, Addgene, USA), and the silencing construct was generated using the pLKO.1-puro vector (#8453, Addgene, USA). Detailed sequence information is provided in Supplementary Materials 2. Lentiviral particles were packaged in HEK293T cells (#CRL-11268, ATCC, USA) cultured in 6-well plates at 70-80% confluency. The lentiviral vector, psPAX2 packaging plasmid (#12260), and pMD2.G envelope plasmid (#12259) were co-transfected at a 4:3:1 ratio using Lipofectamine 3000 (Thermo Fisher, USA). After 6-8 hours, the medium was replaced, and viral supernatants were harvested 72 hours post-transfection, clarified by centrifugation, filtered (0.45 μm), and concentrated by ultracentrifugation at 50,000g for 2 hours. Viral pellets were resuspended in PBS and used to infect HCT116 and CW1474 cells at 60% confluency in the presence of Polybrene (8 μg/mL, HY-112735, MCE, China). After 24 hours, the medium was changed, and stable cell lines were selected using puromycin (E607054, Selleck, China). FADS1 overexpression and knockdown were confirmed by RT-qPCR.

Based on experimental requirements, both HCT116 and CW1474 cells were categorized into the following groups: sh-NC (silencing control group), sh-FADS1 (FADS1 silencing group), sg-NC (overexpression control group), and sg-FADS1 (FADS1 overexpression group).

**RT-qPCR Detection**

Total RNA was extracted using TRIzol reagent (15596026CN, Thermo Fisher, USA) following the manufacturer’s protocol. Briefly, cells were lysed in 1 mL TRIzol and incubated at room temperature for 5 minutes, followed by the addition of 200 µL chloroform. After vigorous mixing and phase separation by centrifugation (12,000g, 15 min, 4°C), the aqueous phase was collected, mixed with an equal volume of isopropanol, and incubated for RNA precipitation. The RNA pellet was washed with 75% ethanol, air-dried, and dissolved in DEPC-treated water. RNA concentration and purity were measured spectrophotometrically. Genomic DNA was removed, and complementary DNA (cDNA) was synthesized using the PrimeScript RT reagent Kit with gDNA Eraser (RR047Q, TaKaRa, Japan). Reverse transcription was performed at 37°C for 15 minutes, followed by inactivation at 85°C for 5 seconds. Quantitative PCR (qPCR) was conducted using AceQ qPCR SYBR Green Master Mix (Q131-02, Vazyme, China) according to the manufacturer’s instructions. The thermal cycling conditions were: 95°C for 5 minutes, followed by 40 cycles of 95°C for 10 seconds and 60°C for 30 seconds. Relative mRNA expression levels were calculated using the 2^-ΔΔCt^ method, normalized to GAPDH. All qPCR reactions were performed in triplicate. Primer sequences are listed in Table 2.

**Western Blot (WB) Detection**

Total protein was extracted from transfected cells, washed twice with cold PBS at 4°C, and then lysed with RIPA lysis buffer containing protease inhibitors (P0013B, Beyotime Biotechnology, Shanghai) on ice for 30 minutes. The lysate was centrifuged at 12000g for 15 minutes at 4°C, and the supernatant was collected for protein concentration determination using the BCA method. The proteins were mixed with 2× SDS-PAG loading buffer at a 1:1 ratio, denatured in a boiling water bath for 10 minutes, and stored at -80°C. A 10% SDS-PAGE gel was prepared, and the protein samples were electrophoresed in the gel. Subsequently, the proteins were transferred from the gel to a PVDF membrane (IPVH85R, Millipore, Darmstadt, Germany). Following the transfer, the membrane was blocked with 5% skim milk solution prepared in 1× TBST at room temperature for 1 hour. The membrane was then incubated overnight at 4°C with a diluted FADS1 specific antibody. After washing the membrane three times for 10 minutes each with 1× TBST solution, it was incubated at room temperature for 1 hour with an HRP-conjugated goat anti-rabbit IgG (ab6721, Abcam, UK, dilution: 1:5000) or goat anti-mouse IgG (ab205719, Abcam, UK, dilution: 1:5000) secondary antibody. Finally, the protein signal was detected using an ECL detection kit (1705062, Bio-Rad, USA), followed by exposure and recording of results. The Quantity One V4.6.2 software from BIO-RAD was used for analysis, with β-actin as the internal control, calculating the ratio of the target band's intensity to the reference band's intensity as the relative protein expression level. The experiment was repeated three times, and the mean ± SD was used for statistical analysis.

**CCK-8 Detection**

The cell suspension was seeded into a 96-well plate at a density of 5000 cells/well, with 5 replicate wells per experimental group. After the cells adhered for 24 hours, cell proliferation was evaluated using the CCK-8 assay kit (C0041, Beyotime, Shanghai, China). 10 µL of CCK-8 reagent was added to each well, the plate was gently shaken and then incubated at 37°C for 1 hour in a cell culture incubator. The absorbance values of each well at OD450 were measured using a microplate reader, and this process was repeated for 3 consecutive days with daily recordings. The average OD values at each time point were calculated, and a cell proliferation curve was plotted to compare the cell proliferation capabilities of different cell lines.

**Scratch Assay**

Cells were seeded in 12-well plates at a density of approximately 100,000 cells per well and allowed to grow to confluence (90-100%). Using a 200 µL pipette tip, a straight line scratch was made on the cell monolayer, followed by a gentle wash with PBS to remove floating cells, then replenished with serum-free culture medium. The initial image of the scratch at 0 hours was captured, and the plate was incubated in a 5% CO_2_, 37°C incubator. Images of the scratch area were taken every 6 hours until the scratch was fully healed. The Image-Pro Plus 6.0 software was used to analyze the scratch closure area and calculate the migration rate.

**Transwell Chamber Assay**

A layer of Matrigel matrix gel was evenly coated on the membrane of the Transwell chamber and left to gel at 37°C for 1 hour. Cells suspended in serum-free culture medium were added to the upper chamber of the Transwell chamber, with a cell suspension concentration of 2.5×10^5^ cells/mL in 200 μL per chamber. The lower chamber was filled with 600 μL of medium containing 10% FBS as a chemoattractant. The Transwell chambers were placed in a 24-well plate and incubated at 37°C for 24 hours. After incubation, the cells that did not migrate through the upper chamber were removed by wiping with a cotton swab. The cells that passed through the membrane were fixed with methanol and stained with 0.1% crystal violet. Images of the cells that crossed the membrane were captured under a microscope, and the number of cells that infiltrated through the membrane was counted to assess their invasion capability.

**Flow Cytometry Analysis**

The treated cells were collected in 1.5 mL EP tubes, centrifuged to remove the supernatant, and washed twice with PBS. Cell apoptosis was evaluated using the Annexin V-FITC/PI assay kit (C1062L, Beyotime, Shanghai, China). The cells were resuspended in 100 μL of 1x Annexin V binding buffer and incubated in the dark for 10-15 minutes after adding 5 μL of Annexin V-FITC and 5 μL of PI. The mixture was diluted with 400 μL of 1x Annexin V binding buffer. Cell apoptosis was analyzed using a flow cytometer to detect the fluorescent signals of Annexin V-FITC (green) and PI (red), assessing the proportion of apoptotic and necrotic cells.

**Tissue Sample Processing and Analysis**

Blood, esophagus, intestine, and other tissue samples of mice were collected, washed with PBS, fixed in 4% paraformaldehyde, embedded in paraffin, and sectioned. Hematoxylin and eosin (HE) staining, Ki-67, MMP-9, CD45, and CD31 immunohistochemical staining were performed to evaluate cell proliferation, invasive capacity, inflammation response, and angiogenesis.

**HE Staining**

For HE staining, fixed tissue samples were deparaffinized in xylene, dehydrated in graded alcohols, stained with hematoxylin for 5 minutes, rinsed with tap water, differentiated in hydrochloric acid-ethanol solution, counterstained with eosin for 2 minutes, rinsed with tap water, dehydrated in graded alcohols, cleared in xylene, and mounted with neutral mounting medium. Tissue morphology changes were observed under a microscope.

**Immunohistochemical Staining**

Tissue sections were deparaffinized in xylene and dehydrated in graded alcohols, followed by incubation in 3% hydrogen peroxide at room temperature for 10 minutes to block endogenous peroxidase activity. Antigen retrieval was performed using sodium citrate buffer (pH 6.0) and heated in a pressure cooker for 3 minutes. After cooling to room temperature, sections were washed thrice with PBS for 5 minutes each. Sections were then incubated with blocking solution (5% bovine serum albumin) at room temperature for 30 minutes, followed by incubation with primary antibodies against Ki-67 (1:200, Abcam, UK), MMP-9 (1:200, Abcam, UK), CD45 (1:200, Abcam, UK), and CD31 (1:200, Abcam, UK) overnight at 4°C. After washing with PBS thrice for 5 minutes each, sections were incubated with secondary antibody (HRP-conjugated goat anti-rabbit IgG, 1:500, Abcam, UK) at room temperature for 1 hour. Following another round of washing with PBS, DAB chromogen (Beyotime, Shanghai, China) was used for visualization. The staining intensity was observed under a microscope, and the reaction was stopped, followed by counterstaining with hematoxylin, dehydration, clearing, and mounting with neutral mounting medium.

**Metabolite Level Detection**

Sample collection and metabolite extraction: collect the cell culture medium for detecting metabolites secreted into the medium. Replace the serum-containing medium with serum-free medium and incubate for 6 hours to reduce serum interference. After collecting the supernatant, centrifuge to remove cell debris, and store the supernatant at -80°C. Collect the cell lysate for detecting intracellular metabolites. Wash the cells twice with PBS, scrape the cells with 4°C PBS, transfer the cell suspension to a 1.5 mL EP tube, centrifuge and discard the supernatant, add cold methanol/water (80:20, v/v) mixture to precipitate proteins and extract metabolites. Vortex to mix well, incubate at -20°C for 30 minutes, then centrifuge again, and store the supernatant at -80°C. Treat the samples with cold methanol to precipitate sample proteins. Mix the samples with cold methanol at a 1:3 ratio, vortex for 1 minute, incubate at -20°C for 30 minutes to ensure complete precipitation, then centrifuge and collect the supernatant for metabolite analysis. For low-concentration metabolites, use a vacuum centrifuge to concentrate the supernatant, then resuspend in an appropriate amount of solvent. Additionally, collect mouse blood samples and centrifuge the whole blood at 3000g for 10 minutes at 4°C. Collect the supernatant plasma, transfer it to a sterile tube, and immediately freeze it at -80°C. Plasma treatment: Take 50-100μL of plasma, add 3 times the volume of ice-cold methanol, vortex for 1 minute, incubate at 4°C for 30 minutes, and centrifuge at 14000g for 10 minutes. Collect the supernatant and transfer it to a new sterile tube. Collect EC and GI tissue samples, wash away the blood with PBS, cut the tissue samples into small pieces (about 50-100mg), place them in pre-cooled EP tubes, and immediately freeze in liquid nitrogen. Tissue treatment: Place the frozen tissue samples in a homogenizer, add an appropriate amount of cold methanol/water (80:20, v/v) mixture (tissue to solvent ratio is 1:10, w/v). Process the samples using an ultrasonic disruptor 3-5 times, each time for 5 seconds, with a 10-second interval. Centrifuge at 14000g for 10 minutes, collect the supernatant, and transfer it to a new sterile tube.

Liquid chromatography-mass spectrometry (LC-MS) analysis: Prepare the HPLC mobile phase (water and acetonitrile, both containing 0.1% formic acid). Inject the sample into the HPLC system, using a HILIC column for separation with ultra-performance liquid chromatography. Set the chromatographic temperature to 40°C, flow rate to 0.4 mL/min, and injection volume to 2 μL. Mobile phase A is water containing 25 mmol/L ammonium acetate and 25 mmol/L ammonia, while mobile phase B is acetonitrile. The gradient elution program is set as follows: 0-0.5 min: maintain 95% acetonitrile; 0.5-7 min: acetonitrile linearly decreases from 95% to 65%; 7-8 min: acetonitrile linearly decreases from 65% to 40%; 8-9 min: maintain 40% acetonitrile; 9-9.5 min: acetonitrile linearly increases from 40% to 95%; 9.5-12 min: maintain 95% acetonitrile. Use metabolite standards to establish calibration curves for the quantitative analysis of target metabolite concentrations in the samples. The separated metabolites were introduced into the mass spectrometer for analysis via electrospray ionization (ESI). The Q-TOF mass spectrometry conditions were set as follows: Ion Source Gas I: 50; Ion Source Gas II: 50; Curtain Gas: 30; Source Temperature: 500°C; Ion Spray Voltage: ±5500 V (in both negative and positive ion modes); Collision Energy: fixed at 35 ± 15 eV; Declustering Potential: ±80 V (in both negative and positive ion modes). Product ion scans were acquired using information-dependent acquisition (IDA) in high sensitivity mode. Mass spectrometry data processing software was used for peak identification and integration, and metabolite levels were compared across different treatment groups.

**WB Analysis**

Colon and esophageal tissues were collected from FADS1 gene knockout and control mice, washed with PBS, fixed in 4% paraformaldehyde, embedded in paraffin, and sectioned. Tissue samples were frozen in liquid nitrogen, ground into powder, and lysed with RIPA buffer containing protein and phosphatase inhibitors. The lysates were then incubated on ice for 30 minutes, sonicated for cell lysis, centrifuged to collect the supernatant, and the protein concentration was determined. The protein concentration was quantified using the BCA protein assay kit. Subsequently, 20 µg of protein samples were separated by SDS-PAGE and transferred to a PVDF membrane at a constant current of 300 mA for 90 minutes. The membrane was blocked with 5% non-fat milk for 1 hour, incubated overnight at 4°C with primary antibodies p-AKT, AKT, p-ERK, ERK, p-PI3K, PI3K (all at 1:1000 dilution, Cell Signaling Technology). The following day, the membrane was incubated for 1 hour at room temperature with an HRP-conjugated goat anti-rabbit secondary antibody (1:5000, Abcam), followed by visualization using an ECL detection kit and imaging of the protein bands with a chemiluminescence imaging system. The grayscale values of the protein bands were analyzed using ImageJ software to calculate the ratio of the target protein to the reference protein β-actin, comparing the differences in phosphorylation levels between groups.
