# Supplementary material for: Targeting FADS1-mediated lipid metabolism and signaling: a novel therapeutic strategy for precision oncology in colorectal and esophageal cancers
Source: Cell Death Discov. 2025 Oct 16;11:460. doi: 10.1038/s41420-025-02768-3 (PMC12533223; doi:10.1038/s41420-025-02768-3)
Supplement: Supplementary file 3 — Supplementary Tables [file 41420_2025_2768_MOESM3_ESM.docx]

| **Table S1. Information of QTL and GWAS datasets.** | | | | | | |  |
| --- | --- | --- | --- | --- | --- | --- | --- |
| **Type of dataset** | **Data subtype** | **Resource** | **Sample size** | **Population** | **Reference** | **Download Site** | |
| **QTL** | **cis-eQTL** | **V8 release of the GTEx eQTL** | **838** | **European** | **GTEx Consortium. Science 2021. PMID:32913098. The GTEx Consortium atlas of genetic regulatory effects across human tissues.** | **https://yanglab.westlake.edu.cn/software/smr/#eQTLsummarydata** | |
|  | **cis-mQTL** | **McRae et al. mQTL summary data** | **1980** | **European** | **Wu Y, et al. Nat Commun. 2018. PMID: 29500431. Integrative analysis of omics summary data reveals putative mechanisms underlying complex traits.** | **https://yanglab.westlake.edu.cn/data/SMR/LBC_BSGS_meta_lite.tar.gz** | |
| **GWAS summary** | **Gastroesophageal reflux** | **GWAS Catalog GCST90018848** | **case: 32,957, control: 434,296** | **European** | **Sakaue S, et al. Nat Genet. 2021. PMID: 34594039. A cross-population atlas of genetic associations for 220 human phenotypes.** | **https://www.ebi.ac.uk/gwas/studies/GCST90018848** | |
|  | **Esophageal cancer** | **GWAS Catalog GCST90018841** | **case: 998, control: 475,308** | **European** | **Sakaue S, et al. Nat Genet. 2021. PMID: 34594039. A cross-population atlas of genetic associations for 220 human phenotypes.** | **https://www.ebi.ac.uk/gwas/studies/GCST90018841** | |
|  | **Gastric ulcer** | **GWAS Catalog GCST90018851** | **case: 6,293, control:467,985** | **European** | **Sakaue S, et al. Nat Genet. 2021. PMID: 34594039. A cross-population atlas of genetic associations for 220 human phenotypes.** | **https://www.ebi.ac.uk/gwas/studies/GCST90018851** | |
|  | **Duodenal ulcer** | **FinnGen** | **case: 3795, control:350064** | **European** | **Kurki,et al. Nature. 2023 PMID:36653562 FinnGen provides genetic insights from a well-phenotyped isolated population.** | **gs://finngen-public-data-r9/summary_stats/finngen_R9_K11_DULC.gz** | |
|  | **Acute gastritis** | **FinnGen** | **case: 2370, control:320387** | **European** | **Kurki,et al. Nature. 2023 PMID:36653562 FinnGen provides genetic insights from a well-phenotyped isolated population.** | **gs://finngen-public-data-r9/summary_stats/finngen_R9_K11_ACUTGASTR.gz** | |
|  | **Chronic gastritis** | **GWAS Catalog GCST90018825** | **case: 3,645, control:441,451** | **European** | **Sakaue S, et al. Nat Genet. 2021. PMID: 34594039. A cross-population atlas of genetic associations for 220 human phenotypes.** | **https://www.ebi.ac.uk/gwas/studies/GCST90018825** | |
|  | **Gastric cancer** | **GWAS Catalog GCST90018849** | **case: 1,029, control:475,087** | **European** | **Sakaue S, et al. Nat Genet. 2021. PMID: 34594039. A cross-population atlas of genetic associations for 220 human phenotypes.** | **https://www.ebi.ac.uk/gwas/studies/GCST90018849** | |
|  | **Irritable bowel syndrome** | **GWAS Catalog GCST90016564** | **case: 53,400, control:433,201** | **European** | **Eijsbouts C, et al. Nat Aging. 2021. PMID:34741163. Genome-wide analysis of 53,400 people with irritable bowel syndrome highlights shared genetic pathways with mood and anxiety disorders.** | **https://www.ebi.ac.uk/gwas/studies/GCST90016564** | |
|  | **Ulcerative colitis** | **GWAS Catalog GCST90018933** | **case: 5,371, control:412,561** | **European** | **Sakaue S, et al. Nat Genet. 2021. PMID: 34594039. A cross-population atlas of genetic associations for 220 human phenotypes.** | **https://www.ebi.ac.uk/gwas/studies/GCST90018933** | |
|  | **Crohn's disease** | **FinnGen** | **case: 2004, control:359927** | **European** | **Kurki,et al. Nature. 2023 PMID:36653562 FinnGen provides genetic insights from a well-phenotyped isolated population.** | **gs://finngen-public-data-r9/summary_stats/finngen_R9_CHRONSMALL.gz** | |
|  | **Diverticular disease** | **GWAS Catalog GCST90038682** | **case: 5,218, control:479,380** | **European** | **Dönertaş HM, et al. Nat Aging. 2021. PMID: 33959723. Common genetic associations between age-related diseases. Apr;1(4):400-412. doi: 10.1038/s43587-021-00051-5. PubMed PMID: 33959723.** | **https://www.ebi.ac.uk/gwas/studies/GCST90038682** | |
|  | **Colorectal cancer** | **GWAS Catalog GCST90018808** | **case: 6,581, control:463,421** | **European** | **Sakaue S, et al. Nat Genet. 2021. PMID: 34594039. A cross-population atlas of genetic associations for 220 human phenotypes.** | **https://www.ebi.ac.uk/gwas/studies/GCST90018808** | |
|  | **Non-alcoholic fatty liver disease** | **GWAS Catalog GCST90054782** | **case: 6,581, control:463,421** | **European** | **Fairfield CJ,et al. Hepatol Commun.2021.PMID: 34535985. A. Genome-Wide Association Study of NAFLD Using Electronic Health Records. Hepatol Commun.** | **https://www.ebi.ac.uk/gwas/studies/GCST90054782** | |
|  | **Liver cancer** | **GWAS Catalog CST90018858** | **case: 379, control:475,259** | **European** | **Sakaue S, et al. Nat Genet. 2021. PMID: 34594039. A cross-population atlas of genetic associations for 220 human phenotypes.** | **https://www.ebi.ac.uk/gwas/studies/CST90018858** | |
|  | **Acute pancreatitis** | **GWAS Catalog GCST90018789** | **case: 3,798, control:476,104** | **European** | **Sakaue S, et al. Nat Genet. 2021. PMID: 34594039. A cross-population atlas of genetic associations for 220 human phenotypes.** | **https://www.ebi.ac.uk/gwas/studies/GCST90018789** | |
|  | **Chronic pancreatitis** | **GWAS Catalog GCST90018821** | **case: 1,424, control:476,104** | **European** | **Sakaue S, et al. Nat Genet. 2021. PMID: 34594039. A cross-population atlas of genetic associations for 220 human phenotypes.** | **https://www.ebi.ac.uk/gwas/studies/GCST90018821** | |
|  | **Pancreatic cancer** | **GWAS Catalog GCST90018893** | **case: 1,196, control:475,049** | **European** | **Sakaue S, et al. Nat Genet. 2021. PMID: 34594039. A cross-population atlas of genetic associations for 220 human phenotypes.** | **https://www.ebi.ac.uk/gwas/studies/GCST90018893** | |
|  | **Acute appendicitis** | **FinnGen** | **case: 28745, control:346283** | **European** | **Kurki,et al. Nature. 2023 PMID:36653562 FinnGen provides genetic insights from a well-phenotyped isolated population.** | **gs://finngen-public-data-r9/summary_stats/finngen_R9_K11_APPENDACUT.gz** | |
|  | **1400 plasma metabolites** | **GWAS Catalog GCST90199621-GCST90201020** | **n=8299** | **European** | **Chen Y, et al. Nat Genet. 2023 PubMed PMID: 36635386. Genomic atlas of the plasma metabolome prioritizes metabolites implicated in human diseases.** | **https://www.ebi.ac.uk/gwas/studies/GCST90199621-GCST90201020** | |

| **Table S2. The Mendelian randomization findings encompassing 1400 metabolites and 20 digestive disorders.** | | | | | | | | | |
| --- | --- | --- | --- | --- | --- | --- | --- | --- | --- |
| **Disease** | **exposure** | **nsnp** | **beta** | **se** | **pval** | **OR** | **95%CI(low）** | **95%CI(up）** | **p_FDR** |
| Colorectal cancer | Arachidonate to oleate to vaccenate ratio | 2 | 0.14 | 0.03 | 1.05E-06 | 1.15 | 1.08 | 1.22 | 1.22E-03 |
|  | Arachidonate to linoleate ratio | 9 | 0.13 | 0.03 | 8.31E-06 | 1.14 | 1.07 | 1.21 | 4.81E-03 |
|  | 1,2-dipalmitoyl-gpc levels | 14 | 0.14 | 0.03 | 2.00E-05 | 1.15 | 1.08 | 1.22 | 5.44E-03 |
|  | 1,2-dilinoleoyl-GPC levels | 10 | -0.16 | 0.04 | 2.34E-05 | 0.85 | 0.79 | 0.92 | 5.44E-03 |
|  | 1-oleoyl-2-linoleoyl-GPE levels | 16 | -0.09 | 0.02 | 2.35E-05 | 0.91 | 0.88 | 0.95 | 5.44E-03 |
|  | 1-palmitoyl-2-arachidonoyl-gpc levels | 4 | 0.08 | 0.02 | 3.41E-04 | 1.08 | 1.04 | 1.13 | 5.63E-02 |
|  | Phosphate to linoleoyl-arachidonoyl-glycerol ratio | 7 | -0.12 | 0.03 | 3.33E-04 | 0.89 | 0.84 | 0.94 | 5.63E-02 |
|  | Uridine to pseudouridine ratio | 3 | -0.39 | 0.11 | 4.38E-04 | 0.68 | 0.55 | 0.84 | 6.34E-02 |
|  | Adrenate levels | 10 | 0.23 | 0.07 | 6.02E-04 | 1.26 | 1.1 | 1.44 | 7.74E-02 |
| Esophageal cancer | Docosapentaenoate levels | 12 | 0.53 | 0.13 | 5.38E-05 | 1.7 | 1.59 | 1.82 | 4.95E-02 |
|  | X-23641 levels | 22 | -0.29 | 0.08 | 2.01E-04 | 0.75 | 0.68 | 0.81 | 1.11E-01 |
|  | 2-hydroxybutyrate/2-hydroxyisobutyrate levels | 2 | -1.02 | 0.3 | 6.06E-04 | 0.36 | 0.11 | 0.61 | 1.68E-01 |
|  | X-11632 levels | 15 | -0.42 | 0.12 | 5.76E-04 | 0.66 | 0.56 | 0.76 | 1.68E-01 |
|  | Mannose to mannitol to sorbitol ratio | 8 | 0.49 | 0.15 | 9.31E-04 | 1.63 | 1.51 | 1.77 | 2.06E-01 |
| Pancreatic cancer | Valine levels | 8 | 0.65 | 0.17 | 1.39E-04 | 1.92 | 1.76 | 2.05 | 1.95E-01 |
|  | 1-stearoyl-2-arachidonoyl-GPI levels | 17 | -0.27 | 0.08 | 3.14E-04 | 0.76 | 0.7 | 0.83 | 2.00E-01 |
| Liver cancer | Adenosine 5'-diphosphate (ADP) to glutamine ratio | 10 | 0.47 | 0.13 | 2.53E-04 | 1.6 | 1.5 | 1.72 | 3.54E-01 |
| Gastric cancer | Palmitate levels | 11 | -0.36 | 0.11 | 1.00E-03 | 0.7 | 0.6 | 0.79 | 6.48E-01 |
| Nonalcoholic fatty liver disease | Bilirubin degradation product levels | 10 | 0.15 | 0.04 | 6.29E-05 | 1.16 | 1.13 | 1.2 | 5.29E-02 |
|  | Glucose-to-mannose ratio | 10 | 0.31 | 0.08 | 8.83E-05 | 1.36 | 1.29 | 1.43 | 5.29E-02 |
|  | Bilirubin (z,z) levels | 15 | 0.13 | 0.03 | 1.63E-04 | 1.14 | 1.11 | 1.16 | 6.50E-02 |
|  | Biliverdin levels | 16 | 0.13 | 0.04 | 2.51E-04 | 1.14 | 1.11 | 1.17 | 7.52E-02 |
|  | Imidazole lactate levels | 16 | -0.11 | 0.03 | 4.82E-04 | 0.9 | 0.87 | 0.92 | 1.05E-01 |
|  | Phosphate to glutamate ratio | 15 | -0.26 | 0.08 | 5.88E-04 | 0.77 | 0.7 | 0.83 | 1.05E-01 |
|  | Mannose to glycerol ratio | 11 | -0.41 | 0.12 | 6.11E-04 | 0.66 | 0.56 | 0.77 | 1.05E-01 |
| Inflammatory bowel disease | Mannose to glycerol ratio | 11 | -0.18 | 0.04 | 2.07E-05 | 0.84 | 0.79 | 0.87 | 2.90E-02 |
| Ulcerative colitis | N-acetyl-isoputreanine levels | 20 | 0.15 | 0.04 | 6.63E-05 | 1.16 | 1.13 | 1.2 | 8.47E-02 |
| Crohns disease | Sphingomyelin levels | 15 | -0.32 | 0.09 | 6.23E-04 | 0.73 | 0.65 | 0.81 | 4.23E-01 |
| Irritable bowel syndrome | 1-stearoyl-2-linoleoyl-GPI levels | 17 | -0.06 | 0.02 | 9.52E-05 | 0.94 | 0.92 | 0.95 | 1.33E-01 |
|  | Urea levels | 12 | -0.11 | 0.03 | 1.91E-04 | 0.9 | 0.87 | 0.92 | 1.33E-01 |
| Acute gastritis | Nervonoylcarnitine levels | 15 | 0.37 | 0.1 | 1.53E-04 | 1.45 | 1.37 | 1.54 | 1.07E-01 |
|  | Mannose to fructose ratio | 11 | -0.65 | 0.17 | 8.27E-05 | 0.52 | 0.38 | 0.66 | 1.07E-01 |
| Acute pancreatitis | (R)-3-hydroxybutyrylcarnitine levels | 10 | 0.34 | 0.09 | 1.36E-04 | 1.4 | 1.33 | 1.49 | 1.91E-01 |
| Acute appendicitis | X-12462 levels | 7 | -0.23 | 0.06 | 4.62E-05 | 0.79 | 0.75 | 0.84 | 6.47E-02 |
| Alcoholic liver disease | 1-oleoyl-GPI levels | 18 | 0.34 | 0.09 | 1.65E-04 | 1.4 | 1.32 | 1.48 | 1.91E-01 |
| Chronic pancreatitis | 1-palmitoyl-2-dihomo-linolenoyl-GPC levels | 16 | 0.28 | 0.07 | 1.39E-04 | 1.32 | 1.26 | 1.39 | 1.95E-01 |
| Chronic gastritis | Laurate levels | 9 | 0.37 | 0.12 | 2.00E-03 | 1.45 | 1.34 | 1.54 | 9.99E-01 |
| Diverticular disease | 4-acetaminophen sulfate levels | 12 | 0 | 0 | 3.25E-04 | 1 | 1 | 1 | 4.25E-01 |
| Duodenal ulcer | Histidine to alanine ratio | 3 | 0.75 | 0.29 | 9.91E-03 | 2.12 | 1.87 | 2.37 | 1.00E+00 |
| Gastric ulcer | Gamma-glutamylcitrulline levels | 19 | -0.11 | 0.03 | 8.86E-04 | 0.9 | 0.87 | 0.93 | 9.31E-01 |
| Gastroesophageal reflux disease | X-24243 levels | 13 | 0.09 | 0.03 | 6.77E-04 | 1.09 | 1.07 | 1.12 | 5.08E-01 |
| The MR analysis method employed in the table was inverse variance weighted, and random or fixed effect models were selected based on the magnitude of heterogeneity. Only results with adjusted P<0.20 were presented. In cases where certain outcomes did not meet the criteria (adjusted P<0.20), only the result with the smallest adjusted P value was displayed. OR, odd ratio; CI, condifence interval; | | | | | | | | | |

| **Table S3. The results of a sensitivity analysis using Mendelian randomization were conducted on 1400 metabolites.** | | | | | | | | | | | | | |
| --- | --- | --- | --- | --- | --- | --- | --- | --- | --- | --- | --- | --- | --- |
| **Disease** | **exposure** | **nsnp** | **MR Egger** | | **Inverse variance weighted** | | **I^2** | **F statistic** | **MR.PRESSO** | | **pleiotropy** | | |
|  |  |  | **Q** | **Pvalue** | **Q** | **Pvalue** |  |  | **Global.Test** | **Pvalue** | **egger intercept** | **se** | **pval** |
| Pancreatic cancer | Valine levels | 8 | 15.38361821 | 0.652635417 | 14.37846174 | 0.43735135 | 0.0%. | 23.3 | 14.05244723 | 0.702 | 0.05226704 | 0.05885075 | 0.4086609 |
|  | 1-stearoyl-2-arachidonoyl-GPI levels | 17 | 18.37235143 | 0.374634618 | 19.383516 | 0.46373468 | 0.0%. | 53.1 | 6.375245378 | 0.718 | 0.02414907 | 0.03071091 | 0.4439169 |
| Esophageal cancer | Docosapentaenoate levels | 12 | 4.679216527 | 0.91155147 | 4.847986997 | 0.938304175 | 0.00% | 27.6 | 5.45905 | 0.948 | 0.01620869 | 0.03945478 | 0.6898715 |
|  | X-23641 levels | 22 | 12.03298441 | 0.017107663 | 14.07521233 | 0.01513865 | 25.40% | 33.4 | 31.81651 | 0.149 | -0.04418501 | 0.03172647 | 0.1782859 |
|  | 2-hydroxybutyrate/2-hydroxyisobutyrate levels | 2 | 7.27732355 | 0.887299879 | 7.289256987 | 0.922985096 | 64.50% | 25.2 | 22.09364 | 0.131 | -0.07334193 | 0.08901371 | 0.456266 |
|  | X-11632 levels | 15 | 26.99479684 | 0.171025468 | 29.48805391 | 0.131447234 | 0.00% | 24.7 | 8.439251 | 0.937 | 0.003787697 | 0.03467308 | 0.9146802 |
|  | Mannose to mannitol to sorbitol ratio | 8 | 7.704917046 | 0.260528665 | 8.014230653 | 0.33134073 | 12.70% | 24.5 | 11.6694 | 0.332 | 0.03513307 | 0.07158548 | 0.6410104 |
| Acute gastritis | Nervonoylcarnitine levels | 15 | 14.57774454 | 0.33444924 | 14.63525486 | 0.403533322 | 4.30% | 45.5 | 16.59588082 | 0.437 | 0.007936083 | 0.03504344 | 0.8243624 |
|  | Mannose to fructose ratio | 11 | 10.7845206 | 0.290768672 | 10.80198115 | 0.37315228 | 7.40% | 24.4 | 12.95174515 | 0.408 | 0.005316037 | 0.04403908 | 0.9065713 |
| Irritable bowel syndrome | 1-stearoyl-2-linoleoyl-GPI levels | 17 | 14.56369982 | 0.483276557 | 14.98554449 | 0.52569753 | 0.00% | 47.8 | 15.94120538 | 0.622 | 0.003631055 | 0.005590575 | 0.5258396 |
|  | Urea levels | 12 | 8.926796698 | 0.539064686 | 10.12950015 | 0.518777567 | 0.00% | 23 | 12.17582305 | 0.555 | -0.01047836 | 0.009554633 | 0.2984885 |
| Inflammatory bowel disease | Mannose to glycerol ratio | 11 | 8.122305163 | 0.521869419 | 9.26668583 | 0.506995075 | 0.00% | 33.5 | 11.52621026 | 0.558 | -0.01236045 | 0.01155444 | 0.3125783 |
| Ulcerative colitis | N-acetyl-isoputreanine levels | 20 | 20.00558006 | 0.520907825 | 21.65381113 | 0.480707122 | 0.00% | 61.2 | 23.76850475 | 0.5 | -0.01424004 | 0.0110918 | 0.2131851 |
| Colorectal cancer | Arachidonate to oleate to vaccenate ratio | 2 | 15.62051035 | 0.075241838 | 17.86093874 | 0.057355206 | 44.00% | 95.4 | 41.26702772 | 0.257 | -0.01376587 | 0.01211615 | 0.2852374 |
|  | Arachidonate to linoleate ratio | 9 | 13.99524998 | 0.173208381 | 16.24035699 | 0.132439677 | 32.30% | 81.2 | 34.05047272 | 0.263 | -0.01115132 | 0.008804364 | 0.2340158 |
|  | 1,2-dipalmitoyl-gpc levels | 14 | 12.6250382 | 0.39687294 | 12.69703286 | 0.47147664 | 0.00% | 45.8 | 21.09835992 | 0.314 | -0.002600935 | 0.009942719 | 0.798072 |
|  | 1,2-dilinoleoyl-GPC levels | 10 | 6.472395404 | 0.594471157 | 10.38220257 | 0.320438675 | 13.30% | 56.5 | 18.70185525 | 0.354 | 0.00874688 | 0.009480941 | 0.18339345 |
|  | 1-oleoyl-2-linoleoyl-GPE levels | 16 | 16.75779448 | 0.333548479 | 20.30988603 | 0.206586662 | 21.20% | 77.9 | 31.56813804 | 0.159 | 0.01492138 | 0.008368156 | 0.09481103 |
|  | 1-palmitoyl-2-arachidonoyl-gpc levels | 4 | 11.11729942 | 0.676797534 | 11.59171616 | 0.709632856 | 0.00% | 113.7 | 11.96788183 | 0.844 | -0.004916176 | 0.007137521 | 0.5022178 |
|  | Phosphate to linoleoyl-arachidonoyl-glycerol ratio | 7 | 15.923587 | 0.81966003 | 21.19278258 | 0.569315693 | 0.00% | 54.5 | 19.76787092 | 0.354 | 0.01641283 | 0.01011175 | 0.1356228 |
|  | Uridine to pseudouridine ratio | 3 | 20.58223513 | 0.195133426 | 21.31891558 | 0.212349064 | 20.30% | 16.6 | 23.47165587 | 0.229 | -0.01281565 | 0.01693508 | 0.4601978 |
|  | Adrenate levels | 10 | 15.45610971 | 0.050860354 | 17.52197707 | 0.041142357 | 48.60% | 33.5 | 24.18921411 | 0.091 | -0.01853989 | 0.01792921 | 0.3313518 |
| Nonalcoholic fatty liver disease | Bilirubin degradation product levels | 10 | 8.563578466 | 0.662105297 | 8.563580707 | 0.739684862 | 0.0%. | 111.9 | 13.6137 | 0.688 | 1.97E-05 | 0.01319144 | 0.9988323 |
|  | Glucose-to-mannose ratio | 10 | 9.74052027 | 0.371897177 | 17.30829065 | 0.067814615 | 42.20% | 50.4 | 48.58184 | 0.112 | 0.06339561 | 0.02397425 | 0.42672382 |
|  | Bilirubin (z,z) levels | 15 | 16.10385944 | 0.307072931 | 16.18105273 | 0.370121918 | 7.30% | 127.7 | 19.73158 | 0.444 | -0.003890676 | 0.01501883 | 0.7993644 |
|  | Biliverdin levels | 16 | 6.101693272 | 0.977988433 | 7.148916099 | 0.970282806 | 0.0%. | 104.5 | 10.29514 | 0.944 | -0.01026573 | 0.01003161 | 0.322369 |
|  | Imidazole lactate levels | 16 | 13.69139241 | 0.47294655 | 13.70119678 | 0.548301096 | 0.00% | 126.8 | 16.22713 | 0.561 | -0.000977065 | 0.009867639 | 0.9225285 |
|  | Phosphate to glutamate ratio | 15 | 13.12898587 | 0.437898555 | 13.63412171 | 0.477309582 | 0.00% | 23.4 | 15.63274 | 0.525 | 0.01699783 | 0.02403441 | 0.4919081 |
|  | Mannose to glycerol ratio | 11 | 31.41715473 | 0.000945395 | 32.23282319 | 0.001273778 | 62.8%. | 32.4 | 51.40169 | **0.002** | 0.01791454 | 0.03352246 | 0.6036952 |
| Liver cancer | Adenosine 5'-diphosphate (ADP) to glutamine ratio | 10 | 5.559370471 | 0.696453741 | 7.487129449 | 0.586537755 | 0.00% | 22.3 | 9.101157619 | 0.673 | 0.09620263 | 0.06928841 | 0.2024396 |
|  | Mannose to trans-4-hydroxyproline ratio | 10 | 6.10687902 | 0.635261087 | 6.272223729 | 0.712394047 | 0.00% | 29.8 | 9.368250846 | 0.656 | -0.01597274 | 0.03928115 | 0.6949437 |
| Acute pancreatitis | (R)-3-hydroxybutyrylcarnitine levels | 10 | 1.675215769 | 0.989402629 | 2.385995338 | 0.983793877 | 0.00% | 25.5 | 2.951263136 | 0.986 | 0.02749128 | 0.03260825 | 0.4236737 |
| Chronic pancreatitis | 1-palmitoyl-2-dihomo-linolenoyl-GPC levels | 16 | 8.228578851 | 0.877080733 | 8.235529965 | 0.913993058 | 0.00% | 21.7 | 8.809680527 | 0.931 | -0.005548124 | 0.02809302 | 0.8490555 |
| Acute appendicitis | X-12462 levels | 7 | 3.33414339 | 0.648618539 | 3.425655114 | 0.753833247 | 0.00% | 22 | 4.819720131 | 0.771 | -0.004350178 | 0.01438032 | 0.774444 |
| Cochran Q statistic implemented in MR Egger and IVW method, P>0.05 indicates no heterogeneity exists. The intercept of MR Egger can be used to indicate whether directional horizontal pleiotropy is driving the results of MR analysis, there are no directional pleiotropies if P>0.05.  MR-PRESSO can detect and adjust for any outliers reflecting horizontal pleiotropic biases, where p value for Global test > 0.05 indicates no horizontal pleiotropic outliers. The I^2^ statistic was calculated to assess the heterogeneity of each outcome from different data sources, and the I^2^values <25%，25-75%,and >75% were considered to indicate low moderate,and high heterogeneity, respectively. | | | | | | | | | | | | | |

| **Table S4. The LDSC results of 1400 metabolites were analyzed in relation to various digestive diseases.** | | | | | | |
| --- | --- | --- | --- | --- | --- | --- |
| **exposure** | **exposure** | **Genetic correlation** | **SE** | P value for LDSC | **Intercept** | **Intercept (SE)** |
| Colorectal cancer | Arachidonate to oleate to vaccenate ratio | 3.33E-01 | 9.16E-02 | **8.10E-03** | 6.50E-03 | 3.70E-03 |
|  | Arachidonate to linoleate ratio | 1.78E-01 | 8.43E-02 | **2.31E-02** | 3.90E-03 | 3.60E-03 |
|  | 1,2-dipalmitoyl-gpc levels | 2.02E-01 | 1.60E-01 | 2.06E-01 | -9.40E-03 | 3.80E-03 |
|  | 1,2-dilinoleoyl-GPC levels | 3.54E-01 | 1.29E-01 | **3.64E-02** | 8.00E-04 | 3.60E-03 |
|  | 1-oleoyl-2-linoleoyl-GPE levels | -1.67E-01 | 8.45E-02 | **3.73E-02** | 6.30E-03 | 3.70E-03 |
|  | 1-palmitoyl-2-arachidonoyl-gpc levels | 9.77E-02 | 1.38E-01 | 4.79E-01 | -3.30E-03 | 3.70E-03 |
|  | Phosphate to linoleoyl-arachidonoyl-glycerol ratio | 1.87E-01 | 1.22E-01 | 1.24E-01 | -7.60E-03 | 3.40E-03 |
|  | Uridine to pseudouridine ratio | 1.65E-01 | 1.45E-01 | 1.55E-01 | -2.40E-03 | 3.90E-03 |
|  | Adrenate levels | -2.49E-02 | 2.09E-01 | 9.05E-01 | -6.00E-04 | 3.50E-03 |
| Esophageal cancer | Docosapentaenoate levels | 2.15E-01 | -9.07E-02 | **1.18E-02** | -1.90E-03 | 4.30E-03 |
|  | X-23641 levels | 2.80E-01 | 2.19E-01 | 2.02E-01 | 8.00E-04 | 6.00E-04 |
|  | 2-hydroxybutyrate/2-hydroxyisobutyrate levels | -5.56E-01 | 2.76E-01 | **3.44E-02** | -5.00E-04 | 6.00E-04 |
|  | X-11632 levels | -1.75E-01 | 1.10E-01 | 7.99E-02 | 6.00E-04 | 7.00E-04 |
|  | Mannose to mannitol to sorbitol ratio | 4.75E-01 | 5.50E-01 | 3.88E-01 | 6.00E-04 | 6.00E-04 |
| Pancreatic cancer | Valine levels | 3.97E-01 | 1.90E-01 | **2.57E-02** | 3.60E-03 | 4.20E-03 |
|  | 1-stearoyl-2-arachidonoyl-GPI levels | -2.68E-01 | 2.27E-01 | 2.12E-01 | 7.80E-03 | 3.80E-03 |
| Irritable bowel syndrome | 1-stearoyl-2-linoleoyl-GPI levels | -4.44E-01 | 1.81E-01 | **2.74E-02** | 4.90E-03 | 4.50E-03 |
|  | Urea levels | -4.88E-01 | 2.21E-01 | **4.34E-02** | -2.70E-03 | 4.10E-03 |
| Inflammatory bowel disease | Mannose to glycerol ratio | -4.71E-01 | 1.65E-01 | 3.09E-02 | 1.80E-03 | 3.90E-03 |
| Ulcerative colitis | N-acetyl-isoputreanine levels | 5.14E-01 | 2.66E-01 | 2.96E-02 | -3.20E-03 | 3.90E-03 |
| Nonalcoholic fatty liver disease | Bilirubin degradation product levels | 5.49E-01 | 1.64E-01 | **8.00E-04** | 2.50E-03 | 4.50E-03 |
|  | Glucose-to-mannose ratio | 3.88E-01 | 1.11E-01 | **3.28E-02** | 1.50E-03 | 5.50E-03 |
|  | Bilirubin (z,z) levels | 2.34E-01 | 8.06E-02 | **2.60E-02** | -4.70E-03 | 5.90E-03 |
|  | Biliverdin levels | 2.15E-01 | 1.18E-01 | **4.04E-02** | -6.40E-03 | 5.60E-03 |
|  | Imidazole lactate levels | -3.19E-01 | 9.67E-02 | **2.88E-02** | 5.20E-03 | 4.10E-03 |
|  | Phosphate to glutamate ratio | -6.90E-01 | 1.55E-01 | **8.48E-06** | -5.01E-05 | 4.70E-03 |
|  | Mannose to glycerol ratio | 1.87E-01 | 9.27E-01 | 1.69E-01 | -5.60E-03 | 4.80E-03 |
| Acute gastritis | Nervonoylcarnitine levels | 3.12E-01 | 1.17E-01 | **3.98E-02** | 7.80E-03 | 3.80E-03 |
|  | Mannose to fructose ratio | -3.71E-01 | 2.86E-01 | 1.93E-01 | 7.40E-03 | 4.10E-03 |
| Acute pancreatitis | (R)-3-hydroxybutyrylcarnitine levels | 5.19E-01 | 1.37E-01 | **8.80E-03** | 5.20E-03 | 4.10E-03 |
| Chronic pancreatitis | 1-palmitoyl-2-dihomo-linolenoyl-GPC levels | 1.69E-01 | 6.31E-02 | **1.50E-02** | 6.70E-03 | 4.90E-03 |
| Pancreatic cancer | Valine levels | 3.97E-01 | 1.90E-01 | **2.57E-02** | 3.60E-03 | 4.20E-03 |
|  | 1-stearoyl-2-arachidonoyl-GPI levels | -2.68E-01 | 2.27E-01 | 2.12E-01 | 7.80E-03 | 3.80E-03 |
| Acute appendicitis | X-12462 levels | -2.47E-01 | 2.18E-01 | 2.00E-01 | 5.20E-03 | 4.10E-03 |

| **Table S5. SMR and colocalization results from eQTL of diverse metabolites causally associated with cancer outcomes** | | | | | | | | | | | | | | | | | | | | | | | | |
| --- | --- | --- | --- | --- | --- | --- | --- | --- | --- | --- | --- | --- | --- | --- | --- | --- | --- | --- | --- | --- | --- | --- | --- | --- |
| **Type of disease** | **Type of metabolites** | **Probe** | **Gene Chr.** | **Gene** | **Probe base pair** | **topSNP** | **Gene Chr.** | **Probe base pair** | **Effect allele** | **Other allele** | **Effect allele frequence** | **GWAS association** | | | **eQTL association** | | | **SMR association** | | | **HEIDI Test** | | **BH correction** | **PP.H4** |
|  |  |  |  |  |  |  |  |  |  |  |  | **β** | **SE** | ***P*** | **β** | **SE** | ***P*** | **β** | **SE** | ***P*** | ***P*** | **nsnp** | ***P-FDR*** |  |
| colon cancer | 1,2-dipalmitoyl-gpc levels | ENSG00000204304 | 6 | PBX2 | 32152512 | rs204995 | 6 | 32154285 | G | A | 0.2167 | 0.0807707 | 0.017662 | 4.80E-06 | -0.135199 | 0.0176371 | 1.78E-14 | -0.597421 | 0.152118 | 8.59E-05 | 0.11415362 | 20 | 5.82E-03 | 0.8635723 |
|  |  | ENSG00000196301 | 6 | HLA-DRB9 | 32427598 | rs9271418 | 6 | 32587832 | G | A | 0.66501 | 0.0917897 | 0.016626 | 3.37E-08 | 0.654765 | 0.044074 | 6.36E-50 | 0.140187 | 0.027089 | 2.28E-07 | 0.1046643 | 20 | 3.09E-04 | 0.0038467 |
|  |  | ENSG00000198502 | 6 | HLA-DRB5 | 32485120 | rs35083819 | 6 | 32526526 | G | A | 0.852883 | 0.0973767 | 0.0210483 | 3.72E-06 | -0.950592 | 0.0391641 | 3.87E-130 | -0.102438 | 0.0225409 | 5.51E-06 | 0.09482306 | 20 | 5.33E-03 | 0.8372351 |
|  |  | ENSG00000196735 | 6 | HLA-DQA1 | 32595956 | rs17843580 | 6 | 32615551 | G | A | 0.506958 | 0.0848077 | 0.015541 | 4.84E-08 | -0.356525 | 0.0205582 | 2.26E-67 | -0.237873 | 0.0456973 | 1.94E-07 | 1 | 20 | 3.09E-04 | 0.8937631 |
|  |  | ENSG00000179344 | 6 | HLA-DQB1 | 32627244 | rs3134993 | 6 | 32637778 | A | G | 0.592445 | 0.0834534 | 0.0155692 | 8.32E-08 | -0.775797 | 0.0178434 | 0 | -0.107571 | 0.0202206 | 1.04E-07 | 1 | 20 | 2.35E-04 | 0.7846583 |
|  |  | ENSG00000232629 | 6 | HLA-DQB2 | 32723875 | rs9274660 | 6 | 32636434 | G | A | 0.595427 | 0.0847367 | 0.0155465 | 5.02E-08 | 0.972685 | 0.0244622 | 0 | 0.0871163 | 0.0161325 | 6.66E-08 | 1 | 20 | 2.26E-04 | 0.8364235 |
|  |  | ENSG00000134825 | 11 | TMEM258 | 61535973 | rs61896141 | 11 | 61556039 | C | A | 0.152087 | -0.146323 | 0.0200084 | 2.61E-13 | 0.187231 | 0.0274773 | 9.49E-12 | -0.781511 | 0.156762 | 6.19E-07 | 0.090345289 | 16 | 6.99E-04 | 0.0346832 |
|  |  | ENSG00000149485 | 11 | FADS1 | 61567099 | rs968567 | 11 | 61595564 | T | C | 0.150099 | -0.143304 | 0.0199298 | 6.46E-13 | 0.289897 | 0.0510035 | 1.32E-08 | -0.494327 | 0.110861 | 8.23E-06 | 0.06130948 | 9 | 6.98E-03 | 0.8658254 |
|  | Adrenate levels | ENSG00000124920 | 11 | MYRF | 61520114 | rs198462 | 11 | 61524119 | A | G | 0.494036 | 0.0684054 | 0.0151753 | 6.55E-06 | -0.328849 | 0.0309449 | 2.23E-26 | -0.208015 | 0.0501266 | 3.33E-05 | 0.3776548 | 20 | 5.64E-02 | 0.0836452 |
|  | 1,2-dilinoleoyl-GPC levels | ENSG00000124920 | 11 | MYRF | 61520114 | rs198462 | 11 | 61524119 | A | G | 0.494036 | -0.0934541 | 0.0150298 | 5.04E-10 | -0.328849 | 0.0309449 | 2.23E-26 | 0.284185 | 0.052953 | 8.02E-08 | 0.08857294 | 20 | 1.96E-04 | 0.0073646 |
|  |  | ENSG00000149485 | 11 | FADS1 | 61567099 | rs968567 | 11 | 61595564 | T | C | 0.150099 | 0.169295 | 0.0196407 | 6.72E-18 | 0.289897 | 0.0510035 | 1.32E-08 | 0.583983 | 0.123071 | 2.08E-06 | 0.060328143 | 9 | 3.53E-03 | 0.8735813 |
|  | 1-oleoyl-2-linoleoyl-GPE levels | ENSG00000124920 | 11 | MYRF | 61520114 | rs198462 | 11 | 61524119 | A | G | 0.494036 | -0.153319 | 0.0153213 | 1.42E-23 | -0.328849 | 0.0309449 | 2.23E-26 | 0.466229 | 0.063996 | 3.21E-13 | 0.06017877 | 20 | 1.09E-09 | 0.187634 |
|  |  | ENSG00000149485 | 11 | FADS1 | 61567099 | rs968567 | 11 | 61595564 | T | C | 0.150099 | 0.27031 | 0.0199284 | 6.54E-42 | 0.289897 | 0.0510035 | 1.32E-08 | 0.932435 | 0.17787 | 1.59E-07 | 0.100847874 | 9 | 2.69E-04 | 0.7749377 |
|  |  | ENSG00000183426 | 16 | NPIPA1 | 15016659 | rs3198697 | 16 | 15129940 | T | C | 0.397614 | -0.0824601 | 0.0156211 | 1.30E-07 | -0.406531 | 0.0363872 | 5.57E-29 | 0.202838 | 0.0424985 | 1.82E-06 | 0.08595893 | 20 | 2.05E-03 | 0.0083765 |
|  |  | ENSG00000157045 | 16 | NTAN1 | 15131710 | rs11644601 | 16 | 15172118 | C | T | 0.290258 | -0.0864721 | 0.0169913 | 3.60E-07 | -0.20327 | 0.0276247 | 1.86E-13 | 0.425405 | 0.101635 | 2.84E-05 | 0.06811751 | 10 | 2.75E-02 | 0.0084762 |
|  |  | ENSG00000183793 | 16 | NPIPA5 | 15457516 | rs3198697 | 16 | 15129940 | T | C | 0.397614 | -0.0824601 | 0.0156211 | 1.30E-07 | 0.800236 | 0.0486044 | 6.63E-61 | -0.103045 | 0.0204994 | 4.99E-07 | 0.06290726 | 20 | 6.76E-04 | 0.8318469 |
|  | 1-palmitoyl-2-arachidonoyl-gpc levels | ENSG00000116641 | 1 | DOCK7 | 62920399 | rs1168086 | 1 | 63112389 | G | A | 0.694831 | 0.0741704 | 0.0160571 | 3.85E-06 | 0.330647 | 0.0337641 | 1.21E-22 | 0.224319 | 0.0536939 | 2.94E-05 | 0.1776528 | 20 | 2.22E-02 | 0.8846623 |
|  |  | ENSG00000180953 | 15 | ST20 | 80191182 | rs12438337 | 15 | 80207919 | G | A | 0.225646 | -0.0778684 | 0.0189236 | 3.87E-05 | -0.329192 | 0.0191326 | 2.40E-66 | 0.236544 | 0.0591061 | 6.28E-05 | 0.719464 | 20 | 4.26E-02 | 0.0038361 |
|  |  | ENSG00000157045 | 16 | NTAN1 | 15131710 | rs11644601 | 16 | 15172118 | C | T | 0.290258 | 0.116362 | 0.0169175 | 6.06E-12 | -0.20327 | 0.0276247 | 1.86E-13 | -0.57245 | 0.113926 | 5.04E-07 | 0.1708491 | 10 | 4.88E-04 | 0.0038365 |
|  | Arachidonate to oleate to vaccenate ratio | ENSG00000124920 | 11 | MYRF | 61520114 | rs198462 | 11 | 61524119 | A | G | 0.494036 | 0.147622 | 0.0153185 | 5.59E-22 | -0.328849 | 0.0309449 | 2.23E-26 | -0.448905 | 0.0628833 | 9.42E-13 | 0.07266128 | 20 | 3.19E-09 | 0.0004767 |
|  |  | ENSG00000149485 | 11 | FADS1 | 61567099 | rs968567 | 11 | 61595564 | T | C | 0.150099 | -0.272964 | 0.0198917 | 7.44E-43 | 0.289897 | 0.0510035 | 1.32E-08 | -0.94159 | 0.179308 | 1.51E-07 | 0.080450891 | 9 | 2.56E-04 | 0.7937466 |
|  |  | ENSG00000157045 | 16 | NTAN1 | 15131710 | rs11644601 | 16 | 15172118 | C | T | 0.290258 | 0.0880392 | 0.0169714 | 2.13E-07 | -0.20327 | 0.0276247 | 1.86E-13 | -0.433115 | 0.102154 | 2.24E-05 | 0.1243315 | 10 | 2.53E-02 | 0.0172364 |
|  |  | ENSG00000183793 | 16 | NPIPA5 | 15457516 | rs3198697 | 16 | 15129940 | T | C | 0.397614 | 0.07034 | 0.0156308 | 6.79E-06 | 0.800236 | 0.0486044 | 6.63E-61 | 0.0878991 | 0.0202492 | 1.42E-05 | 0.101769014 | 20 | 1.92E-02 | 0.9193763 |
|  | Uridine to pseudouridine ratio | ENSG00000183696 | 7 | UPP1 | 48128225 | rs3752889 | 7 | 48129371 | G | C | 0.5666 | -0.0961793 | 0.0146669 | 5.47E-11 | 0.0782783 | 0.0131891 | 2.94E-09 | -1.22868 | 0.279222 | 1.08E-05 | 0.2061733 | 6 | 3.66E-02 | 0.0383613 |
|  |  | ENSG00000130489 | 22 | SCO2 | 50961997 | rs131805 | 22 | 50964153 | C | T | 0.775348 | -0.106083 | 0.0176248 | 1.76E-09 | -0.673941 | 0.0379301 | 1.25E-70 | 0.157407 | 0.0276116 | 1.19E-08 | 0.057800849 | 20 | 8.09E-05 | 0.1826369 |
|  | Phosphate to linoleoyl-arachidonoyl-glycerol ratio | ENSG00000124920 | 11 | MYRF | 61520114 | rs198462 | 11 | 61524119 | A | G | 0.494036 | -0.0802536 | 0.01592 | 4.63E-07 | -0.328849 | 0.0309449 | 2.23E-26 | 0.244044 | 0.053582 | 5.25E-06 | 0.09241368 | 20 | 8.89E-03 | 0.6383623 |
|  |  | ENSG00000149485 | 11 | FADS1 | 61567099 | rs968567 | 11 | 61595564 | T | C | 0.150099 | 0.216176 | 0.0209897 | 7.11E-25 | 0.289897 | 0.0510035 | 1.32E-08 | 0.745699 | 0.149849 | 6.48E-07 | 0.07260474 | 9 | 1.46E-03 | 0.4328173 |
|  | Arachidonate to linoleate ratio | ENSG00000124920 | 11 | MYRF | 61520114 | rs198462 | 11 | 61524119 | A | G | 0.494036 | 0.123312 | 0.0152194 | 5.39E-16 | -0.328849 | 0.0309449 | 2.23E-26 | -0.374981 | 0.058198 | 1.17E-10 | 0.06782945 | 20 | 3.96E-07 | 0.0182634 |
|  |  | ENSG00000149485 | 11 | FADS1 | 61567099 | rs968567 | 11 | 61595564 | T | C | 0.150099 | -0.237511 | 0.0197655 | 2.91E-33 | 0.289897 | 0.0510035 | 1.32E-08 | -0.819294 | 0.159456 | 2.78E-07 | 0.060261837 | 9 | 4.70E-04 | 0.7783792 |
|  |  | ENSG00000183426 | 16 | NPIPA1 | 15016659 | rs3198697 | 16 | 15129940 | T | C | 0.397614 | 0.077166 | 0.0154964 | 6.37E-07 | -0.406531 | 0.0363872 | 5.57E-29 | -0.189816 | 0.0417335 | 5.41E-06 | 0.193181315 | 20 | 6.11E-03 | 0.0883663 |
|  |  | ENSG00000157045 | 16 | NTAN1 | 15131710 | rs11644601 | 16 | 15172118 | C | T | 0.290258 | 0.0847386 | 0.0168514 | 4.94E-07 | -0.20327 | 0.0276247 | 1.86E-13 | -0.416877 | 0.100411 | 3.30E-05 | 0.3041377 | 10 | 3.20E-02 | 0.0083761 |
|  |  | ENSG00000183793 | 16 | NPIPA5 | 15457516 | rs3198697 | 16 | 15129940 | T | C | 0.397614 | 0.077166 | 0.0154964 | 6.37E-07 | 0.800236 | 0.0486044 | 6.63E-61 | 0.0964291 | 0.0202311 | 1.88E-06 | 0.061158725 | 20 | 2.54E-03 | 0.7038451 |
| Esophageal cancer | Docosapentaenoate n3 levels | ENSG00000124920 | 11 | MYRF | 61520114 | rs198462 | 11 | 61524119 | A | G | 0.494036 | 0.0653018 | 0.0147128 | 9.06E-06 | -0.328849 | 0.0309449 | 2.23E-26 | -0.198577 | 0.0484857 | 4.21E-05 | 0.2867981 | 20 | 4.14E-02 | 0.0018365 |
|  |  | ENSG00000149485 | 11 | FADS1 | 61567099 | rs968567 | 11 | 61595564 | T | C | 0.150099 | -0.116291 | 0.0192583 | 1.56E-09 | 0.289897 | 0.0510035 | 1.32E-08 | -0.401146 | 0.0969235 | 3.49E-05 | 0.07179624 | 9 | 4.14E-02 | 0.7466293 |
|  | X-23641 levels | ENSG00000196177 | 10 | ACADSB | 124768495 | rs11592612 | 10 | 124770855 | G | C | 0.393638 | -0.124618 | 0.0167658 | 1.06E-13 | 0.132626 | 0.0219899 | 1.63E-09 | -0.93962 | 0.200629 | 2.82E-06 | NA | NA | 1.91E-02 | 0.0043761 |
| Pancreatic cancer | 1-stearoyl-2-arachidonoyl-GPI levels | ENSG00000116641 | 1 | DOCK7 | 62920399 | rs1168086 | 1 | 63112389 | G | A | 0.694831 | 0.194471 | 0.0157427 | 4.69E-35 | 0.330647 | 0.0337641 | 1.21E-22 | 0.588153 | 0.0766422 | 1.67E-14 | 0.08577641 | 20 | 3.77E-11 | 0.764993 |
|  |  | ENSG00000124920 | 11 | MYRF | 61520114 | rs198462 | 11 | 61524119 | A | G | 0.494036 | 0.0757993 | 0.0151769 | 5.90E-07 | -0.328849 | 0.0309449 | 2.23E-26 | -0.230499 | 0.0509944 | 6.18E-06 | 0.15958037 | 20 | 6.98E-03 | 0.0183664 |
|  |  | ENSG00000134825 | 11 | TMEM258 | 61535973 | rs61896141 | 11 | 61556039 | C | A | 0.152087 | -0.206729 | 0.019876 | 2.45E-25 | 0.187231 | 0.0274773 | 9.49E-12 | -1.10414 | 0.193716 | 1.20E-08 | 0.203245414 | 16 | 2.03E-05 | 0.0018264 |
|  |  | ENSG00000149485 | 11 | FADS1 | 61567099 | rs968567 | 11 | 61595564 | T | C | 0.150099 | -0.205333 | 0.0197851 | 3.12E-25 | 0.289897 | 0.0510035 | 1.32E-08 | -0.708296 | 0.142081 | 6.19E-07 | 0.106791941 | 9 | 8.39E-04 | 0.563279 |
|  |  | ENSG00000088038 | 19 | CNOT3 | 54641444 | rs42318 | 19 | 54657069 | C | T | 0.714712 | 0.0971857 | 0.0169731 | 1.03E-08 | -0.096945 | 0.0165856 | 5.06E-09 | -1.00248 | 0.245087 | 4.31E-05 | 0.07509328 | 4 | 4.17E-02 | 0.0018366 |
| GWAS: genome-wide association study.  eQTL: expression quantitative trait loci.  SMR: summary-data-based Mendelian randomization.  HEIDI: heterogeneity in dependent instruments. Only genome-wide significant eQTLs (P<5E-8) are taken into the analysis. We report SNP-gene combinations with PSMR < genome-wide significance Benjamin Hochberg correction threshold of PFDR＜0.05, and survived after the heterogeneity test (PHEIDI＞0.05). β in GWAS association, regression coefficient of cancer on SNP, log(OR).  SE, standard error.  β in eQTL association, regression coefficient of gene expression on SNP.  β in SMR association, regression coefficient of cancer on gene expression.  PP.H4, posterior probability of H4 ,PP.H4＞0.75 as the cut-off for the evidence of colocalization of cancer GWAS and eQTL association. | | | | | | | | | | | | | | | | | | | | | | | |  |

| **Table S6. SMR and colocalization results from eQTL of diverse metabolites causally associated with intestinal disease outcomes** | | | | | | | | | | | | | | | | | | | | | | | | |
| --- | --- | --- | --- | --- | --- | --- | --- | --- | --- | --- | --- | --- | --- | --- | --- | --- | --- | --- | --- | --- | --- | --- | --- | --- |
| **Type of disease** | **Type of metabolites** | **Probe** | **Gene Chr.** | **Gene** | **Probe base pair** | **topSNP** | **Gene Chr.** | **Probe base pair** | **Effect allele** | **Other allele** | **Effect allele frequence** | **GWAS association** | | | **eQTL association** | | | **SMR association** | | | **HEIDI Test** | | **BH correction** | **PP.H4** |
|  |  |  |  |  |  |  |  |  |  |  |  | **β** | **SE** | ***P*** | **β** | **SE** | ***P*** | **β** | **SE** | ***P*** | ***P*** | **nsnp** | ***P-FDR*** |  |
| Ulcerative colitis | N-acetyl-isoputreanine levels | ENSG00000108528 | 17 | SLC25A11 | 4840425 | rs238231 | 17 | 4886772 | G | A | 0.703777 | 0.142585 | 0.0171058 | 7.72E-17 | 0.0753161 | 0.012727 | 3.26E-09 | 1.89315 | 0.392332 | 1.40E-06 | 0.2230142 | 15 | 0.004736311 | 0.8273641 |
| Inflammatory bowel disease | 1-stearoyl-2-linoleoyl-GPI levels | ENSG00000116641 | 1 | DOCK7 | 62920399 | rs1168086 | 1 | 63112389 | G | A | 0.694831 | 0.146424 | 0.0154377 | 2.43E-21 | 0.330647 | 0.0337641 | 1.21E-22 | 0.442841 | 0.0649986 | 9.55E-12 | 0.062366728 | 20 | 3.24E-08 | 0.00273618 |
|  |  | ENSG00000124920 | 11 | MYRF | 61520114 | rs198462 | 11 | 61524119 | A | G | 0.494036 | -0.0797707 | 0.0147955 | 6.98E-08 | -0.328849 | 0.0309449 | 2.23E-26 | 0.242575 | 0.0504511 | 1.52E-06 | 0.6476913 | 20 | 0.002065187 | 0.018273562 |
|  |  | ENSG00000088038 | 19 | CNOT3 | 54641444 | rs42318 | 19 | 54657069 | C | T | 0.714712 | -0.140167 | 0.0165183 | 2.15E-17 | -0.096945 | 0.0165856 | 5.06E-09 | 1.44584 | 0.300363 | 1.48E-06 | 0.0761359 | 4 | 0.002065187 | 0.34816354 |
|  |  | ENSG00000167608 | 19 | TMC4 | 54663846 | rs60204587 | 19 | 54671421 | G | A | 0.570577 | -0.199056 | 0.0151214 | 1.42E-39 | 0.782387 | 0.0409451 | 2.16E-81 | -0.254421 | 0.0234697 | 2.21E-27 | 0.09212242 | 20 | 1.50E-23 | 0.853656183 |
|  | Urea levels | ENSG00000136827 | 9 | TOR1A | 132575223 | rs7020809 | 9 | 132564706 | G | C | 0.28827 | 0.0707439 | 0.0170159 | 3.22E-05 | -0.271766 | 0.0192921 | 4.57E-45 | -0.260312 | 0.0652823 | 6.68E-05 | 0.2039576 | 20 | 0.220597021 | 0.87352735 |
| Acute gastritis | Nervonoylcarnitine levels | ENSG00000115009 | 2 | CCL20 | 228678558 | rs1811711 | 2 | 228670476 | G | C | 0.17992 | -0.0930586 | 0.0201452 | 3.85E-06 | -0.395784 | 0.0389888 | 3.27E-24 | 0.235125 | 0.0559218 | 2.62E-05 | 0.2287053 | 15 | 0.035465966 | 0.7237481 |
|  |  | ENSG00000233006 | 5 | AC034220.3 | 131646978 | rs272877 | 5 | 131674260 | G | A | 0.676938 | -0.119165 | 0.016983 | 2.27E-12 | 0.30965 | 0.045736 | 1.28E-11 | -0.384838 | 0.0789874 | 1.10E-06 | 0.2391784 | 15 | 0.001870655 | 0.08947281 |
|  |  | ENSG00000197375 | 5 | SLC22A5 | 131705444 | rs2631367 | 5 | 131705458 | G | C | 0.55666 | 0.125409 | 0.0155837 | 8.45E-16 | 0.455593 | 0.035769 | 3.68E-37 | 0.275265 | 0.0404605 | 1.02E-11 | 0.11431939 | 15 | 3.46E-08 | 0.0836452 |
| GWAS: genome-wide association study.  eQTL: expression quantitative trait loci.  SMR: summary-data-based Mendelian randomization.  HEIDI: heterogeneity in dependent instruments. Only genome-wide significant eQTLs (P<5E-8) are taken into the analysis. We report SNP-gene combinations with P_SMR_ < genome-wide significance Benjamin Hochberg correction threshold of PFDR＜0.05, and survived after the heterogeneity test (PHEIDI＞0.05). β in GWAS association, regression coefficient of cancer on SNP, log(OR).  SE, standard error.  β in eQTL association, regression coefficient of gene expression on SNP.  β in SMR association, regression coefficient of cancer on gene expression.  PP.H4, posterior probability of H4 ,PP.H4＞0.75 as the cut-off for the evidence of colocalization of cancer GWAS and eQTL association. | | | | | | | | | | | | | | | | | | | | | | | |  |

| **Table S7. SMR and colocalization results eQTL of diverse metabolites causally associated with other digestive disorders outcomes** | | | | | | | | | | | | | | | | | | | | | | | | |
| --- | --- | --- | --- | --- | --- | --- | --- | --- | --- | --- | --- | --- | --- | --- | --- | --- | --- | --- | --- | --- | --- | --- | --- | --- |
| **Type of disease** | **Type of metabolites** | **Probe** | **Gene Chr.** | **Gene** | **Probe base pair** | **topSNP** | **Gene Chr.** | **Probe base pair** | **Effect allele** | **Other allele** | **Effect allele frequence** | **GWAS association** | | | **eQTL association** | | | **SMR association** | | | **HEIDI Test** | | **BH correction** | **PP.H4** |
|  |  |  |  |  |  |  |  |  |  |  |  | **β** | **SE** | ***P*** | **β** | **SE** | ***P*** | **β** | **SE** | ***P*** | ***P*** | **nsnp** | ***P-FDR*** |  |
| Non-alcoholic liver disease | Bilirubin degradation product levels | ENSG00000077044 | 2 | DGKD | 234263153 | rs62195072 | 2 | 234260879 | C | T | 0.322068 | -0.114539 | 0.0159611 | 7.17E-13 | 0.118329 | 0.0200402 | 3.54E-09 | -0.967971 | 0.212296 | 5.13E-06 | 0.05203509 | 4 | 1.74E-02 | 0.17833634 |
|  |  | ENSG00000085982 | 2 | USP40 | 234384166 | rs62192778 | 2 | 234475415 | A | G | 0.149105 | -0.143689 | 0.0204001 | 1.87E-12 | -0.412207 | 0.0433737 | 2.03E-21 | 0.348585 | 0.0616005 | 1.52E-08 | 0.05405707 | 16 | 1.03E-04 | 0.4371529 |
|  |  | ENSG00000115216 | 2 | NRBP1 | 27650657 | rs1728911 | 2 | 27649929 | G | T | 0.412525 | -0.183072 | 0.0155696 | 6.40E-32 | -0.157802 | 0.0182461 | 5.22E-18 | 1.16014 | 0.166521 | 3.24E-12 | 0.073014402 | 20 | 2.20E-08 | 0.7864663 |
|  | Bilirubin (z,z) levels | ENSG00000077044 | 2 | DGKD | 234263153 | rs62195072 | 2 | 234260879 | C | T | 0.322068 | -0.128702 | 0.0160631 | 1.13E-15 | 0.118329 | 0.0200402 | 3.54E-09 | -1.08766 | 0.228823 | 2.00E-06 | 0.12244929 | 4 | 6.78E-03 | 0.01725381 |
|  |  | ENSG00000085982 | 2 | USP40 | 234384166 | rs62192778 | 2 | 234475415 | A | G | 0.149105 | -0.178944 | 0.0204936 | 2.51E-18 | -0.412207 | 0.0433737 | 2.03E-21 | 0.434112 | 0.0675151 | 1.28E-10 | 0.11454043 | 16 | 8.66E-07 | 0.38265513 |
|  | Biliverdin levels | ENSG00000077044 | 2 | DGKD | 234263153 | rs62195072 | 2 | 234260879 | C | T | 0.322068 | -0.133458 | 0.0155526 | 9.40E-18 | 0.118329 | 0.0200402 | 3.54E-09 | -1.12786 | 0.231865 | 1.15E-06 | 0.07765351 | 4 | 3.89E-03 | 0.18263898 |
|  |  | ENSG00000085982 | 2 | USP40 | 234384166 | rs62192778 | 2 | 234475415 | A | G | 0.149105 | -0.160271 | 0.0198567 | 6.95E-16 | -0.412207 | 0.0433737 | 2.03E-21 | 0.388812 | 0.0632005 | 7.65E-10 | 0.06622542 | 16 | 5.19E-06 | 0.79384613 |
|  | Imidazole lactate levels | ENSG00000137944 | 1 | CCBL2 | 89401456 | rs3738055 | 1 | 89426902 | A | G | 0.477137 | 0.395735 | 0.014505 | 6.83E-164 | -0.260098 | 0.0209737 | 2.58E-35 | -1.52148 | 0.134768 | 1.48E-29 | 0.23757911 | 20 | 1.00E-25 | 0.8461398 |
|  |  | ENSG00000117226 | 1 | GBP3 | 89472349 | rs12121223 | 1 | 89481583 | T | C | 0.32008 | 0.105541 | 0.0160867 | 5.35E-11 | -0.563479 | 0.0290949 | 1.47E-83 | -0.187302 | 0.0301425 | 5.17E-10 | 0.2078793 | 20 | 1.75E-06 | 0.0083633 |
|  |  | ENSG00000167136 | 9 | ENDOG | 131580753 | rs6478854 | 9 | 131588888 | C | G | 0.294235 | -0.0932472 | 0.0168253 | 2.99E-08 | -0.208876 | 0.030247 | 5.00E-12 | 0.446424 | 0.103284 | 1.54E-05 | 0.4858293 | 7 | 1.34E-02 | 0.7974713 |
|  |  | ENSG00000198917 | 9 | C9orf114 | 131581930 | rs6478854 | 9 | 131588888 | C | G | 0.294235 | -0.0932472 | 0.0168253 | 2.99E-08 | -0.132096 | 0.0170547 | 9.53E-15 | 0.705905 | 0.15662 | 6.57E-06 | 0.5013276 | 12 | 8.91E-03 | 0.00846713 |
|  |  | ENSG00000110768 | 11 | GTF2H1 | 18343842 | rs11024614 | 11 | 18326758 | T | C | 0.583499 | 0.0912617 | 0.0151148 | 1.56E-09 | 0.217218 | 0.0239313 | 1.12E-19 | 0.420139 | 0.0835727 | 4.98E-07 | 0.7071216 | 12 | 8.43E-04 | 0.173538 |
|  |  | ENSG00000169710 | 17 | FASN | 80036214 | rs59638227 | 17 | 80056066 | G | A | 0.285288 | 0.0841659 | 0.0162123 | 2.09E-07 | -0.161735 | 0.0214773 | 5.05E-14 | -0.520394 | 0.121752 | 1.92E-05 | 0.09355298 | 20 | 1.44E-02 | 0.77837651 |
|  | Mannose to glycerol ratio | ENSG00000115216 | 2 | NRBP1 | 27650657 | rs1728911 | 2 | 27649929 | G | T | 0.412525 | 0.0935141 | 0.0136388 | 7.06E-12 | -0.157802 | 0.0182461 | 5.22E-18 | -0.592604 | 0.110296 | 7.75E-08 | 0.106194601 | 20 | 5.25E-04 | 0.0182638 |
| Acute pancreatitis | (R)-3-hydroxybutyrylcarnitine levels | ENSG00000197375 | 5 | SLC22A5 | 131705444 | rs2631367 | 5 | 131705458 | G | C | 0.55666 | 0.0753876 | 0.0157293 | 1.64E-06 | 0.455593 | 0.035769 | 3.68E-37 | 0.165471 | 0.0368882 | 7.27E-06 | 0.1987088 | 20 | 4.93E-02 | 0.0384983 |
| Chronic pancreatitis | 1-palmitoyl-2-dihomo-linolenoyl-GPC levels | ENSG00000116641 | 1 | DOCK7 | 62920399 | rs1168086 | 1 | 63112389 | G | A | 0.694831 | 0.071811 | 0.0157466 | 5.11E-06 | 0.330647 | 0.0337641 | 1.21E-22 | 0.217183 | 0.0525344 | 3.56E-05 | 0.06835431 | 20 | 2.68E-02 | 0.78376163 |
|  |  | ENSG00000124920 | 11 | MYRF | 61520114 | rs198462 | 11 | 61524119 | A | G | 0.494036 | -0.114756 | 0.0150366 | 2.32E-14 | -0.328849 | 0.0309449 | 2.23E-26 | 0.348963 | 0.0562946 | 5.69E-10 | 0.1386488 | 20 | 7.71E-07 | 0.008366198 |
|  |  | ENSG00000134825 | 11 | TMEM258 | 61535973 | rs61896141 | 11 | 61556039 | C | A | 0.152087 | 0.381974 | 0.0193544 | 1.06E-86 | 0.187231 | 0.0274773 | 9.49E-12 | 2.04012 | 0.316743 | 1.19E-10 | 0.1151748 | 16 | 2.01E-07 | 0.29572632 |
|  |  | ENSG00000149485 | 11 | FADS1 | 61567099 | rs968567 | 11 | 61595564 | T | C | 0.150099 | 0.384062 | 0.0192691 | 2.17E-88 | 0.289897 | 0.0510035 | 1.32E-08 | 1.32482 | 0.242377 | 4.60E-08 | 0.1317342 | 9 | 4.46E-05 | 0.76354183 |
|  |  | ENSG00000157045 | 16 | NTAN1 | 15131710 | rs11644601 | 16 | 15172118 | C | T | 0.290258 | 0.161339 | 0.0165484 | 1.85E-22 | -0.20327 | 0.0276247 | 1.86E-13 | -0.793718 | 0.135141 | 4.27E-09 | 0.07948852 | 10 | 4.83E-06 | 0.01826379 |
| GWAS: genome-wide association study.  eQTL: expression quantitative trait loci.  SMR: summary-data-based Mendelian randomization.  HEIDI: heterogeneity in dependent instruments. Only genome-wide significant eQTLs (P<5E-8) are taken into the analysis. We report SNP-gene combinations with P_SMR_ < genome-wide significance Benjamin Hochberg correction threshold of PFDR＜0.05, and survived after the heterogeneity test (PHEIDI＞0.05). β in GWAS association, regression coefficient of cancer on SNP, log(OR).  SE, standard error.  β in eQTL association, regression coefficient of gene expression on SNP.  β in SMR association, regression coefficient of cancer on gene expression.  PP.H4, posterior probability of H4 ,PP.H4＞0.75 as the cut-off for the evidence of colocalization of cancer GWAS and eQTL association. | | | | | | | | | | | | | | | | | | | | | | | | |

| **Table S8. SMR and colocalization results from mQTL of diverse metabolites causally associated with cancer outcomes** | | | | | | | | | | | | | | | | | | | | | | | | | | |
| --- | --- | --- | --- | --- | --- | --- | --- | --- | --- | --- | --- | --- | --- | --- | --- | --- | --- | --- | --- | --- | --- | --- | --- | --- | --- | --- |
| **Type of disease** | **Type of metabolites** | **probeID** | **Probe Chr** | **Gene** | **Probe base pair** | **topSNP** | **SNP Chr** | **SNP base pair** | **Effect allele** | **Other allele** | **Effect allele frequence** | **GWAS association** | | | **mQTL association** | | | **SMR association** | | | **HEIDI Test** | | **BH correction** | **PP.H4** | | |
|  |  |  |  |  |  |  |  |  |  |  |  | **β** | **SE** | ***P*** | **β** | **SE** | ***P*** | **β** | **SE** | ***P*** | ***P*** | **nsnp** | ***P-FDR*** |  | | |
| colon cancer | 1,2-dipalmitoyl-gpc levels | cg27594095 | 6 | PSORS1C1 | 31095276 | rs3095300 | 6 | 31082476 | T | C | 0.181909 | 0.101019 | 0.0181949 | 2.82E-08 | 0.586954 | 0.0347521 | 5.35E-64 | 0.172107 | 0.0326308 | 1.33E-07 | 0.9568338 | 20 | 0.000312676 | 0.1746283 | | |
|  |  | cg14646094 | 6 | MCCD1 | 31496752 | rs2855811 | 6 | 31471801 | A | G | 0.262425 | -0.0788526 | 0.0168868 | 3.02E-06 | -0.540227 | 0.0347079 | 1.26E-54 | 0.145962 | 0.0326351 | 7.73E-06 | 0.65994 | 20 | 0.00663362 | 0.02837632 | | |
|  |  | cg04755523 | 6 | BAT1 | 31508665 | rs2239707 | 6 | 31525319 | C | T | 0.289264 | -0.0836564 | 0.0164346 | 3.58E-07 | 0.248143 | 0.0341531 | 3.71E-13 | -0.33713 | 0.0808671 | 3.06E-05 | 0.8975091 | 20 | 0.019166207 | 0.283748371 | | |
|  |  | cg03031988 | 6 |  | 31510729 | rs2523504 | 6 | 31510858 | T | C | 0.274354 | -0.0753784 | 0.0173907 | 1.46E-05 | -0.5995 | 0.035352 | 1.68E-64 | 0.125735 | 0.0299412 | 2.68E-05 | 0.8444807 | 20 | 0.017226274 | 0.28371736 | | |
|  |  | cg16175906 | 6 | BAT3 | 31607374 | rs142831262 | 6 | 31576714 | A | G | 0.157058 | -0.102941 | 0.0223158 | 3.97E-06 | 0.384257 | 0.0478036 | 9.11E-16 | -0.267896 | 0.0669587 | 6.31E-05 | 0.711026 | 10 | 0.031275812 | 0.383917732 | | |
|  |  | cg06884718 | 6 | NOTCH4 | 32191821 | rs3891175 | 6 | 32634467 | T | C | 0.170974 | 0.094005 | 0.0180172 | 1.81E-07 | -0.306379 | 0.0376663 | 4.15E-16 | -0.306826 | 0.0698652 | 1.12E-05 | 0.9053789 | 20 | 0.00896967 | 0.83746134 | | |
|  |  | cg27064867 | 6 | C6orf10 | 32314182 | rs9271421 | 6 | 32587859 | T | C | 0.12326 | -0.115825 | 0.0222508 | 1.94E-07 | 0.646574 | 0.0430498 | 5.50E-51 | -0.179136 | 0.0364217 | 8.73E-07 | 0.5951568 | 20 | 0.001139103 | 0.018364923 | | |
|  |  | cg23214071 | 6 | HLA-DQB1 | 32627784 | rs9274623 | 6 | 32635998 | T | G | 0.202783 | 0.0942011 | 0.0180678 | 1.85E-07 | -1.04398 | 0.0308717 | 1.12E-250 | -0.0902327 | 0.0175111 | 2.57E-07 | 0.6461582 | 20 | 0.000475559 | 0.914736823 | | |
|  |  | cg10180404 | 6 |  | 32632334 | rs9274623 | 6 | 32635998 | T | G | 0.202783 | 0.0942011 | 0.0180678 | 1.85E-07 | -1.06859 | 0.030453 | 9.64E-270 | -0.0881546 | 0.0170937 | 2.51E-07 | 0.9804801 | 20 | 0.000474338 | 0.914972654 | | |
|  |  | cg22984282 | 6 |  | 32632568 | rs9274662 | 6 | 32636494 | T | C | 0.356859 | -0.0883515 | 0.0159925 | 3.30E-08 | -0.965099 | 0.0268547 | 7.85E-283 | 0.0915466 | 0.0167655 | 4.75E-08 | 0.5303193 | 20 | 0.000151829 | 0.914628836 | | |
|  |  | cg06255955 | 6 |  | 32632639 | rs9274623 | 6 | 32635998 | T | G | 0.202783 | 0.0942011 | 0.0180678 | 1.85E-07 | 0.804037 | 0.0331923 | 1.26E-129 | 0.11716 | 0.022986 | 3.45E-07 | 0.7088629 | 20 | 0.000567946 | 0.914572837 | | |
|  |  | cg03796735 | 6 |  | 32632643 | rs9274623 | 6 | 32635998 | T | G | 0.202783 | 0.0942011 | 0.0180678 | 1.85E-07 | 0.820436 | 0.0335811 | 7.93E-132 | 0.114818 | 0.0225181 | 3.42E-07 | 0.9848525 | 20 | 0.000567946 | 0.914579174 | | |
|  |  | cg23336481 | 6 |  | 32634344 | rs9274623 | 6 | 32635998 | T | G | 0.202783 | 0.0942011 | 0.0180678 | 1.85E-07 | 0.863779 | 0.0324933 | 1.06E-155 | 0.109057 | 0.0213157 | 3.12E-07 | 0.8107372 | 20 | 0.000538937 | 0.914572837 | | |
|  |  | cg05724777 | 6 |  | 32634362 | rs3828789 | 6 | 32635754 | G | T | 0.409543 | -0.0847367 | 0.0155465 | 5.02E-08 | -0.703593 | 0.0296202 | 1.00E-124 | 0.120434 | 0.0226701 | 1.08E-07 | 0.5564536 | 20 | 0.000286414 | 0.914573917 | | |
|  |  | cg04322111 | 6 | HLA-DQB2 | 32729764 | rs3763354 | 6 | 32786917 | G | A | 0.198807 | -0.0891981 | 0.0184682 | 1.37E-06 | 0.332842 | 0.0382004 | 2.96E-18 | -0.267989 | 0.0634409 | 2.40E-05 | 0.66425 | 20 | 0.016004708 | 0.0283626 | | |
|  |  | cg24298476 | 6 |  | 32729808 | rs1044043 | 6 | 32793981 | A | C | 0.195825 | -0.0933863 | 0.0184074 | 3.91E-07 | 0.247647 | 0.0377925 | 5.65E-11 | -0.377094 | 0.0940026 | 6.03E-05 | 0.8575173 | 20 | 0.030225689 | 0.000183832 | | |
|  |  | cg03735013 | 11 | FADS1 | 61582769 | rs174561 | 11 | 61582708 | C | T | 0.303181 | -0.221968 | 0.0164647 | 2.01E-41 | -0.279615 | 0.0334227 | 5.96E-17 | 0.793834 | 0.111673 | 1.17E-12 | 0.7056825 | 20 | 1.3589E-08 | 0.927351836 | | |
|  |  | cg24803730 | 19 | ANGPTL4 | 8429439 | rs4044436 | 19 | 8448034 | T | C | 0.460239 | -0.0640336 | 0.0154434 | 3.38E-05 | 0.33876 | 0.0328272 | 5.75E-25 | -0.189024 | 0.0491303 | 0.000119382 | 0.7729639 | 20 | 0.046690916 | 0.008373513 | | |
|  | Adrenate levels | cg03735013 | 11 | FADS1 | 61582769 | rs174561 | 11 | 61582708 | C | T | 0.303181 | -0.179651 | 0.0164546 | 9.46E-28 | -0.279615 | 0.0334227 | 5.96E-17 | 0.642494 | 0.0967519 | 3.12E-11 | 0.3899308 | 20 | 2.89512E-07 | 0.883374823 | | |
|  |  | cg25326896 | 11 |  | 61582785 | rs174561 | 11 | 61582708 | C | T | 0.303181 | -0.179651 | 0.0164546 | 9.46E-28 | -0.346722 | 0.0336723 | 7.27E-25 | 0.518141 | 0.0691687 | 6.84E-14 | 0.1270866 | 20 | 1.05611E-09 | 0.88471374 | | |
|  |  | cg24870774 | 11 |  | 61583342 | rs174559 | 11 | 61581656 | A | G | 0.267396 | -0.175684 | 0.0169394 | 3.35E-25 | -0.198633 | 0.034376 | 7.55E-09 | 0.884465 | 0.175221 | 4.47E-07 | 0.1250971 | 19 | 0.001973626 | 0.883628368 | | |
|  | 1,2-dilinoleoyl-GPC levels | cg03735013 | 11 | FADS1 | 61582769 | rs174561 | 11 | 61582708 | C | T | 0.303181 | 0.269376 | 0.0161453 | 1.70E-62 | -0.279615 | 0.0334227 | 5.96E-17 | -0.963382 | 0.12882 | 7.52E-14 | 0.06654498 | 20 | 4.35394E-10 | 0.9236113 | |  |
|  |  | cg16576620 | 11 | FADS2 | 61596068 | rs174534 | 11 | 61549458 | G | A | 0.346918 | 0.242127 | 0.0157164 | 1.49E-53 | -0.189375 | 0.0331083 | 1.07E-08 | -1.27856 | 0.238439 | 8.22E-08 | 0.05469476 | 14 | 0.000331315 | 0.194823 | |  |
|  | 1-oleoyl-2-linoleoyl-GPE levels | cg01825921 | 11 | C11orf9 | 61525711 | rs198460 | 11 | 61524974 | A | G | 0.497018 | -0.15153 | 0.015331 | 4.89E-23 | -0.180962 | 0.031494 | 9.14E-09 | 0.837358 | 0.168567 | 6.78E-07 | 0.7591244 | 3 | 0.002328196 | 0.29375323 | |  |
|  |  | cg04074454 | 11 |  | 61543928 | rs174529 | 11 | 61543961 | C | T | 0.383698 | 0.368459 | 0.0157492 | 4.76E-121 | -0.23121 | 0.0326113 | 1.34E-12 | -1.59361 | 0.234867 | 1.16E-11 | 0.06148275 | 20 | 5.11822E-08 | 0.29348493 | |  |
|  |  | cg03735013 | 11 | FADS1 | 61582769 | rs174561 | 11 | 61582708 | C | T | 0.303181 | 0.378128 | 0.0162652 | 1.50E-119 | -0.279615 | 0.0334227 | 5.96E-17 | -1.35232 | 0.171792 | 3.49E-15 | 0.1337355 | 20 | 1.90555E-11 | 0.85628361 | |  |
|  |  | cg25326896 | 11 |  | 61582785 | rs174561 | 11 | 61582708 | C | T | 0.303181 | 0.378128 | 0.0162652 | 1.50E-119 | -0.346722 | 0.0336723 | 7.27E-25 | -1.09058 | 0.115837 | 4.74E-21 | 0.1004837 | 20 | 3.13866E-17 | 0.85693723 | |  |
|  |  | cg12517394 | 11 |  | 61582795 | rs174592 | 11 | 61618608 | G | A | 0.38668 | 0.369233 | 0.0155709 | 2.64E-124 | -0.274167 | 0.0318942 | 8.24E-18 | -1.34674 | 0.166645 | 6.40E-16 | 0.174033 | 20 | 3.70568E-12 | 0.85628361 | |  |
|  |  | cg24870774 | 11 |  | 61583342 | rs174559 | 11 | 61581656 | A | G | 0.267396 | 0.364644 | 0.0168047 | 2.10E-104 | -0.198633 | 0.034376 | 7.55E-09 | -1.83577 | 0.328774 | 2.35E-08 | 0.100204 | 19 | 9.49046E-05 | 0.85628361 | |  |
|  |  | cg16213375 | 11 |  | 61584727 | rs174560 | 11 | 61581764 | C | T | 0.313121 | 0.375233 | 0.016208 | 1.42E-118 | -0.434186 | 0.033468 | 1.74E-38 | -0.864222 | 0.0763623 | 1.08E-29 | 0.61402243 | 20 | 1.10851E-25 | 0.85628361 | |  |
|  |  | cg10515671 | 11 |  | 61585899 | rs174533 | 11 | 61549025 | A | G | 0.347913 | 0.392637 | 0.0158222 | 6.08E-136 | 0.260664 | 0.0334249 | 6.26E-15 | 1.5063 | 0.202465 | 1.01E-13 | 0.12170076 | 20 | 5.19353E-10 | 0.85628361 | |  |
|  |  | cg16576620 | 11 | FADS2 | 61596068 | rs174534 | 11 | 61549458 | G | A | 0.346918 | 0.342176 | 0.015858 | 2.92E-103 | -0.189375 | 0.0331083 | 1.07E-08 | -1.80687 | 0.326805 | 3.22E-08 | 0.1082528 | 14 | 0.000124456 | 0.01283642 | |  |
|  |  | cg19610905 | 11 |  | 61596333 | rs174566 | 11 | 61592362 | G | A | 0.355865 | 0.389084 | 0.0156882 | 8.75E-136 | -0.851216 | 0.0301912 | 6.89E-175 | -0.457092 | 0.0245462 | 2.14E-77 | 0.1530061 | 20 | 1.98616E-72 | 0.0128846 | |  |
|  | Arachidonate (20:4n6) to oleate to vaccenate ratio | cg01825921 | 11 | C11orf9 | 61525711 | rs198460 | 11 | 61524974 | A | G | 0.497018 | 0.147316 | 0.0153265 | 7.13E-22 | -0.180962 | 0.031494 | 9.14E-09 | -0.814071 | 0.165063 | 8.14E-07 | 0.3521875 | 3 | 0.00269607 | 0.00746283 | |  |
|  |  | cg03735013 | 11 | FADS1 | 61582769 | rs174561 | 11 | 61582708 | C | T | 0.303181 | -0.442123 | 0.0160174 | 1.03E-167 | -0.279615 | 0.0334227 | 5.96E-17 | 1.58118 | 0.197491 | 1.18E-15 | 0.2742567 | 20 | 6.44185E-12 | 0.79273561 | |  |
|  |  | cg16576620 | 11 | FADS2 | 61596068 | rs174534 | 11 | 61549458 | G | A | 0.346918 | -0.359527 | 0.015776 | 5.82E-115 | -0.189375 | 0.0331083 | 1.07E-08 | 1.89849 | 0.342207 | 2.89E-08 | 0.04475789 | 14 | 0.000107266 | 0.02837441 | |  |
|  | Arachidonate to linoleate ratio | cg01825921 | 11 | C11orf9 | 61525711 | rs198460 | 11 | 61524974 | A | G | 0.497018 | 0.123189 | 0.0152271 | 5.96E-16 | -0.180962 | 0.031494 | 9.14E-09 | -0.680745 | 0.145316 | 2.81E-06 | 0.2314106 | 3 | 0.009017215 | 0.08238771 | |  |
|  |  | cg03735013 | 11 | FADS1 | 61582769 | rs174561 | 11 | 61582708 | C | T | 0.303181 | -0.409931 | 0.0159741 | 3.09E-145 | -0.279615 | 0.0334227 | 5.96E-17 | 1.46606 | 0.184316 | 1.81E-15 | 0.1320621 | 20 | 9.84422E-12 | 0.6946381 | |  |
|  |  | cg16576620 | 11 | FADS2 | 61596068 | rs174534 | 11 | 61549458 | G | A | 0.346918 | -0.340471 | 0.0156872 | 1.89E-104 | -0.189375 | 0.0331083 | 1.07E-08 | 1.79787 | 0.325052 | 3.18E-08 | 0.24231111 | 14 | 0.000118053 | 0.38619442 | |  |
|  | 1-palmitoyl-2-arachidonoyl-gpc levels | cg03735013 | 11 | FADS1 | 61582769 | rs174561 | 11 | 61582708 | C | T | 0.303181 | -0.615629 | 0.0154337 | 1.00E-200 | -0.279615 | 0.0334227 | 5.96E-17 | 2.2017 | 0.268898 | 2.66E-16 | 0.4553861 | 20 | 1.23217E-12 | 0.8038371 | |  |
|  |  | cg24870774 | 11 |  | 61583342 | rs174559 | 11 | 61581656 | A | G | 0.267396 | -0.582897 | 0.0160913 | 1.00E-200 | -0.198633 | 0.034376 | 7.55E-09 | 2.93454 | 0.51428 | 1.16E-08 | 0.0927091 | 19 | 3.82629E-05 | 0.8038993 | |  |
|  |  | cg01772980 | 11 | SCGB1D1 | 61957695 | rs10897232 | 11 | 61962057 | C | T | 0.272366 | -0.0971119 | 0.017005 | 1.12E-08 | 0.608013 | 0.0358021 | 1.10E-64 | -0.15972 | 0.0295071 | 6.20E-08 | 0.4406499 | 20 | 0.000169026 | 0.0038361 | |  |
|  | Uridine to pseudouridine ratio | cg04087271 | 1 | CDA | 20915334 | rs603412 | 1 | 20915418 | G | C | 0.408549 | -0.10505 | 0.0147653 | 1.12E-12 | -0.767321 | 0.0298677 | 1.49E-145 | 0.136905 | 0.0199669 | 7.05E-12 | 0.7142293 | 20 | 6.53724E-07 | 0.000483613 | |  |
|  |  | cg02006203 | 7 | UPP1 | 48128151 | rs2708870 | 7 | 48111833 | T | C | 0.452286 | 0.092645 | 0.0145996 | 2.21E-10 | -0.92258 | 0.0250282 | 1.91E-297 | -0.100419 | 0.0160575 | 4.01E-10 | 0.2349119 | 20 | 1.85736E-05 | 0.73937997 | |  |
|  |  | cg02399030 | 7 |  | 48128821 | rs3763505 | 7 | 48128850 | G | A | 0.4334 | 0.0970116 | 0.0146541 | 3.59E-11 | 0.428957 | 0.0319319 | 3.85E-41 | 0.226157 | 0.0380852 | 2.88E-09 | 0.2545631 | 20 | 5.34261E-05 | 0.739355273 | |  |
|  |  | cg20001070 | 7 |  | 48131586 | rs13227958 | 7 | 48131580 | T | C | 0.45328 | 0.0908698 | 0.0145963 | 4.80E-10 | 0.709649 | 0.0246249 | 1.26E-182 | 0.128049 | 0.0210428 | 1.16E-09 | 0.1008254 | 20 | 2.69666E-05 | 0.739309374 | |  |
|  | Phosphate to linoleoyl-arachidonoyl-glycerol ratio | cg15676405 | 11 | C11orf9 | 61525677 | rs198465 | 11 | 61521318 | A | G | 0.489066 | -0.0828447 | 0.0159413 | 2.03E-07 | -0.324263 | 0.0318393 | 2.33E-24 | 0.255486 | 0.0551922 | 3.67E-06 | 0.06368789 | 20 | 0.014805702 | 0.000048363 | |  |
|  |  | cg07689907 | 11 | FADS1 | 61582574 | rs174561 | 11 | 61582708 | C | T | 0.303181 | 0.300599 | 0.0172455 | 4.83E-68 | -0.701605 | 0.0330689 | 6.73E-100 | -0.428445 | 0.0318116 | 2.41E-41 | 0.08726158 | 20 | 7.43471E-37 | 0.69283957 | |  |
|  |  | cg03735013 | 11 |  | 61582769 | rs174561 | 11 | 61582708 | C | T | 0.303181 | 0.300599 | 0.0172455 | 4.83E-68 | -0.279615 | 0.0334227 | 5.96E-17 | -1.07505 | 0.142536 | 4.62E-14 | 0.8374682 | 20 | 2.62817E-10 | 0.692834848 | |  |
|  |  | cg16213375 | 11 |  | 61584727 | rs174560 | 11 | 61581764 | C | T | 0.313121 | 0.301584 | 0.0171683 | 4.46E-69 | -0.434186 | 0.033468 | 1.74E-38 | -0.694596 | 0.0665594 | 1.70E-25 | 0.082895566 | 20 | 3.15683E-21 | 0.692838375 | |  |
|  |  | cg10515671 | 11 |  | 61585899 | rs174533 | 11 | 61549025 | A | G | 0.347913 | 0.290071 | 0.016836 | 1.60E-66 | 0.260664 | 0.0334249 | 6.26E-15 | 1.11282 | 0.156633 | 1.21E-12 | 0.060534053 | 20 | 6.2137E-09 | 0.692835624 | |  |
|  | Docosapentaenoate levels | cg07689907 | 11 | FADS1 | 61582574 | rs174561 | 11 | 61582708 | C | T | 0.303181 | -0.142048 | 0.0159861 | 6.35E-19 | -0.701605 | 0.0330689 | 6.73E-100 | 0.202461 | 0.0247026 | 2.49E-16 | 0.06724892 | 20 | 7.68198E-12 | 0.864673445 | |  |
|  |  | cg14725641 | 11 |  | 61582763 | rs174561 | 11 | 61582708 | C | T | 0.303181 | -0.142048 | 0.0159861 | 6.35E-19 | -0.45784 | 0.0334317 | 1.09E-42 | 0.310257 | 0.0416222 | 9.05E-14 | 0.2113619 | 20 | 2.09637E-09 | 0.864653458 | |  |
|  |  | cg03735013 | 11 |  | 61582769 | rs174561 | 11 | 61582708 | C | T | 0.303181 | -0.142048 | 0.0159861 | 6.35E-19 | -0.279615 | 0.0334227 | 5.96E-17 | 0.508013 | 0.0834022 | 1.12E-09 | 0.4729157 | 20 | 1.03931E-05 | 0.86465363 | |  |
|  |  | cg25326896 | 11 |  | 61582785 | rs174561 | 11 | 61582708 | C | T | 0.303181 | -0.142048 | 0.0159861 | 6.35E-19 | -0.346722 | 0.0336723 | 7.27E-25 | 0.409688 | 0.0609002 | 1.73E-11 | 0.4084484 | 20 | 2.67174E-07 | 0.86467345 | |  |
|  |  | cg12517394 | 11 |  | 61582795 | rs174592 | 11 | 61618608 | G | A | 0.38668 | -0.133802 | 0.0153288 | 2.57E-18 | -0.274167 | 0.0318942 | 8.24E-18 | 0.488031 | 0.0796818 | 9.08E-10 | 0.233362 | 20 | 9.35432E-06 | 0.8646284 | |  |
|  |  | cg16213375 | 11 |  | 61584727 | rs174560 | 11 | 61581764 | C | T | 0.313121 | -0.14129 | 0.0159257 | 7.19E-19 | -0.434186 | 0.033468 | 1.74E-38 | 0.325414 | 0.0444361 | 2.42E-13 | 0.2760181 | 20 | 4.48934E-09 | 0.86463572 | |  |
|  |  | cg10515671 | 11 |  | 61585899 | rs174533 | 11 | 61549025 | A | G | 0.347913 | -0.139346 | 0.0156185 | 4.58E-19 | 0.260664 | 0.0334249 | 6.26E-15 | -0.534581 | 0.0910449 | 4.32E-09 | 0.07656923 | 20 | 3.33354E-05 | 0.86468345 | |  |
|  |  | cg07005513 | 11 | FADS2 | 61595956 | rs174559 | 11 | 61581656 | A | G | 0.267396 | -0.143823 | 0.0164472 | 2.24E-18 | -0.326923 | 0.034982 | 9.15E-21 | 0.439929 | 0.0688983 | 1.71E-10 | 0.2526641 | 20 | 2.26664E-06 | 0.73846534 | |  |
|  |  | cg19610905 | 11 |  | 61596333 | rs174566 | 11 | 61592362 | G | A | 0.355865 | -0.136402 | 0.0154875 | 1.28E-18 | -0.851216 | 0.0301912 | 6.89E-175 | 0.160244 | 0.0190616 | 4.22E-17 | 0.040177 | 20 | 3.9116E-12 | 0.73849764 | |  |
|  |  | cg11250194 | 11 |  | 61601937 | rs174559 | 11 | 61581656 | A | G | 0.267396 | -0.143823 | 0.0164472 | 2.24E-18 | -0.80871 | 0.0324609 | 5.34E-137 | 0.177842 | 0.021554 | 1.57E-16 | 0.0684073 | 20 | 7.27826E-12 | 0.73847735 | |  |
|  |  | cg07591205 | 11 |  | 61633557 | rs174544 | 11 | 61567753 | A | C | 0.304175 | -0.143179 | 0.0159985 | 3.57E-19 | 0.24516 | 0.0335969 | 2.94E-13 | -0.584023 | 0.103267 | 1.55E-08 | 0.1641548 | 19 | 9.00479E-05 | 0.73849435 | |  |
| Esophageal cancer | X-23641 levels | cg24594295 | 1 | S100A10 | 151966792 | rs2999508 | 1 | 151934447 | A | C | 0.392644 | 0.138495 | 0.0164834 | 4.39E-17 | 0.276268 | 0.0310038 | 5.07E-19 | 0.501307 | 0.0820052 | 9.77E-10 | 0.3147793 | 20 | 1.1321E-05 | 0.90383561 | |  |
|  |  | cg25848158 | 1 |  | 151966808 | rs7523082 | 1 | 151848126 | A | T | 0.39662 | 0.152765 | 0.0165041 | 2.12E-20 | 0.43946 | 0.0318267 | 2.28E-43 | 0.34762 | 0.0452129 | 1.49E-14 | 0.4829761 | 20 | 1.37983E-09 | 0.90386248 | |  |
|  |  | cg13249591 | 1 |  | 151966892 | rs11582739 | 1 | 151934541 | A | C | 0.285288 | 0.150049 | 0.0176157 | 1.62E-17 | 0.508472 | 0.0330505 | 2.07E-53 | 0.295098 | 0.0395999 | 9.19E-14 | 0.1028858 | 20 | 4.26139E-09 | 0.90389743 | |  |
|  |  | cg13445177 | 1 |  | 151967021 | rs1602251 | 1 | 151903671 | A | G | 0.352883 | 0.141674 | 0.0168616 | 4.38E-17 | 0.371557 | 0.0324462 | 2.31E-30 | 0.381298 | 0.056286 | 1.25E-11 | 0.09552032 | 20 | 1.93116E-07 | 0.903888345 | |  |
|  |  | cg20167074 | 1 |  | 151967023 | rs6662602 | 1 | 151915155 | T | C | 0.276342 | 0.162881 | 0.0180319 | 1.67E-19 | 0.424892 | 0.0342143 | 2.07E-35 | 0.383347 | 0.052478 | 2.77E-13 | 0.2333393 | 20 | 6.4292E-09 | 0.903889452 | |  |
|  |  | cg18348690 | 1 |  | 151967697 | rs1038747 | 1 | 151942345 | G | T | 0.39165 | 0.139472 | 0.0164884 | 2.70E-17 | -0.31423 | 0.0319028 | 6.88E-23 | -0.443853 | 0.0691667 | 1.39E-10 | 0.1122513 | 20 | 1.83851E-06 | 0.903889346 |  |  |
|  |  | cg03643559 | 10 | IKZF5 | 124757121 | rs61863178 | 10 | 124762694 | T | C | 0.390656 | -0.123873 | 0.0167571 | 1.44E-13 | -1.21911 | 0.0205046 | 0 | 0.101609 | 0.0138512 | 2.20E-13 | 0.0553639 | 20 | 6.4292E-09 | 0.00384613 |  |  |
| Pancreatic cancer | 1-stearoyl-2-arachidonoyl-GPI levels | cg08107208 | 1 | USP1 | 62901199 | rs111337204 | 1 | 63146249 | A | C | 0.303181 | -0.189396 | 0.0157852 | 3.62E-33 | 0.261304 | 0.0332497 | 3.88E-15 | -0.724811 | 0.110252 | 4.89E-11 | 0.05418904 | 8 | 1.97206E-07 | 0.00386135 |  |  |
|  |  | cg14087351 | 7 | MLXIPL | 73037990 | rs3812316 | 7 | 73020337 | G | C | 0.116302 | -0.136926 | 0.023095 | 3.05E-09 | -0.939377 | 0.0436364 | 8.63E-103 | 0.145763 | 0.0255008 | 1.09E-08 | 0.6595161 | 20 | 3.61039E-05 | 0.000167384 |  |  |
|  |  | cg07689907 | 11 | FADS1 | 61582574 | rs174561 | 11 | 61582708 | C | T | 0.303181 | -0.259442 | 0.0163149 | 6.13E-57 | -0.701605 | 0.0330689 | 6.73E-100 | 0.369784 | 0.0290604 | 4.31E-37 | 0.2543625 | 20 | 1.33274E-32 | 0.01836513 |  |  |
|  |  | cg14725641 | 11 |  | 61582763 | rs174561 | 11 | 61582708 | C | T | 0.303181 | -0.259442 | 0.0163149 | 6.13E-57 | -0.45784 | 0.0334317 | 1.09E-42 | 0.566665 | 0.0546074 | 3.15E-25 | 0.08241166 | 20 | 7.30618E-21 | 0.018365864 |  |  |
|  |  | cg25326896 | 11 | FADS3 | 61582785 | rs174561 | 11 | 61582708 | C | T | 0.303181 | -0.259442 | 0.0163149 | 6.13E-57 | -0.346722 | 0.0336723 | 7.27E-25 | 0.748271 | 0.0865734 | 5.47E-18 | 0.1628714 | 20 | 3.89733E-14 | 0.002387663 |  |  |
|  |  | cg12517394 | 11 | FADS4 | 61582795 | rs174592 | 11 | 61618608 | G | A | 0.38668 | -0.232798 | 0.0156723 | 6.54E-50 | -0.274167 | 0.0318942 | 8.24E-18 | 0.84911 | 0.114126 | 1.01E-13 | 0.1208321 | 20 | 5.48552E-10 | 0.000047153 |  |  |
|  |  | cg24870774 | 11 | FADS5 | 61583342 | rs174559 | 11 | 61581656 | A | G | 0.267396 | -0.255 | 0.0168198 | 6.44E-52 | -0.198633 | 0.034376 | 7.55E-09 | 1.28377 | 0.237763 | 6.69E-08 | 0.1908739 | 19 | 0.000206613 | 0.3047613 |  |  |
|  |  | cg07005513 | 11 | FADS2 | 61595956 | rs174559 | 11 | 61581656 | A | G | 0.267396 | -0.255 | 0.0168198 | 6.44E-52 | -0.326923 | 0.034982 | 9.15E-21 | 0.78 | 0.0980461 | 1.78E-15 | 0.06491871 | 20 | 1.03406E-11 | 0.853735 |  |  |
|  |  | cg19610905 | 11 |  | 61596333 | rs174566 | 11 | 61592362 | G | A | 0.355865 | -0.244323 | 0.0158165 | 7.86E-54 | -0.851216 | 0.0301912 | 6.89E-175 | 0.287028 | 0.0211872 | 8.22E-42 | 0.08335429 | 20 | 7.62357E-37 | 0.85368634 |  |  |
|  |  | cg07591205 | 11 |  | 61633557 | rs174544 | 11 | 61567753 | A | C | 0.304175 | -0.262161 | 0.0163243 | 4.90E-58 | 0.24516 | 0.0335969 | 2.94E-13 | -1.06935 | 0.160962 | 3.06E-11 | 0.07606116 | 19 | 1.291E-07 | 0.853664862 |  |  |
| GWAS: genome-wide association study.  mQTL: methylation quantitative trait loci.  SMR: summary-data-based Mendelian randomization.  HEIDI: heterogeneity in dependent instruments. Only genome-wide significant mQTLs (P<5E-8) are taken into the analysis. We report SNP-gene combinations with P_SMR_ < genome-wide significance Benjamin Hochberg correction threshold of PFDR＜0.05, and survived after the heterogeneity test (PHEIDI＞0.05). β in GWAS association, regression coefficient of cancer on SNP, log(OR).  SE, standard error.  β in eQTL association, regression coefficient of gene expression on SNP.  β in SMR association, regression coefficient of cancer on gene expression.  PP.H4, posterior probability of H4 ,PP.H4＞0.75 as the cut-off for the evidence of colocalization of cancer GWAS and eQTL association. | | | | | | | | | | | | | | | | | | | | | | | | |  |  |

| **Table S9. SMR and colocalization results mQTL of diverse metabolites causally associated with intestinal disease outcomes** | | | | | | | | | | | | | | | | | | | | | | | | |
| --- | --- | --- | --- | --- | --- | --- | --- | --- | --- | --- | --- | --- | --- | --- | --- | --- | --- | --- | --- | --- | --- | --- | --- | --- |
| **Type of disease** | **Type of metabolites** | **probeID** | **Probe Chr** | **Gene** | **Probe base pair** | **topSNP** | **SNP Chr** | **SNP base pair** | **Effect allele** | **Other allele** | **Effect allele frequence** | **GWAS association** | | | **mQTL association** | | | **SMR association** | | | **HEIDI Test** | | **BH correction** | **PP.H4** |
|  |  |  |  |  |  |  |  |  |  |  |  | **β** | **SE** | ***P*** | **β** | **SE** | ***P*** | **β** | **SE** | ***P*** | ***P*** | **nsnp** | ***P-FDR*** |  |
| Inflammatory bowel disease | 1-stearoyl-2-linoleoyl-GPI levels | cg03735013 | 11 | FADS1 | 61582769 | rs174561 | 11 | 61582708 | C | T | 0.303181 | 0.191421 | 0.016009 | 5.96E-33 | -0.279615 | 0.0334227 | 5.96E-17 | -0.684588 | 0.0998702 | 7.14E-12 | 0.08846633 | 20 | 4.14E-08 | 0.88463613 |
|  |  | cg12517394 | 11 | FADS1 | 61582795 | rs174592 | 11 | 61618608 | G | A | 0.38668 | 0.188226 | 0.0153555 | 1.52E-34 | -0.274167 | 0.0318942 | 8.24E-18 | -0.686538 | 0.0975471 | 1.95E-12 | 0.07996548 | 20 | 1.29E-08 | 0.88463613 |
|  |  | cg19610905 | 11 | FADS2 | 61596333 | rs174566 | 11 | 61592362 | G | A | 0.355865 | 0.206552 | 0.0154795 | 1.29E-40 | -0.851216 | 0.0301912 | 6.89E-175 | -0.242655 | 0.020119 | 1.70E-33 | 0.05910825 | 20 | 1.57E-28 | 0.000384623 |
|  |  | cg19250101 | 19 | TMC4 | 54666215 | rs36643 | 19 | 54640480 | C | T | 0.372763 | 0.160332 | 0.0151092 | 2.63E-26 | 0.242633 | 0.0333611 | 3.52E-13 | 0.6608 | 0.110149 | 1.98E-09 | 0.0648465 | 13 | 7.99E-06 | 0.00058633 |
| IBD | Mannose to glycerol ratio | cg15478930 | 2 | NRBP1 | 27652102 | rs7597145 | 2 | 27671255 | C | T | 0.375746 | 0.0810601 | 0.0139273 | 5.88E-09 | 0.228603 | 0.0323583 | 1.61E-12 | 0.354589 | 0.0789357 | 7.05E-06 | 0.07492799 | 17 | 0.046688033 | 0.79347613 |
| Ulcerative colitis | N-acetyl-isoputreanine levels | cg03634777 | 6 | IGF2R | 160512657 | rs662138 | 6 | 160564476 | G | C | 0.184891 | 0.138468 | 0.0197639 | 2.45E-12 | 0.27711 | 0.0410753 | 1.52E-11 | 0.499686 | 0.102824 | 1.18E-06 | 0.3181638 | 5 | 0.001703172 | 0.003836193 |
|  |  | cg03469471 | 7 | NOS3 | 150707830 | rs891512 | 7 | 150708089 | A | G | 0.249503 | -0.13393 | 0.0182519 | 2.17E-13 | -0.242835 | 0.0391714 | 5.67E-10 | 0.551527 | 0.116466 | 2.18E-06 | 0.06208321 | 6 | 0.00293501 | 0.008347818 |
|  |  | cg04994447 | 7 | ATG9B | 150718483 | rs12539718 | 7 | 150718522 | G | C | 0.253479 | 0.0853352 | 0.0174081 | 9.48E-07 | 0.323579 | 0.0376631 | 8.59E-18 | 0.263723 | 0.0619398 | 2.07E-05 | 0.1119615 | 13 | 0.020149836 | 0.000384613 |
|  |  | cg25020570 | 10 | GPR123 | 134943284 | rs61862687 | 10 | 134953254 | A | G | 0.163022 | 0.091696 | 0.0206578 | 9.05E-06 | 0.669526 | 0.0450618 | 6.18E-50 | 0.136957 | 0.0322018 | 2.11E-05 | 0.1968703 | 18 | 0.020359643 | 8.3623E-06 |
|  |  | cg25142028 | 10 | GPR123 | 134943309 | rs61862687 | 10 | 134953254 | A | G | 0.163022 | 0.091696 | 0.0206578 | 9.05E-06 | 0.504211 | 0.0450408 | 4.34E-29 | 0.18186 | 0.0440738 | 3.69E-05 | 0.09315514 | 15 | 0.030243595 | 8.39488E-06 |
|  |  | cg18550632 | 10 | KNDC1 | 135001604 | rs11594978 | 10 | 135001595 | A | G | 0.373757 | -0.0690703 | 0.0156872 | 1.07E-05 | -0.405305 | 0.032851 | 5.68E-35 | 0.170416 | 0.0410955 | 3.37E-05 | 0.1794036 | 20 | 0.028668709 | 3.84623E-06 |
|  |  | cg17838816 | 10 | KNDC1 | 135015675 | rs3008382 | 10 | 135017243 | A | C | 0.439364 | -0.0669456 | 0.0152344 | 1.11E-05 | -0.418169 | 0.0316267 | 6.55E-40 | 0.160092 | 0.0383906 | 3.04E-05 | 0.2425882 | 20 | 0.02662513 | 3.84494E-06 |
|  |  | cg12077322 | 10 | TUBGCP2 | 135098662 | rs113697323 | 10 | 135148616 | G | C | 0.0298211 | 0.388162 | 0.0488293 | 1.87E-15 | -0.674909 | 0.109205 | 6.40E-10 | -0.575132 | 0.117876 | 1.07E-06 | 0.1831423 | 7 | 0.001592957 | 0.000382762 |
|  |  | cg09915092 | 10 | ECHS1 | 135187482 | rs4838721 | 10 | 135160950 | C | T | 0.189861 | 0.155571 | 0.0195835 | 1.96E-15 | -0.315526 | 0.0425715 | 1.25E-13 | -0.493053 | 0.0909816 | 5.98E-08 | 0.05051245 | 20 | 0.000118026 | 0.000348461 |
|  |  | cg17833184 | 10 | PAOX | 135191291 | rs2860672 | 10 | 135107301 | C | T | 0.0745527 | 0.171814 | 0.0282559 | 1.20E-09 | 0.383318 | 0.0609902 | 3.28E-10 | 0.448228 | 0.102567 | 1.24E-05 | 0.08395646 | 5 | 0.0132319 | 0.92491633 |
|  |  | cg05296940 | 10 | PAOX | 135193666 | rs7081338 | 10 | 135104493 | C | G | 0.198807 | 0.18765 | 0.0190524 | 6.91E-23 | -0.224881 | 0.0404591 | 2.73E-08 | -0.834441 | 0.172383 | 1.29E-06 | 0.2078684 | 8 | 0.00184583 | 0.924993752 |
|  |  | cg11631091 | 10 | MTG1 | 135217018 | rs2265909 | 10 | 135210261 | A | G | 0.340954 | 0.131453 | 0.0159115 | 1.44E-16 | 0.305442 | 0.0363437 | 4.31E-17 | 0.43037 | 0.0730481 | 3.82E-09 | 0.5325816 | 9 | 9.09E-06 | 0.8634672 |
|  |  | cg12133118 | 17 | SLC47A1 | 19436770 | rs2453580 | 17 | 19438321 | C | T | 0.406561 | 0.0716741 | 0.015461 | 3.56E-06 | 0.287925 | 0.0342867 | 4.56E-17 | 0.248933 | 0.0613369 | 4.94E-05 | 0.5690088 | 14 | 0.03880278 | 4.84713E-05 |
|  |  | cg21692194 | 17 | SLC47A1 | 19436881 | rs8071709 | 17 | 19429790 | C | A | 0.406561 | 0.0734686 | 0.0156411 | 2.64E-06 | 0.449507 | 0.0337423 | 1.73E-40 | 0.163443 | 0.0368957 | 9.43E-06 | 0.2284201 | 20 | 0.010659329 | 4.89586E-05 |
|  |  | cg25387636 | 17 | SLC47A1 | 19436896 | rs8071709 | 17 | 19429790 | C | A | 0.406561 | 0.0734686 | 0.0156411 | 2.64E-06 | 0.436442 | 0.03374 | 2.84E-38 | 0.168335 | 0.0381274 | 1.01E-05 | 0.238104 | 20 | 0.01127635 | 4.87435E-05 |
|  |  | cg15971010 | 17 | SLC47A1 | 19436900 | rs8071709 | 17 | 19429790 | C | A | 0.406561 | 0.0734686 | 0.0156411 | 2.64E-06 | 0.550966 | 0.0334535 | 6.07E-61 | 0.133345 | 0.0295205 | 6.27E-06 | 0.2495602 | 20 | 0.007549178 | 4.86383E-05 |
|  |  | cg20930201 | 17 | SLC47A1 | 19437691 | rs11871125 | 17 | 19432393 | T | C | 0.403579 | 0.073129 | 0.0154861 | 2.33E-06 | 0.374615 | 0.0337229 | 1.14E-28 | 0.195211 | 0.0449188 | 1.39E-05 | 0.768575 | 13 | 0.014287586 | 4.84859E-05 |
| GWAS: genome-wide association study.  mQTL: methylation quantitative trait loci.  SMR: summary-data-based Mendelian randomization.  HEIDI: heterogeneity in dependent instruments. IBD: Inflammatory bowel disease. Only genome-wide significant mQTLs (P<5E-8) are taken into the analysis. We report SNP-gene combinations with P_SMR_ < genome-wide significance Benjamin Hochberg correction threshold of PFDR＜0.05, and survived after the heterogeneity test (PHEIDI＞0.05). β in GWAS association, regression coefficient of cancer on SNP, log(OR).  SE, standard error.  β in eQTL association, regression coefficient of gene expression on SNP.  β in SMR association, regression coefficient of cancer on gene expression.  PP.H4, posterior probability of H4 ,PP.H4＞0.75 as the cut-off for the evidence of colocalization of cancer GWAS and eQTL association. | | | | | | | | | | | | | | | | | | | | | | | | |

| **Table S10. SMR and colocalization results from mQTL of diverse metabolites causally associated with other digestive disorders outcomes** | | | | | | | | | | | | | | | | | | | | | | | | |
| --- | --- | --- | --- | --- | --- | --- | --- | --- | --- | --- | --- | --- | --- | --- | --- | --- | --- | --- | --- | --- | --- | --- | --- | --- |
| **Type of disease** | **Type of metabolites** | **probeID** | **Probe Chr** | **Gene** | **Probe base pair** | **topSNP** | **SNP Chr** | **SNP base pair** | **Effect allele** | **Other allele** | **Effect allele frequence** | **GWAS association** | | | **mQTL association** | | | **SMR association** | | | **HEIDI Test** | | **BH correction** | **PP.H4** |
|  |  |  |  |  |  |  |  |  |  |  |  | **β** | **SE** | ***P*** | **β** | **SE** | ***P*** | **β** | **SE** | ***P*** | ***P*** | **nsnp** | ***P-FDR*** |  |
| Non-alcoholic liver disease | Bilirubin degradation product levels | cg02901644 | 2 | DGKD | 234369787 | rs2305538 | 2 | 234376177 | T | C | 0.467197 | 0.140196 | 0.0147904 | 2.57E-21 | -0.25569 | 0.0331027 | 1.13E-14 | -0.548305 | 0.0915699 | 2.13E-09 | 0.2569399 | 20 | 1.79E-05 | 0.00484624 |
|  |  | cg03607648 | 2 | UGT1A10 | 234668930 | rs6744284 | 2 | 234625297 | T | C | 0.274354 | 0.542892 | 0.0155139 | 1.00E-200 | -0.223621 | 0.0354517 | 2.83E-10 | -2.42773 | 0.391082 | 5.38E-10 | 0.1961674 | 18 | 4.98E-06 | 0.1937461 |
|  | Imidazole lactate levels | cg14669130 | 1 | CCBL2 | 89459504 | rs1409150 | 1 | 89473175 | C | T | 0.436382 | 0.103551 | 0.0151479 | 8.15E-12 | -0.813995 | 0.0288048 | 1.10E-175 | -0.127213 | 0.0191461 | 3.05E-11 | 0.1704108 | 20 | 2.57E-07 | 0.7937361 |
|  |  | cg11309454 | 1 | CCBL2 | 89459529 | rs1409150 | 1 | 89473175 | C | T | 0.436382 | 0.103551 | 0.0151479 | 8.15E-12 | -0.990014 | 0.0255321 | 0 | -0.104595 | 0.0155367 | 1.67E-11 | 0.1569272 | 20 | 1.72E-07 | 0.7937764 |
|  |  | cg23627354 | 1 | CCBL2 | 89459658 | rs1409150 | 1 | 89473175 | C | T | 0.436382 | 0.103551 | 0.0151479 | 8.15E-12 | -0.928225 | 0.0266315 | 3.65E-266 | -0.111558 | 0.0166301 | 1.97E-11 | 0.1256441 | 20 | 1.83E-07 | 0.79376347 |
|  |  | cg13939156 | 17 | NA | 80058883 | rs8080682 | 17 | 80058901 | G | A | 0.516899 | -0.0978643 | 0.0152169 | 1.27E-10 | -0.474722 | 0.0321227 | 2.02E-49 | 0.206151 | 0.0349581 | 3.70E-09 | 0.4157254 | 20 | 2.29E-05 | 0.0018365 |
|  |  | cg03388043 | 17 | CCDC57 | 80084554 | rs78303892 | 17 | 80088941 | A | G | 0.483101 | -0.0882633 | 0.0153789 | 9.51E-09 | -0.231613 | 0.0331516 | 2.82E-12 | 0.381081 | 0.0859305 | 9.22E-06 | 0.06896817 | 16 | 0.01654175 | 3.84613E-06 |
|  |  | cg21773646 | 17 | CCDC57 | 80085082 | rs78303892 | 17 | 80088941 | A | G | 0.483101 | -0.0882633 | 0.0153789 | 9.51E-09 | -0.230543 | 0.0334551 | 5.54E-12 | 0.38285 | 0.0868126 | 1.03E-05 | 0.08875324 | 17 | 0.018072911 | 3.84857E-06 |
|  |  | cg22476252 | 17 | CCDC57 | 80087405 | rs7406630 | 17 | 80084080 | C | T | 0.483101 | -0.0877041 | 0.0153764 | 1.17E-08 | -0.273502 | 0.0333626 | 2.45E-16 | 0.320671 | 0.0684896 | 2.84E-06 | 0.07644287 | 20 | 0.00612283 | 3.82572E-06 |
|  |  | cg01048272 | 17 | CCDC57 | 80169878 | rs4789784 | 17 | 80179213 | G | A | 0.287276 | 0.0848237 | 0.0161018 | 1.38E-07 | -0.327272 | 0.0336443 | 2.30E-22 | -0.259184 | 0.0559517 | 3.62E-06 | 0.09829113 | 20 | 0.00728737 | 3.88635E-06 |
|  | Bilirubin (z,z) levels | cg02901644 | 2 | DGKD | 234369787 | rs2305538 | 2 | 234376177 | T | C | 0.467197 | 0.164291 | 0.0148729 | 2.28E-28 | -0.25569 | 0.0331027 | 1.13E-14 | -0.64254 | 0.101506 | 2.45E-10 | 0.1549523 | 20 | 2.27E-06 | 0.0038361 |
|  |  | cg23874600 | 2 | UGT1A10 | 234601954 | rs75444879 | 2 | 234587848 | A | G | 0.333002 | 0.621481 | 0.0143621 | 1.00E-200 | -0.194878 | 0.0345072 | 1.63E-08 | -3.18908 | 0.569482 | 2.14E-08 | 0.02946457 | 18 | 0.000124207 | 0.77354813 |
|  |  | cg08697797 | 2 | UGT1A10 | 234668834 | rs887829 | 2 | 234668570 | T | C | 0.298211 | 0.72876 | 0.0139548 | 1.00E-200 | 0.294621 | 0.0340171 | 4.68E-18 | 2.47355 | 0.289498 | 1.29E-17 | 0.0554802 | 20 | 3.00E-13 | 0.773563458 |
|  |  | cg03607648 | 2 | UGT1A10 | 234668930 | rs6744284 | 2 | 234625297 | T | C | 0.274354 | 0.652561 | 0.0151774 | 1.00E-200 | -0.223621 | 0.0354517 | 2.83E-10 | -2.91816 | 0.467581 | 4.35E-10 | 0.2461008 | 18 | 3.36E-06 | 0.773558792 |
|  | Biliverdin levels | cg02901644 | 2 | DGKD | 234369787 | rs2305538 | 2 | 234376177 | T | C | 0.467197 | 0.145086 | 0.0144245 | 8.44E-24 | -0.25569 | 0.0331027 | 1.13E-14 | -0.567429 | 0.0926239 | 9.00E-10 | 0.153679 | 20 | 7.59E-06 | 0.01937623 |
|  |  | cg08697797 | 2 | UGT1A10 | 234668834 | rs887829 | 2 | 234668570 | T | C | 0.298211 | 0.586012 | 0.0141468 | 1.00E-200 | 0.294621 | 0.0340171 | 4.68E-18 | 1.98904 | 0.234621 | 2.30E-17 | 0.06313895 | 20 | 5.32E-13 | 0.8465133 |
|  |  | cg03607648 | 2 | UGT1A10 | 234668930 | rs6744284 | 2 | 234625297 | T | C | 0.274354 | 0.525876 | 0.0151638 | 1.00E-200 | -0.223621 | 0.0354517 | 2.83E-10 | -2.35164 | 0.378933 | 5.44E-10 | 0.230443 | 18 | 5.04E-06 | 0.846575464 |
| Acute gastritis | Nervonoylcarnitine levels | cg24117468 | 5 | P4HA2 | 131562848 | rs12521097 | 5 | 131575338 | A | G | 0.4334 | -0.11414 | 0.0156691 | 3.23E-13 | 0.697767 | 0.0286919 | 1.23E-130 | -0.163579 | 0.0234418 | 2.99E-12 | 0.3635854 | 20 | 2.52E-08 | 3.84385E-07 |
|  |  | cg18318560 | 5 | P4HA2 | 131563015 | rs12521097 | 5 | 131575338 | A | G | 0.4334 | -0.11414 | 0.0156691 | 3.23E-13 | 0.366916 | 0.030843 | 1.24E-32 | -0.311079 | 0.0500749 | 5.22E-10 | 0.384226 | 20 | 2.55E-06 | 3.84635E-07 |
|  |  | cg22598563 | 5 | P4HA2 | 131563921 | rs13357280 | 5 | 131600187 | G | A | 0.424453 | -0.114717 | 0.0157092 | 2.82E-13 | -0.422967 | 0.030531 | 1.21E-43 | 0.27122 | 0.0419844 | 1.05E-10 | 0.08587797 | 20 | 5.71E-07 | 3.85826E-07 |
|  |  | cg06968155 | 5 | SLC22A5 | 131705112 | rs11242109 | 5 | 131677047 | T | G | 0.44334 | -0.126411 | 0.0155821 | 4.95E-16 | 0.372312 | 0.03102 | 3.45E-33 | -0.33953 | 0.0505159 | 1.80E-11 | 0.2346632 | 20 | 1.28E-07 | 5.87838E-05 |
|  |  | cg07538946 | 5 | SLC22A5 | 131705188 | rs200838 | 5 | 131710399 | A | C | 0.444334 | -0.125233 | 0.0155767 | 9.00E-16 | 0.360171 | 0.0310322 | 3.83E-31 | -0.347704 | 0.0526107 | 3.87E-11 | 0.1429773 | 20 | 2.56E-07 | 5.87809E-05 |
|  |  | cg19040266 | 5 | SLC22A5 | 131723239 | rs200838 | 5 | 131710399 | A | C | 0.444334 | -0.125233 | 0.0155767 | 9.00E-16 | -0.348672 | 0.030522 | 3.19E-30 | 0.359171 | 0.0546291 | 4.87E-11 | 0.2557896 | 20 | 3.01E-07 | 5.87898E-05 |
|  |  | cg03954425 | 8 | FAM82B | 87520894 | rs34219490 | 8 | 87502042 | G | C | 0.266402 | 0.207982 | 0.0174259 | 7.76E-33 | 0.346204 | 0.0377702 | 4.91E-20 | 0.60075 | 0.0826384 | 3.60E-13 | 0.2822255 | 16 | 3.71E-09 | 9.43782E-07 |
|  |  | cg05049323 | 8 | FAM82B | 87520900 | rs7465511 | 8 | 87521481 | T | C | 0.26839 | 0.209263 | 0.0174116 | 2.84E-33 | -0.524077 | 0.0365903 | 1.58E-46 | -0.399298 | 0.0433705 | 3.36E-20 | 0.3810098 | 20 | 5.20E-16 | 9.43786E-07 |
|  |  | cg27223183 | 8 | FAM82B | 87520930 | rs7465511 | 8 | 87521481 | T | C | 0.26839 | 0.209263 | 0.0174116 | 2.84E-33 | -0.895455 | 0.0340099 | 8.87E-153 | -0.233695 | 0.0213744 | 7.98E-28 | 0.1986406 | 20 | 7.40E-23 | 9.43836E-07 |
|  |  | cg07448949 | 8 | FAM82B | 87521177 | rs9297923 | 8 | 87548989 | T | C | 0.265408 | 0.200015 | 0.0174541 | 2.11E-30 | 0.486538 | 0.037158 | 3.57E-39 | 0.411098 | 0.0476727 | 6.50E-18 | 0.1516395 | 20 | 8.61E-14 | 9.43563E-07 |
|  |  | cg00550725 | 8 | FAM82B | 87521180 | rs6985066 | 8 | 87526439 | C | A | 0.266402 | 0.20415 | 0.0174662 | 1.46E-31 | 0.69842 | 0.0364448 | 7.43E-82 | 0.292303 | 0.0292926 | 1.89E-23 | 0.1350965 | 20 | 5.84E-19 | 9.43318E-07 |
|  |  | cg05786429 | 8 | FAM82B | 87521202 | rs7462883 | 8 | 87526790 | A | G | 0.266402 | 0.204325 | 0.017459 | 1.23E-31 | 0.664719 | 0.0365366 | 5.84E-74 | 0.307386 | 0.0312302 | 7.38E-23 | 0.05399705 | 20 | 1.44E-18 | 9.43529E-07 |
|  |  | cg07667469 | 8 | FAM82B | 87521214 | rs56752411 | 8 | 87515156 | T | C | 0.266402 | 0.208465 | 0.0174283 | 5.67E-33 | 0.288781 | 0.0368685 | 4.77E-15 | 0.721879 | 0.110164 | 5.65E-11 | 0.215856 | 11 | 3.27E-07 | 9.43746E-07 |
|  |  | cg15253520 | 8 | FAM82B | 87521369 | rs7462883 | 8 | 87526790 | A | G | 0.266402 | 0.204325 | 0.017459 | 1.23E-31 | 0.702156 | 0.0361318 | 4.05E-84 | 0.290997 | 0.0290256 | 1.18E-23 | 0.06379947 | 20 | 5.46E-19 | 9.43118E-07 |
| Chronic pancreatitis | 1-palmitoyl-2-dihomo-linolenoyl-GPC levels | cg04074454 | 11 | C11orf9 | 61543928 | rs174529 | 11 | 61543961 | C | T | 0.383698 | 0.308078 | 0.0155511 | 2.40E-87 | -0.23121 | 0.0326113 | 1.34E-12 | -1.33246 | 0.199611 | 2.47E-11 | 0.06370023 | 20 | 1.14E-07 | 0.0018374 |
|  |  | cg03735013 | 11 | FADS1 | 61582769 | rs174561 | 11 | 61582708 | C | T | 0.303181 | 0.334298 | 0.0160054 | 7.10E-97 | -0.279615 | 0.0334227 | 5.96E-17 | -1.19557 | 0.153945 | 8.09E-15 | 0.05479865 | 20 | 4.41E-11 | 7.28456E-05 |
|  |  | cg25326896 | 11 | FADS1 | 61582785 | rs174561 | 11 | 61582708 | C | T | 0.303181 | 0.334298 | 0.0160054 | 7.10E-97 | -0.346722 | 0.0336723 | 7.27E-25 | -0.964167 | 0.104397 | 2.57E-20 | 0.3134997 | 20 | 1.83E-16 | 7.28234E-05 |
|  |  | cg12517394 | 11 | FADS1 | 61582795 | rs174592 | 11 | 61618608 | G | A | 0.38668 | 0.296228 | 0.0154153 | 2.70E-82 | -0.274167 | 0.0318942 | 8.24E-18 | -1.08047 | 0.137695 | 4.27E-15 | 0.1947175 | 20 | 2.47E-11 | 7.28347E-05 |
|  |  | cg16213375 | 11 | FADS1 | 61584727 | rs174560 | 11 | 61581764 | C | T | 0.313121 | 0.331131 | 0.015949 | 9.59E-96 | -0.434186 | 0.033468 | 1.74E-38 | -0.762648 | 0.0693194 | 3.74E-28 | 0.3078618 | 20 | 2.89E-24 | 7.28248E-05 |
|  |  | cg10515671 | 11 | FADS1 | 61585899 | rs174533 | 11 | 61549025 | A | G | 0.347913 | 0.327597 | 0.0156428 | 2.20E-97 | 0.260664 | 0.0334249 | 6.26E-15 | 1.25678 | 0.171967 | 2.71E-13 | 0.3639376 | 20 | 1.39E-09 | 7.28895E-05 |
|  |  | cg07005513 | 11 | FADS2 | 61595956 | rs174559 | 11 | 61581656 | A | G | 0.267396 | 0.335807 | 0.0164876 | 3.26E-92 | -0.326923 | 0.034982 | 9.15E-21 | -1.02717 | 0.12093 | 2.00E-17 | 0.07715787 | 20 | 1.23E-13 | 0.833755571 |
|  |  | cg14911132 | 11 | FADS2 | 61596755 | rs968567 | 11 | 61595564 | T | C | 0.150099 | 0.384062 | 0.0192691 | 2.17E-88 | -0.563176 | 0.0385648 | 2.67E-48 | -0.681957 | 0.0578915 | 4.95E-32 | 0.3440362 | 20 | 4.59E-28 | 0.833756347 |
|  |  | cg07591205 | 11 | FADS2 | 61633557 | rs174544 | 11 | 61567753 | A | C | 0.304175 | 0.334 | 0.0160203 | 1.57E-96 | 0.24516 | 0.0335969 | 2.94E-13 | 1.36238 | 0.197806 | 5.68E-12 | 0.3581699 | 19 | 2.77E-08 | 0.833755792 |
| GWAS: genome-wide association study.  mQTL: methylation quantitative trait loci.  SMR: summary-data-based Mendelian randomization.  HEIDI: heterogeneity in dependent instruments. Only genome-wide significant mQTLs (P<5E-8) are taken into the analysis. We report SNP-gene combinations with P_SMR_ < genome-wide significance Benjamin Hochberg correction threshold of PFDR＜0.05, and survived after the heterogeneity test (PHEIDI＞0.05). β in GWAS association, regression coefficient of cancer on SNP, log(OR).  SE, standard error.  β in eQTL association, regression coefficient of gene expression on SNP.  β in SMR association, regression coefficient of cancer on gene expression.  PP.H4, posterior probability of H4 ,PP.H4＞0.75 as the cut-off for the evidence of colocalization of cancer GWAS and eQTL association. | | | | | | | | | | | | | | | | | | | | | | | | |

| **Table S11. SMR results of the relationship between DNA methylation and causal metabolite-related gene expression.** | | | | | | | | | | | | | | | | | | | | | | | | | |
| --- | --- | --- | --- | --- | --- | --- | --- | --- | --- | --- | --- | --- | --- | --- | --- | --- | --- | --- | --- | --- | --- | --- | --- | --- | --- |
| **Expo Probe** | **Expo Chr** | **Expo Gene** | **Expo base pair** | **Outco Probe** | **Outco Chr** | **Outco Gene** | **Outco base pair** | **topSNP** | **SNP Chr** | **SNP base pair** | **Effect allele** | **Other allele** | **Effect allele frequence** | **mQTL association** | | | **eQTL association** | | | **SMR association** | | | **HEIDI Test** | | **BH correction** |
|  |  |  |  |  |  |  |  |  |  |  |  |  |  | **β** | **SE** | ***P*** | **β** | **SE** | ***P*** | **β** | **SE** | ***P*** | ***P*** | **nsnp** | ***P-FDR*** |
| cg08107208 | 1 | USP1 | 62901199 | ENSG00000116641 | 1 | DOCK7 | 62920399 | rs7531579 | 1 | 63147040 | C | A | 0.301193 | -0.31 | 0.0341505 | 1.11E-19 | 0.256853 | 0.0330247 | 7.39E-15 | -1.20692 | 0.204348 | 3.50E-09 | 0.1013631 | 7 | 2.05E-05 |
| cg10830713 | 1 | USP1 | 62904021 | ENSG00000116641 | 1 | DOCK7 | 62920399 | rs1168128 | 1 | 63135846 | C | G | 0.305169 | -0.319638 | 0.0339173 | 4.34E-21 | 0.189974 | 0.0345335 | 3.77E-08 | -1.68254 | 0.354147 | 2.02E-06 | 0.1294725 | 4 | 0.01186037 |
| cg26877327 | 1 | USP1 | 62907616 | ENSG00000116641 | 1 | DOCK7 | 62920399 | rs1168107 | 1 | 63161173 | A | T | 0.308151 | -0.320155 | 0.0340868 | 5.87E-21 | -0.19179 | 0.0331407 | 7.16E-09 | 1.6693 | 0.338809 | 8.35E-07 | 0.32418 | 5 | 0.004892333 |
| cg01744354 | 2 | IFT172 | 27712243 | ENSG00000115216 | 2 | NRBP1 | 27650657 | rs1260327 | 2 | 27711893 | G | A | 0.440358 | -0.153595 | 0.0184666 | 8.99E-17 | 0.28987 | 0.0319967 | 1.31E-19 | -0.529875 | 0.0864842 | 8.96E-10 | 0.09806573 | 19 | 5.25E-06 |
| cg26672776 | 6 | SFTA2 | 30899568 | ENSG00000204304 | 6 | PBX2 | 32152512 | rs9270585 | 6 | 32561300 | C | T | 0.427435 | 0.0800365 | 0.0165373 | 1.30E-06 | 0.407412 | 0.0320672 | 5.55E-37 | 0.196451 | 0.0434366 | 6.11E-06 | 0.5085005 | 4 | 0.035765064 |
| cg13561028 | 6 | SFTA2 | 30899649 | ENSG00000204304 | 6 | PBX2 | 32152512 | rs9270591 | 6 | 32561424 | C | T | 0.422465 | 0.0800365 | 0.0165373 | 1.30E-06 | 0.419672 | 0.0320037 | 2.77E-39 | 0.190712 | 0.0420036 | 5.62E-06 | 0.5443636 | 4 | 0.032894668 |
| cg04390683 | 6 | SFTA2 | 30900149 | ENSG00000204304 | 6 | PBX2 | 32152512 | rs9270591 | 6 | 32561424 | C | T | 0.422465 | 0.0800365 | 0.0165373 | 1.30E-06 | 0.380589 | 0.032316 | 5.12E-32 | 0.210296 | 0.0469779 | 7.59E-06 | 0.8207987 | 4 | 0.044449573 |
| cg04559908 | 6 | DPCR1 | 30920123 | ENSG00000204304 | 6 | PBX2 | 32152512 | rs9270591 | 6 | 32561424 | C | T | 0.422465 | 0.0800365 | 0.0165373 | 1.30E-06 | 0.411052 | 0.0321663 | 2.15E-37 | 0.194711 | 0.0430204 | 6.01E-06 | 0.4985216 | 4 | 0.035209122 |
| cg22731440 | 6 | HLA-B | 31323506 | ENSG00000204304 | 6 | PBX2 | 32152512 | rs9270591 | 6 | 32561424 | C | T | 0.422465 | 0.0800365 | 0.0165373 | 1.30E-06 | 0.417718 | 0.0321589 | 1.41E-38 | 0.191604 | 0.0422486 | 5.76E-06 | 0.3230519 | 4 | 0.03371781 |
| cg04985482 | 6 | MICA | 31382065 | ENSG00000204304 | 6 | PBX2 | 32152512 | rs9270560 | 6 | 32560741 | T | C | 0.423459 | 0.0788637 | 0.0165671 | 1.93E-06 | -0.405249 | 0.0321483 | 1.97E-36 | -0.194606 | 0.0436992 | 8.46E-06 | 0.8576011 | 3 | 0.049535102 |
| cg23224191 | 6 | MCCD1 | 31497414 | ENSG00000204304 | 6 | PBX2 | 32152512 | rs9270591 | 6 | 32561424 | C | T | 0.422465 | 0.0800365 | 0.0165373 | 1.30E-06 | -0.422441 | 0.0317419 | 2.06E-40 | -0.189462 | 0.0416553 | 5.41E-06 | 0.1267198 | 4 | 0.031675864 |
| cg23634079 | 6 | MSH5 | 31712195 | ENSG00000204304 | 6 | PBX2 | 32152512 | rs9270591 | 6 | 32561424 | C | T | 0.422465 | 0.0800365 | 0.0165373 | 1.30E-06 | 0.481363 | 0.0319661 | 3.03E-51 | 0.166271 | 0.036086 | 4.07E-06 | 0.3605559 | 4 | 0.023860009 |
| cg06431527 | 6 | PBX2 | 32158513 | ENSG00000204304 | 6 | PBX2 | 32152512 | rs2760981 | 6 | 32565465 | G | A | 0.44334 | 0.0812975 | 0.0164367 | 7.57E-07 | 0.489511 | 0.0320674 | 1.31E-52 | 0.166079 | 0.0352965 | 2.54E-06 | 0.2036559 | 4 | 0.014852748 |
| cg11981868 | 6 | NOTCH4 | 32185954 | ENSG00000204304 | 6 | PBX2 | 32152512 | rs9270591 | 6 | 32561424 | C | T | 0.422465 | 0.0800365 | 0.0165373 | 1.30E-06 | -0.430741 | 0.0322312 | 9.80E-41 | -0.185811 | 0.0408328 | 5.35E-06 | 0.2442055 | 4 | 0.031346129 |
| cg00366603 | 6 | NOTCH4 | 32186049 | ENSG00000204304 | 6 | PBX2 | 32152512 | rs9270591 | 6 | 32561424 | C | T | 0.422465 | 0.0800365 | 0.0165373 | 1.30E-06 | 0.668676 | 0.0301828 | 9.51E-109 | 0.119694 | 0.0253147 | 2.26E-06 | 0.1293047 | 4 | 0.013267263 |
| cg12179641 | 6 | NOTCH4 | 32188404 | ENSG00000204304 | 6 | PBX2 | 32152512 | rs2760981 | 6 | 32565465 | G | A | 0.44334 | 0.0812975 | 0.0164367 | 7.57E-07 | -0.366857 | 0.032855 | 5.99E-29 | -0.221605 | 0.0490031 | 6.12E-06 | 0.44911 | 4 | 0.035838711 |
| cg21241195 | 6 | C6orf10 | 32289357 | ENSG00000204304 | 6 | PBX2 | 32152512 | rs9270591 | 6 | 32561424 | C | T | 0.422465 | 0.0800365 | 0.0165373 | 1.30E-06 | 0.561047 | 0.0313655 | 1.48E-71 | 0.142656 | 0.0305357 | 2.99E-06 | 0.363899 | 4 | 0.017493581 |
| cg13966843 | 6 | C6orf10 | 32336160 | ENSG00000204304 | 6 | PBX2 | 32152512 | rs9270591 | 6 | 32561424 | C | T | 0.422465 | 0.0800365 | 0.0165373 | 1.30E-06 | 0.547084 | 0.0313943 | 5.22E-68 | 0.146297 | 0.0313723 | 3.11E-06 | 0.4897944 | 4 | 0.01823422 |
| cg15011943 | 6 | HLA-DRB5 | 32493917 | ENSG00000204304 | 6 | PBX2 | 32152512 | rs9270591 | 6 | 32561424 | C | T | 0.422465 | 0.0800365 | 0.0165373 | 1.30E-06 | 0.434738 | 0.0321856 | 1.42E-41 | 0.184103 | 0.0404079 | 5.21E-06 | 0.2725245 | 4 | 0.030526495 |
| cg19383211 | 6 | HLA-DRB6 | 32527588 | ENSG00000204304 | 6 | PBX2 | 32152512 | rs9270560 | 6 | 32560741 | T | C | 0.423459 | 0.0788637 | 0.0165671 | 1.93E-06 | 0.557391 | 0.0316453 | 1.93E-69 | 0.141487 | 0.030789 | 4.32E-06 | 0.7193403 | 3 | 0.025303379 |
| cg08269402 | 6 | HLA-DRB1 | 32549631 | ENSG00000204304 | 6 | PBX2 | 32152512 | rs35139284 | 6 | 32561370 | T | C | 0.304175 | 0.0778266 | 0.017029 | 4.87E-06 | -1.04501 | 0.0275784 | 0 | -0.0744745 | 0.0164136 | 5.70E-06 | 0.5184024 | 4 | 0.033368099 |
| cg04601775 | 6 | HLA-DRB1 | 32557478 | ENSG00000204304 | 6 | PBX2 | 32152512 | rs9270560 | 6 | 32560741 | T | C | 0.423459 | 0.0788637 | 0.0165671 | 1.93E-06 | -0.484973 | 0.0321993 | 2.90E-51 | -0.162615 | 0.0358265 | 5.65E-06 | 0.7521151 | 3 | 0.033117893 |
| cg15820961 | 6 | HLA-DRB1 | 32558459 | ENSG00000204304 | 6 | PBX2 | 32152512 | rs2760981 | 6 | 32565465 | G | A | 0.44334 | 0.0812975 | 0.0164367 | 7.57E-07 | 0.543081 | 0.0312875 | 1.72E-67 | 0.149697 | 0.0314705 | 1.97E-06 | 0.362253 | 4 | 0.011525392 |
| cg23214071 | 6 | HLA-DQB1 | 32627784 | ENSG00000204304 | 6 | PBX2 | 32152512 | rs2760981 | 6 | 32565465 | G | A | 0.44334 | 0.0812975 | 0.0164367 | 7.57E-07 | 0.563611 | 0.0315556 | 2.38E-71 | 0.144244 | 0.0302608 | 1.87E-06 | 0.1582064 | 4 | 0.010970663 |
| cg19301366 | 6 | HLA-DQB1 | 32627845 | ENSG00000204304 | 6 | PBX2 | 32152512 | rs9270560 | 6 | 32560741 | T | C | 0.423459 | 0.0788637 | 0.0165671 | 1.93E-06 | 0.431296 | 0.0317933 | 6.40E-42 | 0.182853 | 0.0407087 | 7.06E-06 | 0.4434542 | 3 | 0.041384028 |
| cg01745539 | 6 | HLA-DQB1 | 32632331 | ENSG00000204304 | 6 | PBX2 | 32152512 | rs9270585 | 6 | 32561300 | C | T | 0.427435 | 0.0800365 | 0.0165373 | 1.30E-06 | 0.547851 | 0.0312803 | 1.12E-68 | 0.146092 | 0.0313171 | 3.09E-06 | 0.9814292 | 3 | 0.018086311 |
| cg10180404 | 6 | HLA-DQB1 | 32632334 | ENSG00000204304 | 6 | PBX2 | 32152512 | rs2760981 | 6 | 32565465 | G | A | 0.44334 | 0.0812975 | 0.0164367 | 7.57E-07 | 0.609681 | 0.0308762 | 8.69E-87 | 0.133344 | 0.0277925 | 1.60E-06 | 0.9791221 | 3 | 0.009394574 |
| cg22984282 | 6 | HLA-DQB1 | 32632568 | ENSG00000204304 | 6 | PBX2 | 32152512 | rs9270560 | 6 | 32560741 | T | C | 0.423459 | 0.0788637 | 0.0165671 | 1.93E-06 | -0.405442 | 0.0322334 | 2.78E-36 | -0.194513 | 0.0436902 | 8.50E-06 | 0.4484517 | 3 | 0.04981444 |
| cg06255955 | 6 | HLA-DQB1 | 32632639 | ENSG00000204304 | 6 | PBX2 | 32152512 | rs9270591 | 6 | 32561424 | C | T | 0.422465 | 0.0800365 | 0.0165373 | 1.30E-06 | -0.441488 | 0.0314365 | 8.41E-45 | -0.181288 | 0.0396201 | 4.75E-06 | 0.5764915 | 4 | 0.027809285 |
| cg03796735 | 6 | HLA-DQB1 | 32632643 | ENSG00000204304 | 6 | PBX2 | 32152512 | rs9270591 | 6 | 32561424 | C | T | 0.422465 | 0.0800365 | 0.0165373 | 1.30E-06 | -0.445096 | 0.0317728 | 1.38E-44 | -0.179819 | 0.0393094 | 4.77E-06 | 0.5056478 | 4 | 0.027971681 |
| cg21588215 | 6 | HLA-DQB1 | 32632937 | ENSG00000204304 | 6 | PBX2 | 32152512 | rs2760981 | 6 | 32565465 | G | A | 0.44334 | 0.0812975 | 0.0164367 | 7.57E-07 | -0.40968 | 0.0319583 | 1.28E-37 | -0.198441 | 0.0430037 | 3.94E-06 | 0.1723506 | 4 | 0.023080953 |
| cg24593918 | 6 | HLA-DQB1 | 32633157 | ENSG00000204304 | 6 | PBX2 | 32152512 | rs2760981 | 6 | 32565465 | G | A | 0.44334 | 0.0812975 | 0.0164367 | 7.57E-07 | -0.622617 | 0.0309336 | 4.23E-90 | -0.130574 | 0.0271848 | 1.56E-06 | 0.06668665 | 4 | 0.009147917 |
| cg23464743 | 6 | HLA-DQB1 | 32633163 | ENSG00000204304 | 6 | PBX2 | 32152512 | rs2760981 | 6 | 32565465 | G | A | 0.44334 | 0.0812975 | 0.0164367 | 7.57E-07 | -0.552491 | 0.0314341 | 3.75E-69 | -0.147147 | 0.0309058 | 1.92E-06 | 0.9880868 | 3 | 0.011275924 |
| cg13353717 | 6 | HLA-DQB1 | 32634276 | ENSG00000204304 | 6 | PBX2 | 32152512 | rs9270585 | 6 | 32561300 | C | T | 0.427435 | 0.0800365 | 0.0165373 | 1.30E-06 | -0.380202 | 0.0324207 | 9.25E-32 | -0.21051 | 0.0470547 | 7.69E-06 | 0.9095286 | 3 | 0.045027289 |
| cg23336481 | 6 | HLA-DQB1 | 32634344 | ENSG00000204304 | 6 | PBX2 | 32152512 | rs9270585 | 6 | 32561300 | C | T | 0.427435 | 0.0800365 | 0.0165373 | 1.30E-06 | -0.452246 | 0.0315375 | 1.23E-46 | -0.176976 | 0.0385936 | 4.53E-06 | 0.9498236 | 3 | 0.02651588 |
| cg19350679 | 6 | HLA-DQA2 | 32710506 | ENSG00000204304 | 6 | PBX2 | 32152512 | rs2760981 | 6 | 32565465 | G | A | 0.44334 | 0.0812975 | 0.0164367 | 7.57E-07 | -0.383899 | 0.0323445 | 1.71E-32 | -0.211768 | 0.0463841 | 4.98E-06 | 0.9210975 | 3 | 0.02918687 |
| cg10645648 | 6 | HLA-DQA2 | 32711617 | ENSG00000204304 | 6 | PBX2 | 32152512 | rs2760981 | 6 | 32565465 | G | A | 0.44334 | 0.0812975 | 0.0164367 | 7.57E-07 | 0.796474 | 0.0291614 | 3.00E-164 | 0.102072 | 0.0209725 | 1.13E-06 | 0.4379946 | 4 | 0.006639943 |
| cg07180897 | 6 | HLA-DQB2 | 32729130 | ENSG00000204304 | 6 | PBX2 | 32152512 | rs2760981 | 6 | 32565465 | G | A | 0.44334 | 0.0812975 | 0.0164367 | 7.57E-07 | 0.683481 | 0.0301619 | 1.10E-113 | 0.118946 | 0.0246148 | 1.35E-06 | 0.5354449 | 4 | 0.007905681 |
| cg24080129 | 6 | TAP2 | 32797488 | ENSG00000204304 | 6 | PBX2 | 32152512 | rs2760981 | 6 | 32565465 | G | A | 0.44334 | 0.0812975 | 0.0164367 | 7.57E-07 | 0.369671 | 0.032479 | 5.15E-30 | 0.219919 | 0.04848 | 5.73E-06 | 0.8829653 | 3 | 0.033540811 |
| cg02680487 | 6 | DDR1 | 30851529 | ENSG00000196301 | 6 | HLA-DRB9 | 32427598 | rs9270555 | 6 | 32560584 | C | T | 0.417495 | -0.309966 | 0.0432997 | 8.15E-13 | -0.18827 | 0.0322829 | 5.48E-09 | 1.64639 | 0.364132 | 6.14E-06 | 0.1980006 | 17 | 0.035984839 |
| cg16537676 | 6 | DDR1 | 30851624 | ENSG00000196301 | 6 | HLA-DRB9 | 32427598 | rs9274623 | 6 | 32635998 | T | G | 0.202783 | 0.401628 | 0.0543229 | 1.43E-13 | 0.252006 | 0.0373502 | 1.51E-11 | 1.59372 | 0.319783 | 6.24E-07 | 0.2730749 | 17 | 0.003653045 |
| cg18093866 | 6 | DDR1 | 30851753 | ENSG00000196301 | 6 | HLA-DRB9 | 32427598 | rs9274623 | 6 | 32635998 | T | G | 0.202783 | 0.401628 | 0.0543229 | 1.43E-13 | 0.305175 | 0.0378208 | 7.09E-16 | 1.31606 | 0.241429 | 5.01E-08 | 0.1249877 | 19 | 0.000293223 |
| cg10158679 | 6 | VARS2 | 30883074 | ENSG00000196301 | 6 | HLA-DRB9 | 32427598 | rs521977 | 6 | 31836827 | T | G | 0.232604 | -0.262999 | 0.0499639 | 1.41E-07 | 0.304553 | 0.0336521 | 1.43E-19 | -0.863557 | 0.189788 | 5.36E-06 | 0.2078374 | 7 | 0.031409319 |
| cg05545351 | 6 | PSORS1C1 | 31082835 | ENSG00000196301 | 6 | HLA-DRB9 | 32427598 | rs386480 | 6 | 31946837 | C | G | 0.215706 | -0.299372 | 0.0485926 | 7.24E-10 | -0.352969 | 0.0347339 | 2.93E-24 | 0.848154 | 0.160992 | 1.38E-07 | 0.07804127 | 20 | 0.000806658 |
| cg13808979 | 6 | PSORS1C1 | 31093153 | ENSG00000196301 | 6 | HLA-DRB9 | 32427598 | rs386480 | 6 | 31946837 | C | G | 0.215706 | -0.299372 | 0.0485926 | 7.24E-10 | -0.471151 | 0.0335984 | 1.13E-44 | 0.635406 | 0.112651 | 1.70E-08 | 0.07816975 | 20 | 9.93E-05 |
| cg27594095 | 6 | PSORS1C1 | 31095276 | ENSG00000196301 | 6 | HLA-DRB9 | 32427598 | rs9274514 | 6 | 32634243 | A | G | 0.191849 | 0.401128 | 0.0536355 | 7.50E-14 | 0.255933 | 0.0378591 | 1.38E-11 | 1.56732 | 0.312525 | 5.30E-07 | 0.3208169 | 17 | 0.003106541 |
| cg02357491 | 6 | PSORS1C1 | 31105744 | ENSG00000196301 | 6 | HLA-DRB9 | 32427598 | rs3117572 | 6 | 31717692 | A | G | 0.156064 | -0.462895 | 0.0573917 | 7.29E-16 | 0.221482 | 0.0404839 | 4.48E-08 | -2.08999 | 0.461613 | 5.97E-06 | 0.2163726 | 4 | 0.03495058 |
| cg04099091 | 6 | POU5F1 | 31138947 | ENSG00000196301 | 6 | HLA-DRB9 | 32427598 | rs521977 | 6 | 31836827 | T | G | 0.232604 | -0.262999 | 0.0499639 | 1.41E-07 | 0.283148 | 0.0332769 | 1.76E-17 | -0.928839 | 0.207494 | 7.59E-06 | 0.1637686 | 4 | 0.044457996 |
| cg24005169 | 6 | PSORS1C3 | 31146942 | ENSG00000196301 | 6 | HLA-DRB9 | 32427598 | rs3130342 | 6 | 32080146 | A | C | 0.107356 | -0.532201 | 0.062768 | 2.27E-17 | 0.246084 | 0.0439452 | 2.15E-08 | -2.16268 | 0.462834 | 2.97E-06 | 0.3187034 | 20 | 0.017414703 |
| cg05619024 | 6 | HLA-C | 31239411 | ENSG00000196301 | 6 | HLA-DRB9 | 32427598 | rs386480 | 6 | 31946837 | C | G | 0.215706 | -0.299372 | 0.0485926 | 7.24E-10 | 0.262881 | 0.0347069 | 3.61E-14 | -1.13881 | 0.238273 | 1.76E-06 | 0.5135853 | 8 | 0.010296905 |
| cg18785300 | 6 | HLA-C | 31240047 | ENSG00000196301 | 6 | HLA-DRB9 | 32427598 | rs386480 | 6 | 31946837 | C | G | 0.215706 | -0.299372 | 0.0485926 | 7.24E-10 | -0.225664 | 0.0336351 | 1.96E-11 | 1.32663 | 0.292346 | 5.68E-06 | 0.178662 | 13 | 0.033288079 |
| cg25843003 | 6 | HCP5 | 31431312 | ENSG00000196301 | 6 | HLA-DRB9 | 32427598 | rs3891175 | 6 | 32634467 | T | C | 0.170974 | 0.39367 | 0.0537619 | 2.43E-13 | 0.234762 | 0.0375559 | 4.08E-10 | 1.67689 | 0.352713 | 1.99E-06 | 0.9617497 | 5 | 0.011667771 |
| cg00218406 | 6 | HCP5 | 31431407 | ENSG00000196301 | 6 | HLA-DRB9 | 32427598 | rs9274623 | 6 | 32635998 | T | G | 0.202783 | 0.401628 | 0.0543229 | 1.43E-13 | 0.268739 | 0.0375026 | 7.73E-13 | 1.49449 | 0.290442 | 2.67E-07 | 0.8735607 | 5 | 0.0015623 |
| cg18808777 | 6 | HCP5 | 31431503 | ENSG00000196301 | 6 | HLA-DRB9 | 32427598 | rs9274623 | 6 | 32635998 | T | G | 0.202783 | 0.401628 | 0.0543229 | 1.43E-13 | 0.235524 | 0.0378379 | 4.83E-10 | 1.70525 | 0.35812 | 1.92E-06 | 0.5064939 | 5 | 0.011246692 |
| cg02248037 | 6 | MICB | 31465876 | ENSG00000196301 | 6 | HLA-DRB9 | 32427598 | rs9274623 | 6 | 32635998 | T | G | 0.202783 | 0.401628 | 0.0543229 | 1.43E-13 | -0.23051 | 0.0369923 | 4.63E-10 | -1.74235 | 0.365678 | 1.89E-06 | 0.8031907 | 20 | 0.011079429 |
| cg04219977 | 6 | BAT1 | 31506768 | ENSG00000196301 | 6 | HLA-DRB9 | 32427598 | rs2227956 | 6 | 31778272 | G | A | 0.157058 | -0.471657 | 0.057635 | 2.76E-16 | 0.263065 | 0.0408469 | 1.19E-10 | -1.79293 | 0.354265 | 4.17E-07 | 0.1317932 | 15 | 0.002443364 |
| cg00853714 | 6 | BAT1 | 31510077 | ENSG00000196301 | 6 | HLA-DRB9 | 32427598 | rs2523504 | 6 | 31510858 | T | C | 0.274354 | -0.303156 | 0.049662 | 1.03E-09 | 0.237659 | 0.0363753 | 6.42E-11 | -1.27559 | 0.285978 | 8.18E-06 | 0.3159242 | 6 | 0.047910719 |
| cg25420482 | 6 | NFKBIL1 | 31519182 | ENSG00000196301 | 6 | HLA-DRB9 | 32427598 | rs9274623 | 6 | 32635998 | T | G | 0.202783 | 0.401628 | 0.0543229 | 1.43E-13 | 0.248143 | 0.0377295 | 4.80E-11 | 1.61853 | 0.329374 | 8.92E-07 | 0.1840117 | 7 | 0.005228203 |
| cg26333342 | 6 | NFKBIL1 | 31519394 | ENSG00000196301 | 6 | HLA-DRB9 | 32427598 | rs9274623 | 6 | 32635998 | T | G | 0.202783 | 0.401628 | 0.0543229 | 1.43E-13 | 0.246366 | 0.0379918 | 8.89E-11 | 1.63021 | 0.33439 | 1.09E-06 | 0.182007 | 5 | 0.006368613 |
| cg19563932 | 6 | AIF1 | 31583915 | ENSG00000196301 | 6 | HLA-DRB9 | 32427598 | rs1048709 | 6 | 31914935 | A | G | 0.152087 | -0.473411 | 0.0549177 | 6.67E-18 | 0.260978 | 0.0395198 | 4.01E-11 | -1.81399 | 0.346029 | 1.59E-07 | 0.08396492 | 6 | 0.000929016 |
| cg12608306 | 6 | BAT4 | 31632740 | ENSG00000196301 | 6 | HLA-DRB9 | 32427598 | rs9270980 | 6 | 32573909 | C | A | 0.137177 | -0.602789 | 0.0575794 | 1.20E-25 | 0.273005 | 0.0415586 | 5.06E-11 | -2.20798 | 0.396806 | 2.63E-08 | 0.1492345 | 20 | 0.000154115 |
| cg12951348 | 6 | LY6G6D | 31685430 | ENSG00000196301 | 6 | HLA-DRB9 | 32427598 | rs3117572 | 6 | 31717692 | A | G | 0.156064 | -0.462895 | 0.0573917 | 7.29E-16 | -0.229045 | 0.0409795 | 2.28E-08 | 2.02098 | 0.439917 | 4.35E-06 | 0.07875373 | 3 | 0.025472593 |
| cg00406022 | 6 | C6orf25 | 31691696 | ENSG00000196301 | 6 | HLA-DRB9 | 32427598 | rs9267576 | 6 | 31812038 | T | G | 0.107356 | -0.50138 | 0.0641271 | 5.34E-15 | 0.247192 | 0.044935 | 3.77E-08 | -2.0283 | 0.450828 | 6.83E-06 | 0.6943518 | 20 | 0.039982736 |
| cg26749976 | 6 | C6orf25 | 31691717 | ENSG00000196301 | 6 | HLA-DRB9 | 32427598 | rs9274537 | 6 | 32634655 | G | A | 0.473161 | -0.339976 | 0.0428364 | 2.08E-15 | 0.187698 | 0.0327669 | 1.01E-08 | -1.81129 | 0.389959 | 3.40E-06 | 0.4446596 | 20 | 0.019937949 |
| cg11261908 | 6 | DDAH2 | 31697955 | ENSG00000196301 | 6 | HLA-DRB9 | 32427598 | rs9267658 | 6 | 31845985 | T | C | 0.104374 | -0.511585 | 0.0644843 | 2.13E-15 | 0.248308 | 0.0431906 | 8.97E-09 | -2.06028 | 0.442569 | 3.24E-06 | 0.05043848 | 20 | 0.018952469 |
| cg10709727 | 6 | DDAH2 | 31698145 | ENSG00000196301 | 6 | HLA-DRB9 | 32427598 | rs9274623 | 6 | 32635998 | T | G | 0.202783 | 0.401628 | 0.0543229 | 1.43E-13 | 0.52874 | 0.0364276 | 9.76E-48 | 0.759595 | 0.115301 | 4.46E-11 | 0.1077815 | 20 | 2.61E-07 |
| cg00124375 | 6 | DDAH2 | 31698218 | ENSG00000196301 | 6 | HLA-DRB9 | 32427598 | rs3117572 | 6 | 31717692 | A | G | 0.156064 | -0.462895 | 0.0573917 | 7.29E-16 | -0.258694 | 0.0407415 | 2.16E-10 | 1.78935 | 0.358652 | 6.07E-07 | 0.05992552 | 6 | 0.003553427 |
| cg00657529 | 6 | CLIC1 | 31698687 | ENSG00000196301 | 6 | HLA-DRB9 | 32427598 | rs9274623 | 6 | 32635998 | T | G | 0.202783 | 0.401628 | 0.0543229 | 1.43E-13 | -0.342269 | 0.0371387 | 3.08E-20 | -1.17343 | 0.203475 | 8.07E-09 | 0.1193235 | 20 | 4.73E-05 |
| cg04387059 | 6 | CLIC1 | 31698722 | ENSG00000196301 | 6 | HLA-DRB9 | 32427598 | rs3891175 | 6 | 32634467 | T | C | 0.170974 | 0.39367 | 0.0537619 | 2.43E-13 | -0.232647 | 0.037837 | 7.81E-10 | -1.69213 | 0.359359 | 2.49E-06 | 0.4999003 | 20 | 0.014599852 |
| cg20640261 | 6 | MSH5 | 31707019 | ENSG00000196301 | 6 | HLA-DRB9 | 32427598 | rs9267659 | 6 | 31846234 | A | G | 0.191849 | -0.473981 | 0.053227 | 5.34E-19 | 0.31905 | 0.0381599 | 6.23E-17 | -1.4856 | 0.24373 | 1.09E-09 | 0.08457795 | 20 | 6.40E-06 |
| cg25641533 | 6 | C6orf27 | 31734580 | ENSG00000196301 | 6 | HLA-DRB9 | 32427598 | rs9274623 | 6 | 32635998 | T | G | 0.202783 | 0.401628 | 0.0543229 | 1.43E-13 | -0.215019 | 0.0377719 | 1.25E-08 | -1.86787 | 0.414118 | 6.47E-06 | 0.2547561 | 12 | 0.03789037 |
| cg03877020 | 6 | VARS | 31762680 | ENSG00000196301 | 6 | HLA-DRB9 | 32427598 | rs3117572 | 6 | 31717692 | A | G | 0.156064 | -0.462895 | 0.0573917 | 7.29E-16 | 0.244482 | 0.0406484 | 1.80E-09 | -1.89337 | 0.392689 | 1.42E-06 | 0.1845634 | 12 | 0.008344393 |
| cg15665090 | 6 | HSPA1L | 31778371 | ENSG00000196301 | 6 | HLA-DRB9 | 32427598 | rs2227956 | 6 | 31778272 | G | A | 0.157058 | -0.471657 | 0.057635 | 2.76E-16 | 0.267455 | 0.0400837 | 2.52E-11 | -1.7635 | 0.341014 | 2.32E-07 | 0.2038311 | 7 | 0.001361541 |
| cg16372051 | 6 | HSPA1B | 31794302 | ENSG00000196301 | 6 | HLA-DRB9 | 32427598 | rs9274623 | 6 | 32635998 | T | G | 0.202783 | 0.401628 | 0.0543229 | 1.43E-13 | 0.223822 | 0.0378496 | 3.35E-09 | 1.79441 | 0.388568 | 3.87E-06 | 0.4324328 | 10 | 0.022696874 |
| cg24508713 | 6 | ZBTB12 | 31870783 | ENSG00000196301 | 6 | HLA-DRB9 | 32427598 | rs9274497 | 6 | 32633928 | A | G | 0.183897 | 0.410636 | 0.053744 | 2.16E-14 | -0.243996 | 0.0382059 | 1.70E-10 | -1.68296 | 0.343457 | 9.58E-07 | 0.07322991 | 20 | 0.005612215 |
| cg17810099 | 6 | ZBTB12 | 31870990 | ENSG00000196301 | 6 | HLA-DRB9 | 32427598 | rs79638487 | 6 | 32626664 | A | C | 0.0984095 | 0.51649 | 0.0741686 | 3.31E-12 | -0.283639 | 0.0485853 | 5.28E-09 | -1.82094 | 0.407022 | 7.68E-06 | 0.1211502 | 13 | 0.045011396 |
| cg22575347 | 6 | CYP21A2 | 31973180 | ENSG00000196301 | 6 | HLA-DRB9 | 32427598 | rs28707527 | 6 | 32587717 | A | G | 0.205765 | -0.457459 | 0.0575496 | 1.88E-15 | -0.233863 | 0.0418961 | 2.38E-08 | 1.9561 | 0.428204 | 4.92E-06 | 0.1269377 | 14 | 0.028824517 |
| cg05045332 | 6 | TNXB | 32012897 | ENSG00000196301 | 6 | HLA-DRB9 | 32427598 | rs386480 | 6 | 31946837 | C | G | 0.215706 | -0.299372 | 0.0485926 | 7.24E-10 | 0.397919 | 0.0346461 | 1.56E-30 | -0.752344 | 0.138576 | 5.66E-08 | 0.09641753 | 20 | 0.000331811 |
| cg16225663 | 6 | TNXB | 32026797 | ENSG00000196301 | 6 | HLA-DRB9 | 32427598 | rs1009382 | 6 | 32026107 | C | T | 0.281312 | -0.295985 | 0.0445305 | 3.00E-11 | -0.234952 | 0.0353509 | 3.01E-11 | 1.25977 | 0.268046 | 2.60E-06 | 0.05053178 | 20 | 0.015254045 |
| cg01188191 | 6 | TNXB | 32047349 | ENSG00000196301 | 6 | HLA-DRB9 | 32427598 | rs78654105 | 6 | 32510536 | C | T | 0.0715706 | 0.619578 | 0.098083 | 2.67E-10 | 0.404267 | 0.0554813 | 3.18E-13 | 1.5326 | 0.321098 | 1.82E-06 | 0.2212289 | 17 | 0.010632926 |
| cg08362880 | 6 | TNXB | 32053600 | ENSG00000196301 | 6 | HLA-DRB9 | 32427598 | rs1009382 | 6 | 32026107 | C | T | 0.281312 | -0.295985 | 0.0445305 | 3.00E-11 | -0.221735 | 0.0357116 | 5.33E-10 | 1.33486 | 0.294195 | 5.70E-06 | 0.4364512 | 7 | 0.033373524 |
| cg20471413 | 6 | TNXB | 32055135 | ENSG00000196301 | 6 | HLA-DRB9 | 32427598 | rs9274574 | 6 | 32635219 | A | G | 0.347913 | 0.373726 | 0.0459034 | 3.90E-16 | -0.236018 | 0.0340114 | 3.94E-12 | -1.58346 | 0.299825 | 1.28E-07 | 0.1335206 | 20 | 0.000751443 |
| cg24055029 | 6 | TNXB | 32055137 | ENSG00000196301 | 6 | HLA-DRB9 | 32427598 | rs9274574 | 6 | 32635219 | A | G | 0.347913 | 0.373726 | 0.0459034 | 3.90E-16 | -0.21281 | 0.0343498 | 5.81E-10 | -1.75615 | 0.356198 | 8.21E-07 | 0.1597435 | 20 | 0.00481061 |
| cg10812186 | 6 | TNXB | 32055146 | ENSG00000196301 | 6 | HLA-DRB9 | 32427598 | rs9270911 | 6 | 32572202 | C | T | 0.463221 | -0.447234 | 0.0427146 | 1.18E-25 | 0.207062 | 0.0327308 | 2.51E-10 | -2.1599 | 0.398903 | 6.14E-08 | 0.07282674 | 20 | 0.000359808 |
| cg10923662 | 6 | TNXB | 32064258 | ENSG00000196301 | 6 | HLA-DRB9 | 32427598 | rs389883 | 6 | 31947460 | G | T | 0.215706 | -0.299372 | 0.0485926 | 7.24E-10 | -0.2886 | 0.0347831 | 1.07E-16 | 1.03733 | 0.209714 | 7.56E-07 | 0.08934383 | 20 | 0.004429261 |
| cg03556669 | 6 | TNXB | 32064497 | ENSG00000196301 | 6 | HLA-DRB9 | 32427598 | rs386480 | 6 | 31946837 | C | G | 0.215706 | -0.299372 | 0.0485926 | 7.24E-10 | -0.268892 | 0.0349435 | 1.41E-14 | 1.11335 | 0.231498 | 1.51E-06 | 0.2042135 | 20 | 0.008870307 |
| cg24882324 | 6 | TNXB | 32064508 | ENSG00000196301 | 6 | HLA-DRB9 | 32427598 | rs386480 | 6 | 31946837 | C | G | 0.215706 | -0.299372 | 0.0485926 | 7.24E-10 | -0.261521 | 0.0348422 | 6.10E-14 | 1.14473 | 0.240384 | 1.92E-06 | 0.2061263 | 20 | 0.011222616 |
| cg15196197 | 6 | TNXB | 32064573 | ENSG00000196301 | 6 | HLA-DRB9 | 32427598 | rs389883 | 6 | 31947460 | G | T | 0.215706 | -0.299372 | 0.0485926 | 7.24E-10 | -0.277881 | 0.0346823 | 1.13E-15 | 1.07734 | 0.220588 | 1.04E-06 | 0.1014664 | 20 | 0.006091529 |
| cg13400512 | 6 | TNXB | 32064578 | ENSG00000196301 | 6 | HLA-DRB9 | 32427598 | rs389883 | 6 | 31947460 | G | T | 0.215706 | -0.299372 | 0.0485926 | 7.24E-10 | -0.28821 | 0.0343964 | 5.33E-17 | 1.03873 | 0.209271 | 6.92E-07 | 0.1244509 | 20 | 0.004054356 |
| cg12694372 | 6 | TNXB | 32064582 | ENSG00000196301 | 6 | HLA-DRB9 | 32427598 | rs389883 | 6 | 31947460 | G | T | 0.215706 | -0.299372 | 0.0485926 | 7.24E-10 | -0.292215 | 0.0347342 | 4.00E-17 | 1.02449 | 0.206112 | 6.68E-07 | 0.09946712 | 20 | 0.003910623 |
| cg15265085 | 6 | TNXB | 32064588 | ENSG00000196301 | 6 | HLA-DRB9 | 32427598 | rs389883 | 6 | 31947460 | G | T | 0.215706 | -0.299372 | 0.0485926 | 7.24E-10 | -0.29531 | 0.0347293 | 1.84E-17 | 1.01376 | 0.203198 | 6.07E-07 | 0.07893315 | 20 | 0.003555445 |
| cg21642103 | 6 | TNXB | 32064656 | ENSG00000196301 | 6 | HLA-DRB9 | 32427598 | rs389883 | 6 | 31947460 | G | T | 0.215706 | -0.299372 | 0.0485926 | 7.24E-10 | -0.288404 | 0.0348082 | 1.18E-16 | 1.03803 | 0.209962 | 7.66E-07 | 0.1134817 | 20 | 0.004485618 |
| cg21342636 | 6 | TNXB | 32064671 | ENSG00000196301 | 6 | HLA-DRB9 | 32427598 | rs389883 | 6 | 31947460 | G | T | 0.215706 | -0.299372 | 0.0485926 | 7.24E-10 | -0.284116 | 0.03469 | 2.61E-16 | 1.0537 | 0.214018 | 8.50E-07 | 0.1548266 | 20 | 0.004982128 |
| cg01485117 | 6 | TNXB | 32064677 | ENSG00000196301 | 6 | HLA-DRB9 | 32427598 | rs389883 | 6 | 31947460 | G | T | 0.215706 | -0.299372 | 0.0485926 | 7.24E-10 | -0.270587 | 0.0346612 | 5.87E-15 | 1.10638 | 0.228769 | 1.32E-06 | 0.09908898 | 20 | 0.007751434 |
| cg15014577 | 6 | TNXB | 32064785 | ENSG00000196301 | 6 | HLA-DRB9 | 32427598 | rs386480 | 6 | 31946837 | C | G | 0.215706 | -0.299372 | 0.0485926 | 7.24E-10 | -0.30337 | 0.0348646 | 3.28E-18 | 0.986821 | 0.19626 | 4.95E-07 | 0.08368946 | 20 | 0.002901495 |
| cg07518714 | 6 | TNXB | 32064945 | ENSG00000196301 | 6 | HLA-DRB9 | 32427598 | rs389883 | 6 | 31947460 | G | T | 0.215706 | -0.299372 | 0.0485926 | 7.24E-10 | -0.316973 | 0.0349762 | 1.27E-19 | 0.944472 | 0.185372 | 3.49E-07 | 0.06162412 | 20 | 0.002042805 |
| cg17066403 | 6 | TNXB | 32065028 | ENSG00000196301 | 6 | HLA-DRB9 | 32427598 | rs9274569 | 6 | 32635092 | A | C | 0.310139 | 0.373726 | 0.0459034 | 3.90E-16 | -0.199764 | 0.0342236 | 5.31E-09 | -1.87084 | 0.394374 | 2.10E-06 | 0.5514822 | 15 | 0.012285948 |
| cg04037640 | 6 | TNXB | 32065034 | ENSG00000196301 | 6 | HLA-DRB9 | 32427598 | rs389883 | 6 | 31947460 | G | T | 0.215706 | -0.299372 | 0.0485926 | 7.24E-10 | -0.237407 | 0.0353665 | 1.91E-11 | 1.26101 | 0.277818 | 5.65E-06 | 0.2518192 | 20 | 0.033114266 |
| cg19108771 | 6 | TNXB | 32076337 | ENSG00000196301 | 6 | HLA-DRB9 | 32427598 | rs389883 | 6 | 31947460 | G | T | 0.215706 | -0.299372 | 0.0485926 | 7.24E-10 | -0.313538 | 0.0344241 | 8.38E-20 | 0.954819 | 0.187107 | 3.34E-07 | 0.06148447 | 20 | 0.001957703 |
| cg21448535 | 6 | TNXB | 32076533 | ENSG00000196301 | 6 | HLA-DRB9 | 32427598 | rs389883 | 6 | 31947460 | G | T | 0.215706 | -0.299372 | 0.0485926 | 7.24E-10 | -0.235582 | 0.0343723 | 7.19E-12 | 1.27078 | 0.27735 | 4.61E-06 | 0.09480262 | 20 | 0.026997823 |
| cg11606877 | 6 | TNXB | 32076592 | ENSG00000196301 | 6 | HLA-DRB9 | 32427598 | rs386480 | 6 | 31946837 | C | G | 0.215706 | -0.299372 | 0.0485926 | 7.24E-10 | -0.251452 | 0.0352126 | 9.27E-13 | 1.19057 | 0.255229 | 3.09E-06 | 0.06088901 | 20 | 0.018102702 |
| cg02925367 | 6 | PRRT1 | 32116905 | ENSG00000196301 | 6 | HLA-DRB9 | 32427598 | rs9267654 | 6 | 31841009 | A | G | 0.158052 | -0.449566 | 0.0577073 | 6.68E-15 | 0.217919 | 0.0398711 | 4.61E-08 | -2.063 | 0.46108 | 7.67E-06 | 0.08338866 | 15 | 0.044914282 |
| cg08424749 | 6 | PRRT1 | 32117292 | ENSG00000196301 | 6 | HLA-DRB9 | 32427598 | rs3130342 | 6 | 32080146 | A | C | 0.107356 | -0.532201 | 0.062768 | 2.27E-17 | 0.247302 | 0.0451922 | 4.44E-08 | -2.15203 | 0.468056 | 4.27E-06 | 0.06365196 | 13 | 0.025012149 |
| cg17218813 | 6 | PRRT1 | 32119616 | ENSG00000196301 | 6 | HLA-DRB9 | 32427598 | rs589428 | 6 | 31848220 | T | G | 0.27833 | -0.264915 | 0.0465199 | 1.24E-08 | -0.336797 | 0.0330863 | 2.45E-24 | 0.786572 | 0.15827 | 6.70E-07 | 0.09689421 | 6 | 0.003925661 |
| cg12602633 | 6 | PRRT1 | 32119691 | ENSG00000196301 | 6 | HLA-DRB9 | 32427598 | rs589428 | 6 | 31848220 | T | G | 0.27833 | -0.264915 | 0.0465199 | 1.24E-08 | -0.267776 | 0.0332085 | 7.42E-16 | 0.989316 | 0.212683 | 3.29E-06 | 0.1995711 | 5 | 0.019295057 |
| cg12883279 | 6 | PPT2 | 32120773 | ENSG00000196301 | 6 | HLA-DRB9 | 32427598 | rs113972770 | 6 | 32501169 | C | T | 0.143141 | -0.590716 | 0.0539368 | 6.50E-28 | 0.236274 | 0.0413194 | 1.08E-08 | -2.50013 | 0.493229 | 4.00E-07 | 0.5267376 | 20 | 0.002343793 |
| cg17229678 | 6 | PPT2 | 32121555 | ENSG00000196301 | 6 | HLA-DRB9 | 32427598 | rs3891175 | 6 | 32634467 | T | C | 0.170974 | 0.39367 | 0.0537619 | 2.43E-13 | -0.219801 | 0.037503 | 4.60E-09 | -1.79103 | 0.391422 | 4.75E-06 | 0.9547235 | 5 | 0.027803738 |
| cg00963496 | 6 | PBX2 | 32154023 | ENSG00000196301 | 6 | HLA-DRB9 | 32427598 | rs9274498 | 6 | 32633941 | G | A | 0.187873 | 0.373334 | 0.0525807 | 1.25E-12 | 0.216272 | 0.0371156 | 5.64E-09 | 1.72622 | 0.383237 | 6.66E-06 | 0.3270253 | 9 | 0.039005587 |
| cg20808711 | 6 | GPSM3 | 32160998 | ENSG00000196301 | 6 | HLA-DRB9 | 32427598 | rs9271702 | 6 | 32593220 | T | G | 0.210736 | -0.560838 | 0.0491381 | 3.58E-30 | -0.230625 | 0.0410369 | 1.91E-08 | 2.43182 | 0.482324 | 4.61E-07 | 0.1174972 | 18 | 0.002700443 |
| cg20116128 | 6 | GPSM3 | 32161004 | ENSG00000196301 | 6 | HLA-DRB9 | 32427598 | rs9271683 | 6 | 32592950 | C | T | 0.218688 | -0.560838 | 0.0491381 | 3.58E-30 | -0.241717 | 0.0409133 | 3.46E-09 | 2.32023 | 0.44222 | 1.55E-07 | 0.141506 | 18 | 0.000906752 |
| cg24856537 | 6 | NOTCH4 | 32169929 | ENSG00000196301 | 6 | HLA-DRB9 | 32427598 | rs9271775 | 6 | 32594328 | C | T | 0.181909 | -0.529872 | 0.0501057 | 3.89E-26 | 0.226967 | 0.0400976 | 1.51E-08 | -2.33458 | 0.467809 | 6.02E-07 | 0.1498149 | 20 | 0.00352874 |
| cg24697648 | 6 | NOTCH4 | 32188317 | ENSG00000196301 | 6 | HLA-DRB9 | 32427598 | rs386480 | 6 | 31946837 | C | G | 0.215706 | -0.299372 | 0.0485926 | 7.24E-10 | 0.240072 | 0.0350979 | 7.92E-12 | -1.24701 | 0.272408 | 4.70E-06 | 0.2512847 | 9 | 0.027535383 |
| cg10158182 | 6 | NOTCH4 | 32188822 | ENSG00000196301 | 6 | HLA-DRB9 | 32427598 | rs386480 | 6 | 31946837 | C | G | 0.215706 | -0.299372 | 0.0485926 | 7.24E-10 | 0.249701 | 0.0347643 | 6.84E-13 | -1.19892 | 0.256383 | 2.92E-06 | 0.06934854 | 14 | 0.017111341 |
| cg06884718 | 6 | NOTCH4 | 32191821 | ENSG00000196301 | 6 | HLA-DRB9 | 32427598 | rs9274505 | 6 | 32634126 | A | G | 0.137177 | 0.395646 | 0.0537506 | 1.83E-13 | -0.314842 | 0.038474 | 2.76E-16 | -1.25665 | 0.229626 | 4.43E-08 | 0.1318457 | 20 | 0.000259795 |
| cg11075234 | 6 | C6orf10 | 32312513 | ENSG00000196301 | 6 | HLA-DRB9 | 32427598 | rs9271085 | 6 | 32576152 | T | C | 0.143141 | -0.602789 | 0.0575794 | 1.20E-25 | 0.26094 | 0.0414693 | 3.13E-10 | -2.31007 | 0.428334 | 6.92E-08 | 0.06422395 | 20 | 0.000405585 |
| cg21195277 | 6 | C6orf10 | 32339208 | ENSG00000196301 | 6 | HLA-DRB9 | 32427598 | rs78654105 | 6 | 32510536 | C | T | 0.0715706 | 0.619578 | 0.098083 | 2.67E-10 | 0.389856 | 0.0552116 | 1.65E-12 | 1.58925 | 0.337569 | 2.50E-06 | 0.583745 | 20 | 0.014660032 |
| cg04970287 | 6 | BTNL2 | 32362638 | ENSG00000196301 | 6 | HLA-DRB9 | 32427598 | rs112627226 | 6 | 32582969 | C | T | 0.115308 | -0.333947 | 0.0736654 | 5.81E-06 | -1.6164 | 0.0434841 | 1.92E-302 | 0.206599 | 0.0459114 | 6.80E-06 | 0.2482846 | 20 | 0.039815115 |
| cg03036047 | 6 | BTNL2 | 32362744 | ENSG00000196301 | 6 | HLA-DRB9 | 32427598 | rs112627226 | 6 | 32582969 | C | T | 0.115308 | -0.333947 | 0.0736654 | 5.81E-06 | -1.36815 | 0.0482977 | 1.59E-176 | 0.244087 | 0.0545282 | 7.59E-06 | 0.2465746 | 20 | 0.04448195 |
| cg19117063 | 6 | BTNL2 | 32365553 | ENSG00000196301 | 6 | HLA-DRB9 | 32427598 | rs112627226 | 6 | 32582969 | C | T | 0.115308 | -0.333947 | 0.0736654 | 5.81E-06 | -1.74745 | 0.0411387 | 0 | 0.191105 | 0.0423953 | 6.55E-06 | 0.2784459 | 20 | 0.03839011 |
| cg03100814 | 6 | BTNL2 | 32367672 | ENSG00000196301 | 6 | HLA-DRB9 | 32427598 | rs9271718 | 6 | 32593465 | T | A | 0.365805 | 0.298033 | 0.0446832 | 2.56E-11 | 0.211332 | 0.0332881 | 2.17E-10 | 1.41026 | 0.306677 | 4.25E-06 | 0.05493596 | 20 | 0.024925726 |
| cg14241129 | 6 | BTNL2 | 32367729 | ENSG00000196301 | 6 | HLA-DRB9 | 32427598 | rs9273040 | 6 | 32611958 | G | C | 0.276342 | 0.354665 | 0.0460731 | 1.38E-14 | 0.363591 | 0.033706 | 3.96E-27 | 0.975451 | 0.155673 | 3.70E-10 | 0.8088149 | 20 | 2.17E-06 |
| cg03515726 | 6 | BTNL2 | 32367750 | ENSG00000196301 | 6 | HLA-DRB9 | 32427598 | rs28366340 | 6 | 32564727 | G | A | 0.27336 | 0.343472 | 0.0420608 | 3.19E-16 | 0.459675 | 0.0324922 | 1.94E-45 | 0.747206 | 0.105651 | 1.52E-12 | 0.5215731 | 20 | 8.92E-09 |
| cg24911364 | 6 | BTNL2 | 32372236 | ENSG00000196301 | 6 | HLA-DRB9 | 32427598 | rs28707527 | 6 | 32587717 | A | G | 0.205765 | -0.457459 | 0.0575496 | 1.88E-15 | 0.355973 | 0.0470288 | 3.75E-14 | -1.28509 | 0.234438 | 4.22E-08 | 0.1814437 | 20 | 0.000246926 |
| cg00978102 | 6 | BTNL2 | 32375231 | ENSG00000196301 | 6 | HLA-DRB9 | 32427598 | rs9271383 | 6 | 32587391 | C | T | 0.481113 | -0.425554 | 0.0427168 | 2.23E-23 | 0.187657 | 0.0323541 | 6.63E-09 | -2.26772 | 0.452417 | 5.37E-07 | 0.05787197 | 20 | 0.003148077 |
| cg18067840 | 6 | BTNL2 | 32375672 | ENSG00000196301 | 6 | HLA-DRB9 | 32427598 | rs2454138 | 6 | 32570401 | A | G | 0.267396 | 0.339329 | 0.0467705 | 4.01E-13 | 0.541742 | 0.0328854 | 5.68E-61 | 0.626366 | 0.0943355 | 3.14E-11 | 0.242159 | 20 | 1.84E-07 |
| cg00383136 | 6 | HLA-DRA | 32410247 | ENSG00000196301 | 6 | HLA-DRB9 | 32427598 | rs78654105 | 6 | 32510536 | C | T | 0.0715706 | 0.619578 | 0.098083 | 2.67E-10 | -0.36174 | 0.0557964 | 8.98E-11 | -1.71277 | 0.378566 | 6.06E-06 | 0.242458 | 11 | 0.035485362 |
| cg08882389 | 6 | HLA-DRA | 32410690 | ENSG00000196301 | 6 | HLA-DRB9 | 32427598 | rs389883 | 6 | 31947460 | G | T | 0.215706 | -0.299372 | 0.0485926 | 7.24E-10 | -0.295589 | 0.0341428 | 4.82E-18 | 1.0128 | 0.201769 | 5.18E-07 | 0.08354767 | 20 | 0.003033176 |
| cg10466124 | 6 | HLA-DRB5 | 32498285 | ENSG00000196301 | 6 | HLA-DRB9 | 32427598 | rs9271464 | 6 | 32588544 | T | A | 0.284294 | 0.348538 | 0.0460915 | 3.97E-14 | -0.682594 | 0.0315205 | 5.39E-104 | -0.510608 | 0.0715223 | 9.39E-13 | 0.133434 | 20 | 5.50E-09 |
| cg09663314 | 6 | HLA-DRB6 | 32525979 | ENSG00000196301 | 6 | HLA-DRB9 | 32427598 | rs9271435 | 6 | 32588069 | T | C | 0.387674 | 0.34202 | 0.0412006 | 1.03E-16 | -0.276741 | 0.0331179 | 6.48E-17 | -1.23588 | 0.209855 | 3.88E-09 | 0.4088505 | 20 | 2.27E-05 |
| cg06204447 | 6 | HLA-DRB1 | 32546665 | ENSG00000196301 | 6 | HLA-DRB9 | 32427598 | rs9271464 | 6 | 32588544 | T | A | 0.284294 | 0.348538 | 0.0460915 | 3.97E-14 | -1.04665 | 0.0271458 | 0 | -0.333003 | 0.0448761 | 1.17E-13 | 0.304338 | 20 | 6.83E-10 |
| cg13778567 | 6 | HLA-DQA1 | 32609783 | ENSG00000196301 | 6 | HLA-DRB9 | 32427598 | rs28366319 | 6 | 32561495 | A | G | 0.300199 | 0.36255 | 0.0465074 | 6.41E-15 | 1.09606 | 0.0248368 | 0 | 0.330776 | 0.0430883 | 1.63E-14 | 0.1189628 | 20 | 9.56E-11 |
| cg10180404 | 6 | HLA-DQB1 | 32632334 | ENSG00000196301 | 6 | HLA-DRB9 | 32427598 | rs9274498 | 6 | 32633941 | G | A | 0.187873 | 0.373334 | 0.0525807 | 1.25E-12 | -1.0635 | 0.0300529 | 2.65E-274 | -0.351043 | 0.0504266 | 3.37E-12 | 0.09421916 | 20 | 1.97E-08 |
| cg23464743 | 6 | HLA-DQB1 | 32633163 | ENSG00000196301 | 6 | HLA-DRB9 | 32427598 | rs9274617 | 6 | 32635904 | A | T | 0.379722 | 0.373726 | 0.0459034 | 3.90E-16 | 1.03933 | 0.0265367 | 0 | 0.359584 | 0.0451105 | 1.57E-15 | 0.4203026 | 20 | 9.21E-12 |
| cg11986643 | 6 | HLA-DQB1 | 32634316 | ENSG00000196301 | 6 | HLA-DRB9 | 32427598 | rs28383313 | 6 | 32587117 | G | C | 0.32008 | 0.352302 | 0.045453 | 9.12E-15 | 0.584468 | 0.0318952 | 5.26E-75 | 0.602774 | 0.0844389 | 9.43E-13 | 0.9530414 | 20 | 5.52E-09 |
| cg23336481 | 6 | HLA-DQB1 | 32634344 | ENSG00000196301 | 6 | HLA-DRB9 | 32427598 | rs9274498 | 6 | 32633941 | G | A | 0.187873 | 0.373334 | 0.0525807 | 1.25E-12 | 0.885915 | 0.0319429 | 2.69E-169 | 0.421411 | 0.061266 | 6.05E-12 | 0.06819297 | 20 | 3.55E-08 |
| cg01244342 | 6 | HLA-DQA2 | 32709470 | ENSG00000196301 | 6 | HLA-DRB9 | 32427598 | rs28707773 | 6 | 32572278 | A | C | 0.139165 | -0.347679 | 0.0667036 | 1.87E-07 | -0.466907 | 0.051379 | 1.01E-19 | 0.744643 | 0.164694 | 6.14E-06 | 0.4625615 | 20 | 0.035992208 |
| cg01050736 | 6 | HLA-DQA2 | 32710583 | ENSG00000196301 | 6 | HLA-DRB9 | 32427598 | rs116070749 | 6 | 32501088 | G | A | 0.0715706 | 0.59992 | 0.0930645 | 1.15E-10 | -0.556428 | 0.0540347 | 7.22E-25 | -1.07816 | 0.197322 | 4.66E-08 | 0.08022495 | 20 | 0.000272729 |
| cg02293354 | 6 | HLA-DQA2 | 32711008 | ENSG00000196301 | 6 | HLA-DRB9 | 32427598 | rs9271430 | 6 | 32588002 | C | T | 0.303181 | 0.351475 | 0.0459267 | 1.96E-14 | -0.242383 | 0.0335999 | 5.44E-13 | -1.45008 | 0.276242 | 1.53E-07 | 0.1123158 | 20 | 0.000894321 |
| cg14740554 | 6 | HLA-DQA2 | 32713607 | ENSG00000196301 | 6 | HLA-DRB9 | 32427598 | rs9271523 | 6 | 32589806 | G | A | 0.293241 | 0.349863 | 0.0454354 | 1.36E-14 | 0.572868 | 0.0318429 | 2.31E-72 | 0.610722 | 0.0862717 | 1.45E-12 | 0.4982648 | 20 | 8.50E-09 |
| cg22812614 | 6 | HLA-DQA2 | 32713827 | ENSG00000196301 | 6 | HLA-DRB9 | 32427598 | rs9271464 | 6 | 32588544 | T | A | 0.284294 | 0.348538 | 0.0460915 | 3.97E-14 | -1.14986 | 0.0242236 | 0 | -0.303113 | 0.0405898 | 8.16E-14 | 0.2936367 | 20 | 4.78E-10 |
| cg20985082 | 6 | HLA-DQB2 | 32727265 | ENSG00000196301 | 6 | HLA-DRB9 | 32427598 | rs9271517 | 6 | 32589740 | G | A | 0.279324 | 0.356022 | 0.0458438 | 8.10E-15 | -0.248133 | 0.033876 | 2.39E-13 | -1.4348 | 0.269268 | 9.90E-08 | 0.3324451 | 20 | 0.000579996 |
| cg23418102 | 6 | HLA-DQB2 | 32729847 | ENSG00000196301 | 6 | HLA-DRB9 | 32427598 | rs9271683 | 6 | 32592950 | C | T | 0.218688 | -0.560838 | 0.0491381 | 3.58E-30 | 0.307965 | 0.0402517 | 1.99E-14 | -1.82111 | 0.286555 | 2.08E-10 | 0.05440804 | 20 | 1.22E-06 |
| cg02010152 | 6 | HLA-DOB | 32782617 | ENSG00000196301 | 6 | HLA-DRB9 | 32427598 | rs28895049 | 6 | 32416806 | G | A | 0.178926 | -0.305449 | 0.0615794 | 7.04E-07 | 0.4965 | 0.0450862 | 3.34E-28 | -0.615204 | 0.136028 | 6.11E-06 | 0.08967588 | 20 | 0.03577903 |
| cg13563634 | 6 | TAP2 | 32805684 | ENSG00000196301 | 6 | HLA-DRB9 | 32427598 | rs1812006 | 6 | 32778656 | A | G | 0.0934394 | -0.478147 | 0.0646942 | 1.46E-13 | -0.272226 | 0.0438195 | 5.22E-10 | 1.75643 | 0.369341 | 1.98E-06 | 0.2405643 | 3 | 0.011590926 |
| cg26685246 | 6 | TAP2 | 32805692 | ENSG00000196301 | 6 | HLA-DRB9 | 32427598 | rs1812006 | 6 | 32778656 | A | G | 0.0934394 | -0.478147 | 0.0646942 | 1.46E-13 | -0.297404 | 0.044524 | 2.40E-11 | 1.60774 | 0.324426 | 7.21E-07 | 0.2187357 | 3 | 0.004223239 |
| cg08491668 | 6 | BRD2 | 32935236 | ENSG00000196301 | 6 | HLA-DRB9 | 32427598 | rs3891175 | 6 | 32634467 | T | C | 0.170974 | 0.39367 | 0.0537619 | 2.43E-13 | -0.250748 | 0.0375281 | 2.36E-11 | -1.56998 | 0.31809 | 7.99E-07 | 0.3223225 | 15 | 0.00467944 |
| cg26450574 | 6 | BRD2 | 32944067 | ENSG00000196301 | 6 | HLA-DRB9 | 32427598 | rs9271435 | 6 | 32588069 | T | C | 0.387674 | 0.34202 | 0.0412006 | 1.03E-16 | -0.198406 | 0.0333331 | 2.65E-09 | -1.72384 | 0.356367 | 1.32E-06 | 0.4773695 | 20 | 0.00771143 |
| cg20024110 | 6 | HLA-DOA | 32977708 | ENSG00000196301 | 6 | HLA-DRB9 | 32427598 | rs17191234 | 6 | 32564681 | C | A | 0.280318 | 0.343472 | 0.0420608 | 3.19E-16 | 0.211798 | 0.0335426 | 2.71E-10 | 1.6217 | 0.324652 | 5.88E-07 | 0.1867055 | 20 | 0.003443361 |
| cg17313945 | 6 | HLA-DOA | 32977983 | ENSG00000196301 | 6 | HLA-DRB9 | 32427598 | rs72853943 | 6 | 32509260 | C | T | 0.303181 | -0.640281 | 0.0434974 | 4.80E-49 | -0.193705 | 0.0346617 | 2.29E-08 | 3.30544 | 0.63267 | 1.75E-07 | 0.1725048 | 20 | 0.001022454 |
| cg05798641 | 6 | HLA-DPB1 | 33048919 | ENSG00000196301 | 6 | HLA-DRB9 | 32427598 | rs1812006 | 6 | 32778656 | A | G | 0.0934394 | -0.478147 | 0.0646942 | 1.46E-13 | -0.28559 | 0.0437937 | 6.97E-11 | 1.67424 | 0.342386 | 1.01E-06 | 0.1465431 | 13 | 0.005909281 |
| cg09510698 | 6 | HLA-DPB2 | 33092130 | ENSG00000196301 | 6 | HLA-DRB9 | 32427598 | rs1812006 | 6 | 32778656 | A | G | 0.0934394 | -0.478147 | 0.0646942 | 1.46E-13 | 0.248833 | 0.0436308 | 1.18E-08 | -1.92156 | 0.425577 | 6.33E-06 | 0.4101252 | 6 | 0.037059741 |
| cg15019001 | 6 | HLA-DPB2 | 33096303 | ENSG00000196301 | 6 | HLA-DRB9 | 32427598 | rs9271419 | 6 | 32587846 | T | C | 0.412525 | 0.346307 | 0.0411922 | 4.20E-17 | -0.245756 | 0.0325391 | 4.27E-14 | -1.40915 | 0.25081 | 1.93E-08 | 0.09438224 | 20 | 0.0001129 |
| cg05636843 | 6 | COL11A2 | 33138669 | ENSG00000196301 | 6 | HLA-DRB9 | 32427598 | rs482044 | 6 | 32576064 | C | G | 0.408549 | 0.329471 | 0.0416963 | 2.75E-15 | -0.186302 | 0.0333692 | 2.36E-08 | -1.76848 | 0.387848 | 5.12E-06 | 0.09609156 | 4 | 0.030006738 |
| cg00866054 | 6 | COL11A2 | 33142284 | ENSG00000196301 | 6 | HLA-DRB9 | 32427598 | rs1048709 | 6 | 31914935 | A | G | 0.152087 | -0.473411 | 0.0549177 | 6.67E-18 | 0.255627 | 0.0395423 | 1.02E-10 | -1.85196 | 0.358081 | 2.32E-07 | 0.1332626 | 20 | 0.001357436 |
| cg11960243 | 6 | COL11A2 | 33143135 | ENSG00000196301 | 6 | HLA-DRB9 | 32427598 | rs9271419 | 6 | 32587846 | T | C | 0.412525 | 0.346307 | 0.0411922 | 4.20E-17 | -0.209697 | 0.0326566 | 1.35E-10 | -1.65146 | 0.323623 | 3.34E-07 | 0.2628955 | 20 | 0.001957966 |
| cg02266086 | 6 | COL11A2 | 33161336 | ENSG00000196301 | 6 | HLA-DRB9 | 32427598 | rs481139 | 6 | 32576145 | A | C | 0.364811 | 0.322082 | 0.0426168 | 4.10E-14 | 0.210211 | 0.0337065 | 4.47E-10 | 1.53218 | 0.318527 | 1.51E-06 | 0.0886809 | 20 | 0.008831609 |
| cg10916998 | 6 | FLOT1 | 30711832 | ENSG00000198502 | 6 | HLA-DRB5 | 32485120 | rs3130062 | 6 | 31525912 | C | T | 0.0586481 | 0.509389 | 0.0726847 | 2.41E-12 | -0.357076 | 0.0550591 | 8.85E-11 | -1.42656 | 0.2997 | 1.94E-06 | 0.8635639 | 6 | 0.011343865 |
| cg06893977 | 6 | DDR1 | 30851928 | ENSG00000198502 | 6 | HLA-DRB5 | 32485120 | rs116667074 | 6 | 32285362 | T | C | 0.0785288 | -0.39122 | 0.0636047 | 7.71E-10 | 0.377097 | 0.046047 | 2.63E-16 | -1.03745 | 0.210945 | 8.74E-07 | 0.1385682 | 20 | 0.005118846 |
| cg13695585 | 6 | DDR1 | 30853014 | ENSG00000198502 | 6 | HLA-DRB5 | 32485120 | rs2516396 | 6 | 31521913 | A | G | 0.243539 | 0.317349 | 0.0415474 | 2.20E-14 | -0.255108 | 0.036596 | 3.15E-12 | -1.24398 | 0.241597 | 2.62E-07 | 0.05141066 | 12 | 0.001534261 |
| cg16215084 | 6 | DDR1 | 30853948 | ENSG00000198502 | 6 | HLA-DRB5 | 32485120 | rs2516396 | 6 | 31521913 | A | G | 0.243539 | 0.317349 | 0.0415474 | 2.20E-14 | -0.272804 | 0.0363419 | 6.07E-14 | -1.16329 | 0.217278 | 8.61E-08 | 0.1108542 | 11 | 0.000504244 |
| cg25251478 | 6 | DDR1 | 30853959 | ENSG00000198502 | 6 | HLA-DRB5 | 32485120 | rs2516396 | 6 | 31521913 | A | G | 0.243539 | 0.317349 | 0.0415474 | 2.20E-14 | -0.264368 | 0.0358789 | 1.73E-13 | -1.20041 | 0.226361 | 1.14E-07 | 0.3512852 | 9 | 0.000667116 |
| cg17879299 | 6 | DDR1 | 30860300 | ENSG00000198502 | 6 | HLA-DRB5 | 32485120 | rs2516396 | 6 | 31521913 | A | G | 0.243539 | 0.317349 | 0.0415474 | 2.20E-14 | -0.202981 | 0.0362391 | 2.13E-08 | -1.56344 | 0.346134 | 6.28E-06 | 0.5621978 | 6 | 0.036766026 |
| cg12669395 | 6 | DDR1 | 30860866 | ENSG00000198502 | 6 | HLA-DRB5 | 32485120 | rs3130062 | 6 | 31525912 | C | T | 0.0586481 | 0.509389 | 0.0726847 | 2.41E-12 | -0.362438 | 0.0554932 | 6.52E-11 | -1.40545 | 0.294151 | 1.77E-06 | 0.05490544 | 12 | 0.01037139 |
| cg07906263 | 6 | VARS2 | 30893133 | ENSG00000198502 | 6 | HLA-DRB5 | 32485120 | rs75237145 | 6 | 32594295 | T | G | 0.428429 | -0.523696 | 0.0317877 | 5.57E-61 | 0.194706 | 0.0329444 | 3.42E-09 | -2.68968 | 0.483493 | 2.65E-08 | 0.06183621 | 19 | 0.000155332 |
| cg11396628 | 6 | PSORS1C1 | 31095913 | ENSG00000198502 | 6 | HLA-DRB5 | 32485120 | rs9267654 | 6 | 31841009 | A | G | 0.158052 | 0.473473 | 0.0466728 | 3.50E-24 | 0.240911 | 0.0412022 | 5.00E-09 | 1.96534 | 0.387961 | 4.07E-07 | 0.06683888 | 3 | 0.002382039 |
| cg02357491 | 6 | PSORS1C1 | 31105744 | ENSG00000198502 | 6 | HLA-DRB5 | 32485120 | rs3117572 | 6 | 31717692 | A | G | 0.156064 | 0.458998 | 0.0466129 | 7.06E-23 | 0.221482 | 0.0404839 | 4.48E-08 | 2.07239 | 0.433343 | 1.73E-06 | 0.2070724 | 5 | 0.010150022 |
| cg27314422 | 6 | PSORS1C1 | 31105927 | ENSG00000198502 | 6 | HLA-DRB5 | 32485120 | rs9267654 | 6 | 31841009 | A | G | 0.158052 | 0.473473 | 0.0466728 | 3.50E-24 | 0.265428 | 0.0406255 | 6.42E-11 | 1.78381 | 0.324748 | 3.95E-08 | 0.05868848 | 10 | 0.000231622 |
| cg13815684 | 6 | LTA | 31540440 | ENSG00000198502 | 6 | HLA-DRB5 | 32485120 | rs2239704 | 6 | 31540141 | A | C | 0.407555 | 0.249581 | 0.0362772 | 5.99E-12 | 0.196768 | 0.0332407 | 3.23E-09 | 1.2684 | 0.282674 | 7.22E-06 | 0.5795776 | 5 | 0.042283249 |
| cg26348243 | 6 | LTA | 31540461 | ENSG00000198502 | 6 | HLA-DRB5 | 32485120 | rs746868 | 6 | 31540429 | C | G | 0.406561 | 0.248405 | 0.0362469 | 7.23E-12 | -0.277476 | 0.032953 | 3.75E-17 | -0.895231 | 0.168428 | 1.07E-07 | 0.1287536 | 8 | 0.000624021 |
| cg08610982 | 6 | NCR3 | 31560871 | ENSG00000198502 | 6 | HLA-DRB5 | 32485120 | rs2256965 | 6 | 31555130 | A | G | 0.451292 | 0.22307 | 0.0350714 | 2.01E-10 | 0.223943 | 0.0322664 | 3.91E-12 | 0.996102 | 0.212426 | 2.74E-06 | 0.1184001 | 9 | 0.016068705 |
| cg19563932 | 6 | AIF1 | 31583915 | ENSG00000198502 | 6 | HLA-DRB5 | 32485120 | rs1144708 | 6 | 31710020 | T | C | 0.361829 | -0.272625 | 0.0368048 | 1.29E-13 | 0.338079 | 0.0344652 | 1.03E-22 | -0.806394 | 0.136417 | 3.40E-09 | 0.240814 | 5 | 1.99E-05 |
| cg18113826 | 6 | AIF1 | 31583942 | ENSG00000198502 | 6 | HLA-DRB5 | 32485120 | rs1144708 | 6 | 31710020 | T | C | 0.361829 | -0.272625 | 0.0368048 | 1.29E-13 | 0.338958 | 0.0346463 | 1.33E-22 | -0.804303 | 0.136194 | 3.51E-09 | 0.2839672 | 5 | 2.06E-05 |
| cg25403205 | 6 | AIF1 | 31584215 | ENSG00000198502 | 6 | HLA-DRB5 | 32485120 | rs707938 | 6 | 31729359 | G | A | 0.319085 | -0.230656 | 0.0379962 | 1.28E-09 | 0.288542 | 0.0355984 | 5.25E-16 | -0.799384 | 0.16452 | 1.18E-06 | 0.1516953 | 6 | 0.006915428 |
| cg04812347 | 6 | AIF1 | 31584223 | ENSG00000198502 | 6 | HLA-DRB5 | 32485120 | rs707938 | 6 | 31729359 | G | A | 0.319085 | -0.230656 | 0.0379962 | 1.28E-09 | 0.302127 | 0.035667 | 2.44E-17 | -0.763441 | 0.154722 | 8.05E-07 | 0.1072822 | 6 | 0.004712815 |
| cg04762213 | 6 | BAT2 | 31587888 | ENSG00000198502 | 6 | HLA-DRB5 | 32485120 | rs1144708 | 6 | 31710020 | T | C | 0.361829 | -0.272625 | 0.0368048 | 1.29E-13 | -0.190008 | 0.0338779 | 2.04E-08 | 1.43481 | 0.320882 | 7.77E-06 | 0.9466479 | 3 | 0.045511159 |
| cg18842363 | 6 | C6orf47 | 31629088 | ENSG00000198502 | 6 | HLA-DRB5 | 32485120 | rs1144708 | 6 | 31710020 | T | C | 0.361829 | -0.272625 | 0.0368048 | 1.29E-13 | 0.228561 | 0.0276393 | 1.35E-16 | -1.19279 | 0.216184 | 3.44E-08 | 0.1621907 | 3 | 0.000201466 |
| cg26467528 | 6 | BAT4 | 31632248 | ENSG00000198502 | 6 | HLA-DRB5 | 32485120 | rs1144708 | 6 | 31710020 | T | C | 0.361829 | -0.272625 | 0.0368048 | 1.29E-13 | -0.2216 | 0.0344447 | 1.25E-10 | 1.23026 | 0.253283 | 1.19E-06 | 0.2298439 | 4 | 0.006973135 |
| cg17867605 | 6 | BAT4 | 31634162 | ENSG00000198502 | 6 | HLA-DRB5 | 32485120 | rs1144708 | 6 | 31710020 | T | C | 0.361829 | -0.272625 | 0.0368048 | 1.29E-13 | -0.248294 | 0.0343257 | 4.71E-13 | 1.09799 | 0.212164 | 2.28E-07 | 0.5818549 | 3 | 0.001333604 |
| cg10970124 | 6 | BAT4 | 31634602 | ENSG00000198502 | 6 | HLA-DRB5 | 32485120 | rs1144708 | 6 | 31710020 | T | C | 0.361829 | -0.272625 | 0.0368048 | 1.29E-13 | -0.19361 | 0.0346995 | 2.41E-08 | 1.40811 | 0.315953 | 8.32E-06 | 0.0653803 | 3 | 0.048753135 |
| cg19240857 | 6 | LY6G6E | 31683109 | ENSG00000198502 | 6 | HLA-DRB5 | 32485120 | rs1269852 | 6 | 32080191 | C | G | 0.0755467 | -0.392754 | 0.0647687 | 1.33E-09 | -0.393056 | 0.0466625 | 3.66E-17 | 0.999232 | 0.20304 | 8.60E-07 | 0.7041758 | 20 | 0.005035014 |
| cg10189661 | 6 | LY6G6E | 31683120 | ENSG00000198502 | 6 | HLA-DRB5 | 32485120 | rs1269852 | 6 | 32080191 | C | G | 0.0755467 | -0.392754 | 0.0647687 | 1.33E-09 | -0.406831 | 0.046791 | 3.48E-18 | 0.965398 | 0.194098 | 6.57E-07 | 0.3570309 | 20 | 0.003846643 |
| cg20288341 | 6 | LY6G6E | 31683131 | ENSG00000198502 | 6 | HLA-DRB5 | 32485120 | rs1269852 | 6 | 32080191 | C | G | 0.0755467 | -0.392754 | 0.0647687 | 1.33E-09 | -0.388125 | 0.0471017 | 1.72E-16 | 1.01193 | 0.207192 | 1.04E-06 | 0.5409253 | 20 | 0.006089555 |
| cg21506565 | 6 | C6orf25 | 31691506 | ENSG00000198502 | 6 | HLA-DRB5 | 32485120 | rs1150757 | 6 | 32029205 | A | G | 0.0755467 | -0.392754 | 0.0647687 | 1.33E-09 | -0.344267 | 0.0471623 | 2.89E-13 | 1.14084 | 0.244583 | 3.09E-06 | 0.2825969 | 20 | 0.018128073 |
| cg21620139 | 6 | C6orf25 | 31691535 | ENSG00000198502 | 6 | HLA-DRB5 | 32485120 | rs1150757 | 6 | 32029205 | A | G | 0.0755467 | -0.392754 | 0.0647687 | 1.33E-09 | -0.319972 | 0.0470628 | 1.05E-11 | 1.22746 | 0.271235 | 6.03E-06 | 0.5695718 | 20 | 0.035303635 |
| cg22235293 | 6 | C6orf25 | 31691539 | ENSG00000198502 | 6 | HLA-DRB5 | 32485120 | rs1150757 | 6 | 32029205 | A | G | 0.0755467 | -0.392754 | 0.0647687 | 1.33E-09 | -0.332948 | 0.0470731 | 1.52E-12 | 1.17963 | 0.256237 | 4.15E-06 | 0.5477193 | 20 | 0.024319534 |
| cg07014308 | 6 | C6orf25 | 31691597 | ENSG00000198502 | 6 | HLA-DRB5 | 32485120 | rs1150757 | 6 | 32029205 | A | G | 0.0755467 | -0.392754 | 0.0647687 | 1.33E-09 | -0.32853 | 0.0469678 | 2.66E-12 | 1.19549 | 0.260917 | 4.61E-06 | 0.4497903 | 20 | 0.026994713 |
| cg00406022 | 6 | C6orf25 | 31691696 | ENSG00000198502 | 6 | HLA-DRB5 | 32485120 | rs1150757 | 6 | 32029205 | A | G | 0.0755467 | -0.392754 | 0.0647687 | 1.33E-09 | -0.352843 | 0.0470159 | 6.15E-14 | 1.11311 | 0.235996 | 2.40E-06 | 0.4332068 | 20 | 0.014044912 |
| cg26749976 | 6 | C6orf25 | 31691717 | ENSG00000198502 | 6 | HLA-DRB5 | 32485120 | rs1269852 | 6 | 32080191 | C | G | 0.0755467 | -0.392754 | 0.0647687 | 1.33E-09 | -0.332929 | 0.0473426 | 2.03E-12 | 1.17969 | 0.256881 | 4.38E-06 | 0.5831418 | 20 | 0.025670892 |
| cg23895220 | 6 | C6orf25 | 31691870 | ENSG00000198502 | 6 | HLA-DRB5 | 32485120 | rs1150757 | 6 | 32029205 | A | G | 0.0755467 | -0.392754 | 0.0647687 | 1.33E-09 | -0.35133 | 0.0470649 | 8.34E-14 | 1.11791 | 0.237515 | 2.52E-06 | 0.6069965 | 20 | 0.014749255 |
| cg01338864 | 6 | C6orf25 | 31691905 | ENSG00000198502 | 6 | HLA-DRB5 | 32485120 | rs433061 | 6 | 32014828 | A | G | 0.0735587 | -0.392754 | 0.0647687 | 1.33E-09 | -0.334907 | 0.0466682 | 7.16E-13 | 1.17273 | 0.253191 | 3.63E-06 | 0.9746655 | 20 | 0.02123648 |
| cg00718684 | 6 | C6orf25 | 31692026 | ENSG00000198502 | 6 | HLA-DRB5 | 32485120 | rs1269852 | 6 | 32080191 | C | G | 0.0755467 | -0.392754 | 0.0647687 | 1.33E-09 | -0.354931 | 0.0471265 | 5.02E-14 | 1.10656 | 0.23428 | 2.32E-06 | 0.661483 | 20 | 0.013596213 |
| cg06132876 | 6 | C6orf25 | 31692080 | ENSG00000198502 | 6 | HLA-DRB5 | 32485120 | rs652888 | 6 | 31851234 | G | A | 0.200795 | -0.243242 | 0.0415497 | 4.79E-09 | -0.270343 | 0.0385019 | 2.19E-12 | 0.899753 | 0.200104 | 6.91E-06 | 0.0726668 | 20 | 0.040482119 |
| cg17731470 | 6 | C6orf25 | 31692152 | ENSG00000198502 | 6 | HLA-DRB5 | 32485120 | rs1144708 | 6 | 31710020 | T | C | 0.361829 | -0.272625 | 0.0368048 | 1.29E-13 | 0.260104 | 0.0345221 | 4.91E-14 | -1.04814 | 0.198431 | 1.28E-07 | 0.4688465 | 3 | 0.000748017 |
| cg03658690 | 6 | C6orf25 | 31692260 | ENSG00000198502 | 6 | HLA-DRB5 | 32485120 | rs1150757 | 6 | 32029205 | A | G | 0.0755467 | -0.392754 | 0.0647687 | 1.33E-09 | -0.372392 | 0.0469896 | 2.28E-15 | 1.05468 | 0.219001 | 1.47E-06 | 0.5076544 | 20 | 0.008585456 |
| cg26114961 | 6 | C6orf25 | 31692295 | ENSG00000198502 | 6 | HLA-DRB5 | 32485120 | rs433061 | 6 | 32014828 | A | G | 0.0735587 | -0.392754 | 0.0647687 | 1.33E-09 | -0.362051 | 0.046759 | 9.72E-15 | 1.0848 | 0.227226 | 1.81E-06 | 0.5886597 | 20 | 0.010573995 |
| cg20165831 | 6 | CLIC1 | 31701011 | ENSG00000198502 | 6 | HLA-DRB5 | 32485120 | rs501942 | 6 | 31840477 | T | C | 0.0735587 | -0.364815 | 0.06374 | 1.04E-08 | -0.36883 | 0.0468209 | 3.34E-15 | 0.989114 | 0.213615 | 3.65E-06 | 0.2222151 | 20 | 0.021384541 |
| cg13026137 | 6 | CLIC1 | 31701260 | ENSG00000198502 | 6 | HLA-DRB5 | 32485120 | rs3117577 | 6 | 31727474 | G | A | 0.0745527 | -0.369098 | 0.0628242 | 4.23E-09 | -0.488102 | 0.0454741 | 7.08E-27 | 0.75619 | 0.146731 | 2.56E-07 | 0.07582596 | 20 | 0.001496928 |
| cg12257344 | 6 | CLIC1 | 31701288 | ENSG00000198502 | 6 | HLA-DRB5 | 32485120 | rs3117577 | 6 | 31727474 | G | A | 0.0745527 | -0.369098 | 0.0628242 | 4.23E-09 | -0.665278 | 0.0448957 | 1.12E-49 | 0.554803 | 0.101584 | 4.72E-08 | 0.1557228 | 20 | 0.000276571 |
| cg18953509 | 6 | MSH5 | 31707301 | ENSG00000198502 | 6 | HLA-DRB5 | 32485120 | rs9272324 | 6 | 32604152 | G | A | 0.372763 | -0.23312 | 0.033797 | 5.29E-12 | -0.222099 | 0.0325451 | 8.83E-12 | 1.04962 | 0.216361 | 1.23E-06 | 0.1985213 | 20 | 0.007186647 |
| cg25264948 | 6 | C6orf27 | 31734232 | ENSG00000198502 | 6 | HLA-DRB5 | 32485120 | rs3115672 | 6 | 31727897 | T | C | 0.0745527 | -0.353207 | 0.0623111 | 1.44E-08 | -0.621875 | 0.0455222 | 1.74E-42 | 0.567971 | 0.108482 | 1.64E-07 | 0.1141361 | 20 | 0.000963261 |
| cg10095777 | 6 | C6orf27 | 31734292 | ENSG00000198502 | 6 | HLA-DRB5 | 32485120 | rs693906 | 6 | 31835164 | C | G | 0.11332 | -0.286933 | 0.0538243 | 9.77E-08 | -0.525149 | 0.0427564 | 1.13E-34 | 0.546384 | 0.111731 | 1.01E-06 | 0.05949519 | 20 | 0.005901988 |
| cg23287992 | 6 | C6orf27 | 31734390 | ENSG00000198502 | 6 | HLA-DRB5 | 32485120 | rs693906 | 6 | 31835164 | C | G | 0.11332 | -0.286933 | 0.0538243 | 9.77E-08 | -0.55131 | 0.0423741 | 1.07E-38 | 0.520457 | 0.105507 | 8.10E-07 | 0.06396734 | 20 | 0.004746159 |
| cg00848392 | 6 | C6orf27 | 31734401 | ENSG00000198502 | 6 | HLA-DRB5 | 32485120 | rs497309 | 6 | 31892484 | C | A | 0.0735587 | -0.363029 | 0.0643327 | 1.67E-08 | -0.484859 | 0.0462522 | 1.03E-25 | 0.748731 | 0.150686 | 6.74E-07 | 0.2948937 | 20 | 0.003945951 |
| cg25641533 | 6 | C6orf27 | 31734580 | ENSG00000198502 | 6 | HLA-DRB5 | 32485120 | rs693906 | 6 | 31835164 | C | G | 0.11332 | -0.286933 | 0.0538243 | 9.77E-08 | -0.364482 | 0.0429442 | 2.11E-17 | 0.787235 | 0.174387 | 6.35E-06 | 0.08193814 | 20 | 0.037213004 |
| cg16372051 | 6 | HSPA1B | 31794302 | ENSG00000198502 | 6 | HLA-DRB5 | 32485120 | rs1008438 | 6 | 31783208 | C | A | 0.403579 | -0.240464 | 0.0371925 | 1.01E-10 | 0.274625 | 0.0342214 | 1.02E-15 | -0.875609 | 0.173915 | 4.79E-07 | 0.2052251 | 14 | 0.002803795 |
| cg00232453 | 6 | EHMT2 | 31864574 | ENSG00000198502 | 6 | HLA-DRB5 | 32485120 | rs1043618 | 6 | 31783507 | C | G | 0.392644 | -0.234932 | 0.0376846 | 4.54E-10 | 0.302517 | 0.0344534 | 1.63E-18 | -0.776591 | 0.152775 | 3.71E-07 | 0.09734543 | 6 | 0.002173851 |
| cg16505946 | 6 | ZBTB12 | 31870305 | ENSG00000198502 | 6 | HLA-DRB5 | 32485120 | rs501942 | 6 | 31840477 | T | C | 0.0735587 | -0.364815 | 0.06374 | 1.04E-08 | 0.580822 | 0.04417 | 1.71E-39 | -0.628101 | 0.119686 | 1.54E-07 | 0.0699005 | 20 | 0.000901068 |
| cg06540876 | 6 | ZBTB12 | 31870600 | ENSG00000198502 | 6 | HLA-DRB5 | 32485120 | rs693906 | 6 | 31835164 | C | G | 0.11332 | -0.286933 | 0.0538243 | 9.77E-08 | -0.395048 | 0.0425347 | 1.58E-20 | 0.726324 | 0.157096 | 3.77E-06 | 0.09315877 | 4 | 0.022109217 |
| cg24508713 | 6 | ZBTB12 | 31870783 | ENSG00000198502 | 6 | HLA-DRB5 | 32485120 | rs693906 | 6 | 31835164 | C | G | 0.11332 | -0.286933 | 0.0538243 | 9.77E-08 | -0.366387 | 0.0431391 | 2.01E-17 | 0.783142 | 0.173447 | 6.33E-06 | 0.06405948 | 7 | 0.037060127 |
| cg20069688 | 6 | STK19 | 31941049 | ENSG00000198502 | 6 | HLA-DRB5 | 32485120 | rs1270942 | 6 | 31918860 | G | A | 0.0725646 | -0.363029 | 0.0643327 | 1.67E-08 | -0.470909 | 0.046305 | 2.71E-24 | 0.770911 | 0.156236 | 8.04E-07 | 0.1724558 | 20 | 0.004712683 |
| cg21415604 | 6 | C4B | 31948433 | ENSG00000198502 | 6 | HLA-DRB5 | 32485120 | rs693906 | 6 | 31835164 | C | G | 0.11332 | -0.286933 | 0.0538243 | 9.77E-08 | 0.469653 | 0.0428408 | 5.77E-28 | -0.610947 | 0.127436 | 1.63E-06 | 0.08301593 | 20 | 0.009570238 |
| cg04771084 | 6 | CYP21A2 | 31973255 | ENSG00000198502 | 6 | HLA-DRB5 | 32485120 | rs433061 | 6 | 32014828 | A | G | 0.0735587 | -0.392754 | 0.0647687 | 1.33E-09 | -1.26866 | 0.0384522 | 1.02E-238 | 0.309582 | 0.051908 | 2.46E-09 | 0.4701722 | 20 | 1.44E-05 |
| cg21722170 | 6 | TNXB | 31977443 | ENSG00000198502 | 6 | HLA-DRB5 | 32485120 | rs1270942 | 6 | 31918860 | G | A | 0.0725646 | -0.363029 | 0.0643327 | 1.67E-08 | 0.684243 | 0.0455104 | 4.34E-51 | -0.530556 | 0.100424 | 1.27E-07 | 0.2693084 | 20 | 0.00074393 |
| cg14026451 | 6 | CYP21A2 | 32007625 | ENSG00000198502 | 6 | HLA-DRB5 | 32485120 | rs1144708 | 6 | 31710020 | T | C | 0.361829 | -0.272625 | 0.0368048 | 1.29E-13 | -0.193066 | 0.0342471 | 1.73E-08 | 1.41208 | 0.314774 | 7.26E-06 | 0.849255 | 3 | 0.042509315 |
| cg09115485 | 6 | TNXB | 32014605 | ENSG00000198502 | 6 | HLA-DRB5 | 32485120 | rs1150753 | 6 | 32059867 | G | A | 0.0755467 | -0.392754 | 0.0647687 | 1.33E-09 | -0.461117 | 0.046625 | 4.60E-23 | 0.851745 | 0.164761 | 2.35E-07 | 0.08568282 | 20 | 0.001374463 |
| cg02272968 | 6 | TNXB | 32015773 | ENSG00000198502 | 6 | HLA-DRB5 | 32485120 | rs1043618 | 6 | 31783507 | C | G | 0.392644 | -0.234932 | 0.0376846 | 4.54E-10 | -0.25212 | 0.0340092 | 1.23E-13 | 0.931826 | 0.195298 | 1.83E-06 | 0.4133083 | 19 | 0.010722284 |
| cg02432444 | 6 | TNXB | 32016214 | ENSG00000198502 | 6 | HLA-DRB5 | 32485120 | rs433061 | 6 | 32014828 | A | G | 0.0735587 | -0.392754 | 0.0647687 | 1.33E-09 | -0.412349 | 0.0461336 | 3.96E-19 | 0.95248 | 0.189809 | 5.22E-07 | 0.4259423 | 20 | 0.0030572 |
| cg19964491 | 6 | TNXB | 32016236 | ENSG00000198502 | 6 | HLA-DRB5 | 32485120 | rs1269852 | 6 | 32080191 | C | G | 0.0755467 | -0.392754 | 0.0647687 | 1.33E-09 | -0.518279 | 0.0463775 | 5.39E-29 | 0.757804 | 0.142181 | 9.83E-08 | 0.1760836 | 20 | 0.000575835 |
| cg11493661 | 6 | TNXB | 32016239 | ENSG00000198502 | 6 | HLA-DRB5 | 32485120 | rs1269852 | 6 | 32080191 | C | G | 0.0755467 | -0.392754 | 0.0647687 | 1.33E-09 | -0.44277 | 0.0466475 | 2.27E-21 | 0.887038 | 0.173584 | 3.22E-07 | 0.627957 | 20 | 0.001885886 |
| cg08661360 | 6 | TNXB | 32016247 | ENSG00000198502 | 6 | HLA-DRB5 | 32485120 | rs1269852 | 6 | 32080191 | C | G | 0.0755467 | -0.392754 | 0.0647687 | 1.33E-09 | -0.554886 | 0.0463507 | 5.01E-33 | 0.70781 | 0.130845 | 6.32E-08 | 0.3040693 | 20 | 0.000370132 |
| cg05473289 | 6 | TNXB | 32016257 | ENSG00000198502 | 6 | HLA-DRB5 | 32485120 | rs1269852 | 6 | 32080191 | C | G | 0.0755467 | -0.392754 | 0.0647687 | 1.33E-09 | -0.526677 | 0.0464248 | 7.88E-30 | 0.745721 | 0.139441 | 8.90E-08 | 0.408494 | 20 | 0.000521213 |
| cg05298224 | 6 | TNXB | 32016360 | ENSG00000198502 | 6 | HLA-DRB5 | 32485120 | rs501942 | 6 | 31840477 | T | C | 0.0735587 | -0.364815 | 0.06374 | 1.04E-08 | -0.638743 | 0.045425 | 6.55E-45 | 0.571145 | 0.107739 | 1.15E-07 | 0.1838737 | 20 | 0.000674029 |
| cg09474017 | 6 | TNXB | 32016368 | ENSG00000198502 | 6 | HLA-DRB5 | 32485120 | rs1269852 | 6 | 32080191 | C | G | 0.0755467 | -0.392754 | 0.0647687 | 1.33E-09 | -0.619731 | 0.0458156 | 1.09E-41 | 0.633749 | 0.114532 | 3.14E-08 | 0.3220128 | 20 | 0.000184027 |
| cg04293778 | 6 | TNXB | 32016426 | ENSG00000198502 | 6 | HLA-DRB5 | 32485120 | rs1150753 | 6 | 32059867 | G | A | 0.0755467 | -0.392754 | 0.0647687 | 1.33E-09 | -0.394892 | 0.0463772 | 1.67E-17 | 0.994586 | 0.201358 | 7.84E-07 | 0.14103 | 20 | 0.004590973 |
| cg15398152 | 6 | TNXB | 32016535 | ENSG00000198502 | 6 | HLA-DRB5 | 32485120 | rs1150757 | 6 | 32029205 | A | G | 0.0755467 | -0.392754 | 0.0647687 | 1.33E-09 | -0.375444 | 0.0471082 | 1.59E-15 | 1.04611 | 0.21677 | 1.39E-06 | 0.2166129 | 20 | 0.00816532 |
| cg24055029 | 6 | TNXB | 32055137 | ENSG00000198502 | 6 | HLA-DRB5 | 32485120 | rs2856698 | 6 | 32636376 | G | C | 0.281312 | -0.233058 | 0.0377137 | 6.42E-10 | -0.293377 | 0.0360021 | 3.67E-16 | 0.794398 | 0.161334 | 8.48E-07 | 0.08743123 | 20 | 0.004968789 |
| cg03130418 | 6 | TNXB | 32063439 | ENSG00000198502 | 6 | HLA-DRB5 | 32485120 | rs9272324 | 6 | 32604152 | G | A | 0.372763 | -0.23312 | 0.033797 | 5.29E-12 | -0.200318 | 0.0328979 | 1.14E-09 | 1.16375 | 0.254936 | 5.00E-06 | 0.09997871 | 20 | 0.029279087 |
| cg18460422 | 6 | TNXB | 32063553 | ENSG00000198502 | 6 | HLA-DRB5 | 32485120 | rs6933289 | 6 | 32604551 | T | C | 0.0715706 | -0.434361 | 0.0542221 | 1.14E-15 | -0.338749 | 0.04543 | 8.88E-14 | 1.28225 | 0.234931 | 4.82E-08 | 0.3392981 | 20 | 0.000282105 |
| cg14196170 | 6 | TNXB | 32063595 | ENSG00000198502 | 6 | HLA-DRB5 | 32485120 | rs6933289 | 6 | 32604551 | T | C | 0.0715706 | -0.434361 | 0.0542221 | 1.14E-15 | -0.344152 | 0.0455658 | 4.26E-14 | 1.26212 | 0.229667 | 3.90E-08 | 0.3817195 | 20 | 0.000228314 |
| cg26695758 | 6 | TNXB | 32063607 | ENSG00000198502 | 6 | HLA-DRB5 | 32485120 | rs1270942 | 6 | 31918860 | G | A | 0.0725646 | -0.363029 | 0.0643327 | 1.67E-08 | -0.443469 | 0.0467647 | 2.47E-21 | 0.818612 | 0.168808 | 1.24E-06 | 0.7346436 | 20 | 0.007255936 |
| cg04753078 | 6 | TNXB | 32063619 | ENSG00000198502 | 6 | HLA-DRB5 | 32485120 | rs6933289 | 6 | 32604551 | T | C | 0.0715706 | -0.434361 | 0.0542221 | 1.14E-15 | -0.410289 | 0.0453394 | 1.44E-19 | 1.05867 | 0.176499 | 2.00E-09 | 0.1473198 | 20 | 1.17E-05 |
| cg00779476 | 6 | TNXB | 32063726 | ENSG00000198502 | 6 | HLA-DRB5 | 32485120 | rs1270942 | 6 | 31918860 | G | A | 0.0725646 | -0.363029 | 0.0643327 | 1.67E-08 | -0.360455 | 0.0470893 | 1.94E-14 | 1.00714 | 0.221731 | 5.57E-06 | 0.3411362 | 20 | 0.032616993 |
| cg01337207 | 6 | TNXB | 32063835 | ENSG00000198502 | 6 | HLA-DRB5 | 32485120 | rs1150753 | 6 | 32059867 | G | A | 0.0755467 | -0.392754 | 0.0647687 | 1.33E-09 | -0.427682 | 0.0465025 | 3.68E-20 | 0.918332 | 0.181397 | 4.14E-07 | 0.7108313 | 20 | 0.002423264 |
| cg26266427 | 6 | TNXB | 32063838 | ENSG00000198502 | 6 | HLA-DRB5 | 32485120 | rs433061 | 6 | 32014828 | A | G | 0.0735587 | -0.392754 | 0.0647687 | 1.33E-09 | -0.429055 | 0.0465627 | 3.13E-20 | 0.915393 | 0.180712 | 4.07E-07 | 0.7564114 | 20 | 0.002386315 |
| cg10365886 | 6 | TNXB | 32063874 | ENSG00000198502 | 6 | HLA-DRB5 | 32485120 | rs1270942 | 6 | 31918860 | G | A | 0.0725646 | -0.363029 | 0.0643327 | 1.67E-08 | -0.423522 | 0.04665 | 1.10E-19 | 0.857167 | 0.178851 | 1.65E-06 | 0.5232868 | 20 | 0.009642356 |
| cg07524919 | 6 | TNXB | 32063901 | ENSG00000198502 | 6 | HLA-DRB5 | 32485120 | rs1270942 | 6 | 31918860 | G | A | 0.0725646 | -0.363029 | 0.0643327 | 1.67E-08 | -0.44475 | 0.0466635 | 1.56E-21 | 0.816254 | 0.168101 | 1.20E-06 | 0.6147566 | 20 | 0.00702598 |
| cg00872984 | 6 | TNXB | 32063991 | ENSG00000198502 | 6 | HLA-DRB5 | 32485120 | rs1270942 | 6 | 31918860 | G | A | 0.0725646 | -0.363029 | 0.0643327 | 1.67E-08 | -0.446838 | 0.0467016 | 1.09E-21 | 0.81244 | 0.167148 | 1.17E-06 | 0.4331529 | 20 | 0.00685553 |
| cg27387193 | 6 | TNXB | 32064032 | ENSG00000198502 | 6 | HLA-DRB5 | 32485120 | rs1150757 | 6 | 32029205 | A | G | 0.0755467 | -0.392754 | 0.0647687 | 1.33E-09 | -0.438225 | 0.0467498 | 6.99E-21 | 0.896238 | 0.176027 | 3.55E-07 | 0.8670695 | 20 | 0.002081277 |
| cg17662683 | 6 | TNXB | 32064146 | ENSG00000198502 | 6 | HLA-DRB5 | 32485120 | rs6933289 | 6 | 32604551 | T | C | 0.0715706 | -0.434361 | 0.0542221 | 1.14E-15 | -0.379947 | 0.0453648 | 5.51E-17 | 1.14321 | 0.197478 | 7.08E-09 | 0.1813908 | 20 | 4.15E-05 |
| cg01569346 | 6 | TNXB | 32064148 | ENSG00000198502 | 6 | HLA-DRB5 | 32485120 | rs6910310 | 6 | 32604221 | G | A | 0.0666004 | -0.43389 | 0.0544031 | 1.52E-15 | -0.373024 | 0.0453224 | 1.87E-16 | 1.16317 | 0.203084 | 1.02E-08 | 0.2071532 | 20 | 5.97E-05 |
| cg19267551 | 6 | TNXB | 32064161 | ENSG00000198502 | 6 | HLA-DRB5 | 32485120 | rs433061 | 6 | 32014828 | A | G | 0.0735587 | -0.392754 | 0.0647687 | 1.33E-09 | -0.331752 | 0.0469019 | 1.51E-12 | 1.18388 | 0.257156 | 4.15E-06 | 0.7182265 | 20 | 0.024309593 |
| cg08516507 | 6 | TNXB | 32064206 | ENSG00000198502 | 6 | HLA-DRB5 | 32485120 | rs9272729 | 6 | 32609594 | A | G | 0.106362 | -0.340137 | 0.0507436 | 2.04E-11 | -0.33316 | 0.0465419 | 8.17E-13 | 1.02094 | 0.208662 | 9.94E-07 | 0.183903 | 20 | 0.005823619 |
| cg10923662 | 6 | TNXB | 32064258 | ENSG00000198502 | 6 | HLA-DRB5 | 32485120 | rs678770 | 6 | 32570817 | A | T | 0.222664 | -0.265982 | 0.0394367 | 1.54E-11 | -0.340809 | 0.0384524 | 7.78E-19 | 0.780443 | 0.145408 | 7.99E-08 | 0.07125287 | 20 | 0.000468328 |
| cg12694372 | 6 | TNXB | 32064582 | ENSG00000198502 | 6 | HLA-DRB5 | 32485120 | rs433061 | 6 | 32014828 | A | G | 0.0735587 | -0.392754 | 0.0647687 | 1.33E-09 | -0.503299 | 0.046406 | 2.09E-27 | 0.780359 | 0.147437 | 1.20E-07 | 0.792101 | 20 | 0.000705564 |
| cg15265085 | 6 | TNXB | 32064588 | ENSG00000198502 | 6 | HLA-DRB5 | 32485120 | rs497309 | 6 | 31892484 | C | A | 0.0735587 | -0.363029 | 0.0643327 | 1.67E-08 | -0.504927 | 0.0461071 | 6.56E-28 | 0.718973 | 0.14333 | 5.27E-07 | 0.4191514 | 20 | 0.00308731 |
| cg05889186 | 6 | TNXB | 32064842 | ENSG00000198502 | 6 | HLA-DRB5 | 32485120 | rs1150757 | 6 | 32029205 | A | G | 0.0755467 | -0.392754 | 0.0647687 | 1.33E-09 | -0.570207 | 0.0461572 | 4.66E-35 | 0.688792 | 0.126535 | 5.22E-08 | 0.4460667 | 20 | 0.00030604 |
| cg00355613 | 6 | TNXB | 32064956 | ENSG00000198502 | 6 | HLA-DRB5 | 32485120 | rs6910310 | 6 | 32604221 | G | A | 0.0666004 | -0.43389 | 0.0544031 | 1.52E-15 | -0.548759 | 0.0447987 | 1.69E-34 | 0.790675 | 0.1183 | 2.33E-11 | 0.4069925 | 20 | 1.37E-07 |
| cg10783204 | 6 | TNXB | 32065011 | ENSG00000198502 | 6 | HLA-DRB5 | 32485120 | rs9270614 | 6 | 32565115 | A | C | 0.357853 | -0.36572 | 0.035672 | 1.16E-24 | -0.236076 | 0.0360217 | 5.61E-11 | 1.54916 | 0.280549 | 3.35E-08 | 0.3355986 | 20 | 0.000196448 |
| cg26476939 | 6 | TNXB | 32065043 | ENSG00000198502 | 6 | HLA-DRB5 | 32485120 | rs6910310 | 6 | 32604221 | G | A | 0.0666004 | -0.43389 | 0.0544031 | 1.52E-15 | -0.318064 | 0.0460304 | 4.85E-12 | 1.36416 | 0.261212 | 1.77E-07 | 0.3826172 | 20 | 0.001034484 |
| cg21386484 | 6 | GPSM3 | 32159527 | ENSG00000198502 | 6 | HLA-DRB5 | 32485120 | rs693906 | 6 | 31835164 | C | G | 0.11332 | -0.286933 | 0.0538243 | 9.77E-08 | -0.497817 | 0.042591 | 1.46E-31 | 0.576382 | 0.118835 | 1.23E-06 | 0.07975793 | 20 | 0.00722285 |
| cg00442389 | 6 | GPSM3 | 32160074 | ENSG00000198502 | 6 | HLA-DRB5 | 32485120 | rs693906 | 6 | 31835164 | C | G | 0.11332 | -0.286933 | 0.0538243 | 9.77E-08 | -0.563585 | 0.0423699 | 2.27E-40 | 0.509121 | 0.102888 | 7.49E-07 | 0.06046406 | 20 | 0.004385345 |
| cg06023661 | 6 | NOTCH4 | 32164801 | ENSG00000198502 | 6 | HLA-DRB5 | 32485120 | rs9271377 | 6 | 32587165 | G | T | 0.405567 | -0.324184 | 0.0351313 | 2.76E-20 | -0.283338 | 0.0340125 | 8.06E-17 | 1.14416 | 0.185035 | 6.27E-10 | 0.09569024 | 20 | 3.67E-06 |
| cg16390049 | 6 | NOTCH4 | 32165089 | ENSG00000198502 | 6 | HLA-DRB5 | 32485120 | rs1269852 | 6 | 32080191 | C | G | 0.0755467 | -0.392754 | 0.0647687 | 1.33E-09 | -0.493083 | 0.0465366 | 3.12E-26 | 0.796527 | 0.151345 | 1.42E-07 | 0.656242 | 20 | 0.000830286 |
| cg08828723 | 6 | NOTCH4 | 32165176 | ENSG00000198502 | 6 | HLA-DRB5 | 32485120 | rs116667074 | 6 | 32285362 | T | C | 0.0785288 | -0.39122 | 0.0636047 | 7.71E-10 | -0.458843 | 0.0466065 | 7.20E-23 | 0.852623 | 0.16345 | 1.82E-07 | 0.2570659 | 20 | 0.001068548 |
| cg06891815 | 6 | NOTCH4 | 32165237 | ENSG00000198502 | 6 | HLA-DRB5 | 32485120 | rs116667074 | 6 | 32285362 | T | C | 0.0785288 | -0.39122 | 0.0636047 | 7.71E-10 | -0.516795 | 0.046607 | 1.43E-28 | 0.757012 | 0.140743 | 7.50E-08 | 0.7752144 | 20 | 0.000439498 |
| cg12179641 | 6 | NOTCH4 | 32188404 | ENSG00000198502 | 6 | HLA-DRB5 | 32485120 | rs9272310 | 6 | 32603939 | T | G | 0.38171 | -0.227699 | 0.0372786 | 1.01E-09 | 0.430183 | 0.033134 | 1.53E-38 | -0.529307 | 0.0957687 | 3.26E-08 | 0.1535776 | 20 | 0.000190895 |
| cg10158182 | 6 | NOTCH4 | 32188822 | ENSG00000198502 | 6 | HLA-DRB5 | 32485120 | rs1265888 | 6 | 32066447 | A | G | 0.11332 | -0.267776 | 0.053133 | 4.66E-07 | -0.450652 | 0.0419128 | 5.79E-27 | 0.594197 | 0.130211 | 5.03E-06 | 0.3621959 | 5 | 0.029494948 |
| cg09177461 | 6 | C6orf10 | 32280185 | ENSG00000198502 | 6 | HLA-DRB5 | 32485120 | rs1269852 | 6 | 32080191 | C | G | 0.0755467 | -0.392754 | 0.0647687 | 1.33E-09 | 0.382439 | 0.0465862 | 2.23E-16 | -1.02697 | 0.210551 | 1.07E-06 | 0.200928 | 20 | 0.006292031 |
| cg21241195 | 6 | C6orf10 | 32289357 | ENSG00000198502 | 6 | HLA-DRB5 | 32485120 | rs116667074 | 6 | 32285362 | T | C | 0.0785288 | -0.39122 | 0.0636047 | 7.71E-10 | -1.3925 | 0.0367946 | 0 | 0.280948 | 0.046276 | 1.27E-09 | 0.243526 | 20 | 7.44E-06 |
| cg13966843 | 6 | C6orf10 | 32336160 | ENSG00000198502 | 6 | HLA-DRB5 | 32485120 | rs116667074 | 6 | 32285362 | T | C | 0.0785288 | -0.39122 | 0.0636047 | 7.71E-10 | -1.4175 | 0.0363769 | 0 | 0.275993 | 0.0454266 | 1.24E-09 | 0.2949851 | 20 | 7.24E-06 |
| cg15815970 | 6 | BTNL2 | 32371533 | ENSG00000198502 | 6 | HLA-DRB5 | 32485120 | rs9270580 | 6 | 32561123 | C | T | 0.434394 | -0.492495 | 0.0345296 | 3.72E-46 | -0.209843 | 0.0293822 | 9.21E-13 | 2.34697 | 0.367517 | 1.70E-10 | 0.06791395 | 20 | 9.97E-07 |
| cg06968241 | 6 | HLA-DRA | 32411144 | ENSG00000198502 | 6 | HLA-DRB5 | 32485120 | rs9271724 | 6 | 32593552 | C | A | 0.26839 | -0.466294 | 0.0359603 | 1.89E-38 | 0.195761 | 0.035437 | 3.31E-08 | -2.38196 | 0.468684 | 3.73E-07 | 0.0700015 | 20 | 0.002185372 |
| cg08269402 | 6 | HLA-DRB1 | 32549631 | ENSG00000198502 | 6 | HLA-DRB5 | 32485120 | rs9270567 | 6 | 32560801 | G | T | 0.435388 | -0.480438 | 0.0341416 | 5.65E-45 | 1.12494 | 0.0240336 | 0 | -0.427079 | 0.0316916 | 2.16E-41 | 0.06621334 | 20 | 1.27E-37 |
| cg23214071 | 6 | HLA-DQB1 | 32627784 | ENSG00000198502 | 6 | HLA-DRB5 | 32485120 | rs6910310 | 6 | 32604221 | G | A | 0.0666004 | -0.43389 | 0.0544031 | 1.52E-15 | -1.20953 | 0.0384611 | 4.45E-217 | 0.358726 | 0.0464026 | 1.07E-14 | 0.06395105 | 20 | 6.26E-11 |
| cg24593918 | 6 | HLA-DQB1 | 32633157 | ENSG00000198502 | 6 | HLA-DRB5 | 32485120 | rs9270911 | 6 | 32572202 | C | T | 0.463221 | 0.208864 | 0.0362624 | 8.42E-09 | -1.0092 | 0.0256225 | 0 | -0.20696 | 0.036314 | 1.20E-08 | 0.7301185 | 20 | 7.05E-05 |
| cg23464743 | 6 | HLA-DQB1 | 32633163 | ENSG00000198502 | 6 | HLA-DRB5 | 32485120 | rs9274617 | 6 | 32635904 | A | T | 0.379722 | -0.179215 | 0.0384704 | 3.18E-06 | 1.03933 | 0.0265367 | 0 | -0.172433 | 0.0372755 | 3.73E-06 | 0.1028066 | 20 | 0.021847798 |
| cg23336481 | 6 | HLA-DQB1 | 32634344 | ENSG00000198502 | 6 | HLA-DRB5 | 32485120 | rs9272359 | 6 | 32604544 | T | C | 0.333002 | -0.226153 | 0.0372267 | 1.24E-09 | 0.759444 | 0.0300879 | 1.43E-140 | -0.297788 | 0.0504181 | 3.50E-09 | 0.2489334 | 20 | 2.05E-05 |
| cg01050736 | 6 | HLA-DQA2 | 32710583 | ENSG00000198502 | 6 | HLA-DRB5 | 32485120 | rs3763354 | 6 | 32786917 | G | A | 0.198807 | 0.297029 | 0.0422977 | 2.18E-12 | 0.232454 | 0.0372564 | 4.40E-10 | 1.2778 | 0.273957 | 3.10E-06 | 0.08110016 | 14 | 0.018146403 |
| cg06423300 | 6 | HLA-DQB2 | 32729059 | ENSG00000198502 | 6 | HLA-DRB5 | 32485120 | rs6910310 | 6 | 32604221 | G | A | 0.0666004 | -0.43389 | 0.0544031 | 1.52E-15 | -0.829694 | 0.0444661 | 1.07E-77 | 0.522952 | 0.0713088 | 2.24E-13 | 0.1002933 | 20 | 1.31E-09 |
| cg03638120 | 6 | HLA-DQB2 | 32729720 | ENSG00000198502 | 6 | HLA-DRB5 | 32485120 | rs2760994 | 6 | 32574308 | C | T | 0.405567 | 0.468792 | 0.0339287 | 2.01E-43 | 0.196886 | 0.0325691 | 1.49E-09 | 2.38103 | 0.429921 | 3.05E-08 | 0.273853 | 20 | 0.000178915 |
| cg19939773 | 6 | HLA-DQB2 | 32729876 | ENSG00000198502 | 6 | HLA-DRB5 | 32485120 | rs9271539 | 6 | 32590028 | A | G | 0.310139 | -0.475293 | 0.0360428 | 1.04E-39 | -0.227743 | 0.0355208 | 1.44E-10 | 2.08697 | 0.361937 | 8.11E-09 | 0.2481814 | 20 | 4.75E-05 |
| cg11530659 | 6 | HLA-DQB2 | 32730001 | ENSG00000198502 | 6 | HLA-DRB5 | 32485120 | rs3763354 | 6 | 32786917 | G | A | 0.198807 | 0.297029 | 0.0422977 | 2.18E-12 | -0.266892 | 0.0380066 | 2.18E-12 | -1.11292 | 0.224129 | 6.85E-07 | 0.05206061 | 12 | 0.004013726 |
| cg18073883 | 6 | HLA-DOB | 32780861 | ENSG00000198502 | 6 | HLA-DRB5 | 32485120 | rs241439 | 6 | 32797537 | G | T | 0.388668 | -0.270771 | 0.034235 | 2.59E-15 | -0.263605 | 0.0331729 | 1.92E-15 | 1.02718 | 0.183238 | 2.07E-08 | 0.2018077 | 11 | 0.000121467 |
| cg02633767 | 6 | TAP2 | 32794327 | ENSG00000198502 | 6 | HLA-DRB5 | 32485120 | rs241439 | 6 | 32797537 | G | T | 0.388668 | -0.270771 | 0.034235 | 2.59E-15 | -0.372994 | 0.0333209 | 4.36E-29 | 0.725939 | 0.112383 | 1.05E-10 | 0.1009365 | 15 | 6.15E-07 |
| cg07404352 | 6 | TAP2 | 32795705 | ENSG00000198502 | 6 | HLA-DRB5 | 32485120 | rs2621331 | 6 | 32780470 | C | T | 0.333002 | 0.284472 | 0.0363761 | 5.27E-15 | 0.325781 | 0.0296228 | 3.92E-28 | 0.8732 | 0.13701 | 1.85E-10 | 0.08379601 | 16 | 1.08E-06 |
| cg11381564 | 6 | PSMB8 | 32808619 | ENSG00000198502 | 6 | HLA-DRB5 | 32485120 | rs2071540 | 6 | 32812916 | T | C | 0.418489 | 0.207881 | 0.0348154 | 2.36E-09 | 0.217489 | 0.0321419 | 1.32E-11 | 0.955823 | 0.213492 | 7.57E-06 | 0.6087713 | 3 | 0.044326789 |
| cg12048225 | 6 | PSMB8 | 32808669 | ENSG00000198502 | 6 | HLA-DRB5 | 32485120 | rs2071540 | 6 | 32812916 | T | C | 0.418489 | 0.207881 | 0.0348154 | 2.36E-09 | 0.277794 | 0.0327068 | 2.01E-17 | 0.748328 | 0.153199 | 1.04E-06 | 0.6146736 | 3 | 0.006068425 |
| cg00971309 | 6 | PSMB8 | 32809960 | ENSG00000198502 | 6 | HLA-DRB5 | 32485120 | rs2071540 | 6 | 32812916 | T | C | 0.418489 | 0.207881 | 0.0348154 | 2.36E-09 | 0.235079 | 0.0321481 | 2.62E-13 | 0.884303 | 0.191203 | 3.75E-06 | 0.6754593 | 5 | 0.021949364 |
| cg24031377 | 6 | PSMB8 | 32810026 | ENSG00000198502 | 6 | HLA-DRB5 | 32485120 | rs2071540 | 6 | 32812916 | T | C | 0.418489 | 0.207881 | 0.0348154 | 2.36E-09 | 0.275737 | 0.0319869 | 6.68E-18 | 0.75391 | 0.153594 | 9.18E-07 | 0.4604454 | 5 | 0.005377311 |
| cg17451945 | 6 | BRD2 | 32936363 | ENSG00000198502 | 6 | HLA-DRB5 | 32485120 | rs241439 | 6 | 32797537 | G | T | 0.388668 | -0.270771 | 0.034235 | 2.59E-15 | -0.24086 | 0.0331884 | 3.95E-13 | 1.12418 | 0.210232 | 8.93E-08 | 0.141802 | 4 | 0.000522865 |
| cg17313945 | 6 | HLA-DOA | 32977983 | ENSG00000198502 | 6 | HLA-DRB5 | 32485120 | rs72853943 | 6 | 32509260 | C | T | 0.303181 | 0.51753 | 0.0359184 | 4.58E-47 | -0.193705 | 0.0346617 | 2.29E-08 | -2.67174 | 0.512784 | 1.89E-07 | 0.07190974 | 20 | 0.00110463 |
| cg23201206 | 6 | VARS2 | 30885800 | ENSG00000196735 | 6 | HLA-DQA1 | 32595956 | rs2647066 | 6 | 32571122 | T | C | 0.167992 | -0.184086 | 0.030145 | 1.02E-09 | 0.305674 | 0.0404803 | 4.31E-14 | -0.60223 | 0.126831 | 2.05E-06 | 0.1312657 | 20 | 0.012017418 |
| cg03798603 | 6 | SFTA2 | 30899145 | ENSG00000196735 | 6 | HLA-DQA1 | 32595956 | rs9270710 | 6 | 32567368 | C | G | 0.296223 | 0.211558 | 0.0245459 | 6.76E-18 | 0.201037 | 0.0341982 | 4.14E-09 | 1.05233 | 0.216685 | 1.19E-06 | 0.1408322 | 20 | 0.00699874 |
| cg01016122 | 6 | PSORS1C1 | 31082534 | ENSG00000196735 | 6 | HLA-DQA1 | 32595956 | rs9274500 | 6 | 32634024 | A | T | 0.357853 | -0.236152 | 0.0236386 | 1.68E-23 | 0.196507 | 0.034859 | 1.73E-08 | -1.20175 | 0.24478 | 9.13E-07 | 0.05813708 | 20 | 0.005348642 |
| cg01015008 | 6 | PSORS1C1 | 31095845 | ENSG00000196735 | 6 | HLA-DQA1 | 32595956 | rs9270805 | 6 | 32569556 | T | A | 0.269384 | 0.223171 | 0.025459 | 1.85E-18 | -0.212025 | 0.0345742 | 8.65E-10 | -1.05257 | 0.209471 | 5.04E-07 | 0.1125245 | 20 | 0.002950675 |
| cg22827724 | 6 | PSORS1C1 | 31096189 | ENSG00000196735 | 6 | HLA-DQA1 | 32595956 | rs28724021 | 6 | 32550695 | T | C | 0.229622 | -0.200441 | 0.0222111 | 1.81E-19 | -0.203287 | 0.0348862 | 5.64E-09 | 0.986 | 0.201417 | 9.82E-07 | 0.1055923 | 20 | 0.005749812 |
| cg02791903 | 6 | CCHCR1 | 31126284 | ENSG00000196735 | 6 | HLA-DQA1 | 32595956 | rs9272359 | 6 | 32604544 | T | C | 0.333002 | -0.235915 | 0.0229653 | 9.36E-25 | 0.214383 | 0.0341882 | 3.60E-10 | -1.10044 | 0.205601 | 8.68E-08 | 0.1954909 | 16 | 0.000508766 |
| cg01190171 | 6 | CCHCR1 | 31126373 | ENSG00000196735 | 6 | HLA-DQA1 | 32595956 | rs28707527 | 6 | 32587717 | A | G | 0.205765 | 0.165526 | 0.0304625 | 5.52E-08 | -0.401384 | 0.0516529 | 7.80E-15 | -0.412388 | 0.0926077 | 8.47E-06 | 0.3149472 | 20 | 0.04958947 |
| cg07400063 | 6 | CCHCR1 | 31126981 | ENSG00000196735 | 6 | HLA-DQA1 | 32595956 | rs1130455 | 6 | 32627405 | G | C | 0.224652 | 0.201446 | 0.0286026 | 1.88E-12 | 0.303872 | 0.0470557 | 1.06E-10 | 0.66293 | 0.139278 | 1.94E-06 | 0.1656921 | 20 | 0.01135421 |
| cg23679615 | 6 | TCF19 | 31127159 | ENSG00000196735 | 6 | HLA-DQA1 | 32595956 | rs72844390 | 6 | 32633436 | T | C | 0.222664 | 0.1746 | 0.0268769 | 8.23E-11 | 0.322572 | 0.0509678 | 2.47E-10 | 0.541275 | 0.119401 | 5.81E-06 | 0.127561 | 20 | 0.034026919 |
| cg21863888 | 6 | TCF19 | 31127173 | ENSG00000196735 | 6 | HLA-DQA1 | 32595956 | rs1130455 | 6 | 32627405 | G | C | 0.224652 | 0.201446 | 0.0286026 | 1.88E-12 | 0.270063 | 0.0467993 | 7.90E-09 | 0.745922 | 0.167109 | 8.06E-06 | 0.204406 | 20 | 0.04719377 |
| cg09045681 | 6 | TCF19 | 31127178 | ENSG00000196735 | 6 | HLA-DQA1 | 32595956 | rs1130455 | 6 | 32627405 | G | C | 0.224652 | 0.201446 | 0.0286026 | 1.88E-12 | 0.295812 | 0.0468907 | 2.82E-10 | 0.680993 | 0.144921 | 2.61E-06 | 0.3653672 | 20 | 0.015309315 |
| cg17974398 | 6 | HLA-C | 31239324 | ENSG00000196735 | 6 | HLA-DQA1 | 32595956 | rs9271774 | 6 | 32594309 | A | C | 0.22664 | 0.17647 | 0.0264335 | 2.46E-11 | -0.264654 | 0.0401984 | 4.59E-11 | -0.666795 | 0.142244 | 2.76E-06 | 0.2515037 | 20 | 0.016187353 |
| cg25355501 | 6 | HLA-C | 31239910 | ENSG00000196735 | 6 | HLA-DQA1 | 32595956 | rs9272348 | 6 | 32604396 | T | C | 0.204771 | -0.181727 | 0.024168 | 5.51E-14 | -0.251744 | 0.0390645 | 1.16E-10 | 0.721872 | 0.147527 | 9.92E-07 | 0.7786576 | 20 | 0.005813118 |
| cg05030953 | 6 | HLA-C | 31241000 | ENSG00000196735 | 6 | HLA-DQA1 | 32595956 | rs4478449 | 6 | 32571801 | A | G | 0.173956 | 0.188897 | 0.0309611 | 1.05E-09 | 0.486632 | 0.0412953 | 4.71E-32 | 0.388172 | 0.0716447 | 6.03E-08 | 0.1747921 | 20 | 0.000353032 |
| cg12259379 | 6 | MCCD1 | 31496949 | ENSG00000196735 | 6 | HLA-DQA1 | 32595956 | rs28533694 | 6 | 32587235 | T | C | 0.354871 | 0.258833 | 0.0233357 | 1.38E-28 | -0.266484 | 0.0390407 | 8.74E-12 | -0.971289 | 0.167083 | 6.13E-09 | 0.2408137 | 8 | 3.59E-05 |
| cg05945401 | 6 | BAT1 | 31506990 | ENSG00000196735 | 6 | HLA-DQA1 | 32595956 | rs9274617 | 6 | 32635904 | A | T | 0.379722 | -0.236152 | 0.0236386 | 1.68E-23 | -0.206065 | 0.0336829 | 9.49E-10 | 1.14601 | 0.219658 | 1.82E-07 | 0.1121193 | 20 | 0.001063829 |
| cg11489251 | 6 | BAT1 | 31508297 | ENSG00000196735 | 6 | HLA-DQA1 | 32595956 | rs2856698 | 6 | 32636376 | G | C | 0.281312 | -0.190412 | 0.023668 | 8.62E-16 | -0.340389 | 0.036113 | 4.27E-21 | 0.559395 | 0.0914163 | 9.40E-10 | 0.09675875 | 20 | 5.51E-06 |
| cg24631162 | 6 | BAT1 | 31508318 | ENSG00000196735 | 6 | HLA-DQA1 | 32595956 | rs2856698 | 6 | 32636376 | G | C | 0.281312 | -0.190412 | 0.023668 | 8.62E-16 | -0.38224 | 0.0357744 | 1.20E-26 | 0.498148 | 0.077509 | 1.30E-10 | 0.164794 | 20 | 7.63E-07 |
| cg01620360 | 6 | NFKBIL1 | 31526545 | ENSG00000196735 | 6 | HLA-DQA1 | 32595956 | rs115879259 | 6 | 32559213 | T | A | 0.121272 | -0.182548 | 0.0302417 | 1.58E-09 | 0.345236 | 0.0447179 | 1.16E-14 | -0.528763 | 0.111194 | 1.98E-06 | 0.0615823 | 20 | 0.011606883 |
| cg17709873 | 6 | LTA | 31540456 | ENSG00000196735 | 6 | HLA-DQA1 | 32595956 | rs2856698 | 6 | 32636376 | G | C | 0.281312 | -0.190412 | 0.023668 | 8.62E-16 | 0.21795 | 0.0291447 | 7.53E-14 | -0.87365 | 0.159502 | 4.32E-08 | 0.5304794 | 20 | 0.00025291 |
| cg19240857 | 6 | LY6G6E | 31683109 | ENSG00000196735 | 6 | HLA-DQA1 | 32595956 | rs582591 | 6 | 32579373 | A | G | 0.222664 | -0.174341 | 0.0250609 | 3.48E-12 | -0.293072 | 0.038063 | 1.36E-14 | 0.594874 | 0.115244 | 2.45E-07 | 0.1656414 | 20 | 0.001432295 |
| cg10189661 | 6 | LY6G6E | 31683120 | ENSG00000196735 | 6 | HLA-DQA1 | 32595956 | rs582591 | 6 | 32579373 | A | G | 0.222664 | -0.174341 | 0.0250609 | 3.48E-12 | -0.307966 | 0.0383355 | 9.48E-16 | 0.566105 | 0.107646 | 1.45E-07 | 0.1431801 | 20 | 0.000848824 |
| cg06132876 | 6 | C6orf25 | 31692080 | ENSG00000196735 | 6 | HLA-DQA1 | 32595956 | rs2856698 | 6 | 32636376 | G | C | 0.281312 | -0.190412 | 0.023668 | 8.62E-16 | -0.239465 | 0.0352381 | 1.08E-11 | 0.795156 | 0.153167 | 2.09E-07 | 0.2208928 | 20 | 0.001222442 |
| cg11093373 | 6 | CLIC1 | 31698899 | ENSG00000196735 | 6 | HLA-DQA1 | 32595956 | rs9272342 | 6 | 32604294 | A | G | 0.176938 | -0.186003 | 0.024422 | 2.61E-14 | -0.291157 | 0.0402303 | 4.58E-13 | 0.638841 | 0.121768 | 1.55E-07 | 0.06577214 | 20 | 0.000908715 |
| cg03392100 | 6 | C6orf26 | 31731881 | ENSG00000196735 | 6 | HLA-DQA1 | 32595956 | rs28707527 | 6 | 32587717 | A | G | 0.205765 | 0.165526 | 0.0304625 | 5.52E-08 | -0.547526 | 0.050148 | 9.43E-28 | -0.302316 | 0.0621461 | 1.15E-06 | 0.1426037 | 20 | 0.006718189 |
| cg13975172 | 6 | SLC44A4 | 31838675 | ENSG00000196735 | 6 | HLA-DQA1 | 32595956 | rs2647066 | 6 | 32571122 | T | C | 0.167992 | -0.184086 | 0.030145 | 1.02E-09 | 0.347388 | 0.040512 | 9.91E-18 | -0.529915 | 0.106532 | 6.55E-07 | 0.1599553 | 20 | 0.003837612 |
| cg02721751 | 6 | SLC44A4 | 31838684 | ENSG00000196735 | 6 | HLA-DQA1 | 32595956 | rs2647066 | 6 | 32571122 | T | C | 0.167992 | -0.184086 | 0.030145 | 1.02E-09 | 0.316907 | 0.0405568 | 5.54E-15 | -0.580883 | 0.120726 | 1.50E-06 | 0.1202312 | 20 | 0.008771096 |
| cg04567302 | 6 | SLC44A4 | 31846956 | ENSG00000196735 | 6 | HLA-DQA1 | 32595956 | rs9271147 | 6 | 32577385 | T | C | 0.135189 | 0.174228 | 0.0313636 | 2.77E-08 | -0.440408 | 0.0432081 | 2.14E-24 | -0.395606 | 0.0811047 | 1.07E-06 | 0.07215483 | 20 | 0.006286829 |
| cg03045620 | 6 | SLC44A4 | 31846970 | ENSG00000196735 | 6 | HLA-DQA1 | 32595956 | rs9271147 | 6 | 32577385 | T | C | 0.135189 | 0.174228 | 0.0313636 | 2.77E-08 | -0.413361 | 0.0438853 | 4.55E-21 | -0.421491 | 0.0880873 | 1.71E-06 | 0.08028006 | 20 | 0.010020226 |
| cg24707219 | 6 | SLC44A4 | 31847009 | ENSG00000196735 | 6 | HLA-DQA1 | 32595956 | rs4959030 | 6 | 32591751 | A | G | 0.117296 | 0.175864 | 0.0307547 | 1.08E-08 | -0.467273 | 0.0434049 | 5.01E-27 | -0.376362 | 0.0745262 | 4.42E-07 | 0.08879979 | 20 | 0.002587227 |
| cg16553272 | 6 | SLC44A4 | 31847028 | ENSG00000196735 | 6 | HLA-DQA1 | 32595956 | rs4959030 | 6 | 32591751 | A | G | 0.117296 | 0.175864 | 0.0307547 | 1.08E-08 | -0.568885 | 0.042606 | 1.15E-40 | -0.309138 | 0.0588105 | 1.47E-07 | 0.07823277 | 20 | 0.000860043 |
| cg25270367 | 6 | TNXB | 32022898 | ENSG00000196735 | 6 | HLA-DQA1 | 32595956 | rs9271726 | 6 | 32593566 | G | T | 0.178926 | -0.209903 | 0.0284226 | 1.52E-13 | -0.400937 | 0.0398292 | 7.78E-24 | 0.523531 | 0.0879219 | 2.61E-09 | 0.4584039 | 20 | 1.53E-05 |
| cg05598103 | 6 | TNXB | 32022929 | ENSG00000196735 | 6 | HLA-DQA1 | 32595956 | rs9271726 | 6 | 32593566 | G | T | 0.178926 | -0.209903 | 0.0284226 | 1.52E-13 | 0.318824 | 0.0401547 | 2.02E-15 | -0.658366 | 0.12175 | 6.39E-08 | 0.6278629 | 20 | 0.000374296 |
| cg16225663 | 6 | TNXB | 32026797 | ENSG00000196735 | 6 | HLA-DQA1 | 32595956 | rs9273040 | 6 | 32611958 | G | C | 0.276342 | -0.219128 | 0.0238029 | 3.39E-20 | 0.196341 | 0.0342147 | 9.55E-09 | -1.11606 | 0.229177 | 1.12E-06 | 0.3299082 | 20 | 0.006542361 |
| cg00122779 | 6 | TNXB | 32033006 | ENSG00000196735 | 6 | HLA-DQA1 | 32595956 | rs9271726 | 6 | 32593566 | G | T | 0.178926 | -0.209903 | 0.0284226 | 1.52E-13 | -0.698718 | 0.0373255 | 3.43E-78 | 0.300412 | 0.0437294 | 6.43E-12 | 0.1104999 | 20 | 3.77E-08 |
| cg07088771 | 6 | TNXB | 32057846 | ENSG00000196735 | 6 | HLA-DQA1 | 32595956 | rs2647066 | 6 | 32571122 | T | C | 0.167992 | -0.184086 | 0.030145 | 1.02E-09 | -0.545073 | 0.0400326 | 3.23E-42 | 0.337727 | 0.0606122 | 2.52E-08 | 0.08738824 | 20 | 0.000147578 |
| cg21337909 | 6 | TNXB | 32063459 | ENSG00000196735 | 6 | HLA-DQA1 | 32595956 | rs2856698 | 6 | 32636376 | G | C | 0.281312 | -0.190412 | 0.023668 | 8.62E-16 | -0.21899 | 0.03635 | 1.70E-09 | 0.869501 | 0.180309 | 1.42E-06 | 0.5912205 | 20 | 0.008314318 |
| cg26695758 | 6 | TNXB | 32063607 | ENSG00000196735 | 6 | HLA-DQA1 | 32595956 | rs582591 | 6 | 32579373 | A | G | 0.222664 | -0.174341 | 0.0250609 | 3.48E-12 | -0.360918 | 0.0386177 | 9.11E-21 | 0.483049 | 0.086561 | 2.40E-08 | 0.1333675 | 20 | 0.000140536 |
| cg02989255 | 6 | TNXB | 32063774 | ENSG00000196735 | 6 | HLA-DQA1 | 32595956 | rs583177 | 6 | 32579567 | A | C | 0.270378 | -0.155595 | 0.0240625 | 1.00E-10 | -0.2973 | 0.0368537 | 7.20E-16 | 0.52336 | 0.103729 | 4.52E-07 | 0.264544 | 20 | 0.002650405 |
| cg14188106 | 6 | TNXB | 32063895 | ENSG00000196735 | 6 | HLA-DQA1 | 32595956 | rs678770 | 6 | 32570817 | A | T | 0.222664 | -0.172386 | 0.0249996 | 5.37E-12 | -0.379255 | 0.0381479 | 2.74E-23 | 0.454539 | 0.0802215 | 1.46E-08 | 0.1281662 | 20 | 8.56E-05 |
| cg01992382 | 6 | TNXB | 32064212 | ENSG00000196735 | 6 | HLA-DQA1 | 32595956 | rs678770 | 6 | 32570817 | A | T | 0.222664 | -0.172386 | 0.0249996 | 5.37E-12 | -0.327982 | 0.0384581 | 1.49E-17 | 0.525596 | 0.0980208 | 8.23E-08 | 0.2364637 | 20 | 0.000481912 |
| cg16834823 | 6 | TNXB | 32064218 | ENSG00000196735 | 6 | HLA-DQA1 | 32595956 | rs678770 | 6 | 32570817 | A | T | 0.222664 | -0.172386 | 0.0249996 | 5.37E-12 | -0.3428 | 0.0386047 | 6.70E-19 | 0.502876 | 0.0923342 | 5.14E-08 | 0.209403 | 20 | 0.000301326 |
| cg00525277 | 6 | TNXB | 32064239 | ENSG00000196735 | 6 | HLA-DQA1 | 32595956 | rs678770 | 6 | 32570817 | A | T | 0.222664 | -0.172386 | 0.0249996 | 5.37E-12 | -0.329685 | 0.038484 | 1.06E-17 | 0.522881 | 0.0973414 | 7.80E-08 | 0.1789699 | 20 | 0.000457109 |
| cg10890302 | 6 | TNXB | 32064246 | ENSG00000196735 | 6 | HLA-DQA1 | 32595956 | rs678770 | 6 | 32570817 | A | T | 0.222664 | -0.172386 | 0.0249996 | 5.37E-12 | -0.308613 | 0.0385141 | 1.12E-15 | 0.558583 | 0.106871 | 1.73E-07 | 0.1914837 | 20 | 0.001010893 |
| cg10923662 | 6 | TNXB | 32064258 | ENSG00000196735 | 6 | HLA-DQA1 | 32595956 | rs678770 | 6 | 32570817 | A | T | 0.222664 | -0.172386 | 0.0249996 | 5.37E-12 | -0.340809 | 0.0384524 | 7.78E-19 | 0.505814 | 0.0929391 | 5.26E-08 | 0.1712313 | 20 | 0.000307894 |
| cg21241317 | 6 | PPT2 | 32122736 | ENSG00000196735 | 6 | HLA-DQA1 | 32595956 | rs9270885 | 6 | 32571771 | G | A | 0.33996 | 0.220669 | 0.0253605 | 3.28E-18 | -0.283582 | 0.0348032 | 3.70E-16 | -0.778149 | 0.130835 | 2.72E-09 | 0.188252 | 20 | 1.59E-05 |
| cg02681370 | 6 | PBX2 | 32156482 | ENSG00000196735 | 6 | HLA-DQA1 | 32595956 | rs9270809 | 6 | 32569608 | G | A | 0.265408 | 0.223171 | 0.025459 | 1.85E-18 | -0.203754 | 0.0357569 | 1.21E-08 | -1.0953 | 0.229257 | 1.77E-06 | 0.0761087 | 20 | 0.010392274 |
| cg03280235 | 6 | PBX2 | 32158953 | ENSG00000196735 | 6 | HLA-DQA1 | 32595956 | rs9272359 | 6 | 32604544 | T | C | 0.333002 | -0.235915 | 0.0229653 | 9.36E-25 | -0.179672 | 0.0252481 | 1.11E-12 | 1.31303 | 0.224459 | 4.92E-09 | 0.3632163 | 20 | 2.88E-05 |
| cg11884274 | 6 | NOTCH4 | 32164927 | ENSG00000196735 | 6 | HLA-DQA1 | 32595956 | rs2858864 | 6 | 32578229 | T | C | 0.26839 | -0.151663 | 0.0241201 | 3.22E-10 | -0.317838 | 0.0368877 | 6.91E-18 | 0.477171 | 0.0939461 | 3.79E-07 | 0.07126046 | 20 | 0.002220371 |
| cg08801479 | 6 | NOTCH4 | 32165200 | ENSG00000196735 | 6 | HLA-DQA1 | 32595956 | rs678770 | 6 | 32570817 | A | T | 0.222664 | -0.172386 | 0.0249996 | 5.37E-12 | -0.322176 | 0.0383269 | 4.24E-17 | 0.535068 | 0.100364 | 9.75E-08 | 0.1238949 | 20 | 0.000571248 |
| cg14614539 | 6 | NOTCH4 | 32170458 | ENSG00000196735 | 6 | HLA-DQA1 | 32595956 | rs9272348 | 6 | 32604396 | T | C | 0.204771 | -0.181727 | 0.024168 | 5.51E-14 | -0.33725 | 0.0392929 | 9.25E-18 | 0.53885 | 0.0952728 | 1.55E-08 | 0.1044025 | 20 | 9.08E-05 |
| cg07637554 | 6 | NOTCH4 | 32179971 | ENSG00000196735 | 6 | HLA-DQA1 | 32595956 | rs9271634 | 6 | 32592112 | A | G | 0.210736 | 0.175902 | 0.0265707 | 3.59E-11 | -0.323888 | 0.0401562 | 7.28E-16 | -0.543095 | 0.106132 | 3.10E-07 | 0.0581116 | 20 | 0.001816682 |
| cg09198277 | 6 | NOTCH4 | 32187662 | ENSG00000196735 | 6 | HLA-DQA1 | 32595956 | rs9270902 | 6 | 32571962 | A | G | 0.305169 | 0.222566 | 0.0253446 | 1.61E-18 | -0.27382 | 0.0348717 | 4.09E-15 | -0.812819 | 0.138862 | 4.81E-09 | 0.09048322 | 20 | 2.82E-05 |
| cg25317746 | 6 | NOTCH4 | 32192024 | ENSG00000196735 | 6 | HLA-DQA1 | 32595956 | rs9271463 | 6 | 32588535 | A | T | 0.398608 | 0.192256 | 0.0237121 | 5.15E-16 | 0.238374 | 0.0339729 | 2.27E-12 | 0.806531 | 0.152013 | 1.12E-07 | 0.09639872 | 20 | 0.000657573 |
| cg25949002 | 6 | C6orf10 | 32301073 | ENSG00000196735 | 6 | HLA-DQA1 | 32595956 | rs2647062 | 6 | 32570417 | C | A | 0.147117 | -0.184086 | 0.030145 | 1.02E-09 | -0.50541 | 0.0405172 | 1.04E-35 | 0.364231 | 0.0664084 | 4.14E-08 | 0.1035398 | 20 | 0.000242609 |
| cg02808240 | 6 | C6orf10 | 32304143 | ENSG00000196735 | 6 | HLA-DQA1 | 32595956 | rs2647062 | 6 | 32570417 | C | A | 0.147117 | -0.184086 | 0.030145 | 1.02E-09 | -0.463792 | 0.0407989 | 6.05E-30 | 0.396915 | 0.0737815 | 7.47E-08 | 0.1544478 | 20 | 0.000437301 |
| cg09452510 | 6 | C6orf10 | 32330188 | ENSG00000196735 | 6 | HLA-DQA1 | 32595956 | rs2760980 | 6 | 32565201 | A | G | 0.148111 | -0.184086 | 0.030145 | 1.02E-09 | -0.437222 | 0.03952 | 1.89E-28 | 0.421036 | 0.0787526 | 8.98E-08 | 0.08972938 | 20 | 0.000525854 |
| cg25605800 | 6 | C6orf10 | 32333231 | ENSG00000196735 | 6 | HLA-DQA1 | 32595956 | rs114020256 | 6 | 32601797 | A | G | 0.296223 | 0.245706 | 0.0235472 | 1.72E-25 | 0.296715 | 0.0409327 | 4.20E-13 | 0.828088 | 0.139097 | 2.63E-09 | 0.05985675 | 20 | 1.54E-05 |
| cg22863148 | 6 | C6orf10 | 32339554 | ENSG00000196735 | 6 | HLA-DQA1 | 32595956 | rs72492305 | 6 | 32509333 | C | T | 0.342942 | 0.259353 | 0.0218115 | 1.32E-32 | 0.19684 | 0.0336762 | 5.06E-09 | 1.31758 | 0.25118 | 1.56E-07 | 0.0990943 | 20 | 0.000912681 |
| cg04970287 | 6 | BTNL2 | 32362638 | ENSG00000196735 | 6 | HLA-DQA1 | 32595956 | rs115690055 | 6 | 32359521 | T | C | 0.159046 | 0.171121 | 0.0306802 | 2.44E-08 | -1.8625 | 0.0241152 | 0 | -0.0918771 | 0.0165155 | 2.65E-08 | 0.2377974 | 20 | 0.000155262 |
| cg03036047 | 6 | BTNL2 | 32362744 | ENSG00000196735 | 6 | HLA-DQA1 | 32595956 | rs115690055 | 6 | 32359521 | T | C | 0.159046 | 0.171121 | 0.0306802 | 2.44E-08 | -1.61709 | 0.0341642 | 0 | -0.10582 | 0.0191037 | 3.04E-08 | 0.1594574 | 20 | 0.000177948 |
| cg19117063 | 6 | BTNL2 | 32365553 | ENSG00000196735 | 6 | HLA-DQA1 | 32595956 | rs116454630 | 6 | 32359519 | A | T | 0.16004 | 0.170117 | 0.0316118 | 7.39E-08 | -1.5911 | 0.0359783 | 0 | -0.106918 | 0.0200145 | 9.19E-08 | 0.8087065 | 20 | 0.00053842 |
| cg18996355 | 6 | BTNL2 | 32366347 | ENSG00000196735 | 6 | HLA-DQA1 | 32595956 | rs115690055 | 6 | 32359521 | T | C | 0.159046 | 0.171121 | 0.0306802 | 2.44E-08 | -0.734926 | 0.0466399 | 6.11E-56 | -0.232841 | 0.044284 | 1.46E-07 | 0.3172287 | 20 | 0.000853525 |
| cg14241129 | 6 | BTNL2 | 32367729 | ENSG00000196735 | 6 | HLA-DQA1 | 32595956 | rs9273040 | 6 | 32611958 | G | C | 0.276342 | -0.219128 | 0.0238029 | 3.39E-20 | 0.363591 | 0.033706 | 3.96E-27 | -0.602677 | 0.0860656 | 2.51E-12 | 0.1225299 | 20 | 1.47E-08 |
| cg16004593 | 6 | BTNL2 | 32369487 | ENSG00000196735 | 6 | HLA-DQA1 | 32595956 | rs7760841 | 6 | 32574868 | T | C | 0.115308 | -0.185426 | 0.0295456 | 3.48E-10 | -0.643691 | 0.0429776 | 1.03E-50 | 0.288067 | 0.0497671 | 7.11E-09 | 0.1691514 | 20 | 4.17E-05 |
| cg00331257 | 6 | BTNL2 | 32371538 | ENSG00000196735 | 6 | HLA-DQA1 | 32595956 | rs28724021 | 6 | 32550695 | T | C | 0.229622 | -0.200441 | 0.0222111 | 1.81E-19 | 0.188609 | 0.0343045 | 3.84E-08 | -1.06273 | 0.22634 | 2.66E-06 | 0.2069151 | 20 | 0.015594922 |
| cg15011943 | 6 | HLA-DRB5 | 32493917 | ENSG00000196735 | 6 | HLA-DQA1 | 32595956 | rs28366317 | 6 | 32561411 | T | C | 0.307157 | -0.215967 | 0.0241004 | 3.21E-19 | 0.721876 | 0.030815 | 2.31E-121 | -0.299175 | 0.0357451 | 5.78E-17 | 0.2546954 | 20 | 3.38E-13 |
| cg06204447 | 6 | HLA-DRB1 | 32546665 | ENSG00000196735 | 6 | HLA-DQA1 | 32595956 | rs9271464 | 6 | 32588544 | T | A | 0.284294 | -0.218651 | 0.0237952 | 3.97E-20 | -1.04665 | 0.0271458 | 0 | 0.208906 | 0.0233713 | 3.94E-19 | 0.3356563 | 20 | 2.31E-15 |
| cg13778567 | 6 | HLA-DQA1 | 32609783 | ENSG00000196735 | 6 | HLA-DQA1 | 32595956 | rs28366319 | 6 | 32561495 | A | G | 0.300199 | -0.215589 | 0.0240877 | 3.55E-19 | 1.09606 | 0.0248368 | 0 | -0.196695 | 0.0224241 | 1.76E-18 | 0.2835964 | 20 | 1.03E-14 |
| cg10846853 | 6 | HLA-DQB1 | 32632859 | ENSG00000196735 | 6 | HLA-DQA1 | 32595956 | rs145281025 | 6 | 32674252 | A | G | 0.136183 | 0.171035 | 0.0335323 | 3.39E-07 | 0.771572 | 0.0533161 | 1.83E-47 | 0.221671 | 0.04608 | 1.51E-06 | 0.3471741 | 20 | 0.008816753 |
| cg02293354 | 6 | HLA-DQA2 | 32711008 | ENSG00000196735 | 6 | HLA-DQA1 | 32595956 | rs9271430 | 6 | 32588002 | C | T | 0.303181 | -0.216247 | 0.0237375 | 8.24E-20 | -0.242383 | 0.0335999 | 5.44E-13 | 0.892171 | 0.157755 | 1.55E-08 | 0.2757681 | 20 | 9.11E-05 |
| cg14740554 | 6 | HLA-DQA2 | 32713607 | ENSG00000196735 | 6 | HLA-DQA1 | 32595956 | rs9271523 | 6 | 32589806 | G | A | 0.293241 | -0.212627 | 0.0235014 | 1.46E-19 | 0.572868 | 0.0318429 | 2.31E-72 | -0.371162 | 0.0459197 | 6.33E-16 | 0.4814768 | 20 | 3.71E-12 |
| cg22812614 | 6 | HLA-DQA2 | 32713827 | ENSG00000196735 | 6 | HLA-DQA1 | 32595956 | rs9271464 | 6 | 32588544 | T | A | 0.284294 | -0.218651 | 0.0237952 | 3.97E-20 | -1.14986 | 0.0242236 | 0 | 0.190154 | 0.0210781 | 1.86E-19 | 0.3189458 | 20 | 1.09E-15 |
| cg20985082 | 6 | HLA-DQB2 | 32727265 | ENSG00000196735 | 6 | HLA-DQA1 | 32595956 | rs9271517 | 6 | 32589740 | G | A | 0.279324 | -0.216965 | 0.023704 | 5.53E-20 | -0.248133 | 0.033876 | 2.39E-13 | 0.87439 | 0.152893 | 1.07E-08 | 0.4772812 | 20 | 6.28E-05 |
| cg09739413 | 6 | HLA-DQB2 | 32729498 | ENSG00000196735 | 6 | HLA-DQA1 | 32595956 | rs582591 | 6 | 32579373 | A | G | 0.222664 | -0.174341 | 0.0250609 | 3.48E-12 | -0.237936 | 0.0389474 | 1.00E-09 | 0.732722 | 0.159621 | 4.42E-06 | 0.3679697 | 12 | 0.02591629 |
| cg14441480 | 6 | HLA-DQB2 | 32729500 | ENSG00000196735 | 6 | HLA-DQA1 | 32595956 | rs9272314 | 6 | 32604037 | G | C | 0.251491 | -0.182267 | 0.0243453 | 7.06E-14 | -0.238378 | 0.0389157 | 9.04E-10 | 0.764613 | 0.161281 | 2.13E-06 | 0.4042506 | 16 | 0.012465566 |
| cg02964065 | 6 | HLA-DQB2 | 32729545 | ENSG00000196735 | 6 | HLA-DQA1 | 32595956 | rs678770 | 6 | 32570817 | A | T | 0.222664 | -0.172386 | 0.0249996 | 5.37E-12 | -0.316788 | 0.0387444 | 2.93E-16 | 0.544168 | 0.103233 | 1.36E-07 | 0.3721556 | 20 | 0.000793764 |
| cg15255946 | 6 | HLA-DQB2 | 32729563 | ENSG00000196735 | 6 | HLA-DQA1 | 32595956 | rs2856698 | 6 | 32636376 | G | C | 0.281312 | -0.190412 | 0.023668 | 8.62E-16 | -0.296625 | 0.0357503 | 1.07E-16 | 0.641928 | 0.111141 | 7.66E-09 | 0.1412088 | 20 | 4.49E-05 |
| cg18026055 | 6 | HLA-DQB2 | 32729596 | ENSG00000196735 | 6 | HLA-DQA1 | 32595956 | rs2856698 | 6 | 32636376 | G | C | 0.281312 | -0.190412 | 0.023668 | 8.62E-16 | -0.250012 | 0.0359566 | 3.57E-12 | 0.761611 | 0.144775 | 1.44E-07 | 0.4389446 | 20 | 0.00084086 |
| cg02010152 | 6 | HLA-DOB | 32782617 | ENSG00000196735 | 6 | HLA-DQA1 | 32595956 | rs147804272 | 6 | 32662823 | T | C | 0.137177 | 0.176451 | 0.0335225 | 1.41E-07 | 0.491064 | 0.0526165 | 1.03E-20 | 0.359324 | 0.0783737 | 4.55E-06 | 0.0659706 | 20 | 0.026626531 |
| cg10850215 | 6 | HLA-DPB1 | 33048469 | ENSG00000196735 | 6 | HLA-DQA1 | 32595956 | rs114567049 | 6 | 32601719 | A | G | 0.244533 | 0.258826 | 0.0245172 | 4.72E-26 | 0.233332 | 0.0418031 | 2.38E-08 | 1.10926 | 0.2248 | 8.04E-07 | 0.53093 | 9 | 0.004708884 |
| cg14782559 | 6 | COL11A2 | 33131893 | ENSG00000196735 | 6 | HLA-DQA1 | 32595956 | rs80343249 | 6 | 32580268 | A | G | 0.0377734 | 0.399403 | 0.052971 | 4.70E-14 | -0.477988 | 0.0858087 | 2.54E-08 | -0.835592 | 0.186502 | 7.45E-06 | 0.4245346 | 3 | 0.043657729 |
| cg16990009 | 6 | GTF2H4 | 30880155 | ENSG00000179344 | 6 | HLA-DQB1 | 32627244 | rs9267658 | 6 | 31845985 | T | C | 0.104374 | 0.403673 | 0.0529302 | 2.41E-14 | -0.3189 | 0.0436432 | 2.73E-13 | -1.26583 | 0.239915 | 1.32E-07 | 0.2570782 | 6 | 0.000772772 |
| cg26467571 | 6 | VARS2 | 30882355 | ENSG00000179344 | 6 | HLA-DQB1 | 32627244 | rs1269852 | 6 | 32080191 | C | G | 0.0755467 | -0.434887 | 0.0642446 | 1.29E-11 | 0.303727 | 0.0465145 | 6.59E-11 | -1.43184 | 0.304671 | 2.61E-06 | 0.2694688 | 10 | 0.015269685 |
| cg23201206 | 6 | VARS2;VARS2 | 30885800 | ENSG00000179344 | 6 | HLA-DQB1 | 32627244 | rs2647066 | 6 | 32571122 | T | C | 0.167992 | -0.326737 | 0.0470939 | 3.98E-12 | 0.305674 | 0.0404803 | 4.31E-14 | -1.06891 | 0.209223 | 3.24E-07 | 0.1025917 | 20 | 0.001897762 |
| cg17784811 | 6 | C6orf15 | 31079617 | ENSG00000179344 | 6 | HLA-DQB1 | 32627244 | rs115879259 | 6 | 32559213 | T | A | 0.121272 | -0.323409 | 0.0472536 | 7.70E-12 | -0.270628 | 0.0451059 | 1.98E-09 | 1.19503 | 0.264876 | 6.43E-06 | 0.1535688 | 20 | 0.037678867 |
| cg22827724 | 6 | PSORS1C1 | 31096189 | ENSG00000179344 | 6 | HLA-DQB1 | 32627244 | rs28724021 | 6 | 32550695 | T | C | 0.229622 | -0.373768 | 0.0343347 | 1.34E-27 | -0.203287 | 0.0348862 | 5.64E-09 | 1.83862 | 0.357888 | 2.79E-07 | 0.05290151 | 20 | 0.001631607 |
| cg02247838 | 6 | CCHCR1 | 31110639 | ENSG00000179344 | 6 | HLA-DQB1 | 32627244 | rs116667074 | 6 | 32285362 | T | C | 0.0785288 | -0.43495 | 0.0630726 | 5.35E-12 | -0.409891 | 0.047046 | 2.97E-18 | 1.06114 | 0.196244 | 6.40E-08 | 0.06670105 | 20 | 0.000374967 |
| cg01140214 | 6 | CCHCR1 | 31110647 | ENSG00000179344 | 6 | HLA-DQB1 | 32627244 | rs9270868 | 6 | 32571117 | G | A | 0.180915 | -0.402879 | 0.0388566 | 3.45E-25 | -0.260902 | 0.039331 | 3.28E-11 | 1.54418 | 0.27635 | 2.30E-08 | 0.07584237 | 20 | 0.000134749 |
| cg01190171 | 6 | CCHCR1 | 31126373 | ENSG00000179344 | 6 | HLA-DQB1 | 32627244 | rs68033958 | 6 | 32634226 | A | G | 0.196819 | 0.370725 | 0.0440204 | 3.71E-17 | -0.385301 | 0.050787 | 3.28E-14 | -0.96217 | 0.170697 | 1.73E-08 | 0.07188278 | 20 | 0.000101536 |
| cg21863888 | 6 | TCF19 | 31127173 | ENSG00000179344 | 6 | HLA-DQB1 | 32627244 | rs1130455 | 6 | 32627405 | G | C | 0.224652 | 0.392693 | 0.0443587 | 8.54E-19 | 0.270063 | 0.0467993 | 7.90E-09 | 1.45408 | 0.300785 | 1.34E-06 | 0.05338501 | 20 | 0.00782811 |
| cg09045681 | 6 | TCF19 | 31127178 | ENSG00000179344 | 6 | HLA-DQB1 | 32627244 | rs1130455 | 6 | 32627405 | G | C | 0.224652 | 0.392693 | 0.0443587 | 8.54E-19 | 0.295812 | 0.0468907 | 2.82E-10 | 1.32751 | 0.258394 | 2.78E-07 | 0.05844087 | 20 | 0.001630698 |
| cg09329266 | 6 | TCF19 | 31127357 | ENSG00000179344 | 6 | HLA-DQB1 | 32627244 | rs68081734 | 6 | 32628828 | C | T | 0.235586 | 0.31252 | 0.0392716 | 1.75E-15 | 0.276508 | 0.0469992 | 4.02E-09 | 1.13024 | 0.238911 | 2.24E-06 | 0.0609624 | 20 | 0.01310106 |
| cg26504835 | 6 | PSORS1C3 | 31143710 | ENSG00000179344 | 6 | HLA-DQB1 | 32627244 | rs28383182 | 6 | 32571811 | A | G | 0.265408 | -0.381536 | 0.0375305 | 2.81E-24 | 0.192518 | 0.0342847 | 1.96E-08 | -1.98182 | 0.403195 | 8.86E-07 | 0.05313987 | 20 | 0.005193091 |
| cg23536255 | 6 | NA | 31276105 | ENSG00000179344 | 6 | HLA-DQB1 | 32627244 | rs9267658 | 6 | 31845985 | T | C | 0.104374 | 0.403673 | 0.0529302 | 2.41E-14 | -0.274201 | 0.0447869 | 9.22E-10 | -1.47218 | 0.308356 | 1.80E-06 | 0.8867949 | 6 | 0.010564458 |
| cg06414921 | 6 | NA | 31276504 | ENSG00000179344 | 6 | HLA-DQB1 | 32627244 | rs9267658 | 6 | 31845985 | T | C | 0.104374 | 0.403673 | 0.0529302 | 2.41E-14 | -0.268497 | 0.0447627 | 1.99E-09 | -1.50345 | 0.318885 | 2.42E-06 | 0.1096823 | 13 | 0.014178346 |
| cg16655385 | 6 | NA | 31334836 | ENSG00000179344 | 6 | HLA-DQB1 | 32627244 | rs116259801 | 6 | 32592649 | G | T | 0.182903 | 0.363912 | 0.0475048 | 1.85E-14 | 0.358553 | 0.0519592 | 5.18E-12 | 1.01495 | 0.197955 | 2.94E-07 | 0.05158808 | 20 | 0.001723026 |
| cg26990733 | 6 | MICA | 31371465 | ENSG00000179344 | 6 | HLA-DQB1 | 32627244 | rs9267658 | 6 | 31845985 | T | C | 0.104374 | 0.403673 | 0.0529302 | 2.41E-14 | 0.25719 | 0.0432921 | 2.84E-09 | 1.56955 | 0.334896 | 2.78E-06 | 0.4977322 | 4 | 0.016265838 |
| cg11268327 | 6 | MICA | 31380198 | ENSG00000179344 | 6 | HLA-DQB1 | 32627244 | rs34810547 | 6 | 32590184 | T | C | 0.182903 | 0.363912 | 0.0475048 | 1.85E-14 | -0.515805 | 0.0474404 | 1.56E-27 | -0.705522 | 0.112662 | 3.79E-10 | 0.05595979 | 20 | 2.22E-06 |
| cg25843003 | 6 | HCP5 | 31431312 | ENSG00000179344 | 6 | HLA-DQB1 | 32627244 | rs3891175 | 6 | 32634467 | T | C | 0.170974 | -0.459223 | 0.0429687 | 1.17E-26 | 0.234762 | 0.0375559 | 4.08E-10 | -1.95612 | 0.362525 | 6.82E-08 | 0.2028353 | 17 | 0.000399587 |
| cg25420482 | 6 | NFKBIL1 | 31519182 | ENSG00000179344 | 6 | HLA-DQB1 | 32627244 | rs9274623 | 6 | 32635998 | T | G | 0.202783 | -0.45393 | 0.043532 | 1.86E-25 | 0.248143 | 0.0377295 | 4.80E-11 | -1.82931 | 0.328845 | 2.65E-08 | 0.09840801 | 17 | 0.000155487 |
| cg26333342 | 6 | NFKBIL1 | 31519394 | ENSG00000179344 | 6 | HLA-DQB1 | 32627244 | rs9274623 | 6 | 32635998 | T | G | 0.202783 | -0.45393 | 0.043532 | 1.86E-25 | 0.246366 | 0.0379918 | 8.89E-11 | -1.8425 | 0.334591 | 3.66E-08 | 0.2465675 | 13 | 0.000214153 |
| cg25403205 | 6 | AIF1 | 31584215 | ENSG00000179344 | 6 | HLA-DQB1 | 32627244 | rs1048709 | 6 | 31914935 | A | G | 0.152087 | 0.339247 | 0.0453277 | 7.19E-14 | 0.290178 | 0.0398663 | 3.37E-13 | 1.1691 | 0.22405 | 1.81E-07 | 0.6098178 | 6 | 0.001059376 |
| cg12608306 | 6 | BAT4 | 31632740 | ENSG00000179344 | 6 | HLA-DQB1 | 32627244 | rs9270980 | 6 | 32573909 | C | A | 0.137177 | 0.555481 | 0.0465516 | 8.00E-33 | 0.273005 | 0.0415586 | 5.06E-11 | 2.03469 | 0.353569 | 8.68E-09 | 0.2129367 | 20 | 5.08E-05 |
| cg19240857 | 6 | LY6G6E | 31683109 | ENSG00000179344 | 6 | HLA-DQB1 | 32627244 | rs1269852 | 6 | 32080191 | C | G | 0.0755467 | -0.434887 | 0.0642446 | 1.29E-11 | -0.393056 | 0.0466625 | 3.66E-17 | 1.10643 | 0.209687 | 1.32E-07 | 0.08597812 | 20 | 0.000771164 |
| cg10189661 | 6 | LY6G6E | 31683120 | ENSG00000179344 | 6 | HLA-DQB1 | 32627244 | rs1269852 | 6 | 32080191 | C | G | 0.0755467 | -0.434887 | 0.0642446 | 1.29E-11 | -0.406831 | 0.046791 | 3.48E-18 | 1.06896 | 0.200131 | 9.23E-08 | 0.06795918 | 20 | 0.000540567 |
| cg20288341 | 6 | LY6G6E | 31683131 | ENSG00000179344 | 6 | HLA-DQB1 | 32627244 | rs1269852 | 6 | 32080191 | C | G | 0.0755467 | -0.434887 | 0.0642446 | 1.29E-11 | -0.388125 | 0.0471017 | 1.72E-16 | 1.12048 | 0.214217 | 1.69E-07 | 0.09419842 | 20 | 0.000989785 |
| cg19127747 | 6 | C6orf25 | 31691750 | ENSG00000179344 | 6 | HLA-DQB1 | 32627244 | rs1150757 | 6 | 32029205 | A | G | 0.0755467 | -0.434887 | 0.0642446 | 1.29E-11 | -0.287505 | 0.0470841 | 1.02E-09 | 1.51262 | 0.333612 | 5.79E-06 | 0.09687054 | 13 | 0.033892273 |
| cg01338864 | 6 | C6orf25 | 31691905 | ENSG00000179344 | 6 | HLA-DQB1 | 32627244 | rs433061 | 6 | 32014828 | A | G | 0.0735587 | -0.434887 | 0.0642446 | 1.29E-11 | -0.334907 | 0.0466682 | 7.16E-13 | 1.29853 | 0.263703 | 8.47E-07 | 0.07598839 | 20 | 0.004961856 |
| cg24108149 | 6 | DDAH2 | 31697559 | ENSG00000179344 | 6 | HLA-DQB1 | 32627244 | rs693906 | 6 | 31835164 | C | G | 0.11332 | -0.325137 | 0.0533969 | 1.14E-09 | -0.281874 | 0.0431613 | 6.55E-11 | 1.15348 | 0.259002 | 8.45E-06 | 0.08773248 | 11 | 0.049472866 |
| cg26346875 | 6 | VARS | 31762719 | ENSG00000179344 | 6 | HLA-DQB1 | 32627244 | rs9267658 | 6 | 31845985 | T | C | 0.104374 | 0.403673 | 0.0529302 | 2.41E-14 | 0.264478 | 0.0442579 | 2.29E-09 | 1.5263 | 0.32448 | 2.55E-06 | 0.3283631 | 4 | 0.014957003 |
| cg17494781 | 6 | HSPA1A | 31783482 | ENSG00000179344 | 6 | HLA-DQB1 | 32627244 | rs433061 | 6 | 32014828 | A | G | 0.0735587 | -0.434887 | 0.0642446 | 1.29E-11 | -0.336002 | 0.0468421 | 7.33E-13 | 1.2943 | 0.2629 | 8.52E-07 | 0.1114625 | 20 | 0.004988698 |
| cg00397479 | 6 | NEU1 | 31831510 | ENSG00000179344 | 6 | HLA-DQB1 | 32627244 | rs433061 | 6 | 32014828 | A | G | 0.0735587 | -0.434887 | 0.0642446 | 1.29E-11 | -0.307809 | 0.0464238 | 3.35E-11 | 1.41285 | 0.298275 | 2.17E-06 | 0.09757894 | 10 | 0.012723213 |
| cg04293778 | 6 | TNXB | 32016426 | ENSG00000179344 | 6 | HLA-DQB1 | 32627244 | rs1150753 | 6 | 32059867 | G | A | 0.0755467 | -0.434887 | 0.0642446 | 1.29E-11 | -0.394892 | 0.0463772 | 1.67E-17 | 1.10128 | 0.207836 | 1.17E-07 | 0.07903423 | 20 | 0.000682878 |
| cg23164535 | 6 | TNXB | 32044869 | ENSG00000179344 | 6 | HLA-DQB1 | 32627244 | rs3130286 | 6 | 32042322 | T | C | 0.173956 | 0.272313 | 0.0407203 | 2.27E-11 | 0.276685 | 0.0373219 | 1.23E-13 | 0.984199 | 0.198203 | 6.85E-07 | 0.2094533 | 4 | 0.004011789 |
| cg21337909 | 6 | TNXB | 32063459 | ENSG00000179344 | 6 | HLA-DQB1 | 32627244 | rs2856698 | 6 | 32636376 | G | C | 0.281312 | -0.449209 | 0.0357711 | 3.60E-36 | -0.21899 | 0.03635 | 1.70E-09 | 2.05128 | 0.377645 | 5.58E-08 | 0.1291943 | 20 | 0.000326877 |
| cg04286337 | 6 | TNXB | 32063501 | ENSG00000179344 | 6 | HLA-DQB1 | 32627244 | rs2760994 | 6 | 32574308 | C | T | 0.405567 | 0.313766 | 0.0353342 | 6.69E-19 | 0.229127 | 0.0329766 | 3.70E-12 | 1.3694 | 0.25025 | 4.45E-08 | 0.07596786 | 20 | 0.000260495 |
| cg18460422 | 6 | TNXB | 32063553 | ENSG00000179344 | 6 | HLA-DQB1 | 32627244 | rs6933289 | 6 | 32604551 | T | C | 0.0715706 | -0.466806 | 0.0537357 | 3.72E-18 | -0.338749 | 0.04543 | 8.88E-14 | 1.37803 | 0.243552 | 1.53E-08 | 0.1375089 | 20 | 8.97E-05 |
| cg14196170 | 6 | TNXB | 32063595 | ENSG00000179344 | 6 | HLA-DQB1 | 32627244 | rs6933289 | 6 | 32604551 | T | C | 0.0715706 | -0.466806 | 0.0537357 | 3.72E-18 | -0.344152 | 0.0455658 | 4.26E-14 | 1.35639 | 0.237973 | 1.20E-08 | 0.2331122 | 20 | 7.03E-05 |
| cg04753078 | 6 | TNXB | 32063619 | ENSG00000179344 | 6 | HLA-DQB1 | 32627244 | rs6933289 | 6 | 32604551 | T | C | 0.0715706 | -0.466806 | 0.0537357 | 3.72E-18 | -0.410289 | 0.0453394 | 1.44E-19 | 1.13775 | 0.181551 | 3.68E-10 | 0.4040619 | 20 | 2.16E-06 |
| cg00779476 | 6 | TNXB | 32063726 | ENSG00000179344 | 6 | HLA-DQB1 | 32627244 | rs1270942 | 6 | 31918860 | G | A | 0.0725646 | -0.410305 | 0.0637993 | 1.27E-10 | -0.360455 | 0.0470893 | 1.94E-14 | 1.1383 | 0.231173 | 8.48E-07 | 0.1141807 | 20 | 0.004967252 |
| cg01337207 | 6 | TNXB | 32063835 | ENSG00000179344 | 6 | HLA-DQB1 | 32627244 | rs1150753 | 6 | 32059867 | G | A | 0.0755467 | -0.434887 | 0.0642446 | 1.29E-11 | -0.427682 | 0.0465025 | 3.68E-20 | 1.01685 | 0.186518 | 4.99E-08 | 0.08729522 | 20 | 0.000292207 |
| cg26266427 | 6 | TNXB | 32063838 | ENSG00000179344 | 6 | HLA-DQB1 | 32627244 | rs433061 | 6 | 32014828 | A | G | 0.0735587 | -0.434887 | 0.0642446 | 1.29E-11 | -0.429055 | 0.0465627 | 3.13E-20 | 1.01359 | 0.185797 | 4.89E-08 | 0.08845728 | 20 | 0.000286257 |
| cg10365886 | 6 | TNXB | 32063874 | ENSG00000179344 | 6 | HLA-DQB1 | 32627244 | rs1270942 | 6 | 31918860 | G | A | 0.0725646 | -0.410305 | 0.0637993 | 1.27E-10 | -0.423522 | 0.04665 | 1.10E-19 | 0.968793 | 0.184606 | 1.54E-07 | 0.06397238 | 20 | 0.000901284 |
| cg14188106 | 6 | TNXB | 32063895 | ENSG00000179344 | 6 | HLA-DQB1 | 32627244 | rs678770 | 6 | 32570817 | A | T | 0.222664 | -0.431837 | 0.0379437 | 5.20E-30 | -0.379255 | 0.0381479 | 2.74E-23 | 1.13865 | 0.152076 | 7.03E-14 | 0.141003 | 20 | 4.12E-10 |
| cg07524919 | 6 | TNXB | 32063901 | ENSG00000179344 | 6 | HLA-DQB1 | 32627244 | rs1270942 | 6 | 31918860 | G | A | 0.0725646 | -0.410305 | 0.0637993 | 1.27E-10 | -0.44475 | 0.0466635 | 1.56E-21 | 0.922552 | 0.173052 | 9.76E-08 | 0.0578504 | 20 | 0.00057199 |
| cg27387193 | 6 | TNXB | 32064032 | ENSG00000179344 | 6 | HLA-DQB1 | 32627244 | rs1150757 | 6 | 32029205 | A | G | 0.0755467 | -0.434887 | 0.0642446 | 1.29E-11 | -0.438225 | 0.0467498 | 6.99E-21 | 0.992383 | 0.180831 | 4.07E-08 | 0.05843571 | 20 | 0.000238278 |
| cg17662683 | 6 | TNXB | 32064146 | ENSG00000179344 | 6 | HLA-DQB1 | 32627244 | rs6933289 | 6 | 32604551 | T | C | 0.0715706 | -0.466806 | 0.0537357 | 3.72E-18 | -0.379947 | 0.0453648 | 5.51E-17 | 1.22861 | 0.203767 | 1.65E-09 | 0.2971498 | 20 | 9.64E-06 |
| cg01569346 | 6 | TNXB | 32064148 | ENSG00000179344 | 6 | HLA-DQB1 | 32627244 | rs6910310 | 6 | 32604221 | G | A | 0.0666004 | -0.475603 | 0.0538466 | 1.02E-18 | -0.373024 | 0.0453224 | 1.87E-16 | 1.27499 | 0.211743 | 1.73E-09 | 0.3312565 | 20 | 1.01E-05 |
| cg19267551 | 6 | TNXB | 32064161 | ENSG00000179344 | 6 | HLA-DQB1 | 32627244 | rs433061 | 6 | 32014828 | A | G | 0.0735587 | -0.434887 | 0.0642446 | 1.29E-11 | -0.331752 | 0.0469019 | 1.51E-12 | 1.31088 | 0.268044 | 1.01E-06 | 0.2614866 | 20 | 0.005890805 |
| cg08516507 | 6 | TNXB | 32064206 | ENSG00000179344 | 6 | HLA-DQB1 | 32627244 | rs9272729 | 6 | 32609594 | A | G | 0.106362 | -0.438291 | 0.0498554 | 1.48E-18 | -0.33316 | 0.0465419 | 8.17E-13 | 1.31556 | 0.237 | 2.84E-08 | 0.09808466 | 20 | 0.000166507 |
| cg16834823 | 6 | TNXB | 32064218 | ENSG00000179344 | 6 | HLA-DQB1 | 32627244 | rs678770 | 6 | 32570817 | A | T | 0.222664 | -0.431837 | 0.0379437 | 5.20E-30 | -0.3428 | 0.0386047 | 6.70E-19 | 1.25973 | 0.179938 | 2.54E-12 | 0.258573 | 20 | 1.49E-08 |
| cg10923662 | 6 | TNXB | 32064258 | ENSG00000179344 | 6 | HLA-DQB1 | 32627244 | rs678770 | 6 | 32570817 | A | T | 0.222664 | -0.431837 | 0.0379437 | 5.20E-30 | -0.340809 | 0.0384524 | 7.78E-19 | 1.26709 | 0.1812 | 2.69E-12 | 0.240553 | 20 | 1.58E-08 |
| cg20414186 | 6 | TNXB | 32064491 | ENSG00000179344 | 6 | HLA-DQB1 | 32627244 | rs433061 | 6 | 32014828 | A | G | 0.0735587 | -0.434887 | 0.0642446 | 1.29E-11 | -0.326276 | 0.0469863 | 3.81E-12 | 1.33288 | 0.274979 | 1.25E-06 | 0.1957387 | 20 | 0.007335071 |
| cg03556669 | 6 | TNXB | 32064497 | ENSG00000179344 | 6 | HLA-DQB1 | 32627244 | rs433061 | 6 | 32014828 | A | G | 0.0735587 | -0.434887 | 0.0642446 | 1.29E-11 | -0.402953 | 0.0467292 | 6.51E-18 | 1.07925 | 0.202691 | 1.01E-07 | 0.1006415 | 20 | 0.000592662 |
| cg15793329 | 6 | TNXB | 32064749 | ENSG00000179344 | 6 | HLA-DQB1 | 32627244 | rs1269852 | 6 | 32080191 | C | G | 0.0755467 | -0.434887 | 0.0642446 | 1.29E-11 | -0.540476 | 0.0463861 | 2.25E-31 | 0.804637 | 0.137471 | 4.82E-09 | 0.08056485 | 20 | 2.83E-05 |
| cg18330047 | 6 | TNXB | 32064783 | ENSG00000179344 | 6 | HLA-DQB1 | 32627244 | rs6910310 | 6 | 32604221 | G | A | 0.0666004 | -0.475603 | 0.0538466 | 1.02E-18 | -0.478825 | 0.0452619 | 3.73E-26 | 0.993271 | 0.146498 | 1.20E-11 | 0.08605258 | 20 | 7.04E-08 |
| cg07237769 | 6 | TNXB | 32065023 | ENSG00000179344 | 6 | HLA-DQB1 | 32627244 | rs6910310 | 6 | 32604221 | G | A | 0.0666004 | -0.475603 | 0.0538466 | 1.02E-18 | -0.307061 | 0.0461643 | 2.90E-11 | 1.54889 | 0.291508 | 1.08E-07 | 0.2241533 | 20 | 0.000630457 |
| cg04037640 | 6 | TNXB | 32065034 | ENSG00000179344 | 6 | HLA-DQB1 | 32627244 | rs6910310 | 6 | 32604221 | G | A | 0.0666004 | -0.475603 | 0.0538466 | 1.02E-18 | -0.354905 | 0.0458555 | 9.97E-15 | 1.34009 | 0.230215 | 5.85E-09 | 0.1264296 | 20 | 3.43E-05 |
| cg26476939 | 6 | TNXB | 32065043 | ENSG00000179344 | 6 | HLA-DQB1 | 32627244 | rs6910310 | 6 | 32604221 | G | A | 0.0666004 | -0.475603 | 0.0538466 | 1.02E-18 | -0.318064 | 0.0460304 | 4.85E-12 | 1.49531 | 0.274755 | 5.26E-08 | 0.131396 | 20 | 0.000308065 |
| cg11945824 | 6 | PRRT1 | 32116963 | ENSG00000179344 | 6 | HLA-DQB1 | 32627244 | rs9271421 | 6 | 32587859 | T | C | 0.12326 | 0.561458 | 0.0486 | 7.16E-31 | 0.243836 | 0.0440668 | 3.14E-08 | 2.3026 | 0.461404 | 6.02E-07 | 0.09778177 | 12 | 0.003529141 |
| cg12602633 | 6 | PRRT1 | 32119691 | ENSG00000179344 | 6 | HLA-DQB1 | 32627244 | rs116667074 | 6 | 32285362 | T | C | 0.0785288 | -0.43495 | 0.0630726 | 5.35E-12 | -0.296176 | 0.0469967 | 2.94E-10 | 1.46855 | 0.315678 | 3.29E-06 | 0.06999369 | 20 | 0.01925111 |
| cg12883279 | 6 | PPT2 | 32120773 | ENSG00000179344 | 6 | HLA-DQB1 | 32627244 | rs113972770 | 6 | 32501169 | C | T | 0.143141 | 0.475425 | 0.0442681 | 6.63E-27 | 0.236274 | 0.0413194 | 1.08E-08 | 2.01218 | 0.398658 | 4.48E-07 | 0.1181224 | 20 | 0.002624006 |
| cg08828723 | 6 | NOTCH4 | 32165176 | ENSG00000179344 | 6 | HLA-DQB1 | 32627244 | rs116667074 | 6 | 32285362 | T | C | 0.0785288 | -0.43495 | 0.0630726 | 5.35E-12 | -0.458843 | 0.0466065 | 7.20E-23 | 0.947928 | 0.167828 | 1.62E-08 | 0.09521301 | 20 | 9.50E-05 |
| cg08801479 | 6 | NOTCH4 | 32165200 | ENSG00000179344 | 6 | HLA-DQB1 | 32627244 | rs1269852 | 6 | 32080191 | C | G | 0.0755467 | -0.434887 | 0.0642446 | 1.29E-11 | -0.418831 | 0.0467668 | 3.37E-19 | 1.03834 | 0.192278 | 6.66E-08 | 0.1166033 | 20 | 0.000389999 |
| cg25949002 | 6 | C6orf10 | 32301073 | ENSG00000179344 | 6 | HLA-DQB1 | 32627244 | rs2647062 | 6 | 32570417 | C | A | 0.147117 | -0.326737 | 0.0470939 | 3.98E-12 | -0.50541 | 0.0405172 | 1.04E-35 | 0.646479 | 0.106623 | 1.33E-09 | 0.08254925 | 20 | 7.82E-06 |
| cg02808240 | 6 | C6orf10 | 32304143 | ENSG00000179344 | 6 | HLA-DQB1 | 32627244 | rs2647062 | 6 | 32570417 | C | A | 0.147117 | -0.326737 | 0.0470939 | 3.98E-12 | -0.463792 | 0.0407989 | 6.05E-30 | 0.70449 | 0.118959 | 3.18E-09 | 0.1236845 | 20 | 1.86E-05 |
| cg19117063 | 6 | BTNL2 | 32365553 | ENSG00000179344 | 6 | HLA-DQB1 | 32627244 | rs116454630 | 6 | 32359519 | A | T | 0.16004 | 0.271874 | 0.0495841 | 4.18E-08 | -1.5911 | 0.0359783 | 0 | -0.170872 | 0.031402 | 5.29E-08 | 0.1888642 | 20 | 0.000309626 |
| cg18996355 | 6 | BTNL2 | 32366347 | ENSG00000179344 | 6 | HLA-DQB1 | 32627244 | rs115690055 | 6 | 32359521 | T | C | 0.159046 | 0.259222 | 0.048188 | 7.47E-08 | -0.734926 | 0.0466399 | 6.11E-56 | -0.352719 | 0.069284 | 3.56E-07 | 0.1456435 | 20 | 0.00208753 |
| cg03100814 | 6 | BTNL2 | 32367672 | ENSG00000179344 | 6 | HLA-DQB1 | 32627244 | rs9271718 | 6 | 32593465 | T | A | 0.365805 | -0.352827 | 0.0358279 | 7.00E-23 | 0.211332 | 0.0332881 | 2.17E-10 | -1.66954 | 0.312889 | 9.51E-08 | 0.07639062 | 20 | 0.000556995 |
| cg14241129 | 6 | BTNL2 | 32367729 | ENSG00000179344 | 6 | HLA-DQB1 | 32627244 | rs9273040 | 6 | 32611958 | G | C | 0.276342 | -0.407624 | 0.036781 | 1.53E-28 | 0.363591 | 0.033706 | 3.96E-27 | -1.12111 | 0.145034 | 1.08E-14 | 0.1269215 | 20 | 6.30E-11 |
| cg16004593 | 6 | BTNL2 | 32369487 | ENSG00000179344 | 6 | HLA-DQB1 | 32627244 | rs7760841 | 6 | 32574868 | T | C | 0.115308 | -0.333615 | 0.046118 | 4.69E-13 | -0.643691 | 0.0429776 | 1.03E-50 | 0.518284 | 0.0795654 | 7.32E-11 | 0.1413361 | 20 | 4.29E-07 |
| cg00331257 | 6 | BTNL2 | 32371538 | ENSG00000179344 | 6 | HLA-DQB1 | 32627244 | rs28724021 | 6 | 32550695 | T | C | 0.229622 | -0.373768 | 0.0343347 | 1.34E-27 | 0.188609 | 0.0343045 | 3.84E-08 | -1.98171 | 0.403799 | 9.22E-07 | 0.1817909 | 20 | 0.005399307 |
| cg13778567 | 6 | HLA-DQA1 | 32609783 | ENSG00000179344 | 6 | HLA-DQB1 | 32627244 | rs28366319 | 6 | 32561495 | A | G | 0.300199 | -0.400086 | 0.0372641 | 6.86E-27 | 1.09606 | 0.0248368 | 0 | -0.365022 | 0.0349899 | 1.77E-25 | 0.09515196 | 20 | 1.04E-21 |
| cg13249850 | 6 | HLA-DQB1 | 32634508 | ENSG00000179344 | 6 | HLA-DQB1 | 32627244 | rs9272358 | 6 | 32604538 | A | G | 0.370775 | -0.422971 | 0.0347185 | 3.83E-34 | 0.321775 | 0.0320818 | 1.13E-23 | -1.31449 | 0.169759 | 9.69E-15 | 0.3779017 | 20 | 5.67E-11 |
| cg01244342 | 6 | HLA-DQA2 | 32709470 | ENSG00000179344 | 6 | HLA-DQB1 | 32627244 | rs28707773 | 6 | 32572278 | A | C | 0.139165 | 0.309528 | 0.0545305 | 1.38E-08 | -0.466907 | 0.051379 | 1.01E-19 | -0.662933 | 0.137702 | 1.48E-06 | 0.4236787 | 20 | 0.008654885 |
| cg14740554 | 6 | HLA-DQA2 | 32713607 | ENSG00000179344 | 6 | HLA-DQB1 | 32627244 | rs9271523 | 6 | 32589806 | G | A | 0.293241 | -0.39622 | 0.0363291 | 1.07E-27 | 0.572868 | 0.0318429 | 2.31E-72 | -0.691643 | 0.0741594 | 1.09E-20 | 0.4774952 | 20 | 6.41E-17 |
| cg22812614 | 6 | HLA-DQA2 | 32713827 | ENSG00000179344 | 6 | HLA-DQB1 | 32627244 | rs9271464 | 6 | 32588544 | T | A | 0.284294 | -0.40521 | 0.0367864 | 3.23E-28 | -1.14986 | 0.0242236 | 0 | 0.352399 | 0.0328421 | 7.35E-27 | 0.4334742 | 20 | 4.31E-23 |
| cg20985082 | 6 | HLA-DQB2 | 32727265 | ENSG00000179344 | 6 | HLA-DQB1 | 32627244 | rs9271517 | 6 | 32589740 | G | A | 0.279324 | -0.402788 | 0.0366433 | 4.17E-28 | -0.248133 | 0.033876 | 2.39E-13 | 1.62327 | 0.266311 | 1.09E-09 | 0.2674777 | 20 | 6.39E-06 |
| cg10714510 | 6 | HLA-DQB2 | 32729465 | ENSG00000179344 | 6 | HLA-DQB1 | 32627244 | rs9270555 | 6 | 32560584 | C | T | 0.417495 | 0.271415 | 0.0353603 | 1.65E-14 | 0.199712 | 0.0326752 | 9.84E-10 | 1.35903 | 0.284236 | 1.74E-06 | 0.07879117 | 20 | 0.01019978 |
| cg02448295 | 6 | BRD2 | 32941126 | ENSG00000179344 | 6 | HLA-DQB1 | 32627244 | rs241426 | 6 | 32804553 | T | A | 0.414513 | 0.236507 | 0.0324362 | 3.07E-13 | 0.204971 | 0.0328973 | 4.65E-10 | 1.15386 | 0.243594 | 2.17E-06 | 0.1666305 | 3 | 0.012718661 |
| cg17313945 | 6 | HLA-DOA | 32977983 | ENSG00000179344 | 6 | HLA-DQB1 | 32627244 | rs72853943 | 6 | 32509260 | C | T | 0.303181 | 0.529443 | 0.035565 | 4.02E-50 | -0.193705 | 0.0346617 | 2.29E-08 | -2.73324 | 0.522416 | 1.68E-07 | 0.06818789 | 20 | 0.00098265 |
| cg00526843 | 6 | HLA-DPB2 | 33084420 | ENSG00000179344 | 6 | HLA-DQB1 | 32627244 | rs9271732 | 6 | 32593627 | G | A | 0.255467 | 0.486084 | 0.036617 | 3.24E-40 | 0.227665 | 0.0386777 | 3.95E-09 | 2.13508 | 0.396786 | 7.41E-08 | 0.07645028 | 20 | 0.00043405 |
| cg09510698 | 6 | HLA-DPB2 | 33092130 | ENSG00000179344 | 6 | HLA-DQB1 | 32627244 | rs1812006 | 6 | 32778656 | A | G | 0.0934394 | 0.432337 | 0.0527252 | 2.41E-16 | 0.248833 | 0.0436308 | 1.18E-08 | 1.73746 | 0.37109 | 2.84E-06 | 0.2674424 | 8 | 0.016639016 |
| cg13695585 | 6 | DDR1 | 30853014 | ENSG00000232629 | 6 | HLA-DQB2 | 32723875 | rs204887 | 6 | 32029226 | A | G | 0.306163 | -0.326299 | 0.0455215 | 7.61E-13 | -0.21274 | 0.034827 | 1.01E-09 | 1.53379 | 0.329899 | 3.33E-06 | 0.3966721 | 13 | 0.019513086 |
| cg07939626 | 6 | DDR1 | 30853073 | ENSG00000232629 | 6 | HLA-DQB2 | 32723875 | rs204887 | 6 | 32029226 | A | G | 0.306163 | -0.326299 | 0.0455215 | 7.61E-13 | -0.247713 | 0.0343489 | 5.53E-13 | 1.31725 | 0.2591 | 3.70E-07 | 0.08382356 | 17 | 0.002165832 |
| cg26321999 | 6 | DDR1 | 30854011 | ENSG00000232629 | 6 | HLA-DQB2 | 32723875 | rs204887 | 6 | 32029226 | A | G | 0.306163 | -0.326299 | 0.0455215 | 7.61E-13 | -0.268343 | 0.0345521 | 8.08E-15 | 1.21598 | 0.23085 | 1.38E-07 | 0.2502663 | 20 | 0.000810626 |
| cg07187855 | 6 | DDR1 | 30854161 | ENSG00000232629 | 6 | HLA-DQB2 | 32723875 | rs204887 | 6 | 32029226 | A | G | 0.306163 | -0.326299 | 0.0455215 | 7.61E-13 | -0.288941 | 0.0342186 | 3.07E-17 | 1.12929 | 0.206657 | 4.64E-08 | 0.1064002 | 20 | 0.000271806 |
| cg24566261 | 6 | DDR1 | 30854164 | ENSG00000232629 | 6 | HLA-DQB2 | 32723875 | rs2856448 | 6 | 32014575 | G | A | 0.550696 | 0.299383 | 0.0422819 | 1.43E-12 | 0.270629 | 0.0333129 | 4.52E-16 | 1.10625 | 0.20725 | 9.41E-08 | 0.1075275 | 20 | 0.000551281 |
| cg17091577 | 6 | DDR1 | 30854233 | ENSG00000232629 | 6 | HLA-DQB2 | 32723875 | rs2856448 | 6 | 32014575 | G | A | 0.550696 | 0.299383 | 0.0422819 | 1.43E-12 | 0.296357 | 0.0329159 | 2.19E-19 | 1.01021 | 0.181507 | 2.61E-08 | 0.2037702 | 20 | 0.00015296 |
| cg15656686 | 6 | DDR1 | 30854551 | ENSG00000232629 | 6 | HLA-DQB2 | 32723875 | rs204887 | 6 | 32029226 | A | G | 0.306163 | -0.326299 | 0.0455215 | 7.61E-13 | -0.232546 | 0.034919 | 2.75E-11 | 1.40316 | 0.287598 | 1.07E-06 | 0.294384 | 20 | 0.00624973 |
| cg12669395 | 6 | DDR1 | 30860866 | ENSG00000232629 | 6 | HLA-DQB2 | 32723875 | rs185819 | 6 | 32050067 | C | T | 0.550696 | 0.295837 | 0.0423037 | 2.69E-12 | 0.218236 | 0.0335871 | 8.16E-11 | 1.35558 | 0.284782 | 1.94E-06 | 0.1189934 | 20 | 0.011336929 |
| cg16990009 | 6 | GTF2H4 | 30880155 | ENSG00000232629 | 6 | HLA-DQB2 | 32723875 | rs9267658 | 6 | 31845985 | T | C | 0.104374 | -0.587038 | 0.0671542 | 2.30E-18 | -0.3189 | 0.0436432 | 2.73E-13 | 1.84082 | 0.328346 | 2.07E-08 | 0.1204409 | 7 | 0.000121053 |
| cg07906263 | 6 | VARS2 | 30893133 | ENSG00000232629 | 6 | HLA-DQB2 | 32723875 | rs28366261 | 6 | 32559572 | C | G | 0.166004 | 0.432913 | 0.0667254 | 8.70E-11 | -0.295389 | 0.0467545 | 2.65E-10 | -1.46557 | 0.323786 | 6.00E-06 | 0.104441 | 14 | 0.035153764 |
| cg02652369 | 6 | CCHCR1 | 31111076 | ENSG00000232629 | 6 | HLA-DQB2 | 32723875 | rs3117182 | 6 | 32066819 | T | A | 0.10835 | -0.526178 | 0.0651868 | 6.92E-16 | 0.281723 | 0.0447485 | 3.06E-10 | -1.86771 | 0.376231 | 6.90E-07 | 0.06618501 | 20 | 0.004039595 |
| cg12044213 | 6 | CCHCR1 | 31124978 | ENSG00000232629 | 6 | HLA-DQB2 | 32723875 | rs1048709 | 6 | 31914935 | A | G | 0.152087 | -0.519598 | 0.0573237 | 1.25E-19 | -0.256931 | 0.0391893 | 5.52E-11 | 2.02233 | 0.380693 | 1.08E-07 | 0.06952391 | 17 | 0.000634384 |
| cg21863888 | 6 | TCF19 | 31127173 | ENSG00000232629 | 6 | HLA-DQB2 | 32723875 | rs1130455 | 6 | 32627405 | G | C | 0.224652 | -0.630649 | 0.0555532 | 7.23E-30 | 0.270063 | 0.0467993 | 7.90E-09 | -2.33519 | 0.453948 | 2.69E-07 | 0.07649651 | 20 | 0.001573964 |
| cg09045681 | 6 | TCF19 | 31127178 | ENSG00000232629 | 6 | HLA-DQB2 | 32723875 | rs1130455 | 6 | 32627405 | G | C | 0.224652 | -0.630649 | 0.0555532 | 7.23E-30 | 0.295812 | 0.0468907 | 2.82E-10 | -2.13192 | 0.386618 | 3.50E-08 | 0.05096234 | 20 | 0.00020514 |
| cg26504835 | 6 | PSORS1C3 | 31143710 | ENSG00000232629 | 6 | HLA-DQB2 | 32723875 | rs28383182 | 6 | 32571811 | A | G | 0.265408 | 0.627121 | 0.0464855 | 1.77E-41 | 0.192518 | 0.0342847 | 1.96E-08 | 3.25747 | 0.628354 | 2.17E-07 | 0.1214124 | 20 | 0.001271359 |
| cg20073472 | 6 | PSORS1C3 | 31146845 | ENSG00000232629 | 6 | HLA-DQB2 | 32723875 | rs2072633 | 6 | 31919578 | A | G | 0.349901 | -0.28552 | 0.0445264 | 1.43E-10 | 0.213807 | 0.0321966 | 3.12E-11 | -1.33541 | 0.289499 | 3.97E-06 | 0.1893935 | 11 | 0.023269013 |
| cg24005169 | 6 | PSORS1C3 | 31146942 | ENSG00000232629 | 6 | HLA-DQB2 | 32723875 | rs3130342 | 6 | 32080146 | A | C | 0.107356 | -0.54471 | 0.0658196 | 1.28E-16 | 0.246084 | 0.0439452 | 2.15E-08 | -2.21351 | 0.477273 | 3.52E-06 | 0.1086286 | 20 | 0.020624671 |
| cg25355501 | 6 | HLA-C | 31239910 | ENSG00000232629 | 6 | HLA-DQB2 | 32723875 | rs9272348 | 6 | 32604396 | T | C | 0.204771 | 0.482281 | 0.0475727 | 3.76E-24 | -0.251744 | 0.0390645 | 1.16E-10 | -1.91576 | 0.352258 | 5.37E-08 | 0.1466297 | 20 | 0.000314729 |
| cg27529346 | 6 | HLA-B | 31322926 | ENSG00000232629 | 6 | HLA-DQB2 | 32723875 | rs9267658 | 6 | 31845985 | T | C | 0.104374 | -0.587038 | 0.0671542 | 2.30E-18 | 0.29936 | 0.0447977 | 2.35E-11 | -1.96098 | 0.369371 | 1.10E-07 | 0.1505513 | 9 | 0.000645862 |
| cg26990733 | 6 | MICA | 31371465 | ENSG00000232629 | 6 | HLA-DQB2 | 32723875 | rs9267658 | 6 | 31845985 | T | C | 0.104374 | -0.587038 | 0.0671542 | 2.30E-18 | 0.25719 | 0.0432921 | 2.84E-09 | -2.28251 | 0.464536 | 8.95E-07 | 0.3918181 | 4 | 0.005240204 |
| cg26333342 | 6 | NFKBIL1 | 31519394 | ENSG00000232629 | 6 | HLA-DQB2 | 32723875 | rs9274623 | 6 | 32635998 | T | G | 0.202783 | 0.427409 | 0.0568408 | 5.50E-14 | 0.246366 | 0.0379918 | 8.89E-11 | 1.73485 | 0.353274 | 9.07E-07 | 0.08276804 | 9 | 0.005313706 |
| cg09621572 | 6 | LTA | 31539973 | ENSG00000232629 | 6 | HLA-DQB2 | 32723875 | rs204887 | 6 | 32029226 | A | G | 0.306163 | -0.326299 | 0.0455215 | 7.61E-13 | 0.203177 | 0.0345328 | 4.01E-09 | -1.60598 | 0.353136 | 5.42E-06 | 0.1960755 | 6 | 0.031758737 |
| cg14437551 | 6 | LTA | 31539986 | ENSG00000232629 | 6 | HLA-DQB2 | 32723875 | rs204887 | 6 | 32029226 | A | G | 0.306163 | -0.326299 | 0.0455215 | 7.61E-13 | 0.204023 | 0.0345567 | 3.55E-09 | -1.59932 | 0.350945 | 5.18E-06 | 0.2887797 | 8 | 0.030369372 |
| cg03228773 | 6 | C6orf47 | 31627511 | ENSG00000232629 | 6 | HLA-DQB2 | 32723875 | rs1048709 | 6 | 31914935 | A | G | 0.152087 | -0.519598 | 0.0573237 | 1.25E-19 | 0.252324 | 0.0387313 | 7.28E-11 | -2.05925 | 0.389263 | 1.22E-07 | 0.06893073 | 20 | 0.000716058 |
| cg12608306 | 6 | BAT4 | 31632740 | ENSG00000232629 | 6 | HLA-DQB2 | 32723875 | rs9270980 | 6 | 32573909 | C | A | 0.137177 | -0.600228 | 0.060574 | 3.80E-23 | 0.273005 | 0.0415586 | 5.06E-11 | -2.1986 | 0.401552 | 4.37E-08 | 0.1282427 | 20 | 0.000255938 |
| cg07537699 | 6 | LY6G5C | 31646834 | ENSG00000232629 | 6 | HLA-DQB2 | 32723875 | rs3130342 | 6 | 32080146 | A | C | 0.107356 | -0.54471 | 0.0658196 | 1.28E-16 | 0.31118 | 0.0435242 | 8.70E-13 | -1.75047 | 0.323548 | 6.29E-08 | 0.05028228 | 20 | 0.000368728 |
| cg11261908 | 6 | DDAH2 | 31697955 | ENSG00000232629 | 6 | HLA-DQB2 | 32723875 | rs9267658 | 6 | 31845985 | T | C | 0.104374 | -0.587038 | 0.0671542 | 2.30E-18 | 0.248308 | 0.0431906 | 8.97E-09 | -2.36415 | 0.492183 | 1.56E-06 | 0.0655062 | 20 | 0.009137859 |
| cg11073255 | 6 | VARS | 31762455 | ENSG00000232629 | 6 | HLA-DQB2 | 32723875 | rs9267654 | 6 | 31841009 | A | G | 0.158052 | -0.388898 | 0.0609264 | 1.74E-10 | 0.255383 | 0.0407808 | 3.79E-10 | -1.5228 | 0.340655 | 7.81E-06 | 0.05213098 | 5 | 0.04577457 |
| cg26346875 | 6 | VARS | 31762719 | ENSG00000232629 | 6 | HLA-DQB2 | 32723875 | rs9267658 | 6 | 31845985 | T | C | 0.104374 | -0.587038 | 0.0671542 | 2.30E-18 | 0.264478 | 0.0442579 | 2.29E-09 | -2.21961 | 0.449924 | 8.09E-07 | 0.1408338 | 5 | 0.004736444 |
| cg05789250 | 6 | C6orf48 | 31804306 | ENSG00000232629 | 6 | HLA-DQB2 | 32723875 | rs494620 | 6 | 31838713 | A | G | 0.451292 | 0.261498 | 0.0459496 | 1.26E-08 | -0.276077 | 0.0335289 | 1.81E-16 | -0.947192 | 0.202322 | 2.85E-06 | 0.05083551 | 12 | 0.01667458 |
| cg16361343 | 6 | EHMT2 | 31863472 | ENSG00000232629 | 6 | HLA-DQB2 | 32723875 | rs537160 | 6 | 31916400 | A | G | 0.247515 | -0.336469 | 0.0485243 | 4.09E-12 | -0.209827 | 0.0333277 | 3.06E-10 | 1.60355 | 0.344024 | 3.14E-06 | 0.5492208 | 13 | 0.018418179 |
| cg04182226 | 6 | TNXB | 32014300 | ENSG00000232629 | 6 | HLA-DQB2 | 32723875 | rs2269426 | 6 | 32076499 | A | G | 0.412525 | 0.300814 | 0.0441746 | 9.78E-12 | 0.232295 | 0.0337239 | 5.65E-12 | 1.29497 | 0.267407 | 1.28E-06 | 0.1797686 | 3 | 0.007504438 |
| cg25375757 | 6 | TNXB | 32014739 | ENSG00000232629 | 6 | HLA-DQB2 | 32723875 | rs2269426 | 6 | 32076499 | A | G | 0.412525 | 0.300814 | 0.0441746 | 9.78E-12 | 0.250922 | 0.0340227 | 1.64E-13 | 1.19883 | 0.239616 | 5.64E-07 | 0.1464577 | 16 | 0.0033041 |
| cg04841389 | 6 | TNXB | 32015083 | ENSG00000232629 | 6 | HLA-DQB2 | 32723875 | rs2269426 | 6 | 32076499 | A | G | 0.412525 | 0.300814 | 0.0441746 | 9.78E-12 | 0.213468 | 0.033387 | 1.62E-10 | 1.40918 | 0.302323 | 3.14E-06 | 0.4199227 | 4 | 0.018418694 |
| cg02628680 | 6 | TNXB | 32015618 | ENSG00000232629 | 6 | HLA-DQB2 | 32723875 | rs2269426 | 6 | 32076499 | A | G | 0.412525 | 0.300814 | 0.0441746 | 9.78E-12 | 0.277731 | 0.0347668 | 1.37E-15 | 1.08311 | 0.209003 | 2.19E-07 | 0.3897069 | 19 | 0.001283911 |
| cg13036593 | 6 | TNXB | 32026299 | ENSG00000232629 | 6 | HLA-DQB2 | 32723875 | rs9267658 | 6 | 31845985 | T | C | 0.104374 | -0.587038 | 0.0671542 | 2.30E-18 | -0.275605 | 0.044117 | 4.18E-10 | 2.13 | 0.419072 | 3.72E-07 | 0.084919 | 13 | 0.002180392 |
| cg19071976 | 6 | TNXB | 32026656 | ENSG00000232629 | 6 | HLA-DQB2 | 32723875 | rs2269426 | 6 | 32076499 | A | G | 0.412525 | 0.300814 | 0.0441746 | 9.78E-12 | 0.283047 | 0.033734 | 4.84E-17 | 1.06277 | 0.200999 | 1.24E-07 | 0.089282 | 11 | 0.000726622 |
| cg14669361 | 6 | TNXB | 32038747 | ENSG00000232629 | 6 | HLA-DQB2 | 32723875 | rs6463 | 6 | 32006621 | C | A | 0.338966 | 0.297346 | 0.0479985 | 5.83E-10 | 0.293239 | 0.0365929 | 1.11E-15 | 1.01401 | 0.206891 | 9.53E-07 | 0.0849837 | 15 | 0.005581133 |
| cg23462632 | 6 | TNXB | 32038881 | ENSG00000232629 | 6 | HLA-DQB2 | 32723875 | rs17421624 | 6 | 32066177 | C | T | 0.33002 | 0.309719 | 0.0484172 | 1.59E-10 | 0.274565 | 0.0364645 | 5.09E-14 | 1.12804 | 0.231387 | 1.09E-06 | 0.4733457 | 18 | 0.006370862 |
| cg13798746 | 6 | TNXB | 32038887 | ENSG00000232629 | 6 | HLA-DQB2 | 32723875 | rs185819 | 6 | 32050067 | C | T | 0.550696 | 0.295837 | 0.0423037 | 2.69E-12 | 0.28874 | 0.0329508 | 1.91E-18 | 1.02458 | 0.187448 | 4.60E-08 | 0.7721341 | 20 | 0.000269756 |
| cg22851080 | 6 | TNXB | 32044404 | ENSG00000232629 | 6 | HLA-DQB2 | 32723875 | rs2269426 | 6 | 32076499 | A | G | 0.412525 | 0.300814 | 0.0441746 | 9.78E-12 | 0.210058 | 0.0342144 | 8.28E-10 | 1.43205 | 0.314058 | 5.12E-06 | 0.6712997 | 15 | 0.029988824 |
| cg01188191 | 6 | TNXB | 32047349 | ENSG00000232629 | 6 | HLA-DQB2 | 32723875 | rs2269426 | 6 | 32076499 | A | G | 0.412525 | 0.300814 | 0.0441746 | 9.78E-12 | 0.221103 | 0.0335781 | 4.56E-11 | 1.36052 | 0.287415 | 2.21E-06 | 0.3813407 | 17 | 0.012919139 |
| cg00661399 | 6 | TNXB | 32049177 | ENSG00000232629 | 6 | HLA-DQB2 | 32723875 | rs2269426 | 6 | 32076499 | A | G | 0.412525 | 0.300814 | 0.0441746 | 9.78E-12 | 0.243375 | 0.0340562 | 8.92E-13 | 1.23601 | 0.250719 | 8.23E-07 | 0.4408675 | 15 | 0.004820328 |
| cg13199127 | 6 | TNXB | 32049196 | ENSG00000232629 | 6 | HLA-DQB2 | 32723875 | rs2269426 | 6 | 32076499 | A | G | 0.412525 | 0.300814 | 0.0441746 | 9.78E-12 | 0.283116 | 0.0339205 | 7.03E-17 | 1.06251 | 0.201372 | 1.32E-07 | 0.4640217 | 20 | 0.000771964 |
| cg10303653 | 6 | TNXB | 32049516 | ENSG00000232629 | 6 | HLA-DQB2 | 32723875 | rs2269426 | 6 | 32076499 | A | G | 0.412525 | 0.300814 | 0.0441746 | 9.78E-12 | 0.244989 | 0.0335189 | 2.69E-13 | 1.22787 | 0.246444 | 6.28E-07 | 0.2312291 | 11 | 0.00368002 |
| cg05067717 | 6 | TNXB | 32051067 | ENSG00000232629 | 6 | HLA-DQB2 | 32723875 | rs2269426 | 6 | 32076499 | A | G | 0.412525 | 0.300814 | 0.0441746 | 9.78E-12 | 0.234742 | 0.0348284 | 1.58E-11 | 1.28147 | 0.267511 | 1.67E-06 | 0.6902436 | 13 | 0.009754091 |
| cg08362880 | 6 | TNXB | 32053600 | ENSG00000232629 | 6 | HLA-DQB2 | 32723875 | rs2269426 | 6 | 32076499 | A | G | 0.412525 | 0.300814 | 0.0441746 | 9.78E-12 | 0.385682 | 0.0333217 | 5.55E-31 | 0.779953 | 0.132888 | 4.38E-09 | 0.09927163 | 20 | 2.56E-05 |
| cg16662408 | 6 | TNXB | 32053637 | ENSG00000232629 | 6 | HLA-DQB2 | 32723875 | rs2269426 | 6 | 32076499 | A | G | 0.412525 | 0.300814 | 0.0441746 | 9.78E-12 | 0.269336 | 0.0337604 | 1.49E-15 | 1.11687 | 0.215637 | 2.23E-07 | 0.4746117 | 16 | 0.001303872 |
| cg06539391 | 6 | TNXB | 32053641 | ENSG00000232629 | 6 | HLA-DQB2 | 32723875 | rs2269426 | 6 | 32076499 | A | G | 0.412525 | 0.300814 | 0.0441746 | 9.78E-12 | 0.228585 | 0.034552 | 3.70E-11 | 1.31598 | 0.277336 | 2.08E-06 | 0.2185874 | 20 | 0.012208693 |
| cg04286337 | 6 | TNXB | 32063501 | ENSG00000232629 | 6 | HLA-DQB2 | 32723875 | rs2760994 | 6 | 32574308 | C | T | 0.405567 | -0.401414 | 0.045163 | 6.22E-19 | 0.229127 | 0.0329766 | 3.70E-12 | -1.75193 | 0.320044 | 4.40E-08 | 0.1435749 | 20 | 0.000257705 |
| cg14630933 | 6 | TNXB | 32076093 | ENSG00000232629 | 6 | HLA-DQB2 | 32723875 | rs2269426 | 6 | 32076499 | A | G | 0.412525 | 0.300814 | 0.0441746 | 9.78E-12 | 0.545544 | 0.0330607 | 3.59E-61 | 0.551402 | 0.0875974 | 3.08E-10 | 0.05387928 | 20 | 1.80E-06 |
| cg18419045 | 6 | TNXB | 32076147 | ENSG00000232629 | 6 | HLA-DQB2 | 32723875 | rs2269426 | 6 | 32076499 | A | G | 0.412525 | 0.300814 | 0.0441746 | 9.78E-12 | 0.228113 | 0.033872 | 1.64E-11 | 1.31871 | 0.275397 | 1.68E-06 | 0.2631727 | 9 | 0.009848973 |
| cg11606877 | 6 | TNXB | 32076592 | ENSG00000232629 | 6 | HLA-DQB2 | 32723875 | rs2269426 | 6 | 32076499 | A | G | 0.412525 | 0.300814 | 0.0441746 | 9.78E-12 | 0.391282 | 0.0336409 | 2.86E-31 | 0.768791 | 0.130823 | 4.19E-09 | 0.06390971 | 20 | 2.45E-05 |
| cg25587092 | 6 | TNXB | 32076906 | ENSG00000232629 | 6 | HLA-DQB2 | 32723875 | rs204883 | 6 | 32032743 | A | G | 0.446322 | 0.324015 | 0.0431637 | 6.07E-14 | 0.297094 | 0.034523 | 7.59E-18 | 1.09061 | 0.192793 | 1.54E-08 | 0.4406342 | 15 | 9.03E-05 |
| cg14027333 | 6 | PRRT1 | 32116317 | ENSG00000232629 | 6 | HLA-DQB2 | 32723875 | rs3130342 | 6 | 32080146 | A | C | 0.107356 | -0.54471 | 0.0658196 | 1.28E-16 | 0.266552 | 0.0443144 | 1.80E-09 | -2.04354 | 0.419997 | 1.14E-06 | 0.06501769 | 14 | 0.006684189 |
| cg25955180 | 6 | PRRT1 | 32116538 | ENSG00000232629 | 6 | HLA-DQB2 | 32723875 | rs3130342 | 6 | 32080146 | A | C | 0.107356 | -0.54471 | 0.0658196 | 1.28E-16 | 0.258082 | 0.0446075 | 7.22E-09 | -2.11061 | 0.44511 | 2.12E-06 | 0.07370488 | 15 | 0.01241111 |
| cg17272563 | 6 | PRRT1 | 32116548 | ENSG00000232629 | 6 | HLA-DQB2 | 32723875 | rs9267658 | 6 | 31845985 | T | C | 0.104374 | -0.587038 | 0.0671542 | 2.30E-18 | 0.292781 | 0.0444224 | 4.37E-11 | -2.00504 | 0.380994 | 1.42E-07 | 0.09443671 | 17 | 0.000831775 |
| cg14531663 | 6 | PRRT1 | 32116933 | ENSG00000232629 | 6 | HLA-DQB2 | 32723875 | rs3096695 | 6 | 32069806 | G | C | 0.106362 | -0.553527 | 0.0667399 | 1.10E-16 | 0.317408 | 0.0445385 | 1.03E-12 | -1.7439 | 0.322631 | 6.47E-08 | 0.0756774 | 20 | 0.000379147 |
| cg08424749 | 6 | PRRT1 | 32117292 | ENSG00000232629 | 6 | HLA-DQB2 | 32723875 | rs3130342 | 6 | 32080146 | A | C | 0.107356 | -0.54471 | 0.0658196 | 1.28E-16 | 0.247302 | 0.0451922 | 4.44E-08 | -2.20261 | 0.482544 | 5.00E-06 | 0.08219217 | 12 | 0.029318505 |
| cg12883279 | 6 | PPT2 | 32120773 | ENSG00000232629 | 6 | HLA-DQB2 | 32723875 | rs113972770 | 6 | 32501169 | C | T | 0.143141 | -0.491475 | 0.0575698 | 1.38E-17 | 0.236274 | 0.0413194 | 1.08E-08 | -2.08011 | 0.43783 | 2.02E-06 | 0.06692309 | 20 | 0.011860827 |
| cg06108383 | 6 | PPT2 | 32120899 | ENSG00000232629 | 6 | HLA-DQB2 | 32723875 | rs9267576 | 6 | 31812038 | T | G | 0.107356 | -0.548807 | 0.0669866 | 2.55E-16 | 0.272924 | 0.0453423 | 1.75E-09 | -2.01084 | 0.414542 | 1.23E-06 | 0.1924944 | 20 | 0.007204907 |
| cg02956248 | 6 | PPT2 | 32120901 | ENSG00000232629 | 6 | HLA-DQB2 | 32723875 | rs9267576 | 6 | 31812038 | T | G | 0.107356 | -0.548807 | 0.0669866 | 2.55E-16 | 0.303271 | 0.0450592 | 1.69E-11 | -1.80963 | 0.347964 | 1.99E-07 | 0.2413519 | 20 | 0.001163576 |
| cg17329164 | 6 | PPT2 | 32121259 | ENSG00000232629 | 6 | HLA-DQB2 | 32723875 | rs41268896 | 6 | 32070069 | A | G | 0.34493 | 0.292588 | 0.0481371 | 1.22E-09 | 0.250613 | 0.0365233 | 6.80E-12 | 1.16749 | 0.256599 | 5.37E-06 | 0.1419106 | 14 | 0.031447244 |
| cg14614539 | 6 | NOTCH4 | 32170458 | ENSG00000232629 | 6 | HLA-DQB2 | 32723875 | rs9272348 | 6 | 32604396 | T | C | 0.204771 | 0.482281 | 0.0475727 | 3.76E-24 | -0.33725 | 0.0392929 | 9.25E-18 | -1.43004 | 0.218308 | 5.73E-11 | 0.1274856 | 20 | 3.36E-07 |
| cg01978236 | 6 | NOTCH4 | 32191479 | ENSG00000232629 | 6 | HLA-DQB2 | 32723875 | rs115690055 | 6 | 32359521 | T | C | 0.159046 | -0.401032 | 0.0612425 | 5.82E-11 | 0.345259 | 0.0477349 | 4.73E-13 | -1.16154 | 0.239278 | 1.21E-06 | 0.1610837 | 8 | 0.00707555 |
| cg21325723 | 6 | C6orf10 | 32294577 | ENSG00000232629 | 6 | HLA-DQB2 | 32723875 | rs558721 | 6 | 32570573 | T | C | 0.157058 | 0.554042 | 0.0592362 | 8.51E-21 | -0.65457 | 0.0396078 | 2.37E-61 | -0.846421 | 0.103984 | 3.96E-16 | 0.05787669 | 20 | 2.32E-12 |
| cg21587984 | 6 | C6orf10 | 32295237 | ENSG00000232629 | 6 | HLA-DQB2 | 32723875 | rs601945 | 6 | 32573415 | G | A | 0.156064 | 0.556177 | 0.0595273 | 9.34E-21 | -0.364059 | 0.0408909 | 5.43E-19 | -1.52771 | 0.237022 | 1.15E-10 | 0.06055738 | 20 | 6.75E-07 |
| cg25949002 | 6 | C6orf10 | 32301073 | ENSG00000232629 | 6 | HLA-DQB2 | 32723875 | rs2647062 | 6 | 32570417 | C | A | 0.147117 | 0.554042 | 0.0592362 | 8.51E-21 | -0.50541 | 0.0405172 | 1.04E-35 | -1.09622 | 0.146492 | 7.26E-14 | 0.1324022 | 20 | 4.25E-10 |
| cg02808240 | 6 | C6orf10 | 32304143 | ENSG00000232629 | 6 | HLA-DQB2 | 32723875 | rs2647062 | 6 | 32570417 | C | A | 0.147117 | 0.554042 | 0.0592362 | 8.51E-21 | -0.463792 | 0.0407989 | 6.05E-30 | -1.19459 | 0.165396 | 5.10E-13 | 0.1622085 | 20 | 2.99E-09 |
| cg11075234 | 6 | C6orf10 | 32312513 | ENSG00000232629 | 6 | HLA-DQB2 | 32723875 | rs9271085 | 6 | 32576152 | T | C | 0.143141 | -0.600228 | 0.060574 | 3.80E-23 | 0.26094 | 0.0414693 | 3.13E-10 | -2.30025 | 0.43304 | 1.09E-07 | 0.1543547 | 20 | 0.000635633 |
| cg25605800 | 6 | C6orf10 | 32333231 | ENSG00000232629 | 6 | HLA-DQB2 | 32723875 | rs114020256 | 6 | 32601797 | A | G | 0.296223 | -0.674729 | 0.0451266 | 1.51E-50 | 0.296715 | 0.0409327 | 4.20E-13 | -2.274 | 0.348628 | 6.90E-11 | 0.1477161 | 20 | 4.04E-07 |
| cg03100814 | 6 | BTNL2 | 32367672 | ENSG00000232629 | 6 | HLA-DQB2 | 32723875 | rs9271718 | 6 | 32593465 | T | A | 0.365805 | 0.546517 | 0.0448574 | 3.81E-34 | 0.211332 | 0.0332881 | 2.17E-10 | 2.58606 | 0.459331 | 1.80E-08 | 0.1187465 | 20 | 0.000105531 |
| cg14241129 | 6 | BTNL2 | 32367729 | ENSG00000232629 | 6 | HLA-DQB2 | 32723875 | rs9273040 | 6 | 32611958 | G | C | 0.276342 | 0.608752 | 0.0460488 | 6.75E-40 | 0.363591 | 0.033706 | 3.96E-27 | 1.67428 | 0.200326 | 6.39E-17 | 0.06769253 | 20 | 3.74E-13 |
| cg16004593 | 6 | BTNL2 | 32369487 | ENSG00000232629 | 6 | HLA-DQB2 | 32723875 | rs7760841 | 6 | 32574868 | T | C | 0.115308 | 0.554918 | 0.0580139 | 1.12E-21 | -0.643691 | 0.0429776 | 1.03E-50 | -0.862088 | 0.106939 | 7.54E-16 | 0.05181632 | 20 | 4.42E-12 |
| cg00331257 | 6 | BTNL2 | 32371538 | ENSG00000232629 | 6 | HLA-DQB2 | 32723875 | rs28724021 | 6 | 32550695 | T | C | 0.229622 | 0.545092 | 0.0431744 | 1.53E-36 | 0.188609 | 0.0343045 | 3.84E-08 | 2.89006 | 0.57333 | 4.63E-07 | 0.1093385 | 20 | 0.002715174 |
| cg13778567 | 6 | HLA-DQA1 | 32609783 | ENSG00000232629 | 6 | HLA-DQB2 | 32723875 | rs28366319 | 6 | 32561495 | A | G | 0.300199 | 0.606733 | 0.04661 | 9.77E-39 | 1.09606 | 0.0248368 | 0 | 0.553558 | 0.0443365 | 8.97E-36 | 0.08012859 | 20 | 5.25E-32 |
| cg10846853 | 6 | HLA-DQB1 | 32632859 | ENSG00000232629 | 6 | HLA-DQB2 | 32723875 | rs34411532 | 6 | 32592294 | A | G | 0.17992 | -0.585224 | 0.0599028 | 1.52E-22 | 0.663366 | 0.0457982 | 1.52E-47 | -0.882204 | 0.108922 | 5.52E-16 | 0.3057422 | 20 | 3.23E-12 |
| cg13249850 | 6 | HLA-DQB1 | 32634508 | ENSG00000232629 | 6 | HLA-DQB2 | 32723875 | rs9272358 | 6 | 32604538 | A | G | 0.370775 | 0.631427 | 0.0432565 | 2.92E-48 | 0.321775 | 0.0320818 | 1.13E-23 | 1.96232 | 0.237382 | 1.38E-16 | 0.273278 | 20 | 8.08E-13 |
| cg01244342 | 6 | HLA-DQA2 | 32709470 | ENSG00000232629 | 6 | HLA-DQB2 | 32723875 | rs28707773 | 6 | 32572278 | A | C | 0.139165 | -0.606105 | 0.06839 | 7.83E-19 | -0.466907 | 0.051379 | 1.01E-19 | 1.29813 | 0.204598 | 2.23E-10 | 0.09648433 | 20 | 1.30E-06 |
| cg14740554 | 6 | HLA-DQA2 | 32713607 | ENSG00000232629 | 6 | HLA-DQB2 | 32723875 | rs9271523 | 6 | 32589806 | G | A | 0.293241 | 0.597071 | 0.0454518 | 2.04E-39 | 0.572868 | 0.0318429 | 2.31E-72 | 1.04225 | 0.0982407 | 2.70E-26 | 0.1855374 | 20 | 1.58E-22 |
| cg22812614 | 6 | HLA-DQA2 | 32713827 | ENSG00000232629 | 6 | HLA-DQB2 | 32723875 | rs9271464 | 6 | 32588544 | T | A | 0.284294 | 0.604543 | 0.0460758 | 2.51E-39 | -1.14986 | 0.0242236 | 0 | -0.525754 | 0.0415733 | 1.17E-36 | 0.1944156 | 20 | 6.86E-33 |
| cg04090745 | 6 | HLA-DQB2 | 32724369 | ENSG00000232629 | 6 | HLA-DQB2 | 32723875 | rs72851017 | 6 | 32501352 | C | A | 0.149105 | -0.571079 | 0.0648834 | 1.35E-18 | 0.302094 | 0.0494613 | 1.01E-09 | -1.8904 | 0.376733 | 5.22E-07 | 0.08286534 | 20 | 0.003060471 |
| cg20985082 | 6 | HLA-DQB2 | 32727265 | ENSG00000232629 | 6 | HLA-DQB2 | 32723875 | rs9271517 | 6 | 32589740 | G | A | 0.279324 | 0.605653 | 0.0458436 | 7.55E-40 | -0.248133 | 0.033876 | 2.39E-13 | -2.44084 | 0.381022 | 1.49E-10 | 0.1923704 | 20 | 8.75E-07 |
| cg25327122 | 6 | HLA-DQB2 | 32729782 | ENSG00000232629 | 6 | HLA-DQB2 | 32723875 | rs1044043 | 6 | 32793981 | A | C | 0.195825 | -0.343538 | 0.0537348 | 1.62E-10 | 0.237531 | 0.0377338 | 3.08E-10 | -1.44629 | 0.322434 | 7.27E-06 | 0.05078138 | 4 | 0.042608298 |
| cg16899306 | 6 | HLA-DQB2 | 32732585 | ENSG00000232629 | 6 | HLA-DQB2 | 32723875 | rs9274505 | 6 | 32634126 | A | G | 0.137177 | 0.437117 | 0.0561367 | 6.88E-15 | 0.227887 | 0.0382592 | 2.58E-09 | 1.91813 | 0.405443 | 2.23E-06 | 0.8297192 | 7 | 0.013090937 |
| cg07930587 | 6 | PSMB9 | 32823566 | ENSG00000232629 | 6 | HLA-DQB2 | 32723875 | rs9274662 | 6 | 32636494 | T | C | 0.356859 | -0.888792 | 0.0378498 | 6.22E-122 | 0.259843 | 0.0332204 | 5.21E-15 | -3.4205 | 0.460926 | 1.16E-13 | 0.06726191 | 20 | 6.81E-10 |
| cg02448295 | 6 | BRD2 | 32941126 | ENSG00000232629 | 6 | HLA-DQB2 | 32723875 | rs241426 | 6 | 32804553 | T | A | 0.414513 | -0.26606 | 0.0416736 | 1.72E-10 | 0.204971 | 0.0328973 | 4.65E-10 | -1.29804 | 0.291099 | 8.23E-06 | 0.1326413 | 3 | 0.048216993 |
| cg10850215 | 6 | HLA-DPB1 | 33048469 | ENSG00000232629 | 6 | HLA-DQB2 | 32723875 | rs114567049 | 6 | 32601719 | A | G | 0.244533 | -0.72468 | 0.0467234 | 2.97E-54 | 0.233332 | 0.0418031 | 2.38E-08 | -3.10579 | 0.591359 | 1.50E-07 | 0.1111915 | 10 | 0.000881613 |
| cg16055526 | 6 | HLA-DPB2 | 33083287 | ENSG00000232629 | 6 | HLA-DQB2 | 32723875 | rs28383440 | 6 | 32609046 | T | C | 0.134195 | 0.412058 | 0.068659 | 1.95E-09 | -0.315328 | 0.0446508 | 1.64E-12 | -1.30676 | 0.285743 | 4.80E-06 | 0.06383418 | 13 | 0.028138686 |
| cg07460294 | 6 | HLA-DPB2 | 33084985 | ENSG00000232629 | 6 | HLA-DQB2 | 32723875 | rs2072633 | 6 | 31919578 | A | G | 0.349901 | -0.28552 | 0.0445264 | 1.43E-10 | -0.207124 | 0.0325839 | 2.06E-10 | 1.3785 | 0.305356 | 6.35E-06 | 0.2723901 | 12 | 0.037196015 |
| cg12954267 | 6 | HLA-DPB2 | 33093109 | ENSG00000232629 | 6 | HLA-DQB2 | 32723875 | rs2856448 | 6 | 32014575 | G | A | 0.550696 | 0.299383 | 0.0422819 | 1.43E-12 | -0.205042 | 0.0335448 | 9.81E-10 | -1.46011 | 0.315568 | 3.71E-06 | 0.1532416 | 20 | 0.021742307 |
| cg13224161 | 6 | COL11A2 | 33141279 | ENSG00000232629 | 6 | HLA-DQB2 | 32723875 | rs116136354 | 6 | 33001576 | C | A | 0.0168986 | -0.884093 | 0.172754 | 3.09E-07 | -1.11686 | 0.11963 | 1.00E-20 | 0.791588 | 0.176393 | 7.20E-06 | 0.07734723 | 3 | 0.04218883 |
| cg07969781 | 6 | TAPBP | 33282628 | ENSG00000232629 | 6 | HLA-DQB2 | 32723875 | rs79936591 | 6 | 32503543 | A | G | 0.203777 | 0.560677 | 0.0552275 | 3.24E-24 | -0.241579 | 0.0409001 | 3.49E-09 | -2.32088 | 0.454598 | 3.30E-07 | 0.3234475 | 13 | 0.001934161 |
| cg23228529 | 7 | PKD1L1 | 47976869 | ENSG00000183696 | 7 | UPP1 | 48128225 | rs10278152 | 7 | 48128657 | T | A | 0.445328 | -0.0715509 | 0.013268 | 6.94E-08 | 0.369843 | 0.0319843 | 6.33E-31 | -0.193463 | 0.0395843 | 1.02E-06 | 0.1641795 | 7 | 0.005986507 |
| cg11494773 | 7 | **UPP1** | 48128242 | ENSG00000183696 | 7 | UPP1 | 48128225 | rs7459020 | 7 | 48128596 | G | A | 0.422465 | -0.0781922 | 0.0133312 | 4.48E-09 | 0.363758 | 0.031912 | 4.24E-30 | -0.214957 | 0.0412158 | 1.83E-07 | 0.3884451 | 7 | 0.001074524 |
| cg04926556 | 7 | **UPP1** | 48128294 | ENSG00000183696 | 7 | UPP1 | 48128225 | rs3763505 | 7 | 48128850 | G | A | 0.4334 | -0.0782783 | 0.0131891 | 2.94E-09 | 0.312676 | 0.0323518 | 4.25E-22 | -0.25035 | 0.0494999 | 4.25E-07 | 0.2262238 | 6 | 0.002487358 |
| cg02399030 | 7 | **UPP1** | 48128821 | ENSG00000183696 | 7 | UPP1 | 48128225 | rs3763505 | 7 | 48128850 | G | A | 0.4334 | -0.0782783 | 0.0131891 | 2.94E-09 | 0.428957 | 0.0319319 | 3.85E-41 | -0.182485 | 0.0336141 | 5.67E-08 | 0.3025584 | 6 | 0.000332296 |
| cg14983135 | 7 | **UPP1** | 48129822 | ENSG00000183696 | 7 | UPP1 | 48128225 | rs9639018 | 7 | 48117684 | T | C | 0.496024 | 0.0653622 | 0.0134409 | 1.16E-06 | -0.369237 | 0.0321931 | 1.88E-30 | -0.17702 | 0.0395387 | 7.57E-06 | 0.07870639 | 7 | 0.044318992 |
| cg08908992 | 10 | PSTK | 124740529 | ENSG00000196177 | 10 | ACADSB | 124768495 | rs10902855 | 10 | 124749040 | C | T | 0.39662 | 0.1317 | 0.0218671 | 1.71E-09 | 0.292413 | 0.0333258 | 1.72E-18 | 0.45039 | 0.0907032 | 6.85E-07 | 0.09046732 | 3 | 0.004013271 |
| cg22262889 | 10 | IKZF5 | 124768896 | ENSG00000196177 | 10 | ACADSB | 124768495 | rs10902855 | 10 | 124749040 | C | T | 0.39662 | 0.1317 | 0.0218671 | 1.71E-09 | -0.234341 | 0.0327793 | 8.74E-13 | -0.562002 | 0.122013 | 4.10E-06 | 0.4438145 | 3 | 0.024037208 |
| cg15676405 | 11 | C11orf9 | 61525677 | ENSG00000124920 | 11 | MYRF | 61520114 | rs198465 | 11 | 61521318 | A | G | 0.489066 | -0.322277 | 0.0311064 | 3.75E-25 | -0.324263 | 0.0318393 | 2.33E-24 | 0.993875 | 0.136843 | 3.79E-13 | 0.5077923 | 20 | 2.22E-09 |
| cg01825921 | 11 | C11orf9 | 61525711 | ENSG00000124920 | 11 | MYRF | 61520114 | rs198460 | 11 | 61524974 | A | G | 0.497018 | -0.319589 | 0.0309394 | 5.18E-25 | -0.180962 | 0.031494 | 9.14E-09 | 1.76606 | 0.35171 | 5.13E-07 | 0.777515 | 3 | 0.003005954 |
| cg14725641 | 11 | FADS1 | 61582763 | ENSG00000134825 | 11 | TMEM258 | 61535973 | rs174561 | 11 | 61582708 | C | T | 0.303181 | 0.126043 | 0.0220238 | 1.05E-08 | -0.45784 | 0.0334317 | 1.09E-42 | -0.275299 | 0.0521351 | 1.29E-07 | 0.07186515 | 16 | 0.000754728 |
| cg03735013 | 11 | FADS1 | 61582769 | ENSG00000134825 | 11 | TMEM258 | 61535973 | rs174561 | 11 | 61582708 | C | T | 0.303181 | 0.126043 | 0.0220238 | 1.05E-08 | -0.279615 | 0.0334227 | 5.96E-17 | -0.450773 | 0.095431 | 2.32E-06 | 0.1253291 | 16 | 0.013576536 |
| cg25326896 | 11 | FADS1 | 61582785 | ENSG00000134825 | 11 | TMEM258 | 61535973 | rs174561 | 11 | 61582708 | C | T | 0.303181 | 0.126043 | 0.0220238 | 1.05E-08 | -0.346722 | 0.0336723 | 7.27E-25 | -0.363528 | 0.0726718 | 5.66E-07 | 0.3060935 | 16 | 0.003318243 |
| cg12517394 | 11 | FADS1 | 61582795 | ENSG00000134825 | 11 | TMEM258 | 61535973 | rs174592 | 11 | 61618608 | G | A | 0.38668 | 0.109163 | 0.019967 | 4.57E-08 | -0.274167 | 0.0318942 | 8.24E-18 | -0.398162 | 0.0863096 | 3.97E-06 | 0.08390727 | 16 | 0.02322953 |
| cg10515671 | 11 | FADS1 | 61585899 | ENSG00000134825 | 11 | TMEM258 | 61535973 | rs174533 | 11 | 61549025 | A | G | 0.347913 | 0.126672 | 0.021634 | 4.76E-09 | 0.260664 | 0.0334249 | 6.26E-15 | 0.485959 | 0.103785 | 2.84E-06 | 0.7085809 | 14 | 0.016613534 |
| cg21709803 | 11 | FADS2 | 61594965 | ENSG00000134825 | 11 | TMEM258 | 61535973 | rs968567 | 11 | 61595564 | T | C | 0.150099 | 0.172576 | 0.0277231 | 4.82E-10 | -0.858068 | 0.0367893 | 2.54E-120 | -0.201122 | 0.0334397 | 1.80E-09 | 0.7668127 | 16 | 1.06E-05 |
| cg06781209 | 11 | FADS2 | 61594997 | ENSG00000134825 | 11 | TMEM258 | 61535973 | rs968567 | 11 | 61595564 | T | C | 0.150099 | 0.172576 | 0.0277231 | 4.82E-10 | -0.933168 | 0.0359179 | 8.21E-149 | -0.184936 | 0.0305495 | 1.42E-09 | 0.800833 | 16 | 8.30E-06 |
| cg07005513 | 11 | FADS2 | 61595956 | ENSG00000134825 | 11 | TMEM258 | 61535973 | rs174559 | 11 | 61581656 | A | G | 0.267396 | 0.143467 | 0.0229478 | 4.06E-10 | -0.326923 | 0.034982 | 9.15E-21 | -0.43884 | 0.0844517 | 2.03E-07 | 0.7903511 | 16 | 0.001190609 |
| cg00603274 | 11 | FADS2 | 61596626 | ENSG00000134825 | 11 | TMEM258 | 61535973 | rs61896141 | 11 | 61556039 | C | A | 0.152087 | 0.187231 | 0.0274773 | 9.49E-12 | -0.926628 | 0.0364744 | 2.23E-142 | -0.202056 | 0.0307011 | 4.66E-11 | 0.5040536 | 16 | 2.73E-07 |
| cg14911132 | 11 | FADS2 | 61596755 | ENSG00000134825 | 11 | TMEM258 | 61535973 | rs968567 | 11 | 61595564 | T | C | 0.150099 | 0.172576 | 0.0277231 | 4.82E-10 | -0.563176 | 0.0385648 | 2.67E-48 | -0.306434 | 0.0535122 | 1.03E-08 | 0.8169158 | 16 | 6.01E-05 |
| cg01400685 | 11 | FADS2 | 61598025 | ENSG00000134825 | 11 | TMEM258 | 61535973 | rs61897793 | 11 | 61599347 | A | G | 0.151093 | 0.169318 | 0.0277325 | 1.03E-09 | -0.859084 | 0.037219 | 7.04E-118 | -0.197091 | 0.0333917 | 3.58E-09 | 0.7960453 | 16 | 2.10E-05 |
| cg25324164 | 11 | FADS2 | 61598330 | ENSG00000134825 | 11 | TMEM258 | 61535973 | rs968567 | 11 | 61595564 | T | C | 0.150099 | 0.172576 | 0.0277231 | 4.82E-10 | -0.707074 | 0.0376923 | 1.63E-78 | -0.244071 | 0.0413106 | 3.46E-09 | 0.3502938 | 16 | 2.03E-05 |
| cg11250194 | 11 | FADS2 | 61601937 | ENSG00000134825 | 11 | TMEM258 | 61535973 | rs174559 | 11 | 61581656 | A | G | 0.267396 | 0.143467 | 0.0229478 | 4.06E-10 | -0.80871 | 0.0324609 | 5.34E-137 | -0.177402 | 0.0292556 | 1.33E-09 | 0.7584954 | 16 | 7.78E-06 |
| cg07591205 | 11 | FADS2 | 61633557 | ENSG00000134825 | 11 | TMEM258 | 61535973 | rs174544 | 11 | 61567753 | A | C | 0.304175 | 0.126043 | 0.0220238 | 1.05E-08 | 0.24516 | 0.0335969 | 2.94E-13 | 0.514126 | 0.114168 | 6.69E-06 | 0.2341107 | 16 | 0.039203687 |
| **cg07689907** | 11 | FADS1 | 61582574 | ENSG00000149485 | 11 | FADS1 | 61567099 | rs174560 | 11 | 61581764 | C | T | 0.313121 | 0.184021 | 0.0389415 | 2.29E-06 | -0.696743 | 0.0330452 | 1.10E-98 | -0.264116 | 0.0572774 | 4.00E-06 | 0.08804336 | 9 | 0.023455455 |
| cg21709803 | 11 | FADS2 | 61594965 | ENSG00000149485 | 11 | FADS1 | 61567099 | rs968567 | 11 | 61595564 | T | C | 0.150099 | 0.289897 | 0.0510035 | 1.32E-08 | -0.858068 | 0.0367893 | 2.54E-120 | -0.337849 | 0.0611795 | 3.35E-08 | 0.6424895 | 9 | 0.000196056 |
| cg06781209 | 11 | FADS2 | 61594997 | ENSG00000149485 | 11 | FADS1 | 61567099 | rs968567 | 11 | 61595564 | T | C | 0.150099 | 0.289897 | 0.0510035 | 1.32E-08 | -0.933168 | 0.0359179 | 8.21E-149 | -0.310659 | 0.055949 | 2.82E-08 | 0.5683891 | 9 | 0.000164934 |
| cg19610905 | 11 | FADS2 | 61596333 | ENSG00000149485 | 11 | FADS1 | 61567099 | rs174566 | 11 | 61592362 | G | A | 0.355865 | 0.18532 | 0.0385069 | 1.49E-06 | -0.851216 | 0.0301912 | 6.89E-175 | -0.217712 | 0.0458919 | 2.10E-06 | 0.1372281 | 9 | 0.012273447 |
| cg00603274 | 11 | FADS2 | 61596626 | ENSG00000149485 | 11 | FADS1 | 61567099 | rs61896141 | 11 | 61556039 | C | A | 0.152087 | 0.280701 | 0.0507471 | 3.18E-08 | -0.926628 | 0.0364744 | 2.23E-142 | -0.302927 | 0.0560485 | 6.49E-08 | 0.6124914 | 9 | 0.000380195 |
| cg14911132 | 11 | FADS2 | 61596755 | ENSG00000149485 | 11 | FADS1 | 61567099 | rs968567 | 11 | 61595564 | T | C | 0.150099 | 0.289897 | 0.0510035 | 1.32E-08 | -0.563176 | 0.0385648 | 2.67E-48 | -0.514754 | 0.097182 | 1.18E-07 | 0.2237947 | 9 | 0.000690359 |
| cg01400685 | 11 | FADS2 | 61598025 | ENSG00000149485 | 11 | FADS1 | 61567099 | rs61897793 | 11 | 61599347 | A | G | 0.151093 | 0.280884 | 0.051032 | 3.71E-08 | -0.859084 | 0.037219 | 7.04E-118 | -0.326958 | 0.0610684 | 8.61E-08 | 0.7473919 | 9 | 0.000504092 |
| cg25324164 | 11 | FADS2 | 61598330 | ENSG00000149485 | 11 | FADS1 | 61567099 | rs968567 | 11 | 61595564 | T | C | 0.150099 | 0.289897 | 0.0510035 | 1.32E-08 | -0.707074 | 0.0376923 | 1.63E-78 | -0.409995 | 0.0753716 | 5.34E-08 | 0.8786563 | 9 | 0.000312727 |
| cg11250194 | 11 | FADS2 | 61601937 | ENSG00000149485 | 11 | FADS1 | 61567099 | rs174559 | 11 | 61581656 | A | G | 0.267396 | 0.194577 | 0.0424213 | 4.50E-06 | -0.80871 | 0.0324609 | 5.34E-137 | -0.240602 | 0.0533371 | 6.45E-06 | 0.3719103 | 9 | 0.037804152 |
| eQTL: expression quantitative trait loci.  mQTL: methylation quantitative trait loci.  SMR: summary-data-based Mendelian randomization.  HEIDI: heterogeneity in dependent instruments.  Only genome-wide significant mQTLs (P<5E-8) are taken into the analysis. We report SNP-gene combinations with PSMR < genome-wide significance Benjamin Hochberg correction threshold of PFDR＜0.05, and survived after the heterogeneity test (PHEIDI＞0.05). β in eQTL association, regression coefficient of gene expression on SNP, log(OR). SE, standard error. β in mQTL association, regression coefficient of DNA methylation on SNP. β in SMR association, regression coefficient of DNA methylation om gene expression. | | | | | | | | | | | | | | | | | | | | | | | | | |

| **Table S12. SMR results from tissue eQTL of diverse metabolites causally associated with cancer outcomes** | | | | | | | | | | | | | | | | | | | | | | | | | | | | |  |
| --- | --- | --- | --- | --- | --- | --- | --- | --- | --- | --- | --- | --- | --- | --- | --- | --- | --- | --- | --- | --- | --- | --- | --- | --- | --- | --- | --- | --- | --- |
| **Type of disease** | **Type of metabolites** | **Probe** | **Gene Chr.** | **Gene** | **Probe base pair** | | **topSNP** | | **Gene Chr.** | | **Probe base pair** | | **Effect allele** | **Other allele** | **Effect allele frequence** | **GWAS association** | | | **eQTL association** | | | **SMR association** | | | **HEIDI Test** | | **BH correction** | **colocalization** |  |
|  |  |  |  |  |  |  |  |  |  |  |  |  |  |  |  | **β** | **SE** | ***P*** | **β** | **SE** | ***P*** | **β** | **SE** | ***P*** | ***P*** | **nsnp** | ***P-FDR*** | **PP.H4** |  |
| colon cancer | 1,2-dipalmitoyl-gpc levels | ENSG00000204301 | 6 | NOTCH4 | 32162620 | | rs3134932 | | 6 | | 32186147 | | A | C | 0.377734 | 0.0960773 | 0.0155568 | 6.58E-10 | -0.22644 | 0.0325048 | 3.25E-12 | -0.424295 | 0.0918122 | 3.81E-06 | 0.15465182 | 20 | 3.34E-03 | 0.78329552 |  |
|  |  | ENSG00000196301 | 6 | HLA-DRB9 | 32427598 | | rs72853943 | | 6 | | 32509260 | | T | C | 0.696819 | 0.0999264 | 0.0171905 | 6.14E-09 | 0.56369 | 0.0659123 | 1.21E-17 | 0.177272 | 0.0368741 | 1.53E-06 | 0.6189653 | 20 | 1.99E-03 | 0.004836283 |  |
|  |  | ENSG00000196126 | 6 | HLA-DRB1 | 32546546 | | rs9270560 | | 6 | | 32560741 | | C | T | 0.576541 | 0.0843236 | 0.0152993 | 3.56E-08 | -0.345178 | 0.0474525 | 3.49E-13 | -0.24429 | 0.0556089 | 1.12E-05 | 0.2905788 | 20 | 7.28E-03 | 0.034543675 |  |
|  |  | ENSG00000196735 | 6 | HLA-DQA1 | 32595956 | | rs12527758 | | 6 | | 32620286 | | C | T | 0.441352 | 0.0765301 | 0.015329 | 5.96E-07 | -0.434179 | 0.0363677 | 7.45E-33 | -0.176264 | 0.0382685 | 4.11E-06 | 0.2471987 | 20 | 3.34E-03 | 0.07074893 |  |
|  |  | ENSG00000232629 | 6 | HLA-DQB2 | 32723875 | | rs9274562 | | 6 | | 32635000 | | G | A | 0.540755 | 0.082946 | 0.0155642 | 9.86E-08 | 0.96071 | 0.0505158 | 1.21E-80 | 0.0863382 | 0.0168248 | 2.87E-07 | 0.9865369 | 20 | 4.68E-04 | 0.025679284 |  |
|  |  | ENSG00000149485 | 11 | FADS1 | 61567099 | | rs1535 | | 11 | | 61597972 | | G | A | 0.349901 | -0.211095 | 0.0159617 | 6.29E-40 | -0.305737 | 0.0460364 | 3.11E-11 | 0.690446 | 0.116336 | 2.94E-09 | 0.1629577 | 14 | 9.57E-06 | 0.83561235 |  |
|  |  | ENSG00000230513 | 22 | THAP7-AS1 | 21356175 | | rs28372939 | | 22 | | 21356824 | | A | G | 0.21173 | -0.0750194 | 0.0190859 | 8.47E-05 | -0.898663 | 0.058841 | 1.16E-52 | 0.0834789 | 0.0219302 | 1.41E-04 | 0.4809475 | 19 | 6.55E-02 | 0.007937463 |  |
|  | 1,2-dilinoleoyl-GPC levels | ENSG00000149485 | 11 | FADS1 | 61567099 | | rs1535 | | 11 | | 61597972 | | G | A | 0.349901 | 0.290609 | 0.0155819 | 1.25E-77 | -0.305737 | 0.0460364 | 3.11E-11 | -0.95052 | 0.151928 | 3.94E-10 | 0.147403 | 14 | 1.28E-06 | 0.8836834 |  |
|  | 1-oleoyl-2-linoleoyl-GPE levels | ENSG00000149485 | 11 | FADS1 | 61567099 | | rs1535 | | 11 | | 61597972 | | G | A | 0.349901 | 0.388204 | 0.0156976 | 5.07E-135 | -0.305737 | 0.0460364 | 3.11E-11 | -1.26973 | 0.197964 | 1.42E-10 | 0.12857508 | 14 | 4.62E-07 | 0.75856723 |  |
|  |  | ENSG00000183793 | 16 | NPIPA5 | 15457516 | | rs3198697 | | 16 | | 15129940 | | T | C | 0.397614 | -0.0824601 | 0.0156211 | 1.30E-07 | 0.812135 | 0.0717879 | 1.13E-29 | -0.101535 | 0.0212255 | 1.72E-06 | 0.06538708 | 20 | 3.74E-03 | 0.79473812 |  |
|  | 1-palmitoyl-2-arachidonoyl-gpc levels | ENSG00000124920 | 11 | MYRF | 61520114 | | rs2238001 | | 11 | | 61524507 | | C | T | 0.121272 | 0.149477 | 0.0229213 | 6.97E-11 | -0.24528 | 0.0438004 | 2.14E-08 | -0.609414 | 0.143442 | 2.15E-05 | 0.494347287 | 15 | 3.50E-02 | 0.504837234 |  |
|  |  | ENSG00000149485 | 11 | FADS1 | 61567099 | | rs1535 | | 11 | | 61597972 | | G | A | 0.349901 | -0.605509 | 0.0148936 | 1.00E-200 | -0.305737 | 0.0460364 | 3.11E-11 | 1.98049 | 0.302165 | 5.59E-11 | 0.1299926 | 14 | 1.82E-07 | 0.8873834 |  |
|  |  | ENSG00000183793 | 16 | NPIPA5 | 15457516 | | rs3198697 | | 16 | | 15129940 | | T | C | 0.397614 | 0.0989714 | 0.0155622 | 2.02E-10 | 0.812135 | 0.0717879 | 1.13E-29 | 0.121866 | 0.0219824 | 2.96E-08 | 0.091813952 | 20 | 6.42E-05 | 0.773364123 |  |
|  | Arachidonate to oleate to vaccenate ratio | ENSG00000149485 | 11 | FADS1 | 61567099 | | rs1535 | | 11 | | 61597972 | | G | A | 0.349901 | -0.432234 | 0.0155168 | 9.15E-171 | -0.305737 | 0.0460364 | 3.11E-11 | 1.41374 | 0.218841 | 1.05E-10 | 0.1836275 | 14 | 3.41E-07 | 0.79373632 |  |
|  |  | ENSG00000183793 | 16 | NPIPA5 | 15457516 | | rs3198697 | | 16 | | 15129940 | | T | C | 0.397614 | 0.07034 | 0.0156308 | 6.79E-06 | 0.812135 | 0.0717879 | 1.13E-29 | 0.0866112 | 0.0207133 | 2.90E-05 | 0.233421869 | 20 | 6.29E-02 | 0.803847314 |  |
|  | Phosphate to linoleoyl-arachidonoyl-glycerol ratio | ENSG00000149485 | 11 | FADS1 | 61567099 | | rs1535 | | 11 | | 61597972 | | G | A | 0.349901 | 0.285424 | 0.0167162 | 2.29E-65 | -0.305737 | 0.0460364 | 3.11E-11 | -0.933561 | 0.15083 | 6.04E-10 | 0.1386338 | 14 | 1.96E-06 | 0.883927432 |  |
|  | Arachidonate to linoleate ratio | ENSG00000149485 | 11 | FADS1 | | 61567099 | | rs1535 | | 11 | | 61597972 | G | A | 0.349901 | -0.406447 | 0.0154624 | 2.76E-152 | -0.305737 | 0.0460364 | 3.11E-11 | 1.3294 | 0.206465 | 1.20E-10 | 0.3205256 | 14 | 3.92E-07 | 0.902837343 |  |
|  |  | ENSG00000183793 | 16 | NPIPA5 | | 15457516 | | rs3198697 | | 16 | | 15129940 | T | C | 0.397614 | 0.077166 | 0.0154964 | 6.37E-07 | 0.812135 | 0.0717879 | 1.13E-29 | 0.0950162 | 0.0208477 | 5.17E-06 | 0.3510616 | 20 | 1.12E-02 | 0.75937235 |  |
| Esophageal cancer | Docosapentaenoate n3 DPA; 22:5n3 levels | ENSG00000134824 | 11 | FADS2 | | 61560452 | | rs968567 | | 11 | | 61595564 | T | C | 0.150099 | -0.116291 | 0.0192583 | 1.56E-09 | 0.583728 | 0.0609734 | 1.03E-21 | -0.199221 | 0.0390065 | 3.27E-07 | 0.061473633 | 16 | 1.20E-03 | 0.017638272 |  |
|  | Docosapentaenoate n3 levels | ENSG00000149485 | 11 | FADS1 | | 61567099 | | rs28456 | | 11 | | 61589481 | G | A | 0.310139 | -0.140882 | 0.015883 | 7.32E-19 | -0.457743 | 0.0365771 | 6.22E-36 | 0.307775 | 0.0425303 | 4.60E-13 | 0.2273371 | 20 | 3.38E-09 | 0.89338423 |  |
|  |  | ENSG00000167165 | 2 | UGT1A6 | | 234600253 | | rs1105879 | | 2 | | 234602202 | C | A | 0.333002 | -0.0813096 | 0.0166826 | 1.09E-06 | -0.315419 | 0.0371776 | 2.17E-17 | 0.257783 | 0.0609966 | 2.38E-05 | 0.6640683 | 20 | 3.47E-03 | 0.867383742 | |
|  | X-23641 levels | ENSG00000197747 | 1 | S100A10 | | 151955391 | | rs6677973 | | 1 | | 151995248 | T | C | 0.292247 | 0.145303 | 0.017821 | 3.54E-16 | 0.126785 | 0.0232568 | 4.99E-08 | 1.14606 | 0.252889 | 5.85E-06 | 0.14399491 | 3 | 8.60E-03 | 0.85373723 | |
|  |  | ENSG00000009950 | 7 | MLXIPL | | 73007524 | | rs17145750 | | 7 | | 73026378 | T | C | 0.147117 | -0.121461 | 0.0228111 | 1.01E-07 | 0.405917 | 0.0450016 | 1.88E-19 | -0.299226 | 0.0652573 | 4.53E-06 | 0.1943179 | 18 | 8.33E-03 | 0.002837432 | |
|  |  | ENSG00000159445 | 1 | THEM4 | | 151846060 | | rs12067594 | | 1 | | 151865780 | A | G | 0.604374 | -0.151737 | 0.0165012 | 3.73E-20 | 0.310626 | 0.0321529 | 4.42E-22 | -0.488488 | 0.0733392 | 2.73E-11 | 0.12120209 | 20 | 1.00E-07 | 0.031782837 | |
| Pancreatic cancer | 1-stearoyl-2-arachidonoyl-GPI levels | ENSG00000009950 | 7 | MLXIPL | | 73007524 | | rs34121855 | | 7 | | 73040814 | G | T | 0.191849 | -0.100352 | 0.0192436 | 1.84E-07 | 0.450753 | 0.0500627 | 2.18E-19 | -0.222632 | 0.0493358 | 6.40E-06 | 0.4560876 | 20 | 6.07E-03 | 0.017783636 | |
|  |  | ENSG00000124915 | 11 | RP11-467L20.10 | | 61513965 | | rs198462 | | 11 | | 61524119 | A | G | 0.494036 | 0.0757993 | 0.0151769 | 5.90E-07 | 0.5518 | 0.0746806 | 1.48E-13 | 0.137367 | 0.0331983 | 3.51E-05 | 0.08196502 | 20 | 2.77E-02 | 0.436257123 | |
|  |  | ENSG00000149485 | 11 | FADS1 | | 61567099 | | rs174561 | | 11 | | 61582708 | C | T | 0.303181 | -0.259442 | 0.0163149 | 6.13E-57 | -0.807835 | 0.056204 | 7.62E-47 | 0.321157 | 0.0301186 | 1.51E-26 | 0.06567263 | 20 | 7.18E-23 | 0.86363841 | |
|  |  | ENSG00000170892 | 19 | TSEN34 | | 54693789 | | rs39714 | | 19 | | 54693682 | G | C | 0.181909 | 0.102111 | 0.0195424 | 1.74E-07 | 0.255792 | 0.0437905 | 5.18E-09 | 0.399195 | 0.102505 | 9.84E-05 | 0.9365639 | 5 | 6.66E-02 | 0.27385632 | |
| GWAS: genome-wide association study.  eQTL: expression quantitative trait loci.  SMR: summary-data-based Mendelian randomization.  HEIDI: heterogeneity in dependent instruments. Only genome-wide significant eQTLs (P<5E-8) are taken into the analysis. We report SNP-gene combinations with P_SMR_ < genome-wide significance Benjamin Hochberg correction threshold of PFDR＜0.05, and survived after the heterogeneity test (PHEIDI＞0.05). β in GWAS association, regression coefficient of cancer on SNP, log(OR).  SE, standard error.  β in eQTL association, regression coefficient of gene expression on SNP.  β in SMR association, regression coefficient of cancer on gene expression.  PP.H4, posterior probability of H4 ,PP.H4＞0.75 as the cut-off for the evidence of colocalization of cancer GWAS and eQTL association. | | | | | | | | | | | | | | | | | | | | | | | | | | | | |  |

| **Table S13. SMR results from tissue eQTL of diverse metabolites causally associated with intestinal disease outcomes** | | | | | | | | | | | | | | | | | | | | | | | | |
| --- | --- | --- | --- | --- | --- | --- | --- | --- | --- | --- | --- | --- | --- | --- | --- | --- | --- | --- | --- | --- | --- | --- | --- | --- |
| **Type of disease** | **Type of metabolites** | **Probe** | **Gene Chr.** | **Gene** | **Probe base pair** | **topSNP** | **Gene Chr.** | **Probe base pair** | **Effect allele** | **Other allele** | **Effect allele frequence** | **GWAS association** | | | **eQTL association** | | | **SMR association** | | | **HEIDI Test** | | **BH correction** | **colocalization** |
|  |  |  |  |  |  |  |  |  |  |  |  | **β** | **SE** | ***P*** | **β** | **SE** | ***P*** | **β** | **SE** | ***P*** | ***P*** | **SE** | ***P-FDR*** | **PP.H4** |
| Inflammatory bowel disease | Mannose to glycerol ratio | ENSG00000234072 | 2 | AC074117.10 | 27579113 | rs780108 | 2 | 27684957 | C | T | 0.416501 | 0.0949106 | 0.0136144 | 3.14E-12 | -0.180666 | 0.0331291 | 4.94E-08 | -0.525337 | 0.122305 | 1.74E-05 | 0.9553833 | 5 | 5.68E-02 | 0.43547123 |
|  |  | ENSG00000115216 | 2 | NRBP1 | 27650657 | rs2303370 | 2 | 27651375 | T | C | 0.411531 | 0.0916532 | 0.0136654 | 1.99E-11 | -0.203606 | 0.0244366 | 7.95E-17 | -0.45015 | 0.08616 | 1.75E-07 | 0.055437624 | 20 | 1.14E-03 | 0.8384623 |
| Ulcerative colitis | N-acetyl-isoputreanine levels | ENSG00000143149 | 1 | ALDH9A1 | 165631453 | rs12408101 | 1 | 165667781 | G | T | 0.258449 | -0.100074 | 0.0170562 | 4.43E-09 | -0.516029 | 0.0395453 | 6.43E-39 | 0.193931 | 0.0362403 | 8.73E-08 | 0.09345394 | 20 | 1.90E-04 | 0.779347294 |
|  |  | ENSG00000203772 | 10 | SPRN | 135234170 | rs11816147 | 10 | 135228453 | C | T | 0.323062 | 0.124237 | 0.0161812 | 1.62E-14 | 0.158817 | 0.0289526 | 4.13E-08 | 0.782265 | 0.175265 | 8.07E-06 | 0.1700002 | 3 | 8.76E-03 | 0.527263 |
|  |  | ENSG00000132517 | 17 | SLC52A1 | 4935895 | rs10445262 | 17 | 4943176 | G | A | 0.27833 | -0.153063 | 0.0175429 | 2.66E-18 | -0.252315 | 0.0403706 | 4.11E-10 | 0.606635 | 0.119395 | 3.76E-07 | 0.4335901 | 5 | 6.11E-04 | 0.7759824 |
|  |  | ENSG00000142494 | 17 | SLC47A1 | 19398698 | rs2453580 | 17 | 19438321 | C | T | 0.406561 | 0.0716741 | 0.015461 | 3.56E-06 | -0.523423 | 0.065112 | 9.07E-16 | -0.136933 | 0.0340979 | 5.92E-05 | 0.7437567 | 3 | 4.28E-02 | 0.81837153 |
| irritable bowel syndrome | 1-stearoyl-2-linoleoyl-GPI levels | ENSG00000149485 | 11 | FADS1 | 61567099 | rs1535 | 11 | 61597972 | G | A | 0.349901 | 0.207039 | 0.0154817 | 8.68E-41 | -0.305737 | 0.0460364 | 3.11E-11 | -0.67718 | 0.113848 | 2.71E-09 | 0.08019195 | 14 | 8.83E-06 | 0.2836491 |
|  | Urea levels | ENSG00000114054 | 3 | PCCB | 135969148 | rs556788 | 3 | 136027549 | G | A | 0.449304 | 0.0658794 | 0.0151147 | 1.31E-05 | 0.108556 | 0.0181201 | 2.09E-09 | 0.60687 | 0.172185 | 4.24E-04 | 0.06895612 | 14 | 9.47E-01 | 0.0273614 |
|  |  | ENSG00000136827 | 9 | TOR1A | 132575223 | rs2296793 | 9 | 132585058 | A | G | 0.275348 | 0.0656954 | 0.017303 | 0.000146596 | -0.271676 | 0.0376788 | 5.58E-13 | -0.241815 | 0.0719802 | 7.81E-04 | 0.07270673 | 12 | 9.47E-01 | 0.83672523 |
| GWAS: genome-wide association study.  eQTL: expression quantitative trait loci.  SMR: summary-data-based Mendelian randomization.  HEIDI: heterogeneity in dependent instruments. Only genome-wide significant eQTLs (P<5E-8) are taken into the analysis. We report SNP-gene combinations with P_SMR_ < genome-wide significance Benjamin Hochberg correction threshold of PFDR＜0.05, and survived after the heterogeneity test (PHEIDI＞0.05). β in GWAS association, regression coefficient of cancer on SNP, log(OR).  SE, standard error.  β in eQTL association, regression coefficient of gene expression on SNP.  β in SMR association, regression coefficient of cancer on gene expression.  PP.H4, posterior probability of H4 ,PP.H4＞0.75 as the cut-off for the evidence of colocalization of cancer GWAS and eQTL association. | | | | | | | | | | | | | | | | | | | | | | | | |

| **Table S14. SMR results from tissue eQTL of diverse metabolites causally associated with other digestive disorders outcomes** | | | | | | | | | | | | | | | | | | | | | | | | |  |
| --- | --- | --- | --- | --- | --- | --- | --- | --- | --- | --- | --- | --- | --- | --- | --- | --- | --- | --- | --- | --- | --- | --- | --- | --- | --- |
| **Type of disease** | **Type of metabolites** | **Probe** | **Gene Chr.** | **Gene** | **Probe base pair** | **topSNP** | **Gene Chr.** | **Probe base pair** | **Effect allele** | **Other allele** | **Effect allele frequence** | **GWAS association** | | | **eQTL association** | | | **SMR association** | | | **HEIDI Test** | | **BH correction** | **colocalization** | |
|  |  |  |  |  |  |  |  |  |  |  |  | **β** | **SE** | ***P*** | **β** | **SE** | ***P*** | **β** | **SE** | ***P*** | ***P*** | **SE** | ***P-FDR*** | **PP.H4** | |
| NAFLD | Imidazole lactate levels | ENSG00000085982 | 2 | USP40 | 234384166 | rs62192778 | 2 | 234475415 | A | G | 0.149105 | -0.160271 | 0.0198567 | 6.95E-16 | 0.518554 | 0.0849988 | 1.06E-09 | -0.309073 | 0.0635053 | 1.13E-06 | 0.6095356 | 3 | 9.31E-04 | 0.79327364 | |
|  | Bilirubin degradation product levels | ENSG00000085982 | 2 | USP40 | 234384166 | rs62192778 | 2 | 234475415 | A | G | 0.149105 | -0.143689 | 0.0204001 | 1.87E-12 | 0.518554 | 0.0849988 | 1.06E-09 | -0.277096 | 0.0600887 | 4.00E-06 | 0.5060131 | 3 | 3.28E-03 | 0.87542176 | |
|  |  | ENSG00000243135 | 2 | UGT1A3 | 234637754 | rs869283 | 2 | 234626287 | A | G | 0.347913 | 0.458147 | 0.014643 | 1.00E-200 | 0.455153 | 0.0563615 | 6.71E-16 | 1.00658 | 0.128729 | 5.31E-15 | 0.095277535 | 19 | 6.54E-12 | 0.81834677 | |
|  | Bilirubin levels | ENSG00000085982 | 2 | USP40 | 234384166 | rs62192778 | 2 | 234475415 | A | G | 0.149105 | -0.178944 | 0.0204936 | 2.51E-18 | 0.518554 | 0.0849988 | 1.06E-09 | -0.345083 | 0.0690029 | 5.70E-07 | 0.3525416 | 3 | 4.68E-04 | 0.79378461 | |
|  | Biliverdin levels | ENSG00000085982 | 2 | USP40 | 234384166 | rs62192778 | 2 | 234475415 | A | G | 0.149105 | -0.160271 | 0.0198567 | 6.95E-16 | 0.518554 | 0.0849988 | 1.06E-09 | -0.309073 | 0.0635053 | 1.13E-06 | 0.6095356 | 3 | 9.31E-04 | 0.836365384 | |
|  |  | ENSG00000233445 | 2 | RPL17P11 | 234630168 | rs11891311 | 2 | 234639310 | A | G | 0.32505 | 0.401377 | 0.0146665 | 6.80E-165 | 0.639426 | 0.0773324 | 1.36E-16 | 0.627715 | 0.0793054 | 2.47E-15 | 0.060794127 | 20 | 6.08E-12 | 0.28326634 | |
|  |  | ENSG00000243135 | 2 | UGT1A3 | 234637754 | rs869283 | 2 | 234626287 | A | G | 0.347913 | 0.436532 | 0.0143351 | 1.00E-200 | 0.455153 | 0.0563615 | 6.71E-16 | 0.959089 | 0.122869 | 5.91E-15 | 0.024236803 | 19 | 7.28E-12 | 0.363747982 | |
| Acute gastritis | Nervonoylcarnitine levels | ENSG00000162384 | 1 | C1orf123 | 53679771 | rs6663121 | 1 | 53687589 | C | T | 0.452286 | -0.107236 | 0.0158433 | 1.30E-11 | -0.171683 | 0.0309116 | 2.79E-08 | 0.624616 | 0.145478 | 1.76E-05 | 0.13261525 | 8 | 2.33E-02 | 0.383883619 | |
|  |  | ENSG00000197375 | 5 | SLC22A5 | 131705444 | rs2522051 | 5 | 131797578 | C | T | 0.483101 | 0.0883891 | 0.0156797 | 1.73E-08 | 0.197409 | 0.0313104 | 2.88E-10 | 0.447746 | 0.106545 | 2.64E-05 | 0.14081329 | 20 | 2.63E-02 | 0.48237163 | |
| Acute pancreatitis | (R)-3-hydroxybutyrylcarnitine levels | ENSG00000131435 | 5 | PDLIM4 | 131593364 | rs4705852 | 5 | 131602807 | G | A | 0.605368 | -0.0801621 | 0.0165172 | 1.21E-06 | -0.400371 | 0.0498125 | 9.16E-16 | 0.20022 | 0.0481922 | 1.26E-05 | 0.1407129 | 11 | 3.48E-02 | 0.2836114 | |
| Chronic pancreatitis | 1-palmitoyl-2-dihomo-linolenoyl-GPC levels | ENSG00000134780 | 11 | DAGLA | 61447905 | rs10792317 | 11 | 61433285 | T | C | 0.54672 | 0.0746588 | 0.0153106 | 1.08E-06 | 0.347018 | 0.0494814 | 2.33E-12 | 0.215144 | 0.0537375 | 6.24E-05 | 0.2112328 | 20 | 5.91E-03 | 0.82726654 | |
|  |  | ENSG00000124915 | 11 | RP11-467L20.10 | 61513965 | rs198462 | 11 | 61524119 | A | G | 0.494036 | -0.114756 | 0.0150366 | 2.32E-14 | 0.5518 | 0.0746806 | 1.48E-13 | -0.207967 | 0.0391762 | 1.11E-07 | 0.1040271 | 20 | 1.31E-04 | 0.3371518 | |
|  |  | ENSG00000134824 | 11 | FADS2 | 61560452 | rs968567 | 11 | 61595564 | T | C | 0.150099 | 0.384062 | 0.0192691 | 2.17E-88 | 0.495398 | 0.0699576 | 1.43E-12 | 0.775259 | 0.116183 | 2.51E-11 | 0.63366632 | 8 | 5.95E-08 | 0.056387651 | |
| GWAS: genome-wide association study.  eQTL: expression quantitative trait loci.  SMR: summary-data-based Mendelian randomization.  HEIDI: heterogeneity in dependent instruments. Only genome-wide significant eQTLs (P<5E-8) are taken into the analysis. We report SNP-gene combinations with P_SMR_ < genome-wide significance Benjamin Hochberg correction threshold of PFDR＜0.05, and survived after the heterogeneity test (PHEIDI＞0.05). β in GWAS association, regression coefficient of cancer on SNP, log(OR).  SE, standard error.  β in eQTL association, regression coefficient of gene expression on SNP.  β in SMR association, regression coefficient of cancer on gene expression.  PP.H4, posterior probability of H4 ,PP.H4＞0.75 as the cut-off for the evidence of colocalization of cancer GWAS and eQTL association. | | | | | | | | | | | | | | | | | | | | | | | | |  |
