# Supplementary material for: Targeting FADS1-mediated lipid metabolism and signaling: a novel therapeutic strategy for precision oncology in colorectal and esophageal cancers
Source: Cell Death Discov. 2025 Oct 16;11:460. doi: 10.1038/s41420-025-02768-3 (PMC12533223; doi:10.1038/s41420-025-02768-3)

# Full and uncropped western blots

Figure 6B-1.jpg


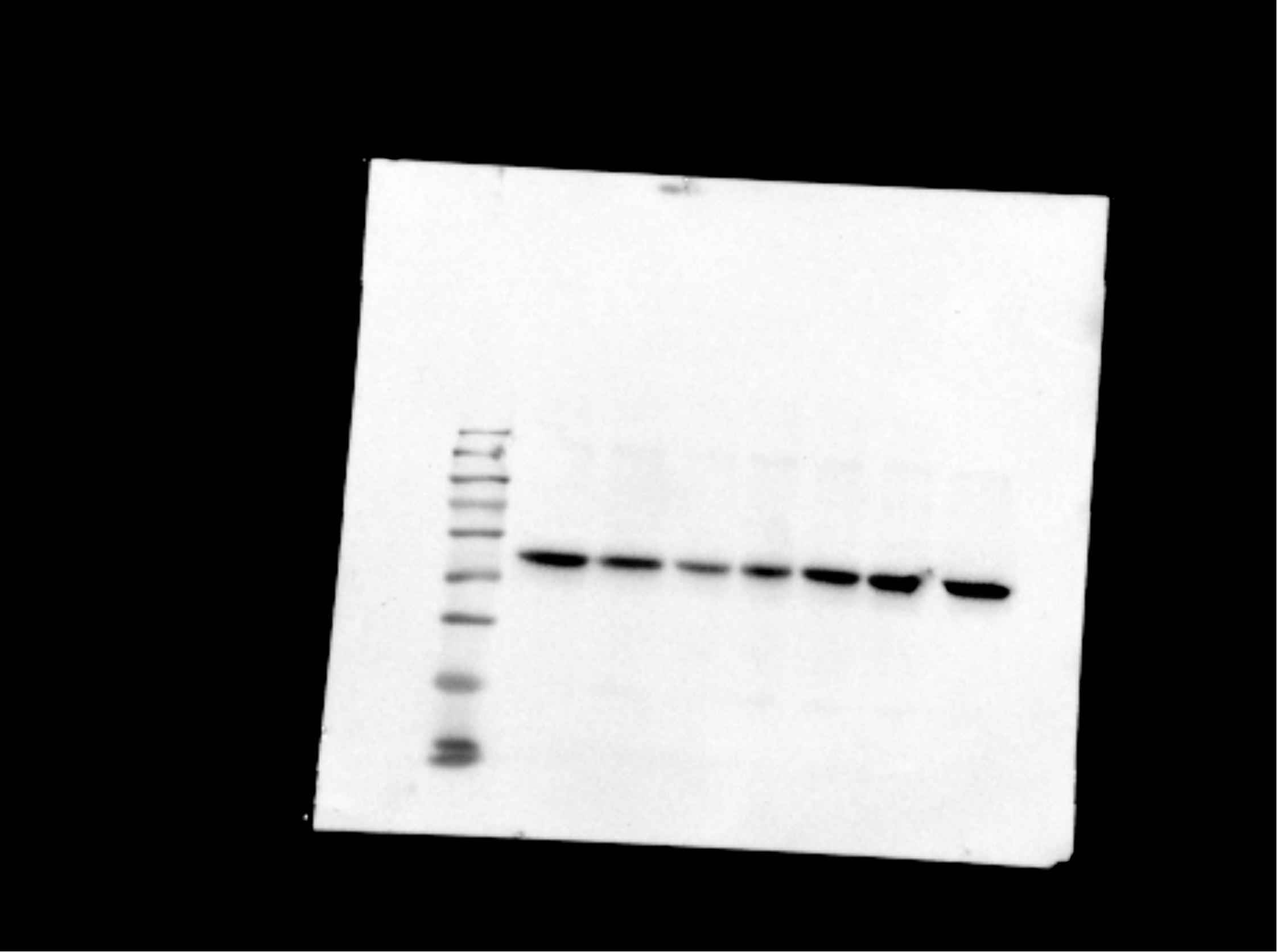


Figure 6B-2.jpg


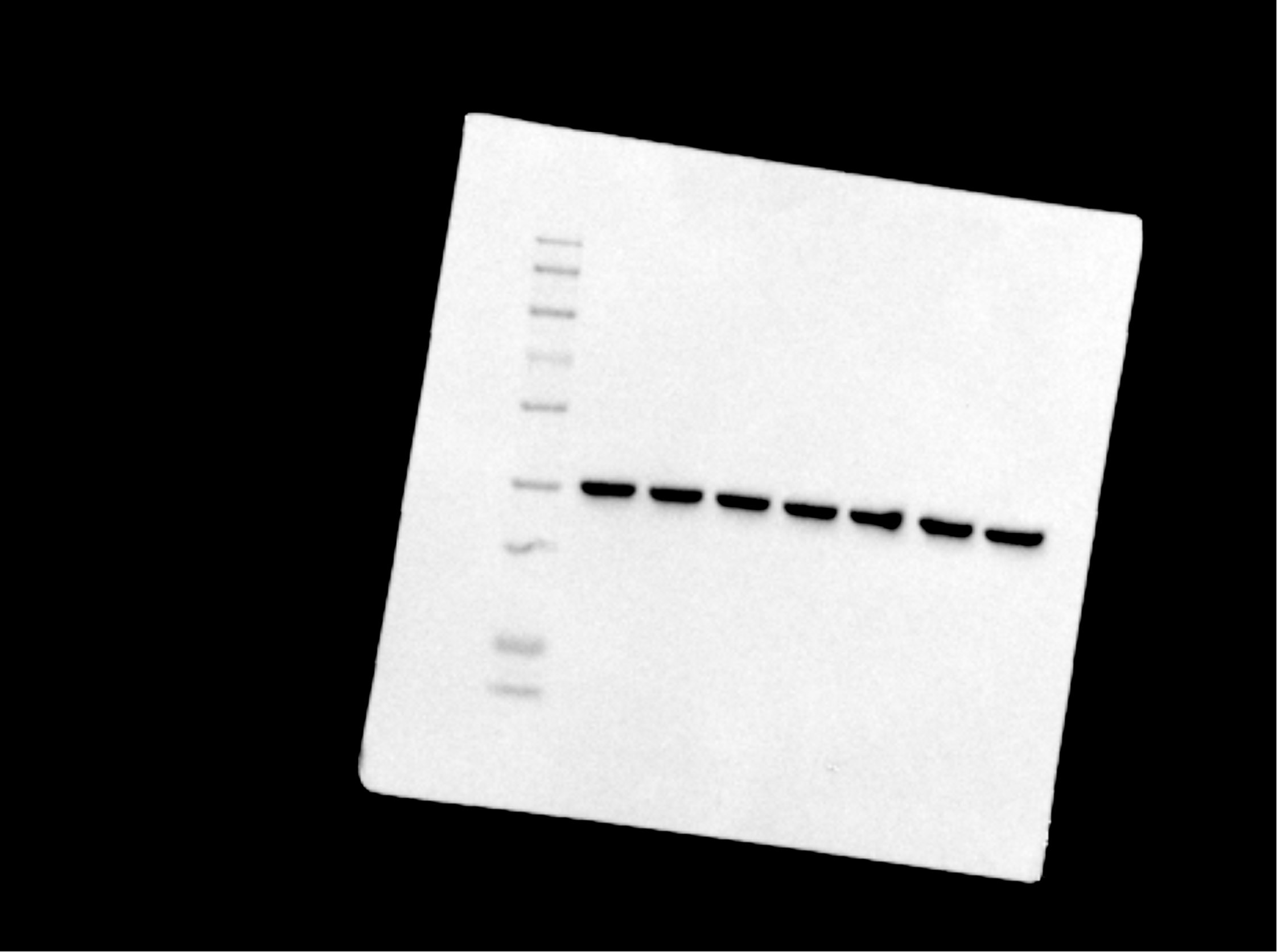


Figure 8D-1.jpg


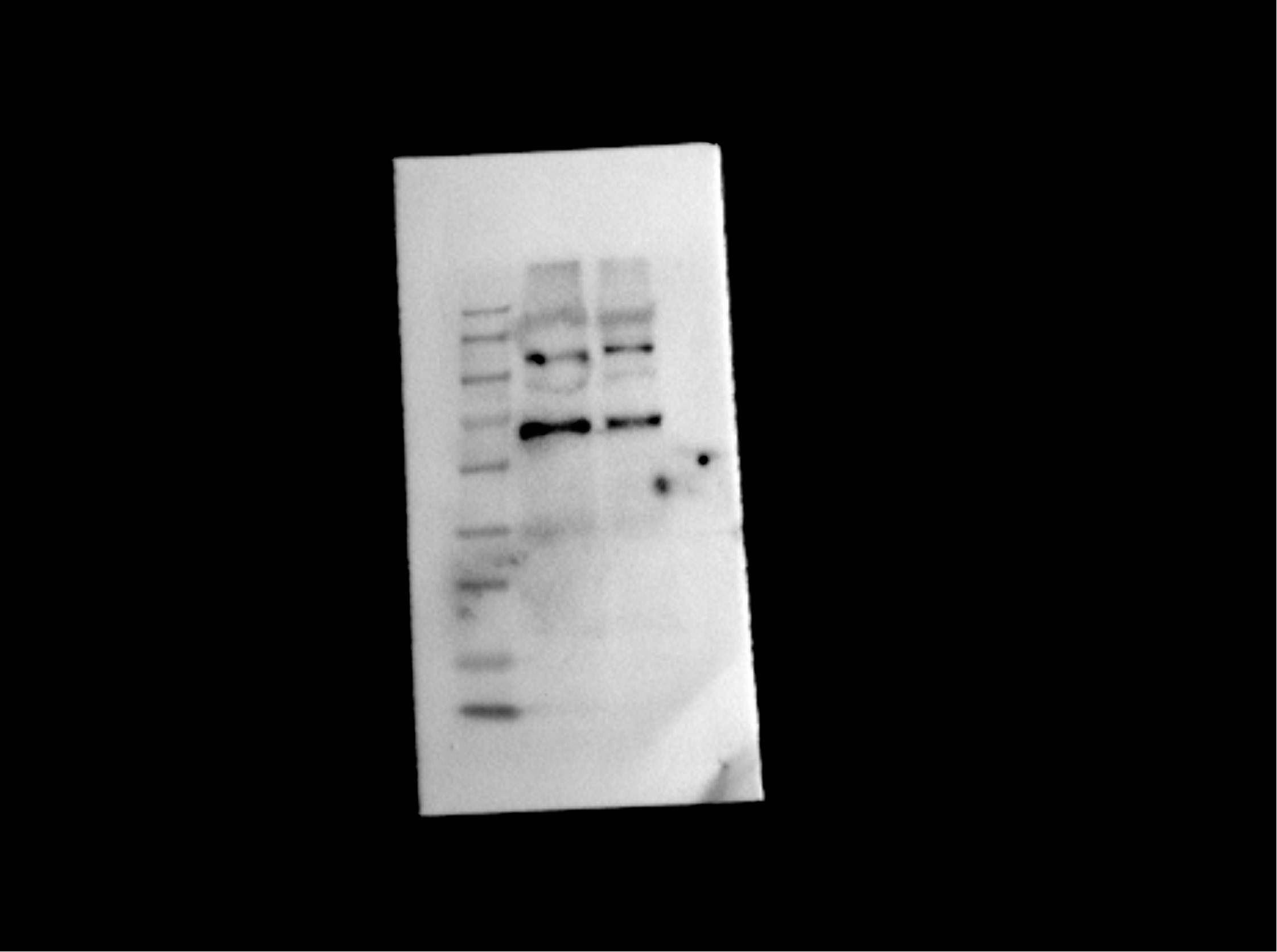


Figure 8D-2.jpg


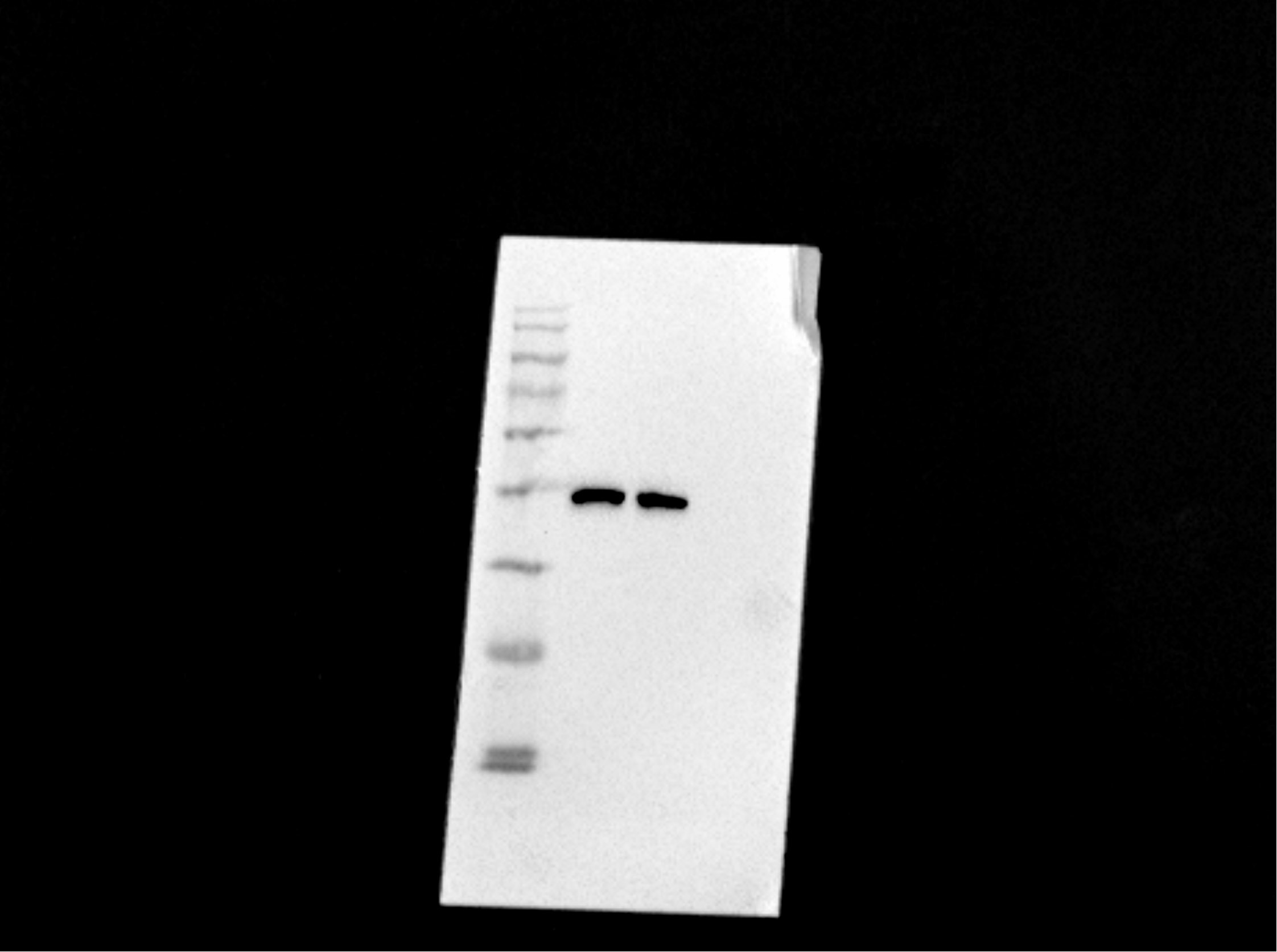


Figure 8F-1.jpg


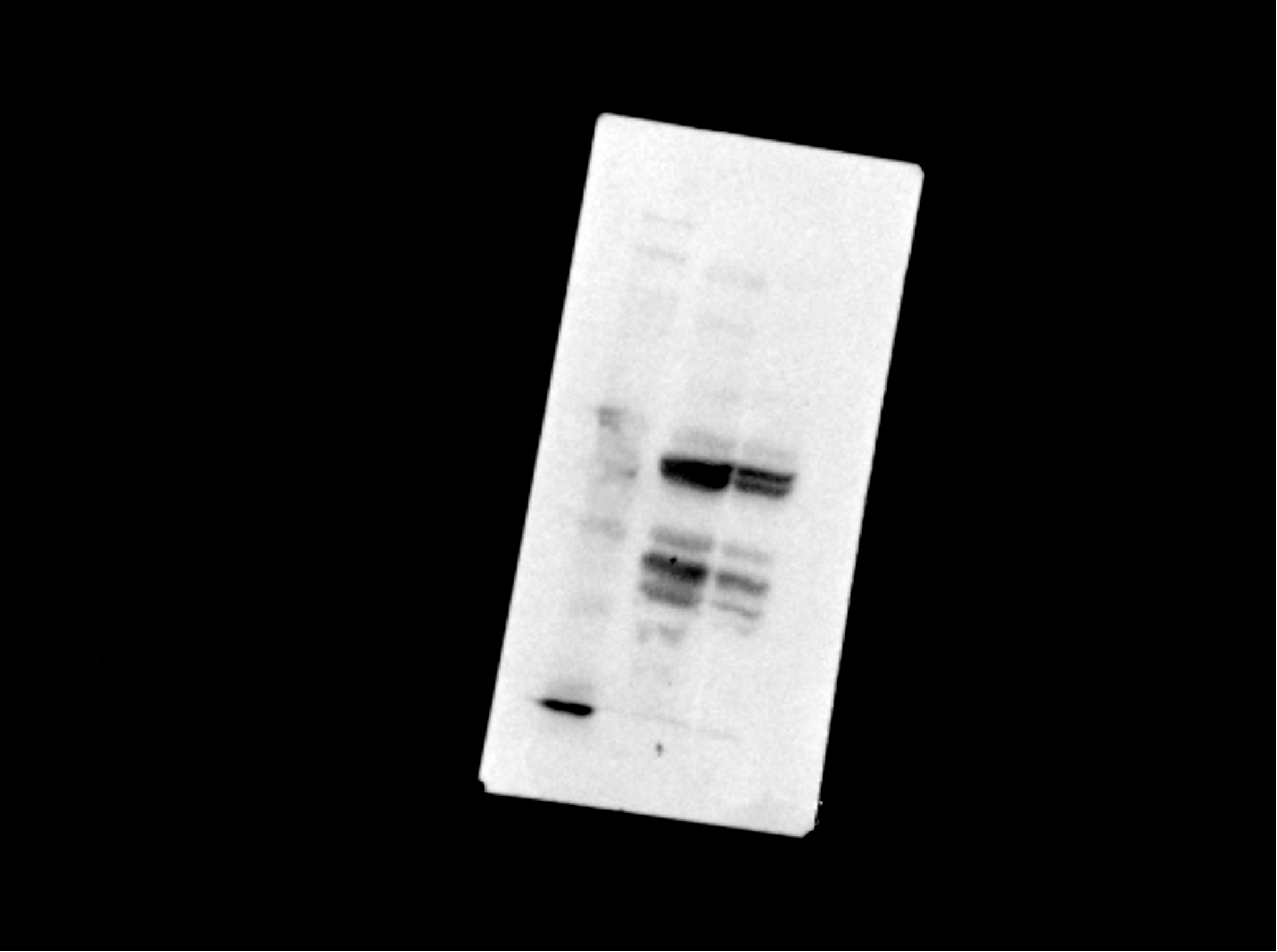


Figure 8F-2.jpg


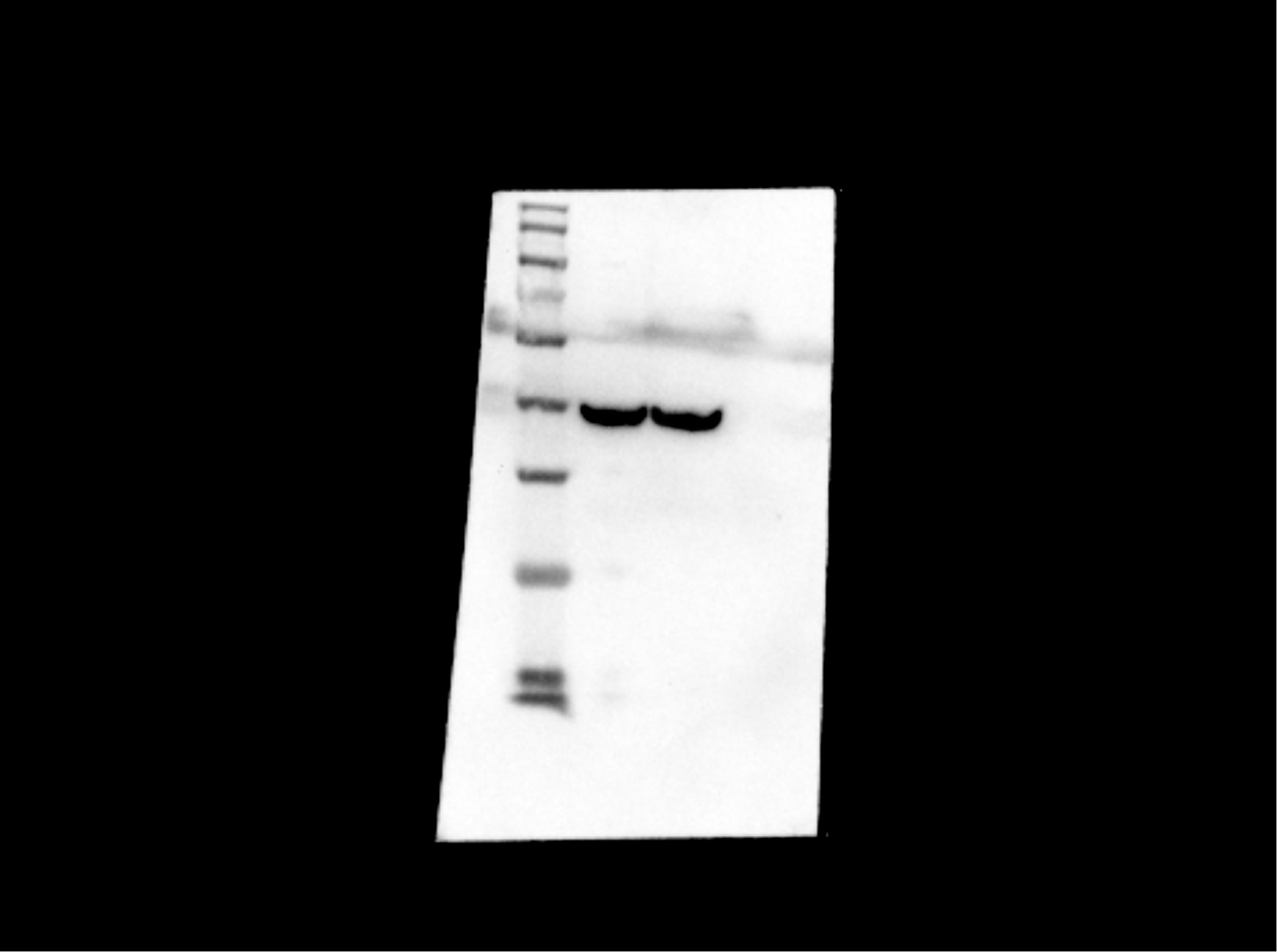


Figure S7B-1.jpg


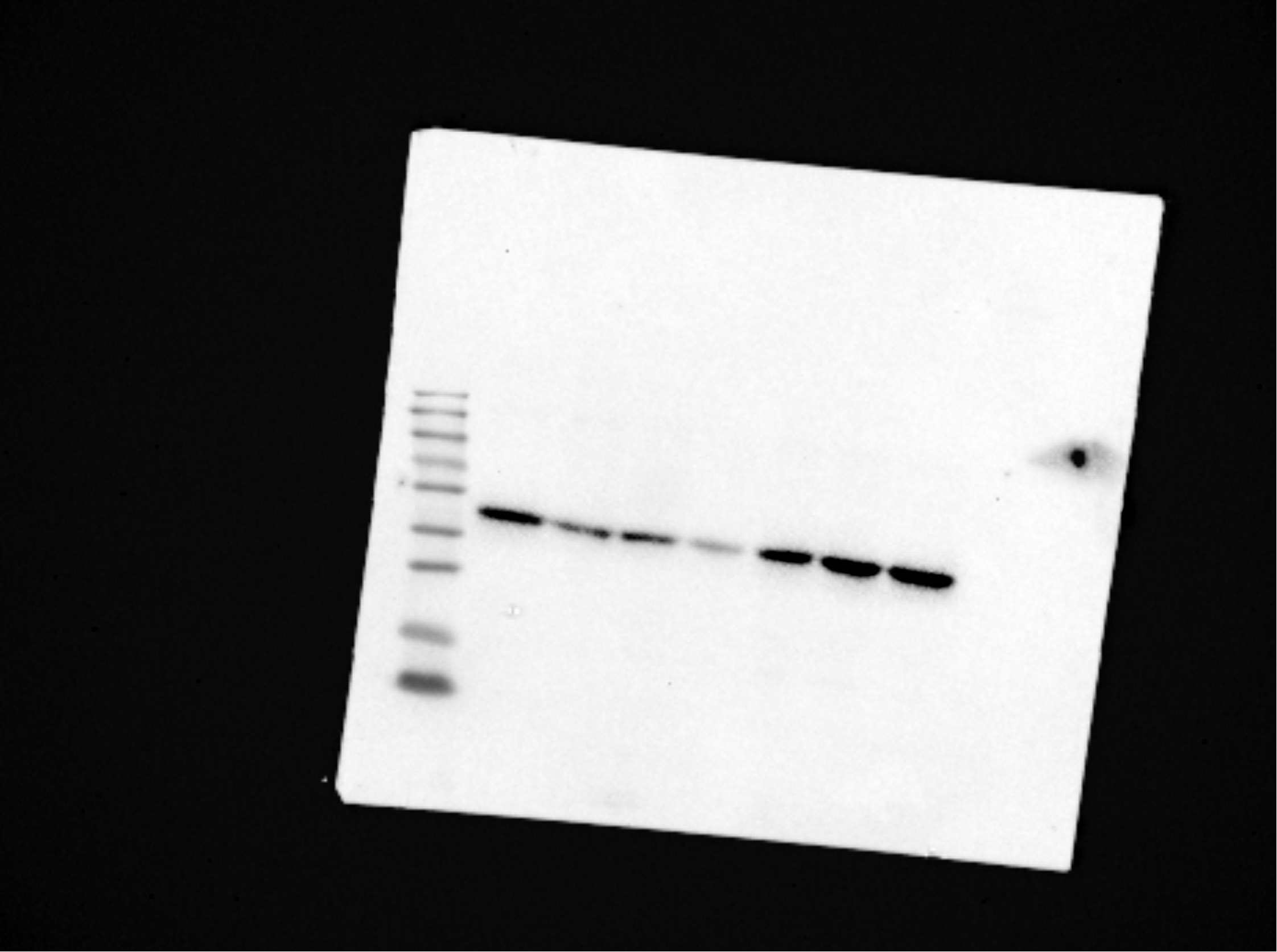


Figure S7B-2.jpg


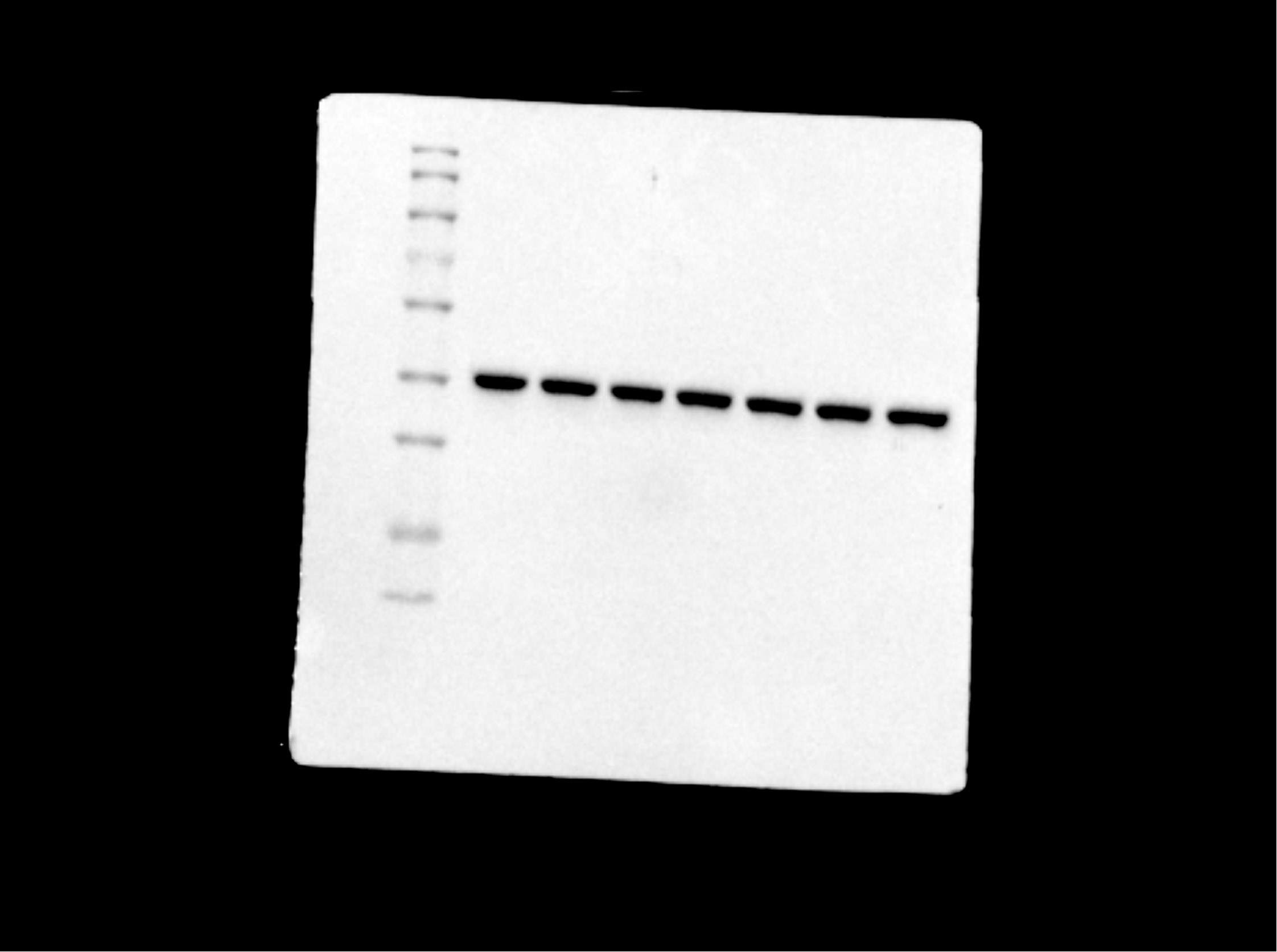

Supplement: Supplementary file 4 — Full and uncropped western blots [file 41420_2025_2768_MOESM4_ESM.docx]
